# Author Correction: Inferring experimental procedures from text-based representations of chemical reactions

**DOI:** 10.1038/s41467-024-53919-6

**Published:** 2024-11-18

**Authors:** Alain C. Vaucher, Philippe Schwaller, Joppe Geluykens, Vishnu H. Nair, Anna Iuliano, Teodoro Laino

**Affiliations:** 1 IBM Research Europe, Rüschlikon, Switzerland; 2 Dipartimento di Chimica e Chimica Industriale, Università di Pisa, Pisa, Italy

Correction to: *Nature Communications* 10.1038/s41467-021-22951-1, published online 06 May 2021

The competing interests statement reported no competing interests for the authors; this has been updated to “The authors are listed as inventors of a filed patent by IBM based on this work (application number 16995853).” This has been corrected in the PDF and HTML versions of this article.

