# Supplementary material for: Inferring experimental procedures from text-based representations of chemical reactions
Source: Nat Commun. 2021 May 6;12:2573. doi: 10.1038/s41467-021-22951-1 (PMC8102565; doi:10.1038/s41467-021-22951-1)
Supplement: Supplementary file 1 — Supplementary Information [file 41467_2021_22951_MOESM1_ESM.pdf]

Supplementary Information for
"Inferring experimental procedures from text-based representations of chemical reactions"

Alain C. Vaucher et al.

Supplementary Note 1: Action types

The action types used for expressing synthesis operations are summarized in the Supplementary Table 1. The properties associated to these actions are available as Supplementary Material in Ref. 1.

Supplementary Table 1: Action types for the prediction of experimental steps. This table is adapted from Ref. 1.

Action name	Description
`Add`	Add a substance to the reactor
`CollectLayer`	Select aqueous or organic fraction(s)
`Concentrate`	Evaporate the solvent (rotavap)
`Degas`	Purge the reaction mixture with a gas
`DrySolid`	Dry a solid
`DrySolution`	Dry an organic solution with a desiccant
`Extract`	Transfer compound into a different solvent
`Filter`	Separate solid and liquid phases
`MakeSolution`	Mix several substances to generate a mixture or solution
`Microwave`	Heat the reaction mixture in a microwave apparatus
`Partition`	Add two immiscible solvents for subsequent phase separation
`PH`	Change the pH of the reaction mixture
`PhaseSeparation`	Separate the aqueous and organic phases
`Purify`	Purify (chromatography)
`Quench`	Stop reaction by adding a substance
`Recrystallize`	Recrystallize a solid from a solvent or mixture of solvents
`Reflux`	Reflux the reaction mixture
`SetTemperature`	Change the temperature of the reaction mixture
`Sonicate`	Agitate the solution with sound waves
`Stir`	Stir the reaction mixture for a specified duration
`Triturate`	Triturate the residue
`Wait`	Leave the reaction mixture to stand for a specified duration
`Wash`	Wash (after filtration, or with immiscible solvent)
`Yield`	Phony action, indicates the product of a reaction

Supplementary Note 2: Class distribution of the data set splits

The Supplementary Figure 1 shows the distribution of the reaction superclasses (following the RXNO ontology[1]). The largest observable change resulting from the generation of the data set is in the superclass 4 (heterocycle forming reactions). The removal of duplicates, which results in a 44% size reduction of the data set, does not change the distribution noticeably. This suggests that duplicated reactions are almost equally distributed among the superclasses. Also, the distributions for the train, validation and test splits are nearly identical.

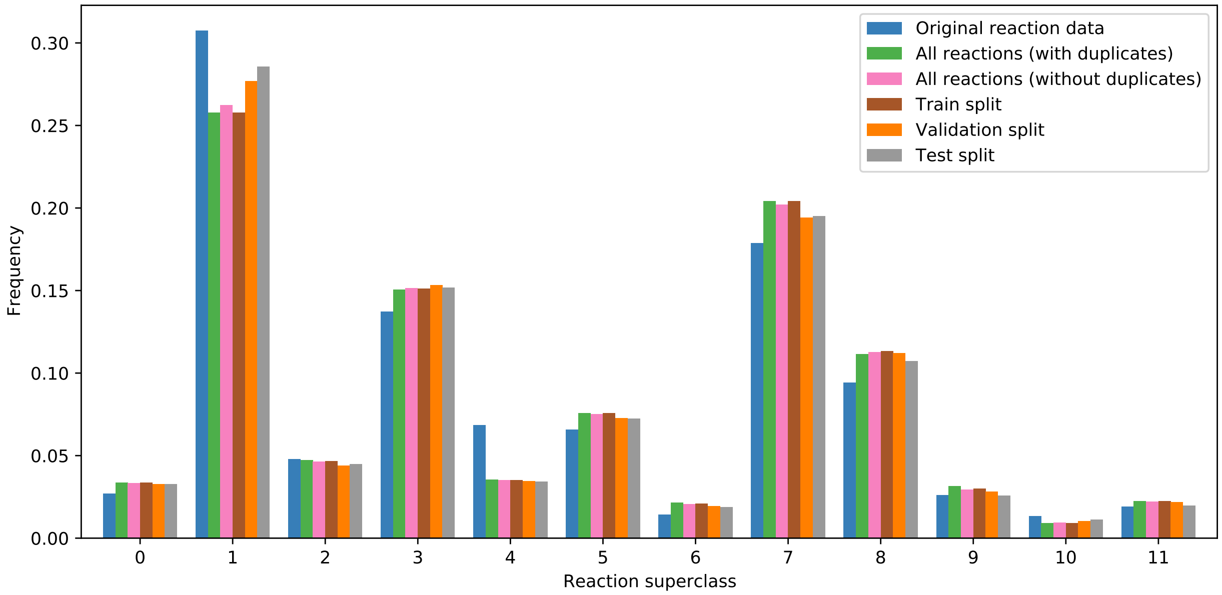

Supplementary Figure 1: Distribution of reaction superclasses for different subsets of the data. In addition to the original data set of 3,464,664 reactions, the distribution of the obtained dataset before and after removal of duplicate reaction SMILES is shown, as well as its division in train, validation, and test splits.

Considering not only the superclasses, 878 reaction classes are represented in the full data set of 693,517 reactions. Not all of these classes are covered in all of the splits. The train split does not cover 15 of these reaction classes, that have an average count of 1.27 in the original data set of 693,517 reactions. For the validation and test splits, the number of missing classes are 146 and 167, with an average count of 4.84 and 6.17, respectively. As this represents, on average, frequencies below 0.001% of the original dataset, the classes missing in the test set are likely not to have a considerable impact on the performance metrics.

[1] `https://github.com/rsc-ontologies/rxno/`, accessed Feb 2021.

Supplementary Note 3: Effect of shuffling molecules

For each of the 69,352 reactions in the test set, we shuffled the order of the molecules (one random molecule shuffling per reaction) to study the effect on the model predictions. In 7,922 cases (11.4%), the predicted actions are the identical and the placeholders are updated as expected from the new order of the molecules in the reaction SMILES. In 3,710 cases (5.3%), the actions are the same, but the placeholders are not updated correctly, which corresponds to a different addition order. In the 57,720 remaining cases (83.2%), different action sequences are suggested.

From these values, it appears that the model fails to recognize that reaction SMILES ordered differently should lead to the same action sequence. It is a sign that the model relies too much on the deterministic order of molecules in the training data (i.e. alphabetical), and it shows that the model should be used only with reactions SMILES processed accordingly. As such, the model is similar to an image recognition model that did not learn to recognize objects in images turned upside down. To be able to account for different orders of molecules in the future, the training data could be augmented to include reaction SMILES with molecules shuffled randomly.

Supplementary Note 4: Performance with respect to the reaction class

Using the 50%, 60%, 70%, 80%, 90% and 100% accuracy metrics as defined in the manuscript, we compared the performance of the model for the different reaction superclasses (following the RXNO ontology[2]). We show these metrics in the Supplementary Figure 2. The trends are similar for all the superclasses. On average, superclasses that perform better at the 100% accuracy metric tend to perform better also for other ones. The exception is the superclass 11 (resolution reactions), which has the highest 100% accuracy, but the lowest 50% accuracy score.

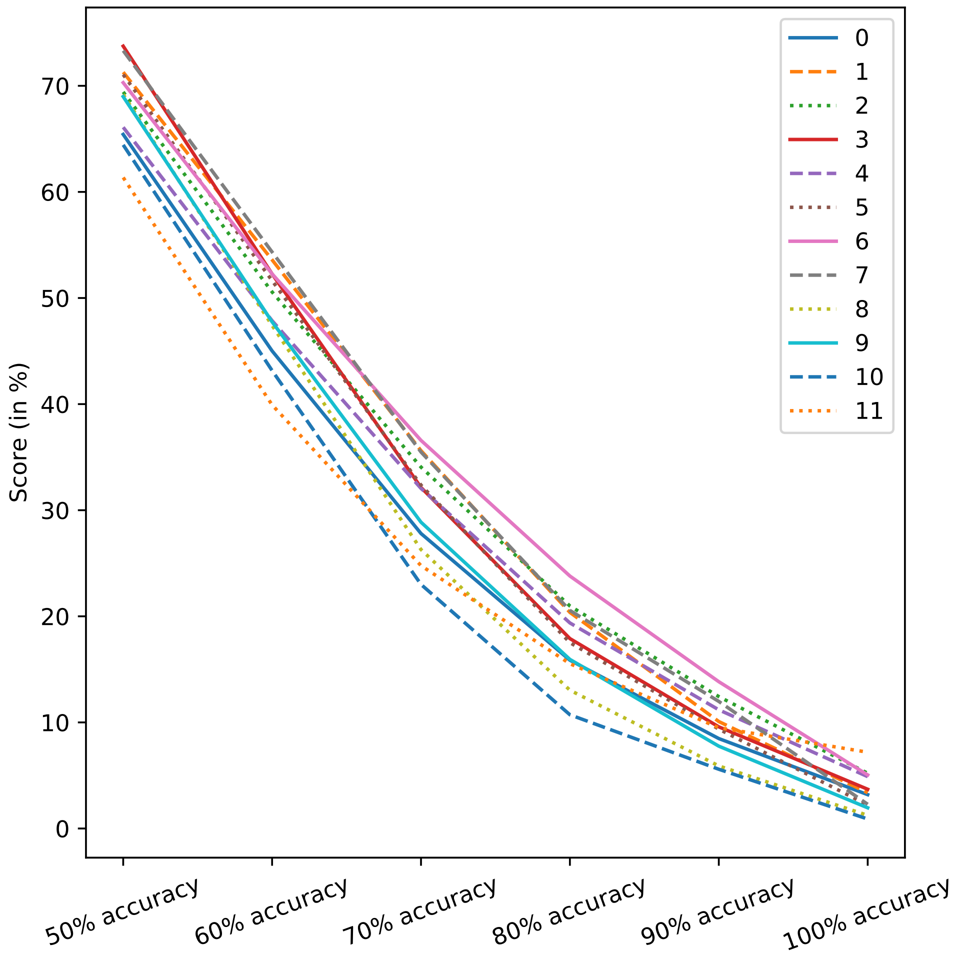

Supplementary Figure 2: Accuracy metrics for reactions from different superclasses.

[2]`https://github.com/rsc-ontologies/rxno/`, accessed Feb 2021.

Supplementary Note 5: Class distribution of the chemist-assessed reactions

The class distribution for the 500 reactions assessed by the expert chemist are is illustrated in the Supplementary Figure 3. The distribution follows closely the one of the full reaction data set of 693,517 reactions.

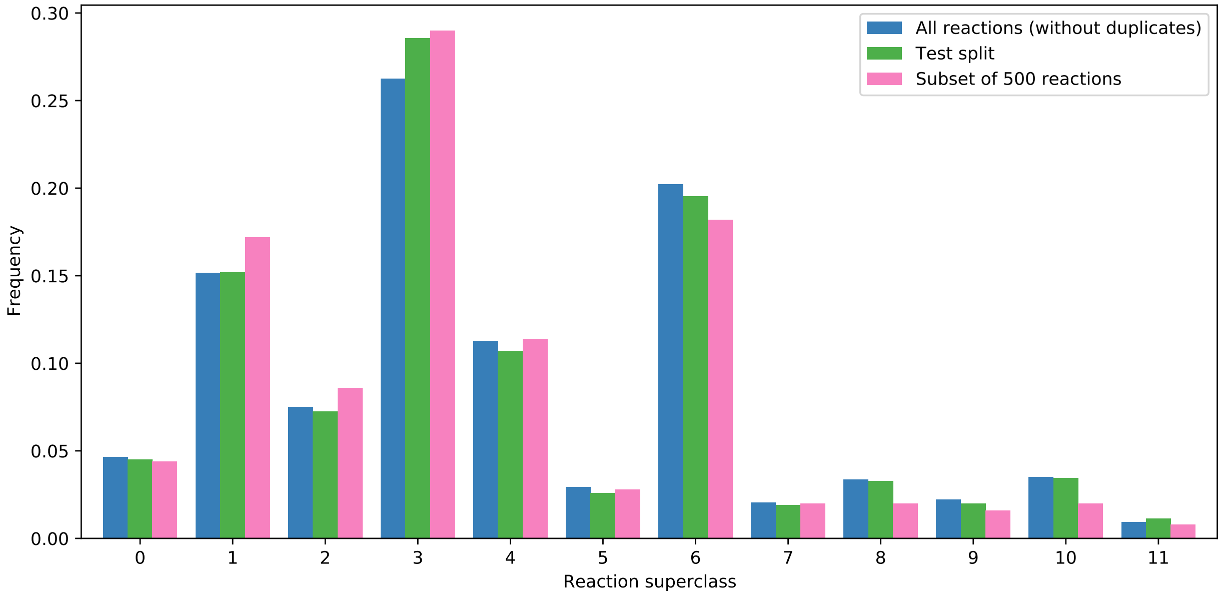

Supplementary Figure 3: Distribution of reaction superclasses for the subset of 500 reactions assessed by the expert chemist. The distributions for the full data set and the test splits correspond to the ones shown in the Supplementary Figure 1.

Supplementary Note 6: Tokenization of quantities

The Supplementary Tables 2, 3, and 4 contain the tokens with which the extracted temperatures, durations, and pH values are replaced, respectively, as well as the values used to convert the predicted instructions into numerical values.

Supplementary Table 2: Ranges for the tokenization of temperature values.

Value range	Token	Value at inference time
< −50 °C	#1#	−70 °C
−50 °C — −10 °C	#2#	−30 °C
−10 °C — +10 °C	#3#	0 °C
+10 °C — +40 °C	#4#	+25 °C
+40 °C — +80 °C	#5#	+60 °C
> +80 °C	#6#	+100 °C

Supplementary Table 3: Ranges for the tokenization of duration values.

Value range	Token	Value at inference time
< 0.5 h	@1@	10 min
0.5 h — 3 h	@2@	1 h
3 h — 10 h	@3@	8 h
10 h — 50 h	@4@	1 d
> 50 h	@5@	7 d

Supplementary Table 4: Ranges for the tokenization of pH values.

Value range	Token	Value at inference time
< 6.5	acidic	acidic
6.5 — 7.5	neutral	neutral
> 7.5	basic	basic

Supplementary Note 7: Generation of the list of common reagents

To generate the list of common reagents (Supplementary Data 1), we iterated through the chemical equations and associated experimental procedures and collected the compound names that were present in the extracted actions but absent from the chemical equation. The 190 compound names with a count of 60 or more were then manually reviewed. Compound names were removed in the following cases, leading to the list of 140 common reagents:

- mixtures of compounds;
- unspecific compounds ("product", "compound 52", "salt", etc.);
- optical character recognition errors (such as "INHCl");
- essential reagents that cannot be considered common and should be present in the chemical equation.

References

(1) Vaucher, A. C.; Zipoli, F.; Geluykens, J.; Nair, V. H.; Schwaller, P.; Laino, T. Automated extraction of chemical synthesis actions from experimental procedures. *Nat. Commun.* **2020**, *11*, 3601.
