# Supplementary material for: Inferring experimental procedures from text-based representations of chemical reactions
Source: Nat Commun. 2021 May 6;12:2573. doi: 10.1038/s41467-021-22951-1 (PMC8102565; doi:10.1038/s41467-021-22951-1)
Supplement: Supplementary file 3 — Description of Additional Supplementary Files [file 41467_2021_22951_MOESM3_ESM.pdf]

Description of Additional Supplementary Files

- **Supplementary Data 1**: List of compound names that are allowed in the action sequences even if they do not come up in the reaction equation.
- **Supplementary Data 2**: Examples of predicted action sequences.
- **Supplementary Data 3**: Chemist assessment of 500 reactions.
- **Supplementary Data 4**: Illustration of the variability of action sequences, exemplified at 293 reactions present multiple times in the original reaction data.
