# Supplementary material for: Inferring experimental procedures from text-based representations of chemical reactions
Source: Nat Commun. 2021 May 6;12:2573. doi: 10.1038/s41467-021-22951-1 (PMC8102565; doi:10.1038/s41467-021-22951-1)
Supplement: Supplementary file 6 — Supplementary Data 3 [file 41467_2021_22951_MOESM6_ESM.zip › 285354_2_data_set_5449725_qqqjk5.html]

actions\_pred\_vs\_truth


# Comparison of action sequences extracted from paragraphs and predicted by ML model¶

Further below you will find a few hundred reactions equations with two suggested action sequences, "A" and "B", to execute the reaction.

For each of the randomly-selected reactions from the test set, "A" and "B" are attributed randomly to:

- Actions extracted from the experimental procedure for these reactions (ground truth)
- Actions predicted by a ML model starting from the reaction equation (ML prediction)

A trained chemist then selected which of both was better, without knowing the ground truth. The chemist also had the possibility to state that none, or both, are adequate.

The results are reported at the end of this document.

---

```
Reaction no 1
```

Generated by the Chemistry Development Kit (http://github.com/cdk)

|  | A | B |
| --- | --- | --- |
| 0 | ADD tert-butyl 4-[(2-methyl-6-{[6-(4,4,5,5-tetramethyl-1,3,2-dioxaborolan-2-yl)-1H-benzimidazol-2-yl]amino}pyridin-4-yl)methyl]piperazine-1-carboxylate | ADD tert-butyl 4-[(2-methyl-6-{[6-(4,4,5,5-tetramethyl-1,3,2-dioxaborolan-2-yl)-1H-benzimidazol-2-yl]amino}pyridin-4-yl)methyl]piperazine-1-carboxylate |
| 1 | ADD 4-bromopyrimidine | ADD 4-bromopyrimidine |
| 2 | ADD dioxane | ADD dioxane |
| 3 | ADD water | ADD water |
| 4 | ADD Na2CO3 | ADD Na2CO3 |
| 5 | ADD Pd(dppf)Cl2 | ADD Pd(dppf)Cl2 |
| 6 | REFLUX for 86400 s | REFLUX for 86400 s |
| 7 | ADD dichloromethane | ADD 4-bromopyrimidine |
| 8 | DRYSOLUTION over magnesium sulfate | REFLUX for 28800 s |
| 9 | FILTER keep filtrate | ADD dichloromethane |
| 10 | CONCENTRATE | DRYSOLUTION over magnesium sulfate |
| 11 | YIELD tert-butyl 4-[(2-methyl-6-{[6-(pyrimidin-4-yl)-1H-benzimidazol-2-yl]amino}pyridin-4-yl)methyl]piperazine-1-carboxylate | FILTER keep filtrate |
| 12 |  | CONCENTRATE |
| 13 |  | PURIFY |
| 14 |  | YIELD tert-butyl 4-[(2-methyl-6-{[6-(pyrimidin-4-yl)-1H-benzimidazol-2-yl]amino}pyridin-4-yl)methyl]piperazine-1-carboxylate |

---

```
Reaction no 2
```

Generated by the Chemistry Development Kit (http://github.com/cdk)

|  | A | B |
| --- | --- | --- |
| 0 | ADD tert-butyl(1-(4-(6-chloro-5-nitro-3-phenylpyridin-2-yl)phenyl)cyclobutyl)carbamate | ADD tert-butyl(1-(4-(6-chloro-5-nitro-3-phenylpyridin-2-yl)phenyl)cyclobutyl)carbamate |
| 1 | ADD sarcosine methyl ester hydrochloride | ADD methanol |
| 2 | ADD triethylamine | ADD sarcosine methyl ester hydrochloride |
| 3 | ADD methanol | ADD triethylamine |
| 4 | MICROWAVE for 3600 s at 100 °C | STIR for 86400 s at 60 °C |
| 5 | CONCENTRATE | CONCENTRATE |
| 6 | PURIFY | PURIFY |
| 7 | YIELD 6-(4-(1-aminocyclobutyl)phenyl)-4-methyl-7-phenyl-3,4-dihydropyrido[2,3-b]pyrazin-2(1H)-one | YIELD 6-(4-(1-aminocyclobutyl)phenyl)-4-methyl-7-phenyl-3,4-dihydropyrido[2,3-b]pyrazin-2(1H)-one |

---

```
Reaction no 3
```

Generated by the Chemistry Development Kit (http://github.com/cdk)

|  | A | B |
| --- | --- | --- |
| 0 | ADD 4-(3-hydroxy-benzyl)-piperazine-1-carboxylic acid benzo[d]isoxazol-3-ylamide | ADD 4-(3-hydroxy-benzyl)-piperazine-1-carboxylic acid benzo[d]isoxazol-3-ylamide |
| 1 | ADD K2CO3 | ADD acetonitrile |
| 2 | ADD 2-(bromomethyl)benzonitrile | ADD K2CO3 |
| 3 | ADD acetonitrile | ADD 2-(bromomethyl)benzonitrile |
| 4 | STIR for 86400 s at 25 °C | STIR for 86400 s at 60 °C |
| 5 | ADD water | SETTEMPERATURE 25 °C |
| 6 | EXTRACT with ethyl acetate | ADD water |
| 7 | COLLECTLAYER organic | EXTRACT with ethyl acetate |
| 8 | WASH with brine | COLLECTLAYER organic |
| 9 | DRYSOLUTION over MgSO4 | DRYSOLUTION over Na2SO4 |
| 10 | FILTER keep filtrate | CONCENTRATE |
| 11 | CONCENTRATE | PURIFY |
| 12 | PURIFY | YIELD 4-[3-(2-Cyano-benzyloxy)-benzyl]-piperazine-1-carboxylic acid benzo[d]isoxazol-3-ylamide |
| 13 | YIELD 4-[3-(2-Cyano-benzyloxy)-benzyl]-piperazine-1-carboxylic acid benzo[d]isoxazol-3-ylamide |  |

---

```
Reaction no 4
```

Generated by the Chemistry Development Kit (http://github.com/cdk)

|  | A | B |
| --- | --- | --- |
| 0 | ADD ((4-(cyanoethynyl)phenyl)carbamoyl)(3-(4-(ethoxycarbonyl)phenyl)-1,2,3-oxadiazol-3-ium-5-yl)amide | ADD ((4-(cyanoethynyl)phenyl)carbamoyl)(3-(4-(ethoxycarbonyl)phenyl)-1,2,3-oxadiazol-3-ium-5-yl)amide |
| 1 | ADD ethanol | ADD ethanol |
| 2 | ADD THF | ADD THF |
| 3 | ADD water | ADD NaOH |
| 4 | ADD NaOH | STIR for 3600 s at 25 °C |
| 5 | STIR for 86400 s at 25 °C | ADD water |
| 6 | CONCENTRATE | EXTRACT with ethyl acetate |
| 7 | ADD water | COLLECTLAYER aqueous |
| 8 | PH with HCl to pH acidic | PH with HCl to pH acidic |
| 9 | EXTRACT with ethyl acetate | EXTRACT with ethyl acetate |
| 10 | COLLECTLAYER organic | COLLECTLAYER organic |
| 11 | WASH with brine | DRYSOLUTION over MgSO4 |
| 12 | DRYSOLUTION over sodium sulfate | CONCENTRATE |
| 13 | FILTER keep filtrate | YIELD (3-(4-carboxyphenyl)-1,2,3-oxadiazol-3-ium-5-yl)((4-(cyanoethynyl)phenyl)carbamoyl)amide |
| 14 | CONCENTRATE |  |
| 15 | YIELD (3-(4-carboxyphenyl)-1,2,3-oxadiazol-3-ium-5-yl)((4-(cyanoethynyl)phenyl)carbamoyl)amide |  |

---

```
Reaction no 5
```

Generated by the Chemistry Development Kit (http://github.com/cdk)

|  | A | B |
| --- | --- | --- |
| 0 | ADD methanol | ADD sodium thiomethoxide |
| 1 | ADD 2,3-epoxy-4-octyl-4-trimethylsilyloxycyclopentanone | ADD methanol |
| 2 | ADD triethylamine | SETTEMPERATURE 0 °C |
| 3 | ADD sodium thiomethoxide | STIR for 600 s |
| 4 | STIR for 3600 s at 25 °C | ADD triethylamine |
| 5 | ADD ammonium chloride | STIR for 600 s |
| 6 | EXTRACT with ethyl acetate | MAKESOLUTION with 2,3-epoxy-4-octyl-4-trimethylsilyloxycyclopentanone and methanol |
| 7 | COLLECTLAYER organic | ADD SLN dropwise |
| 8 | WASH with brine | STIR for 28800 s |
| 9 | DRYSOLUTION over magnesium sulfate | ADD ammonium chloride |
| 10 | CONCENTRATE | EXTRACT with ethyl acetate |
| 11 | PURIFY | COLLECTLAYER organic |
| 12 | YIELD 2-methylthio-4-hydroxy-4-octyl-2-cyclopentenone | WASH with sodium chloride |
| 13 |  | DRYSOLUTION over sodium sulfate |
| 14 |  | FILTER keep filtrate |
| 15 |  | CONCENTRATE |
| 16 |  | YIELD 2-methylthio-4-hydroxy-4-octyl-2-cyclopentenone |

---

```
Reaction no 6
```

Generated by the Chemistry Development Kit (http://github.com/cdk)

|  | A | B |
| --- | --- | --- |
| 0 | ADD 5-(4-Methoxyphenyl)-[1,3,4]oxathiazol-2-one | ADD 5-(4-Methoxyphenyl)-[1,3,4]oxathiazol-2-one |
| 1 | ADD p-xylene | ADD p-xylene |
| 2 | ADD ethyl cyanoformate | ADD ethyl cyanoformate |
| 3 | STIR for 86400 s at 100 °C | ADD dry ice |
| 4 | ADD dry ice | REFLUX for 86400 s |
| 5 | CONCENTRATE | CONCENTRATE |
| 6 | TRITURATE with diisopropyl ether | PURIFY |
| 7 | FILTER keep precipitate | YIELD ethyl 3-(4-methoxyphenyl)-[1,2,4]thiadiazole-5-carboxylate |
| 8 | WASH with diisopropyl ether |  |
| 9 | DRYSOLID |  |
| 10 | YIELD ethyl 3-(4-methoxyphenyl)-[1,2,4]thiadiazole-5-carboxylate |  |

---

```
Reaction no 7
```

Generated by the Chemistry Development Kit (http://github.com/cdk)

|  | A | B |
| --- | --- | --- |
| 0 | ADD 3-(azetidin-3-yl)-6-(4-chlorophenyl)-5-(3,8-dimethyl-[1,2,4]triazolo[4,3-a]pyridin-6-yl)-1-methyl-5,6-dihydropyrrolo[3,4-b]pyrrol-4(1H)-one | ADD 3-(azetidin-3-yl)-6-(4-chlorophenyl)-5-(3,8-dimethyl-[1,2,4]triazolo[4,3-a]pyridin-6-yl)-1-methyl-5,6-dihydropyrrolo[3,4-b]pyrrol-4(1H)-one |
| 1 | ADD dichloromethane | ADD triethylamine |
| 2 | ADD triethylamine | ADD acetic anhydride |
| 3 | ADD acetic anhydride | ADD dichloromethane |
| 4 | STIR for 3600 s at 25 °C | STIR for 3600 s at 25 °C |
| 5 | ADD water | ADD water |
| 6 | EXTRACT with ethyl acetate | EXTRACT with dichloromethane |
| 7 | COLLECTLAYER organic | COLLECTLAYER organic |
| 8 | WASH with water | WASH with brine |
| 9 | DRYSOLUTION over Na2SO4 | DRYSOLUTION over Na2SO4 |
| 10 | CONCENTRATE | FILTER keep filtrate |
| 11 | PURIFY | CONCENTRATE |
| 12 | YIELD 3-(1-acetylazetidin-3-yl)-6-(4-chlorophenyl)-5-(3,8-dimethyl-[1,2,4]triazolo[4,3-a]pyridin-6-yl)-1-methyl-5,6-dihydropyrrolo[3,4-b]pyrrol-4(1H)-one | PURIFY |
| 13 |  | YIELD 3-(1-acetylazetidin-3-yl)-6-(4-chlorophenyl)-5-(3,8-dimethyl-[1,2,4]triazolo[4,3-a]pyridin-6-yl)-1-methyl-5,6-dihydropyrrolo[3,4-b]pyrrol-4(1H)-one |

---

```
Reaction no 8
```

Generated by the Chemistry Development Kit (http://github.com/cdk)

|  | A | B |
| --- | --- | --- |
| 0 | ADD 3-ethyl-7-hydroxy-8-propylcoumarin | ADD 3-ethyl-7-hydroxy-8-propylcoumarin |
| 1 | ADD 1,3-dibromopropane | ADD K2CO3 |
| 2 | ADD K2CO3 | ADD DMF |
| 3 | ADD DMF | ADD 1,3-dibromopropane |
| 4 | STIR for 86400 s at 25 °C | STIR for 3600 s at 25 °C |
| 5 | ADD ammonium chloride | ADD ethyl acetate |
| 6 | ADD ethyl acetate | ADD ammonium chloride |
| 7 | PHASESEPARATION | PHASESEPARATION |
| 8 | COLLECTLAYER organic | COLLECTLAYER aqueous |
| 9 | COLLECTLAYER organic | EXTRACT with ethyl acetate |
| 10 | WASH with brine | COLLECTLAYER organic |
| 11 | DRYSOLUTION over magnesium sulfate | WASH with brine |
| 12 | CONCENTRATE | DRYSOLUTION over magnesium sulfate |
| 13 | PURIFY | FILTER keep filtrate |
| 14 | YIELD 7-(3-bromopropyl)oxy-3-ethyl-8-propylcoumarin | CONCENTRATE |
| 15 |  | PURIFY |
| 16 |  | YIELD 7-(3-bromopropyl)oxy-3-ethyl-8-propylcoumarin |

---

```
Reaction no 9
```

Generated by the Chemistry Development Kit (http://github.com/cdk)

|  | A | B |
| --- | --- | --- |
| 0 | ADD 3-((2,6-dichloro-7-fluoro-1-(1-propyl-1H-pyrazol-4-yl)-1H-indol-3-yl)thio)-2-fluorobenzoic acid | ADD 3-((2,6-dichloro-7-fluoro-1-(1-propyl-1H-pyrazol-4-yl)-1H-indol-3-yl)thio)-2-fluorobenzoic acid |
| 1 | ADD THF | ADD THF |
| 2 | ADD NaOH | SETTEMPERATURE 0 °C |
| 3 | CONCENTRATE | ADD NaOH dropwise over 3600 s |
| 4 | YIELD 3-((2,6-dichloro-7-fluoro-1-(1-propyl-1H-pyrazol-4-yl)-1H-indol-3-yl)thio)-2-fluorobenzoic acid, sodium salt | CONCENTRATE |
| 5 |  | DRYSOLID under vacuum |
| 6 |  | YIELD 3-((2,6-dichloro-7-fluoro-1-(1-propyl-1H-pyrazol-4-yl)-1H-indol-3-yl)thio)-2-fluorobenzoic acid, sodium salt |

---

```
Reaction no 10
```

Generated by the Chemistry Development Kit (http://github.com/cdk)

|  | A | B |
| --- | --- | --- |
| 0 | ADD 7-(4-tert-butoxycarbonylpiperazin-1-yl)benzofuran-5-carboxylic acid | ADD 7-(4-tert-butoxycarbonylpiperazin-1-yl)benzofuran-5-carboxylic acid |
| 1 | ADD dichloromethane | ADD n-butylamine |
| 2 | ADD 1-(3-dimethylaminopropyl)-3-ethylcarbodiimide hydrochloride at 0 °C | ADD 1-(3-dimethylaminopropyl)-3-ethylcarbodiimide hydrochloride |
| 3 | ADD n-butylamine at 0 °C | ADD Dimethylaminopyridin |
| 4 | ADD Dimethylaminopyridin at 25 °C | ADD dichloromethane |
| 5 | STIR for 86400 s at 25 °C | STIR for 86400 s at 25 °C |
| 6 | ADD ethyl acetate | ADD ethyl acetate |
| 7 | WASH with sodium hydroxide | WASH with water |
| 8 | WASH with water | WASH with citric acid |
| 9 | CONCENTRATE | WASH with NaHCO3 |
| 10 | PURIFY | WASH with brine |
| 11 | COLLECTLAYER organic | DRYSOLUTION over sodium sulfate |
| 12 | CONCENTRATE | CONCENTRATE |
| 13 | ADD TFA | ADD ethyl acetate |
| 14 | STIR for 3600 s at 25 °C | STIR for 86400 s at 25 °C |
| 15 | CONCENTRATE | FILTER keep precipitate |
| 16 | ADD HCl | WASH with ethyl acetate |
| 17 | YIELD N-[butyl] 7-(piperazin-1-yl)benzofuran-5-carboxamide dihydrochloride | YIELD N-[butyl] 7-(piperazin-1-yl)benzofuran-5-carboxamide dihydrochloride |

---

```
Reaction no 11
```

Generated by the Chemistry Development Kit (http://github.com/cdk)

|  | A | B |
| --- | --- | --- |
| 0 | ADD 2,3-dichloropyridine | ADD 2,3-dichloropyridine |
| 1 | ADD K2CO3 | ADD benzyl mercaptan |
| 2 | ADD DMSO | ADD K2CO3 |
| 3 | MAKESOLUTION with DMSO and benzyl mercaptan | ADD DMSO |
| 4 | ADD SLN dropwise at 100 °C over 600 s | STIR for 86400 s at 100 °C |
| 5 | STIR for 3600 s at 100 °C | SETTEMPERATURE 25 °C |
| 6 | ADD water | ADD water |
| 7 | EXTRACT with dichloromethane | EXTRACT with ethyl acetate |
| 8 | COLLECTLAYER organic | COLLECTLAYER organic |
| 9 | DRYSOLUTION over sodium sulfate | WASH with water |
| 10 | CONCENTRATE | WASH with brine |
| 11 | PURIFY | DRYSOLUTION over Na2SO4 |
| 12 | YIELD 2-benzylthio-3-chloropyridine | CONCENTRATE |
| 13 |  | PURIFY |
| 14 |  | YIELD 2-benzylthio-3-chloropyridine |

---

```
Reaction no 12
```

Generated by the Chemistry Development Kit (http://github.com/cdk)

|  | A | B |
| --- | --- | --- |
| 0 | ADD ethyl 5-chloro-6-(formamidomethyl)-4-methoxypicolinate | ADD ethyl 5-chloro-6-(formamidomethyl)-4-methoxypicolinate |
| 1 | ADD acetonitrile | ADD acetonitrile |
| 2 | ADD POCl3 | ADD POCl3 |
| 3 | STIR for 3600 s at 60 °C | STIR for 86400 s at 100 °C |
| 4 | SETTEMPERATURE 25 °C | CONCENTRATE |
| 5 | CONCENTRATE | ADD water |
| 6 | ADD NaHCO3 | PH with NaHCO3 to pH neutral |
| 7 | EXTRACT with ethyl acetate | EXTRACT with ethyl acetate |
| 8 | WASH with brine | COLLECTLAYER organic |
| 9 | DRYSOLUTION over Na2SO4 | WASH with brine |
| 10 | CONCENTRATE | DRYSOLUTION over Na2SO4 |
| 11 | PURIFY | CONCENTRATE |
| 12 | YIELD Ethyl 8-chloro-7-methoxyimidazo[1,5-a]pyridine-5-carboxylate | PURIFY |
| 13 |  | YIELD Ethyl 8-chloro-7-methoxyimidazo[1,5-a]pyridine-5-carboxylate |

---

```
Reaction no 13
```

Generated by the Chemistry Development Kit (http://github.com/cdk)

|  | A | B |
| --- | --- | --- |
| 0 | ADD ethanol | ADD 3-[2-acetyl-5-fluoro-3-(3-fluoro-benzyl)-benzofuran-7-yl]-pyrrole-1-carboxylic acid tert-butyl ester |
| 1 | ADD 3-[2-acetyl-5-fluoro-3-(3-fluoro-benzyl)-benzofuran-7-yl]-pyrrole-1-carboxylic acid tert-butyl ester | ADD ethanol |
| 2 | ADD platinum on carbon | ADD platinum on carbon |
| 3 | STIR for 3600 s at 60 °C | STIR for 28800 s at 25 °C |
| 4 | ADD platinum on carbon | FILTER keep filtrate |
| 5 | STIR for 28800 s at 60 °C | CONCENTRATE |
| 6 | FILTER keep filtrate | YIELD 3-[2-acetyl-5-fluoro-3-(3-fluoro-benzyl)-benzofuran-7-yl]-pyrrolidine-1-carboxylic acid tert-butyl ester |
| 7 | CONCENTRATE |  |
| 8 | PURIFY |  |
| 9 | YIELD 3-[2-acetyl-5-fluoro-3-(3-fluoro-benzyl)-benzofuran-7-yl]-pyrrolidine-1-carboxylic acid tert-butyl ester |  |

---

```
Reaction no 14
```

Generated by the Chemistry Development Kit (http://github.com/cdk)

|  | A | B |
| --- | --- | --- |
| 0 | ADD 4-(imidazo[1,2-a]pyridin-5-ylthio)butanoic [3-(1,1,6,8-tetraoxo-9-phenyl-2,3,4,8-tetrahydropyrimido[6,1-b][1,3]thiazin-7-yl)propyl]amide | ADD methanol |
| 1 | ADD methanol | ADD 4-(imidazo[1,2-a]pyridin-5-ylthio)butanoic [3-(1,1,6,8-tetraoxo-9-phenyl-2,3,4,8-tetrahydropyrimido[6,1-b][1,3]thiazin-7-yl)propyl]amide |
| 2 | ADD hydrogen chloride-ethyl acetate | ADD hydrogen chloride-ethyl acetate |
| 3 | CONCENTRATE | STIR |
| 4 | ADD ether | CONCENTRATE |
| 5 | FILTER keep precipitate | WASH with ether |
| 6 | YIELD 4-(imidazo[1,2-a]pyridin-5-ylthio)butanoic [3-(1,1,6,8-tetraoxo-9-phenyl-2,3,4,8-tetrahydropyrimido[6,1-b][1,3]thiazine-7-yl)propyl]amide hydrochloride | YIELD 4-(imidazo[1,2-a]pyridin-5-ylthio)butanoic [3-(1,1,6,8-tetraoxo-9-phenyl-2,3,4,8-tetrahydropyrimido[6,1-b][1,3]thiazine-7-yl)propyl]amide hydrochloride |

---

```
Reaction no 15
```

Generated by the Chemistry Development Kit (http://github.com/cdk)

|  | A | B |
| --- | --- | --- |
| 0 | ADD 6-(pyridin-4-yl)quinazolin-8-ol | ADD 6-(pyridin-4-yl)quinazolin-8-ol |
| 1 | ADD triethylamine | ADD triethylamine |
| 2 | ADD dichloromethane | ADD dichloromethane |
| 3 | ADD PhN(OTf)2 | ADD PhN(OTf)2 |
| 4 | STIR for 86400 s at 25 °C | STIR for 86400 s at 25 °C |
| 5 | CONCENTRATE | CONCENTRATE |
| 6 | PURIFY | PURIFY |
| 7 | YIELD 6-(pyridin-4-yl)quinazolin-8-yl trifluoromethanesulfonate | YIELD 6-(pyridin-4-yl)quinazolin-8-yl trifluoromethanesulfonate |

---

```
Reaction no 16
```

Generated by the Chemistry Development Kit (http://github.com/cdk)

|  | A | B |
| --- | --- | --- |
| 0 | ADD 4-(4-Chlorophenyl)-2-oxo-4-oxazoline | ADD 4-(4-Chlorophenyl)-2-oxo-4-oxazoline |
| 1 | ADD 3-penten-2-one | ADD methanol |
| 2 | ADD methanol | ADD 3-penten-2-one |
| 3 | ADD triethylamine | ADD triethylamine |
| 4 | REFLUX for 86400 s | REFLUX for 86400 s |
| 5 | CONCENTRATE | SETTEMPERATURE 25 °C |
| 6 | ADD diisopropyl ether | CONCENTRATE |
| 7 | FILTER keep precipitate | PURIFY |
| 8 | WASH with diisopropyl ether | YIELD 4-(4-Chlorophenyl)-5-(1-methyl-3-oxobutyl)-2-oxo-4-oxazoline |
| 9 | YIELD 4-(4-Chlorophenyl)-5-(1-methyl-3-oxobutyl)-2-oxo-4-oxazoline |  |

---

```
Reaction no 17
```

Generated by the Chemistry Development Kit (http://github.com/cdk)

|  | A | B |
| --- | --- | --- |
| 0 | ADD 4′-Chloro-3′-[[[(1-hydroxycycloheptyl)methyl]amino]carbonyl]-[1,1′-biphenyl]-2-carboxylic acid, ethyl ester | ADD 4′-Chloro-3′-[[[(1-hydroxycycloheptyl)methyl]amino]carbonyl]-[1,1′-biphenyl]-2-carboxylic acid, ethyl ester |
| 1 | ADD methanol | ADD methanol |
| 2 | ADD water | MAKESOLUTION with KOH and water |
| 3 | ADD KOH | ADD SLN |
| 4 | STIR for 86400 s at 25 °C | STIR for 600 s at 60 °C |
| 5 | CONCENTRATE | CONCENTRATE |
| 6 | ADD water | ADD water |
| 7 | PH with HCl to pH acidic | PH with HCl to pH acidic |
| 8 | EXTRACT with ethyl acetate | EXTRACT with dichloromethane |
| 9 | COLLECTLAYER organic | COLLECTLAYER organic |
| 10 | WASH with brine | CONCENTRATE |
| 11 | DRYSOLUTION over sodium sulfate | PURIFY |
| 12 | FILTER keep filtrate | YIELD 4′-Chloro-3′-[[[(1-hydroxycycloheptyl)methyl]amino]carbonyl]-[1,1′-biphenyl]-2-carboxylic acid |
| 13 | CONCENTRATE |  |
| 14 | YIELD 4′-Chloro-3′-[[[(1-hydroxycycloheptyl)methyl]amino]carbonyl]-[1,1′-biphenyl]-2-carboxylic acid |  |

---

```
Reaction no 18
```

Generated by the Chemistry Development Kit (http://github.com/cdk)

|  | A | B |
| --- | --- | --- |
| 0 | ADD Tert-butyl 4-(3-ethoxy-3-oxopropanoyl)-2-methylpiperidine-1-carboxylate | ADD Tert-butyl 4-(3-ethoxy-3-oxopropanoyl)-2-methylpiperidine-1-carboxylate |
| 1 | ADD 7-bromo-1H-indazol-3-amine | ADD 7-bromo-1H-indazol-3-amine |
| 2 | ADD K3PO4 | ADD K3PO4 |
| 3 | ADD 1-methoxy-2-propanol | ADD 1-methoxy-2-propanol |
| 4 | MICROWAVE for 600 s at 100 °C | MICROWAVE for 600 s at 100 °C |
| 5 | SETTEMPERATURE 25 °C | SETTEMPERATURE 25 °C |
| 6 | ADD water | ADD water |
| 7 | PH with HCl to pH neutral | PH with HCl to pH acidic |
| 8 | FILTER keep precipitate | EXTRACT with ethyl acetate |
| 9 | WASH with ethyl acetate | COLLECTLAYER organic |
| 10 | WASH with acetonitrile | WASH with brine |
| 11 | DRYSOLID for 86400 s at 60 °C | DRYSOLUTION over sodium sulfate |
| 12 | YIELD Tert-butyl 4-(7-bromo-2-oxo-1,2-dihydropyrimido[1,2-b]indazol-4-yl)piperidine-1-carboxylate | FILTER keep filtrate |
| 13 |  | CONCENTRATE |
| 14 |  | TRITURATE with acetonitrile |
| 15 |  | FILTER keep precipitate |
| 16 |  | WASH with acetonitrile |
| 17 |  | DRYSOLID for 86400 s at 60 °C |
| 18 |  | YIELD Tert-butyl 4-(7-bromo-2-oxo-1,2-dihydropyrimido[1,2-b]indazol-4-yl)piperidine-1-carboxylate |

---

```
Reaction no 19
```

Generated by the Chemistry Development Kit (http://github.com/cdk)

|  | A | B |
| --- | --- | --- |
| 0 | ADD 1-[3-(4-Bromobutyl)phenyl]-2-(4-methoxyphenyl)-6-methoxynaphthalene | ADD 1-[3-(4-Bromobutyl)phenyl]-2-(4-methoxyphenyl)-6-methoxynaphthalene |
| 1 | ADD DMF | ADD DMF |
| 2 | ADD piperidine | ADD piperidine |
| 3 | ADD K2CO3 | ADD K2CO3 |
| 4 | REFLUX for 3600 s | STIR for 86400 s at 100 °C |
| 5 | FILTER keep filtrate | CONCENTRATE |
| 6 | CONCENTRATE | PURIFY |
| 7 | ADD ethyl acetate | YIELD 1-[4-[3-[2-(4-Methoxyphenyl)-6-methoxynaphth-1-yl]phenyloxy]butyl]piperidine |
| 8 | EXTRACT with water |  |
| 9 | PHASESEPARATION |  |
| 10 | COLLECTLAYER aqueous |  |
| 11 | EXTRACT with ethyl acetate |  |
| 12 | COLLECTLAYER organic |  |
| 13 | WASH with brine |  |
| 14 | DRYSOLUTION over Na2SO4 |  |
| 15 | CONCENTRATE |  |
| 16 | YIELD 1-[4-[3-[2-(4-Methoxyphenyl)-6-methoxynaphth-1-yl]phenyloxy]butyl]piperidine |  |

---

```
Reaction no 20
```

Generated by the Chemistry Development Kit (http://github.com/cdk)

|  | A | B |
| --- | --- | --- |
| 0 | ADD N-((1S,2S)-2-Aminocyclopentyl)-5-(2-methyl-4-phenoxyphenyl)-4-oxo-4,5-dihydro-3H-1-thia-3,5,8-triazaacenaphthylene-2-carboxamide | ADD N-((1S,2S)-2-Aminocyclopentyl)-5-(2-methyl-4-phenoxyphenyl)-4-oxo-4,5-dihydro-3H-1-thia-3,5,8-triazaacenaphthylene-2-carboxamide |
| 1 | ADD formaldehyde | ADD dichloromethane |
| 2 | ADD dichloromethane | ADD formaldehyde |
| 3 | REFLUX for 86400 s | STIR for 86400 s at 25 °C |
| 4 | FILTER keep precipitate | ADD dichloromethane |
| 5 | WASH with dichloromethane | WASH with NaHCO3 |
| 6 | COLLECTLAYER organic | COLLECTLAYER organic |
| 7 | CONCENTRATE | DRYSOLUTION over MgSO4 |
| 8 | PURIFY | FILTER keep filtrate |
| 9 | YIELD 5-(2-Methyl-4-phenoxyphenyl)-N-((1S,2S)-2-(methylamino)cyclopentyl)-4-oxo-4,5-dihydro-3H-1-thia-3,5,8-triazaacenaphthylene-2-carboxamide | CONCENTRATE |
| 10 |  | PURIFY |
| 11 |  | YIELD 5-(2-Methyl-4-phenoxyphenyl)-N-((1S,2S)-2-(methylamino)cyclopentyl)-4-oxo-4,5-dihydro-3H-1-thia-3,5,8-triazaacenaphthylene-2-carboxamide |

---

```
Reaction no 21
```

Generated by the Chemistry Development Kit (http://github.com/cdk)

|  | A | B |
| --- | --- | --- |
| 0 | ADD 5-(3-(6-cyclopropylpyrazin-2-yl)-1-tosyl-1H-indol-5-yl)-N-(4-methoxybenzyl)-1,3,4-oxadiazol-2-amine | ADD 5-(3-(6-cyclopropylpyrazin-2-yl)-1-tosyl-1H-indol-5-yl)-N-(4-methoxybenzyl)-1,3,4-oxadiazol-2-amine |
| 1 | ADD dioxane | ADD NaOH |
| 2 | ADD NaOH | ADD dioxane |
| 3 | STIR for 600 s at 100 °C | STIR for 3600 s at 100 °C |
| 4 | QUENCH with TFA | CONCENTRATE |
| 5 | ADD methanol | ADD DMSO |
| 6 | PURIFY | FILTER keep filtrate |
| 7 | YIELD 5-(3-(6-cyclopropylpyrazin-2-yl)-1H-indol-5-yl)-N-(4-methoxybenzyl)-1,3,4-oxadiazol-2-amine 2,2,2-trifluoroacetate | PURIFY |
| 8 |  | YIELD 5-(3-(6-cyclopropylpyrazin-2-yl)-1H-indol-5-yl)-N-(4-methoxybenzyl)-1,3,4-oxadiazol-2-amine 2,2,2-trifluoroacetate |

---

```
Reaction no 22
```

Generated by the Chemistry Development Kit (http://github.com/cdk)

|  | A | B |
| --- | --- | --- |
| 0 | ADD 3(R)-[1(S)-ethoxycarbonyl-3-cyclohexylpropyl]amino-4-oxo-2,3,4,5-tetrahydro-1,5-benzothiazepine-5-acetic acid hydrochloride | ADD methanol |
| 1 | ADD methanol | ADD 3(R)-[1(S)-ethoxycarbonyl-3-cyclohexylpropyl]amino-4-oxo-2,3,4,5-tetrahydro-1,5-benzothiazepine-5-acetic acid hydrochloride |
| 2 | ADD NaOH | ADD NaOH |
| 3 | STIR for 3600 s at 25 °C | WAIT for 3600 s at 25 °C |
| 4 | CONCENTRATE | CONCENTRATE |
| 5 | ADD water | PH with HCl to pH acidic |
| 6 | PH with HCl to pH acidic | YIELD 3(R)-[1(S)-carboxy-3-cyclohexylpropyl]amino-4-oxo-2,3,4,5-tetrahydro-1,5-benzothiazepine-5-acetic acid |
| 7 | FILTER keep precipitate |  |
| 8 | WASH with water |  |
| 9 | DRYSOLID under vacuum |  |
| 10 | YIELD 3(R)-[1(S)-carboxy-3-cyclohexylpropyl]amino-4-oxo-2,3,4,5-tetrahydro-1,5-benzothiazepine-5-acetic acid |  |

---

```
Reaction no 23
```

Generated by the Chemistry Development Kit (http://github.com/cdk)

|  | A | B |
| --- | --- | --- |
| 0 | ADD 3-[(morpholin-4-ylacetyl)amino]-4-(trifluoromethoxy)benzoic acid | ADD 3-[(morpholin-4-ylacetyl)amino]-4-(trifluoromethoxy)benzoic acid |
| 1 | ADD DMF | ADD 5-(5-amino-1,3,4-thiadiazol-2-yl)pyrimidin-2-amine |
| 2 | ADD PyBOP | ADD DIPEA |
| 3 | ADD DIPEA | ADD PyBOP |
| 4 | ADD 5-(5-amino-1,3,4-thiadiazol-2-yl)pyrimidin-2-amine | ADD DMF |
| 5 | STIR for 86400 s at 25 °C | STIR for 604800 s at 25 °C |
| 6 | CONCENTRATE | CONCENTRATE |
| 7 | PURIFY | PURIFY |
| 8 | YIELD N-[5-(2-aminopyrimidin-5-yl)-1,3,4-thiadiazol-2-yl]-3-({[1-(morpholin-4-yl)cyclopropyl]carbonyl}amino)-4-(trifluoromethoxy)benzamide | YIELD N-[5-(2-aminopyrimidin-5-yl)-1,3,4-thiadiazol-2-yl]-3-({[1-(morpholin-4-yl)cyclopropyl]carbonyl}amino)-4-(trifluoromethoxy)benzamide |

---

```
Reaction no 24
```

Generated by the Chemistry Development Kit (http://github.com/cdk)

|  | A | B |
| --- | --- | --- |
| 0 | ADD 3-(5-([1,1′-biphenyl]-4-yl)-6-chloro-1-((2-(trimethylsilyl)ethoxy)methyl)-1H-pyrrolo[3,2-b]pyridin-2-yl)benzoic acid | ADD 3-(5-([1,1′-biphenyl]-4-yl)-6-chloro-1-((2-(trimethylsilyl)ethoxy)methyl)-1H-pyrrolo[3,2-b]pyridin-2-yl)benzoic acid |
| 1 | ADD THF | ADD THF |
| 2 | ADD TBAF | ADD TBAF |
| 3 | ADD ethylenediamine | STIR for 86400 s at 60 °C |
| 4 | REFLUX for 28800 s | SETTEMPERATURE 25 °C |
| 5 | SETTEMPERATURE 25 °C | ADD ethylenediamine |
| 6 | ADD water | STIR for 86400 s at 25 °C |
| 7 | EXTRACT with ethyl acetate | ADD water |
| 8 | COLLECTLAYER organic | EXTRACT with ethyl acetate |
| 9 | DRYSOLUTION over magnesium sulfate | COLLECTLAYER organic |
| 10 | FILTER keep filtrate | WASH with water |
| 11 | CONCENTRATE | WASH with brine |
| 12 | PURIFY | DRYSOLUTION over sodium sulfate |
| 13 | YIELD 3-(5-([1,1′-biphenyl]-4-yl)-6-chloro-1H-pyrrolo[3,2-b]pyridin-2-yl)benzoic acid | FILTER keep filtrate |
| 14 |  | CONCENTRATE |
| 15 |  | PURIFY |
| 16 |  | YIELD 3-(5-([1,1′-biphenyl]-4-yl)-6-chloro-1H-pyrrolo[3,2-b]pyridin-2-yl)benzoic acid |

---

```
Reaction no 25
```

Generated by the Chemistry Development Kit (http://github.com/cdk)

|  | A | B |
| --- | --- | --- |
| 0 | ADD 1-((1-(2-fluorophenyl)propyl)amino)-2-methylpropan-2-ol | ADD 1-((1-(2-fluorophenyl)propyl)amino)-2-methylpropan-2-ol |
| 1 | ADD DMSO | ADD DMSO |
| 2 | ADD potassium t-butoxide at 25 °C | ADD potassium t-butoxide |
| 3 | STIR for 86400 s at 100 °C | STIR for 86400 s at 100 °C |
| 4 | SETTEMPERATURE 25 °C | SETTEMPERATURE 25 °C |
| 5 | ADD ice water | QUENCH with water |
| 6 | EXTRACT with ethyl acetate | EXTRACT with ethyl acetate |
| 7 | COLLECTLAYER organic | COLLECTLAYER organic |
| 8 | CONCENTRATE | WASH with brine |
| 9 | PURIFY | DRYSOLUTION over sodium sulfate |
| 10 | CONCENTRATE | FILTER keep filtrate |
| 11 | YIELD 5-ethyl-2,2-dimethyl-2,3,4,5-tetrahydrobenzo[f][1,4]oxazepine | CONCENTRATE |
| 12 |  | YIELD 5-ethyl-2,2-dimethyl-2,3,4,5-tetrahydrobenzo[f][1,4]oxazepine |

---

```
Reaction no 26
```

Generated by the Chemistry Development Kit (http://github.com/cdk)

|  | A | B |
| --- | --- | --- |
| 0 | ADD 4-Amino-4-methoxycarbonylmethyl-piperidine-1-carboxylic acid benzyl ester | ADD 4-Amino-4-methoxycarbonylmethyl-piperidine-1-carboxylic acid benzyl ester |
| 1 | ADD 2,3-dihydro-benzo[1,4]dioxine-6-carbaldehyde | ADD 2,3-dihydro-benzo[1,4]dioxine-6-carbaldehyde |
| 2 | ADD 1,2-dichloroethane | ADD 1,2-dichloroethane |
| 3 | ADD sodium triacetoxyborohydride | ADD sodium triacetoxyborohydride |
| 4 | STIR for 86400 s at 25 °C | STIR for 86400 s at 25 °C |
| 5 | ADD dichloromethane | ADD NaHCO3 |
| 6 | ADD NaHCO3 | ADD dichloromethane |
| 7 | PHASESEPARATION | PHASESEPARATION |
| 8 | COLLECTLAYER aqueous | COLLECTLAYER aqueous |
| 9 | EXTRACT with dichloromethane | EXTRACT with dichloromethane |
| 10 | COLLECTLAYER organic | COLLECTLAYER organic |
| 11 | DRYSOLUTION over MgSO4 | WASH with sodium chloride |
| 12 | FILTER keep filtrate | DRYSOLUTION over sodium sulfate |
| 13 | CONCENTRATE | FILTER keep filtrate |
| 14 | PURIFY | CONCENTRATE |
| 15 | YIELD 4-[(2,3-Dihydro-benzo[1,4]dioxin-6-ylmethyl)-amino]-4-methoxycarbonylmethyl-piperidine-1-carboxylic acid benzyl ester | PURIFY |
| 16 |  | YIELD 4-[(2,3-Dihydro-benzo[1,4]dioxin-6-ylmethyl)-amino]-4-methoxycarbonylmethyl-piperidine-1-carboxylic acid benzyl ester |

---

```
Reaction no 27
```

Generated by the Chemistry Development Kit (http://github.com/cdk)

|  | A | B |
| --- | --- | --- |
| 0 | ADD phloroglucinol monobutylthio-ether | ADD phloroglucinol monobutylthio-ether |
| 1 | ADD 3-n-butylthio-6a,7,10,10a-tetrahydro-6,6,9-trimethyl-6H-dibenzo[b,d]pyran-1-ol | ADD benzene |
| 2 | ADD p-toluenesulfonic acid monohydrate | ADD p-toluenesulfonic acid monohydrate |
| 3 | ADD benzene | REFLUX for 28800 s with Dean-Stark apparatus |
| 4 | REFLUX for 28800 s | SETTEMPERATURE 25 °C |
| 5 | SETTEMPERATURE 25 °C | WASH with sodium bicarbonate |
| 6 | ADD water | WASH with water |
| 7 | FILTER keep filtrate | DRYSOLUTION over magnesium sulfate |
| 8 |  | CONCENTRATE |
| 9 |  | RECRYSTALLIZE from benzene |
| 10 |  | YIELD 3-n-butylthio-6a,7,10,10a-tetrahydro-6,6,9-trimethyl-6H-dibenzo[b,d]pyran-1-ol |

---

```
Reaction no 28
```

Generated by the Chemistry Development Kit (http://github.com/cdk)

|  | A | B |
| --- | --- | --- |
| 0 | ADD N[4-(t-Butoxycarbamoyl)phenyl]-N-phenyl-4-methyl-1-piperazineacetamide | ADD N[4-(t-Butoxycarbamoyl)phenyl]-N-phenyl-4-methyl-1-piperazineacetamide |
| 1 | ADD TFA DCM | ADD TFA DCM |
| 2 | STIR for 3600 s at 25 °C | SETTEMPERATURE 0 °C |
| 3 | CONCENTRATE | STIR for 3600 s |
| 4 | YIELD N-4-aminophenyl-N-phenyl-4-methyl-1-piperazineacetamide | CONCENTRATE |
| 5 |  | PARTITION with NaHCO3 and ethyl acetate |
| 6 |  | PHASESEPARATION |
| 7 |  | COLLECTLAYER organic |
| 8 |  | DRYSOLUTION over MgSO4 |
| 9 |  | CONCENTRATE |
| 10 |  | YIELD N-4-aminophenyl-N-phenyl-4-methyl-1-piperazineacetamide |

---

```
Reaction no 29
```

Generated by the Chemistry Development Kit (http://github.com/cdk)

|  | A | B |
| --- | --- | --- |
| 0 | ADD Orcinol monohydrate | ADD Orcinol monohydrate |
| 1 | ADD 2-chlorobenzenesulfonyl chloride | ADD 2-chlorobenzenesulfonyl chloride |
| 2 | ADD NaHCO3 | ADD NaHCO3 |
| 3 | ADD ether | ADD ether |
| 4 | STIR for 86400 s at 25 °C | STIR for 86400 s at 25 °C |
| 5 | ADD water | ADD water |
| 6 | EXTRACT with ethyl acetate | EXTRACT with ethyl acetate |
| 7 | COLLECTLAYER organic | COLLECTLAYER organic |
| 8 | WASH with brine | WASH with brine |
| 9 | DRYSOLUTION over Na2SO4 | DRYSOLUTION over Na2SO4 |
| 10 | CONCENTRATE | CONCENTRATE |
| 11 | PURIFY | PURIFY |
| 12 | YIELD 2-chlorobenzenesulfonic acid 3-hydroxy-5-methylphenyl ester | YIELD 2-chlorobenzenesulfonic acid 3-hydroxy-5-methylphenyl ester |

---

```
Reaction no 30
```

Generated by the Chemistry Development Kit (http://github.com/cdk)

|  | A | B |
| --- | --- | --- |
| 0 | ADD 1-(cyclopentoxy)-3-fluoro-2-nitro-benzene | ADD 1-(cyclopentoxy)-3-fluoro-2-nitro-benzene |
| 1 | ADD methanol | ADD methanol |
| 2 | ADD Pd/C | ADD Pd/C |
| 3 | STIR for 3600 s at 25 °C | STIR for 86400 s at 25 °C |
| 4 | FILTER keep filtrate | FILTER keep filtrate |
| 5 | WASH with methanol | CONCENTRATE |
| 6 | CONCENTRATE | YIELD 2-(cyclopentoxy)-6-fluoro-aniline |
| 7 | YIELD 2-(cyclopentoxy)-6-fluoro-aniline |  |

---

```
Reaction no 31
```

Generated by the Chemistry Development Kit (http://github.com/cdk)

|  | A | B |
| --- | --- | --- |
| 0 | ADD Agmatine sulfate | ADD Agmatine sulfate |
| 1 | ADD isopropanol water | ADD isopropanol water |
| 2 | ADD NaOH | ADD NaOH at 25 °C |
| 3 | ADD hexadecyl chloroformate | ADD hexadecyl chloroformate at 25 °C |
| 4 | STIR for 3600 s at 25 °C | PH with NaOH to pH basic |
| 5 | ADD ethyl acetate | STIR for 3600 s at 25 °C |
| 6 | PHASESEPARATION | STIR for 28800 s at 25 °C |
| 7 | COLLECTLAYER organic | FILTER keep precipitate |
| 8 | WASH with water | WASH with isopropanol water |
| 9 | WASH with brine | ADD ethyl acetate |
| 10 | DRYSOLUTION over sodium sulfate | ADD hydrogen chloride-ethyl acetate |
| 11 | CONCENTRATE | STIR for 3600 s at 25 °C |
| 12 | ADD ethyl acetate | CONCENTRATE |
| 13 | ADD hydrogen chloride-ethyl acetate | CONCENTRATE |
| 14 | CONCENTRATE | ADD ethyl acetate |
| 15 | RECRYSTALLIZE from methanol | FILTER keep precipitate |
| 16 | YIELD Hexadecyl N-(4-guanidinobutyl)carbamate hydrochloride | YIELD Hexadecyl N-(4-guanidinobutyl)carbamate hydrochloride |

---

```
Reaction no 32
```

Generated by the Chemistry Development Kit (http://github.com/cdk)

|  | A | B |
| --- | --- | --- |
| 0 | ADD 1,1-Dimethylethyl cis-4-[[(3,4-dichlorophenoxy)carbonyl]amino]-5-oxo-1-phenyl-3-pyrrolidinecarboxylate | ADD 1,1-Dimethylethyl cis-4-[[(3,4-dichlorophenoxy)carbonyl]amino]-5-oxo-1-phenyl-3-pyrrolidinecarboxylate |
| 1 | ADD TFA | ADD TFA |
| 2 | STIR for 3600 s at 25 °C | STIR for 86400 s at 25 °C |
| 3 | CONCENTRATE | WAIT for 86400 s at 25 °C |
| 4 | ADD water | CONCENTRATE |
| 5 | FILTER keep precipitate | PURIFY |
| 6 | WASH with water | YIELD cis-4-[[(3,4-Dichlorophenoxy)carbonyl]amino]-5-oxo-1-phenyl-3-pyrrolidinecarboxylic acid |
| 7 | DRYSOLID |  |
| 8 | YIELD cis-4-[[(3,4-Dichlorophenoxy)carbonyl]amino]-5-oxo-1-phenyl-3-pyrrolidinecarboxylic acid |  |

---

```
Reaction no 33
```

Generated by the Chemistry Development Kit (http://github.com/cdk)

|  | A | B |
| --- | --- | --- |
| 0 | ADD Cs2CO3 | ADD 4-(6-chloro-1-isopropyl-1H-pyrazolo[4,3-c]pyridin-3-yl)thiomorpholine 1,1-dioxide |
| 1 | ADD X-Phos | ADD 2-(1-cyclopropanesulfonyl-1H-pyrazol-4-yl)pyrimidin-4-ylamine |
| 2 | ADD Pd2(dba)3 | ADD Pd2(dba)3 |
| 3 | ADD 4-(6-chloro-1-isopropyl-1H-pyrazolo[4,3-c]pyridin-3-yl)thiomorpholine 1,1-dioxide | ADD X-Phos |
| 4 | ADD 2-(1-cyclopropanesulfonyl-1H-pyrazol-4-yl)pyrimidin-4-ylamine | ADD Cs2CO3 |
| 5 | ADD dioxane | ADD dioxane |
| 6 | STIR for 86400 s at 100 °C | STIR for 86400 s at 100 °C |
| 7 | SETTEMPERATURE 25 °C | CONCENTRATE |
| 8 | FILTER keep filtrate | PURIFY |
| 9 | CONCENTRATE | YIELD 4-(6-((2-(1-(cyclopropylsulfonyl)-1H-pyrazol-4-yl)pyrimidin-4-yl)amino)-1-isopropyl-1H-pyrazolo[4,3-c]pyridin-3-yl)thiomorpholine 1,1-dioxide |
| 10 | PURIFY |  |
| 11 | YIELD 4-(6-((2-(1-(cyclopropylsulfonyl)-1H-pyrazol-4-yl)pyrimidin-4-yl)amino)-1-isopropyl-1H-pyrazolo[4,3-c]pyridin-3-yl)thiomorpholine 1,1-dioxide |  |

---

```
Reaction no 34
```

Generated by the Chemistry Development Kit (http://github.com/cdk)

|  | A | B |
| --- | --- | --- |
| 0 | ADD ethyldicyanopropionate | ADD 3-(benzyloxy)benzenediazonium tetrafluoroborate |
| 1 | ADD methanol | ADD ethyldicyanopropionate |
| 2 | ADD pyridine | ADD pyridine |
| 3 | SETTEMPERATURE 0 °C | ADD methanol |
| 4 | ADD 3-(benzyloxy)benzenediazonium tetrafluoroborate over 3600 s | STIR for 86400 s at 25 °C |
| 5 | STIR for 86400 s at 25 °C | CONCENTRATE |
| 6 | CONCENTRATE | TRITURATE with water |
| 7 | ADD dichloromethane | FILTER keep precipitate |
| 8 | ADD potassium carbonate | WASH with water |
| 9 | STIR for 28800 s at 25 °C | DRYSOLID |
| 10 | COLLECTLAYER organic | YIELD 5-amino-1-(3-(benzyloxy)phenyl)-1H-pyrazole-3-carbonitrile |
| 11 | DRYSOLUTION over MgSO4 |  |
| 12 | CONCENTRATE |  |
| 13 | PURIFY |  |
| 14 | YIELD 5-amino-1-(3-(benzyloxy)phenyl)-1H-pyrazole-3-carbonitrile |  |

---

```
Reaction no 35
```

Generated by the Chemistry Development Kit (http://github.com/cdk)

|  | A | B |
| --- | --- | --- |
| 0 | ADD 1-(3-bromo-5-methoxyphenyl)thiourea | ADD 1-(3-bromo-5-methoxyphenyl)thiourea |
| 1 | ADD chloroform | ADD chloroform |
| 2 | SETTEMPERATURE -70 °C | ADD bromine dropwise |
| 3 | MAKESOLUTION with bromine and chloroform | REFLUX for 3600 s |
| 4 | ADD SLN | SETTEMPERATURE 25 °C |
| 5 | STIR for 600 s at 25 °C | PH with ammonium hydroxide to pH basic |
| 6 | STIR for 3600 s at 60 °C | FILTER keep precipitate |
| 7 | WAIT for 3600 s at 60 °C | WASH with water |
| 8 | PH with ammonium hydroxide to pH basic | DRYSOLID |
| 9 | EXTRACT with ethyl acetate | YIELD 7-bromo-5-methoxybenzo[d]thiazol-2-amine |
| 10 | COLLECTLAYER organic |  |
| 11 | WASH with brine |  |
| 12 | DRYSOLUTION over MgSO4 |  |
| 13 | FILTER keep filtrate |  |
| 14 | CONCENTRATE |  |
| 15 | YIELD 7-bromo-5-methoxybenzo[d]thiazol-2-amine |  |

---

```
Reaction no 36
```

Generated by the Chemistry Development Kit (http://github.com/cdk)

|  | A | B |
| --- | --- | --- |
| 0 | ADD 1-(4-{isopropyl-[2-(p-nitrobenzyloxyamino)ethyl]-carbamoyl}-1,3-thiazol-2-yl)-3-methanesulfonyloxyazetidine | ADD 1-(4-{isopropyl-[2-(p-nitrobenzyloxyamino)ethyl]-carbamoyl}-1,3-thiazol-2-yl)-3-methanesulfonyloxyazetidine |
| 1 | ADD DMF | ADD DMF |
| 2 | ADD potassium thioacetate at 25 °C | ADD potassium thioacetate at 25 °C |
| 3 | STIR for 28800 s at 100 °C | STIR for 28800 s at 100 °C |
| 4 | PARTITION with ethyl acetate and sodium chloride | PARTITION with ethyl acetate and sodium chloride |
| 5 | COLLECTLAYER organic | COLLECTLAYER organic |
| 6 | WASH with NaHCO3 | WASH with NaHCO3 |
| 7 | WASH with sodium chloride | WASH with sodium chloride |
| 8 | DRYSOLUTION over sodium sulfate | DRYSOLUTION over sodium sulfate |
| 9 | FILTER keep filtrate | FILTER keep filtrate |
| 10 | CONCENTRATE | CONCENTRATE |
| 11 | PURIFY | PURIFY |
| 12 | YIELD 3-acetylthio-1-(4-{isopropyl-[2-(p-nitrobenzyloxyamino)ethyl]-carbamoyl)-1,3-thiazol-2-yl)azetidine | YIELD 3-acetylthio-1-(4-{isopropyl-[2-(p-nitrobenzyloxyamino)ethyl]-carbamoyl)-1,3-thiazol-2-yl)azetidine |

---

```
Reaction no 37
```

Generated by the Chemistry Development Kit (http://github.com/cdk)

|  | A | B |
| --- | --- | --- |
| 0 | ADD 1-fluoro-4-nitrobenzene | ADD tert-butyl 4-(2-aminoethyl)-1,3-thiazol-2-ylcarbamate |
| 1 | ADD 1,3-dimethyl-2-imidazolidinone | ADD 1-fluoro-4-nitrobenzene |
| 2 | ADD tert-butyl 4-(2-aminoethyl)-1,3-thiazol-2-ylcarbamate | ADD triethylamine |
| 3 | ADD triethylamine | ADD 1,3-dimethyl-2-imidazolidinone |
| 4 | STIR for 86400 s at 25 °C | STIR for 28800 s at 60 °C |
| 5 | ADD water | SETTEMPERATURE 25 °C |
| 6 | FILTER keep precipitate | ADD water |
| 7 | WASH with water | EXTRACT with ethyl acetate |
| 8 | DRYSOLID under vacuum | COLLECTLAYER organic |
| 9 | YIELD tert-butyl 4-[2-(4-nitroanilino)ethyl]-1,3-thiazol-2-ylcarbamate | WASH with brine |
| 10 |  | DRYSOLUTION over magnesium sulfate |
| 11 |  | FILTER keep filtrate |
| 12 |  | CONCENTRATE |
| 13 |  | PURIFY |
| 14 |  | YIELD tert-butyl 4-[2-(4-nitroanilino)ethyl]-1,3-thiazol-2-ylcarbamate |

---

```
Reaction no 38
```

Generated by the Chemistry Development Kit (http://github.com/cdk)

|  | A | B |
| --- | --- | --- |
| 0 | ADD 2-(2-(2-(2-azidoethoxy)ethoxy)ethoxy)ethanamine | ADD 2-(2-(2-(2-azidoethoxy)ethoxy)ethoxy)ethanamine |
| 1 | ADD DMF | ADD DMF |
| 2 | ADD di-tert-butyl dicarbonate | ADD di-tert-butyl dicarbonate |
| 3 | STIR for 3600 s at 25 °C | STIR for 86400 s at 25 °C |
| 4 | CONCENTRATE | CONCENTRATE |
| 5 | PURIFY | PURIFY |
| 6 | YIELD tert-Butyl ‌(2-(2-(2-(2-azidoethoxy)ethoxy)ethoxy)ethyl)carbamate | YIELD tert-Butyl ‌(2-(2-(2-(2-azidoethoxy)ethoxy)ethoxy)ethyl)carbamate |

---

```
Reaction no 39
```

Generated by the Chemistry Development Kit (http://github.com/cdk)

|  | A | B |
| --- | --- | --- |
| 0 | ADD 5-(4-Chlorophenyl)-1-(2,4-dichlorophenyl)-4-methyl-1H-pyrazole-3-carboxamide | ADD 5-(4-Chlorophenyl)-1-(2,4-dichlorophenyl)-4-methyl-1H-pyrazole-3-carboxamide |
| 1 | ADD THF | ADD THF |
| 2 | ADD NaHMDS at -70 °C | ADD NaHMDS at -70 °C |
| 3 | STIR for 600 s | STIR for 600 s at -70 °C |
| 4 | ADD 2-ethylbutyryl chloride | ADD 2-ethylbutyryl chloride |
| 5 | ADD THF dropwise | STIR for 3600 s at -70 °C |
| 6 | STIR for 3600 s | ADD NaHCO3 |
| 7 | STIR for 86400 s at 25 °C | EXTRACT with ethyl acetate |
| 8 | ADD NaHCO3 | COLLECTLAYER organic |
| 9 | EXTRACT with ethyl acetate | WASH with brine |
| 10 | COLLECTLAYER organic | DRYSOLUTION over Na2SO4 |
| 11 | WASH with water | FILTER keep filtrate |
| 12 | DRYSOLUTION over MgSO4 | CONCENTRATE |
| 13 | CONCENTRATE | PURIFY |
| 14 | PURIFY | YIELD 5-(4-Chlorophenyl)-1-(2,4-dichlorophenyl)-N-(2-ethylbutanoyl)-4-methyl-1H-pyrazole-3-carboxamide |
| 15 | YIELD 5-(4-Chlorophenyl)-1-(2,4-dichlorophenyl)-N-(2-ethylbutanoyl)-4-methyl-1H-pyrazole-3-carboxamide |  |

---

```
Reaction no 40
```

Generated by the Chemistry Development Kit (http://github.com/cdk)

|  | A | B |
| --- | --- | --- |
| 0 | ADD 2-(2-(2-(benzyloxy)ethoxy)-4-chlorophenyl)-1-(5-(trifluoromethoxy)-1H-indol-3-yl)ethanone | ADD 2-(2-(2-(benzyloxy)ethoxy)-4-chlorophenyl)-1-(5-(trifluoromethoxy)-1H-indol-3-yl)ethanone |
| 1 | ADD ethyl acetate | ADD Pd/C |
| 2 | ADD THF | ADD ethyl acetate |
| 3 | ADD Pd/C | ADD THF |
| 4 | STIR for 86400 s at 25 °C | STIR for 600 s at 25 °C |
| 5 | FILTER keep filtrate | FILTER keep precipitate |
| 6 | CONCENTRATE | WASH with THF |
| 7 | PURIFY | CONCENTRATE |
| 8 | YIELD 2-(4-chloro-2-(2-hydroxyethoxy)phenyl)-1-(5-(trifluoromethoxy)-1H-indol-3-yl)ethanone | COLLECTLAYER organic |
| 9 |  | ADD dichloromethane |
| 10 |  | FILTER keep precipitate |
| 11 |  | WASH with dichloromethane |
| 12 |  | DRYSOLID at 60 °C under vacuum |
| 13 |  | YIELD 2-(4-chloro-2-(2-hydroxyethoxy)phenyl)-1-(5-(trifluoromethoxy)-1H-indol-3-yl)ethanone |

---

```
Reaction no 41
```

Generated by the Chemistry Development Kit (http://github.com/cdk)

|  | A | B |
| --- | --- | --- |
| 0 | ADD (2S,3S,4R,5R)-5-[2-Chloro-6-(2,2-diphenyl-ethylamino)-purin-9-yl]-3,4-dihydroxy-tetrahydro-furan-2-carboxylic acid ethylamide | ADD (2S,3S,4R,5R)-5-[2-Chloro-6-(2,2-diphenyl-ethylamino)-purin-9-yl]-3,4-dihydroxy-tetrahydro-furan-2-carboxylic acid ethylamide |
| 1 | ADD histamine | ADD histamine |
| 2 | ADD DMSO | ADD DMSO |
| 3 | STIR for 86400 s at 100 °C | STIR for 3600 s at 100 °C |
| 4 | SETTEMPERATURE 25 °C | SETTEMPERATURE 25 °C |
| 5 | ADD ethyl acetate | ADD methanol |
| 6 | WASH with water | PH with HCl to pH acidic |
| 7 | COLLECTLAYER aqueous | ADD ethyl acetate |
| 8 | EXTRACT with ethyl acetate | FILTER keep precipitate |
| 9 | COLLECTLAYER organic | DRYSOLID |
| 10 | WASH with water | YIELD 1-Deoxy-1-[6-[(2,2-diphenylethyl)amino]-2-[[2-(1H-imidazol-4-yl)ethyl]amino]-9H-purin-9-yl]-N-ethyl-β-D-ribofuranuronamide hydrochloride salt |
| 11 | DRYSOLUTION over MgSO4 |  |
| 12 | CONCENTRATE |  |
| 13 | MAKESOLUTION with HCl and methanol |  |
| 14 | ADD SLN |  |
| 15 | CONCENTRATE |  |
| 16 | YIELD 1-Deoxy-1-[6-[(2,2-diphenylethyl)amino]-2-[[2-(1H-imidazol-4-yl)ethyl]amino]-9H-purin-9-yl]-N-ethyl-β-D-ribofuranuronamide hydrochloride salt |  |

---

```
Reaction no 42
```

Generated by the Chemistry Development Kit (http://github.com/cdk)

|  | A | B |
| --- | --- | --- |
| 0 | ADD 1-Azabicyclo[2.2.2]oct-2-ene-3-carboxaldehyde | ADD 1-Azabicyclo[2.2.2]oct-2-ene-3-carboxaldehyde |
| 1 | ADD hydroxylamine hydrochloride | ADD methanol |
| 2 | ADD methanol | ADD hydroxylamine hydrochloride |
| 3 | REFLUX for 3600 s | STIR for 86400 s at 25 °C |
| 4 | SETTEMPERATURE 25 °C | CONCENTRATE |
| 5 | ADD isopropanol | ADD isopropanol |
| 6 | FILTER keep precipitate | REFLUX for 600 s |
| 7 | DRYSOLID under vacuum | PHASESEPARATION |
| 8 | YIELD 1-Azabicyclo[2.2.2]oct-2-ene-3-carboxaldehyde oxime,hydrochloride | YIELD 1-Azabicyclo[2.2.2]oct-2-ene-3-carboxaldehyde oxime,hydrochloride |

---

```
Reaction no 43
```

Generated by the Chemistry Development Kit (http://github.com/cdk)

|  | A | B |
| --- | --- | --- |
| 0 | ADD N-(3,3-diphenyl-3-methoxypropyl)-trifluoroacetamide | ADD N-(3,3-diphenyl-3-methoxypropyl)-trifluoroacetamide |
| 1 | ADD allyl bromide | ADD KOH |
| 2 | ADD acetone | ADD allyl bromide |
| 3 | SETTEMPERATURE 60 °C | ADD acetone |
| 4 | ADD KOH | REFLUX for 86400 s |
| 5 | STIR for 600 s | FILTER keep filtrate |
| 6 | CONCENTRATE | CONCENTRATE |
| 7 | STIR for 3600 s | YIELD 1,1-diphenyl-1-methoxy-3-allylaminopropane |
| 8 | YIELD 1,1-diphenyl-1-methoxy-3-allylaminopropane |  |

---

```
Reaction no 44
```

Generated by the Chemistry Development Kit (http://github.com/cdk)

|  | A | B |
| --- | --- | --- |
| 0 | ADD tert-butyl N-{2-[(tert-butoxycarbonyl)amino]ethyl}-D-valinate | ADD tert-butyl N-{2-[(tert-butoxycarbonyl)amino]ethyl}-D-valinate |
| 1 | ADD dichloromethane | ADD dichloromethane |
| 2 | ADD TFA | ADD TFA |
| 3 | STIR for 3600 s at 25 °C | WAIT for 86400 s |
| 4 | CONCENTRATE | CONCENTRATE |
| 5 | YIELD N-(2-aminoethyl)-D-valine bis(trifluoroacetate) | YIELD N-(2-aminoethyl)-D-valine bis(trifluoroacetate) |

---

```
Reaction no 45
```

Generated by the Chemistry Development Kit (http://github.com/cdk)

|  | A | B |
| --- | --- | --- |
| 0 | ADD 2-carboxypentylamino-4-chlorobenzoic Acid | ADD 2-carboxypentylamino-4-chlorobenzoic Acid |
| 1 | ADD water | ADD water |
| 2 | ADD NaOH dropwise | ADD NaOH |
| 3 | MAKESOLUTION with sodium cyanate and water | ADD sodium cyanate |
| 4 | ADD SLN dropwise at 25 °C over 3600 s | STIR for 86400 s at 100 °C |
| 5 | PH with HCl to pH acidic | SETTEMPERATURE 25 °C |
| 6 | STIR for 3600 s at 25 °C | PH with HCl to pH acidic |
| 7 | ADD NaOH | FILTER keep precipitate |
| 8 | STIR for 600 s at 60 °C | WASH with water |
| 9 | ADD 1-carboxymethyl-7-chloro-2,4 ‌(1H, 3H)-quinazolinedione | DRYSOLID under vacuum |
| 10 |  | YIELD 1-carboxymethyl-7-chloro-2,4 ‌(1H, 3H)-quinazolinedione |

---

```
Reaction no 46
```

Generated by the Chemistry Development Kit (http://github.com/cdk)

|  | A | B |
| --- | --- | --- |
| 0 | ADD Ethyl 1-(3-chlorophenyl)-5-(3-hydroxyphenyl)-1H-pyrazole-3-carboxylate | ADD Ethyl 1-(3-chlorophenyl)-5-(3-hydroxyphenyl)-1H-pyrazole-3-carboxylate |
| 1 | ADD 2-bromoethanol | ADD acetone |
| 2 | ADD K2CO3 | ADD K2CO3 |
| 3 | ADD acetone | ADD 2-bromoethanol |
| 4 | REFLUX for 86400 s | REFLUX for 86400 s |
| 5 | FILTER keep filtrate | PURIFY |
| 6 | CONCENTRATE | YIELD Ethyl 1-(3-chlorophenyl)-5-[3-(2-hydroxyethoxy)phenyl]-1H-pyrazole-3-carboxylate |
| 7 | PURIFY |  |
| 8 | YIELD Ethyl 1-(3-chlorophenyl)-5-[3-(2-hydroxyethoxy)phenyl]-1H-pyrazole-3-carboxylate |  |

---

```
Reaction no 47
```

Generated by the Chemistry Development Kit (http://github.com/cdk)

|  | A | B |
| --- | --- | --- |
| 0 | ADD tert-butyl ‌(3-{(cis-3-{3-[7-fluoro-3-(propan-2-yl)-1H-indazol-1-yl]-1,2,4-oxadiazol-5-yl}cyclobutyl)[(2-nitrophenyl)sulfonyl]amino}propyl)carbamate | ADD tert-butyl ‌(3-{(cis-3-{3-[7-fluoro-3-(propan-2-yl)-1H-indazol-1-yl]-1,2,4-oxadiazol-5-yl}cyclobutyl)[(2-nitrophenyl)sulfonyl]amino}propyl)carbamate |
| 1 | ADD hydrogen chloride-ethyl acetate | ADD hydrogen chloride-ethyl acetate |
| 2 | STIR for 3600 s at 25 °C | STIR for 3600 s at 25 °C |
| 3 | CONCENTRATE | CONCENTRATE |
| 4 | YIELD N-(3-aminopropyl)-N-(cis-3-{3-[7-fluoro-3-(propan-2-yl)-1H-indazol-1-yl]-1,2,4-oxadiazol-5-yl}cyclobutyl)-2-nitrobenzenesulfonamide | ADD pyridine |
| 5 |  | ADD acetic anhydride |
| 6 |  | STIR for 86400 s at 25 °C |
| 7 |  | CONCENTRATE |
| 8 |  | PURIFY |
| 9 |  | YIELD N-(3-aminopropyl)-N-(cis-3-{3-[7-fluoro-3-(propan-2-yl)-1H-indazol-1-yl]-1,2,4-oxadiazol-5-yl}cyclobutyl)-2-nitrobenzenesulfonamide |

---

```
Reaction no 48
```

Generated by the Chemistry Development Kit (http://github.com/cdk)

|  | A | B |
| --- | --- | --- |
| 0 | ADD N-(1-(3-chloro-6-(3-fluorophenyl)-5-oxo-5H-thiazolo[3,2-a]pyridin-7-yl)ethyl)-2-methylpropane-2-sulfinamide | ADD N-(1-(3-chloro-6-(3-fluorophenyl)-5-oxo-5H-thiazolo[3,2-a]pyridin-7-yl)ethyl)-2-methylpropane-2-sulfinamide |
| 1 | ADD ethanol | ADD ethanol |
| 2 | ADD HCl | ADD HCl dropwise at 25 °C |
| 3 | STIR for 86400 s at 25 °C | STIR for 3600 s |
| 4 | CONCENTRATE | QUENCH with ammonium hydroxide |
| 5 | YIELD 7-(1-aminoethyl)-3-chloro-6-(3-fluorophenyl)-5H-thiazolo[3,2-a]pyridin-5-one | EXTRACT with dichloromethane |
| 6 |  | COLLECTLAYER organic |
| 7 |  | WASH with brine |
| 8 |  | DRYSOLUTION over Na2SO4 |
| 9 |  | CONCENTRATE |
| 10 |  | YIELD 7-(1-aminoethyl)-3-chloro-6-(3-fluorophenyl)-5H-thiazolo[3,2-a]pyridin-5-one |

---

```
Reaction no 49
```

Generated by the Chemistry Development Kit (http://github.com/cdk)

|  | A | B |
| --- | --- | --- |
| 0 | ADD O=C([O-])[O-].[Ag+2] | ADD 4-chloro-N-ethyl-2-iodo-N-phenyl-benzamide |
| 1 | ADD triphenylphosphine | ADD acetonitrile |
| 2 | ADD 4-chloro-N-ethyl-2-iodo-N-phenyl-benzamide | ADD Pd(OAc)2 |
| 3 | ADD acetonitrile | ADD triphenylphosphine |
| 4 | ADD Pd(OAc)2 | ADD O=C([O-])[O-].[Ag+2] |
| 5 | REFLUX for 3600 s | STIR for 86400 s at 100 °C |
| 6 | SETTEMPERATURE 25 °C | FILTER keep filtrate |
| 7 | FILTER keep filtrate | CONCENTRATE |
| 8 | WASH with ethyl acetate | PURIFY |
| 9 | WASH with ether | YIELD 9-chloro-5-ethyl-5H-phenanthridin-6-one |
| 10 | WASH with sodium chloride |  |
| 11 | DRYSOLUTION over sodium sulfate |  |
| 12 | CONCENTRATE |  |
| 13 | PURIFY |  |
| 14 | YIELD 9-chloro-5-ethyl-5H-phenanthridin-6-one |  |

---

```
Reaction no 50
```

Generated by the Chemistry Development Kit (http://github.com/cdk)

|  | A | B |
| --- | --- | --- |
| 0 | ADD 5-(1-methyl-4-(pyridin-4-yl)-1H-pyrazol-3-yl)-2-((trimethylsilyl)ethynyl)pyridine | ADD 5-(1-methyl-4-(pyridin-4-yl)-1H-pyrazol-3-yl)-2-((trimethylsilyl)ethynyl)pyridine |
| 1 | ADD TBAF THF | ADD TBAF THF |
| 2 | STIR for 3600 s at 25 °C | STIR for 3600 s at 25 °C |
| 3 | CONCENTRATE | PARTITION with water and ethyl acetate |
| 4 | PURIFY | PHASESEPARATION |
| 5 | YIELD 2-ethynyl-5-(4-(pyridin-4-yl)-1H-pyrazol-3-yl)pyridine | COLLECTLAYER organic |
| 6 |  | COLLECTLAYER aqueous |
| 7 |  | EXTRACT with ethyl acetate |
| 8 |  | COLLECTLAYER organic |
| 9 |  | WASH with brine |
| 10 |  | DRYSOLUTION over Na2SO4 |
| 11 |  | FILTER keep filtrate |
| 12 |  | CONCENTRATE |
| 13 |  | PURIFY |
| 14 |  | YIELD 2-ethynyl-5-(4-(pyridin-4-yl)-1H-pyrazol-3-yl)pyridine |

---

```
Reaction no 51
```

Generated by the Chemistry Development Kit (http://github.com/cdk)

|  | A | B |
| --- | --- | --- |
| 0 | ADD tert-butyl 4-((4-(1-(dimethylcarbamoyl)-6-fluoro-1H-indol-3-yl)phenyl)-sulfonyl) piperazine-1-carboxylate | ADD tert-butyl 4-((4-(1-(dimethylcarbamoyl)-6-fluoro-1H-indol-3-yl)phenyl)-sulfonyl) piperazine-1-carboxylate |
| 1 | ADD ether | ADD ether |
| 2 | ADD HCl Et2O | ADD HCl Et2O |
| 3 | STIR for 3600 s at 25 °C | STIR for 28800 s at 25 °C |
| 4 | CONCENTRATE | CONCENTRATE |
| 5 | PURIFY | RECRYSTALLIZE from ethyl acetate |
| 6 | YIELD 6-fluoro-N,N-dimethyl-3-(4-(piperazin-1-ylsulfonyl)phenyl)-1H-indole-1-carboxamide | YIELD 6-fluoro-N,N-dimethyl-3-(4-(piperazin-1-ylsulfonyl)phenyl)-1H-indole-1-carboxamide |

---

```
Reaction no 52
```

Generated by the Chemistry Development Kit (http://github.com/cdk)

|  | A | B |
| --- | --- | --- |
| 0 | ADD N-{4-[4-(2-Trimethylsilanyl-ethoxymethyl)-4,7-dihydro-1-thia-4,5-diaza-cyclopenta[a]pentalen-6-yl]-phenyl}-acetamide | ADD N-{4-[4-(2-Trimethylsilanyl-ethoxymethyl)-4,7-dihydro-1-thia-4,5-diaza-cyclopenta[a]pentalen-6-yl]-phenyl}-acetamide |
| 1 | ADD methanol | ADD methanol |
| 2 | ADD HCl | ADD HCl |
| 3 | STIR for 28800 s at 100 °C | STIR for 28800 s at 100 °C |
| 4 | SETTEMPERATURE 25 °C | SETTEMPERATURE 25 °C |
| 5 | FILTER keep precipitate | FILTER keep precipitate |
| 6 | WASH with methanol | WASH with methanol |
| 7 | CONCENTRATE | CONCENTRATE |
| 8 | YIELD 4-(4,7-Dihydro-1-thia-4,5-diaza-cyclopenta[a]pentalen-6-yl)-phenylamine hydrochloride | YIELD 4-(4,7-Dihydro-1-thia-4,5-diaza-cyclopenta[a]pentalen-6-yl)-phenylamine hydrochloride |

---

```
Reaction no 53
```

Generated by the Chemistry Development Kit (http://github.com/cdk)

|  | A | B |
| --- | --- | --- |
| 0 | ADD thionyl chloride at 25 °C | ADD 3-carboxy-1-(isoquinol-1-yl)-4-methyl-1H-pyrrole |
| 1 | ADD 3-carboxy-1-(isoquinol-1-yl)-4-methyl-1H-pyrrole at 25 °C | ADD thionyl chloride |
| 2 | ADD chloroform at 25 °C | ADD chloroform |
| 3 | REFLUX for 3600 s | REFLUX for 3600 s |
| 4 | CONCENTRATE | CONCENTRATE |
| 5 | ADD chloroform | YIELD 3-chlorocarbonyl-1-(isoquinol-1-yl)-4-methyl-1H-pyrrole |
| 6 | YIELD 3-chlorocarbonyl-1-(isoquinol-1-yl)-4-methyl-1H-pyrrole |  |

---

```
Reaction no 54
```

Generated by the Chemistry Development Kit (http://github.com/cdk)

|  | A | B |
| --- | --- | --- |
| 0 | ADD methyl 2-(2-(1-methyl-1H-1,2,3-triazol-4-yl)cyclopropanecarbonyl)-3-oxobutanoate | ADD methyl 2-(2-(1-methyl-1H-1,2,3-triazol-4-yl)cyclopropanecarbonyl)-3-oxobutanoate |
| 1 | ADD acetic acid | ADD acetic acid |
| 2 | ADD 3-bromophenylhydrazine hydrochloride | MAKESOLUTION with 3-bromophenylhydrazine hydrochloride and water |
| 3 | STIR for 86400 s at 25 °C | ADD SLN at 25 °C |
| 4 | ADD water | STIR for 86400 s at 25 °C |
| 5 | EXTRACT with ethyl acetate | EXTRACT with ethyl acetate |
| 6 | COLLECTLAYER organic | COLLECTLAYER organic |
| 7 | WASH with brine | CONCENTRATE |
| 8 | DRYSOLUTION over sodium sulfate | PURIFY |
| 9 | CONCENTRATE | YIELD methyl 1-(3-bromophenyl)-3-methyl-5-(2-(1-methyl-1H-1,2,3-triazol-4-yl)cyclopropyl)-1H-pyrazole-4-carboxylate |
| 10 | PURIFY |  |
| 11 | YIELD methyl 1-(3-bromophenyl)-3-methyl-5-(2-(1-methyl-1H-1,2,3-triazol-4-yl)cyclopropyl)-1H-pyrazole-4-carboxylate |  |

---

```
Reaction no 55
```

Generated by the Chemistry Development Kit (http://github.com/cdk)

|  | A | B |
| --- | --- | --- |
| 0 | ADD (R)-3-amino-N-(3-(cyclopropylmethyl)-1-isopropyl-2,4-dioxo-1,2,3,4-tetrahydroquinazolin-6-yl)piperidine-1-carboxamide | ADD (R)-3-amino-N-(3-(cyclopropylmethyl)-1-isopropyl-2,4-dioxo-1,2,3,4-tetrahydroquinazolin-6-yl)piperidine-1-carboxamide |
| 1 | ADD 4-{[(tert-butoxycarbonyl)amino]methyl}benzoic acid | ADD 4-{[(tert-butoxycarbonyl)amino]methyl}benzoic acid |
| 2 | ADD DMF | ADD DIPEA |
| 3 | ADD DIPEA at 0 °C | ADD DMF |
| 4 | ADD T3P at 0 °C | ADD T3P at 25 °C |
| 5 | STIR for 86400 s at 25 °C | STIR for 86400 s at 25 °C |
| 6 | ADD ammonium chloride | ADD ammonium chloride |
| 7 | EXTRACT with ethyl acetate | EXTRACT with ethyl acetate |
| 8 | COLLECTLAYER organic | COLLECTLAYER organic |
| 9 | WASH with water | WASH with brine |
| 10 | WASH with NaHCO3 | DRYSOLUTION over magnesium sulfate |
| 11 | WASH with brine | CONCENTRATE |
| 12 | DRYSOLUTION over magnesium sulfate | PURIFY |
| 13 | CONCENTRATE | YIELD tert-Butyl ‌(4-(((3R)-1-((3-(cyclopropylmethyl)-1-isopropyl-2,4-dioxo-1,2,3,4-tetrahydroquinazolin-6-yl)carbamoyl)piperidin-3-yl)carbamoyl)benzyl)carbamate |
| 14 | PURIFY |  |
| 15 | YIELD tert-Butyl ‌(4-(((3R)-1-((3-(cyclopropylmethyl)-1-isopropyl-2,4-dioxo-1,2,3,4-tetrahydroquinazolin-6-yl)carbamoyl)piperidin-3-yl)carbamoyl)benzyl)carbamate |  |

---

```
Reaction no 56
```

Generated by the Chemistry Development Kit (http://github.com/cdk)

|  | A | B |
| --- | --- | --- |
| 0 | ADD (S)-2-fluoro-4-(6-methyl-4-(3-methylmorpholino)-6,7-dihydro-5H-pyrrolo[3,4-d]pyrimidin-2-yl)aniline | ADD (S)-2-fluoro-4-(6-methyl-4-(3-methylmorpholino)-6,7-dihydro-5H-pyrrolo[3,4-d]pyrimidin-2-yl)aniline |
| 1 | ADD THF | ADD THF |
| 2 | ADD ethyl isocyanate dropwise | ADD ethyl isocyanate |
| 3 | STIR for 86400 s at 60 °C | STIR for 3600 s at 25 °C |
| 4 | SETTEMPERATURE 25 °C | CONCENTRATE |
| 5 | PURIFY | PURIFY |
| 6 | YIELD (S)-1-ethyl-3-(2-fluoro-4-(6-methyl-4-(3-methylmorpholino)-6,7-dihydro-5H-pyrrolo[3,4-d]pyrimidin-2-yl)phenyl)urea | YIELD (S)-1-ethyl-3-(2-fluoro-4-(6-methyl-4-(3-methylmorpholino)-6,7-dihydro-5H-pyrrolo[3,4-d]pyrimidin-2-yl)phenyl)urea |

---

```
Reaction no 57
```

Generated by the Chemistry Development Kit (http://github.com/cdk)

|  | A | B |
| --- | --- | --- |
| 0 | ADD 3,5-dimethyl-4-phenyl-1H-pyrazole | ADD 3,5-dimethyl-4-phenyl-1H-pyrazole |
| 1 | ADD ethyl 6-chloronicotinate | ADD ethyl 6-chloronicotinate |
| 2 | ADD K2CO3 | ADD K2CO3 |
| 3 | ADD DMSO | ADD toluene |
| 4 | STIR for 86400 s at 100 °C | ADD DMSO |
| 5 | SETTEMPERATURE 25 °C | STIR for 86400 s at 100 °C |
| 6 | ADD ethyl acetate | SETTEMPERATURE 25 °C |
| 7 | ADD toluene | ADD ethyl acetate |
| 8 | WASH with water | WASH with water |
| 9 | DRYSOLUTION over MgSO4 | WASH with brine |
| 10 | CONCENTRATE | DRYSOLUTION over Na2SO4 |
| 11 | PURIFY | FILTER keep filtrate |
| 12 | YIELD ethyl 6-(3,5-dimethyl-4-phenyl-1H-pyrazol-1-yl)nicotinate | CONCENTRATE |
| 13 |  | PURIFY |
| 14 |  | YIELD ethyl 6-(3,5-dimethyl-4-phenyl-1H-pyrazol-1-yl)nicotinate |

---

```
Reaction no 58
```

Generated by the Chemistry Development Kit (http://github.com/cdk)

|  | A | B |
| --- | --- | --- |
| 0 | ADD 6-(3-chlorophenyl)-7-(((trans)-4-methylcyclohexyl)methyl)-7H-purine-2-carbonitrile | ADD 6-(3-chlorophenyl)-7-(((trans)-4-methylcyclohexyl)methyl)-7H-purine-2-carbonitrile |
| 1 | ADD THF | ADD THF |
| 2 | ADD 2,2,6,6-tetramethylpiperidinylmagnesium chloride at -70 °C | ADD 2,2,6,6-tetramethylpiperidinylmagnesium chloride |
| 3 | ADD Lithiumchlorid at -70 °C | ADD Lithiumchlorid |
| 4 | STIR for 3600 s at 60 °C | ADD N-methoxy-N,5-dimethyloxazole-4-carboxamide |
| 5 | MAKESOLUTION with N-methoxy-N,5-dimethyloxazole-4-carboxamide and THF | PURIFY |
| 6 | ADD SLN at -70 °C | YIELD 6-(3-chlorophenyl)-7-(((trans)-4-methylcyclohexyl)methyl)-8-(5-methyloxazole-4-carbonyl)-7H-purine-2-carbonitrile |
| 7 | SETTEMPERATURE 0 °C |  |
| 8 | STIR for 3600 s |  |
| 9 | QUENCH with ammonium chloride |  |
| 10 | EXTRACT with ethyl acetate |  |
| 11 | COLLECTLAYER organic |  |
| 12 | WASH with brine |  |
| 13 | DRYSOLUTION over Na2SO4 |  |
| 14 | FILTER keep filtrate |  |
| 15 | CONCENTRATE |  |
| 16 | PURIFY |  |
| 17 | YIELD 6-(3-chlorophenyl)-7-(((trans)-4-methylcyclohexyl)methyl)-8-(5-methyloxazole-4-carbonyl)-7H-purine-2-carbonitrile |  |

---

```
Reaction no 59
```

Generated by the Chemistry Development Kit (http://github.com/cdk)

|  | A | B |
| --- | --- | --- |
| 0 | ADD (3S,5S)-Thioacetic acid S-[5-(piperidine-1-carbonyl)-pyrrolidin-3-yl] ester | ADD (3S,5S)-Thioacetic acid S-[5-(piperidine-1-carbonyl)-pyrrolidin-3-yl] ester |
| 1 | ADD 2-naphthalenesulfonyl chloride | ADD dichloromethane |
| 2 | ADD triethylamine | ADD 2-naphthalenesulfonyl chloride |
| 3 | ADD DMAP | ADD DMAP |
| 4 | ADD dichloromethane | STIR for 3600 s at 25 °C |
| 5 | STIR for 86400 s at 25 °C | ADD KHSO4 |
| 6 | ADD KHSO4 | PHASESEPARATION |
| 7 | EXTRACT with ethyl acetate | COLLECTLAYER organic |
| 8 | COLLECTLAYER organic | WASH with KHSO4 |
| 9 | WASH with water | WASH with water |
| 10 | WASH with brine | WASH with brine |
| 11 | DRYSOLUTION over Na2SO4 | DRYSOLUTION over Na2SO4 |
| 12 | CONCENTRATE | CONCENTRATE |
| 13 | PURIFY | YIELD (3S,5S)-Thioacetic acid S-[1-(naphthalene-2-sulfonyl)-5-(piperidine-1-carbonyl)-pyrrolidin-3-yl] ester |
| 14 | YIELD (3S,5S)-Thioacetic acid S-[1-(naphthalene-2-sulfonyl)-5-(piperidine-1-carbonyl)-pyrrolidin-3-yl] ester |  |

---

```
Reaction no 60
```

Generated by the Chemistry Development Kit (http://github.com/cdk)

|  | A | B |
| --- | --- | --- |
| 0 | ADD tert-butyl 4-bromo-6-formylisoindoline-2-carboxylate | ADD tert-butyl 4-bromo-6-formylisoindoline-2-carboxylate |
| 1 | ADD methanol | ADD methanol |
| 2 | ADD hydroxylamine hydrochloride | ADD hydroxylamine hydrochloride at 25 °C |
| 3 | ADD zinc | STIR for 3600 s at 25 °C |
| 4 | STIR for 86400 s at 25 °C | CONCENTRATE |
| 5 | ADD NaHCO3 | ADD acetic acid |
| 6 | EXTRACT with ethyl acetate | ADD zinc at 25 °C |
| 7 | COLLECTLAYER organic | STIR for 28800 s at 25 °C |
| 8 | DRYSOLUTION over Na2SO4 | ADD NaHCO3 |
| 9 | FILTER keep filtrate | EXTRACT with ethyl acetate |
| 10 | CONCENTRATE | COLLECTLAYER organic |
| 11 | YIELD tert-butyl 6-(aminomethyl)-4-bromoisoindoline-2-carboxylate | DRYSOLUTION over Na2SO4 |
| 12 |  | FILTER keep filtrate |
| 13 |  | CONCENTRATE |
| 14 |  | YIELD tert-butyl 6-(aminomethyl)-4-bromoisoindoline-2-carboxylate |

---

```
Reaction no 61
```

Generated by the Chemistry Development Kit (http://github.com/cdk)

|  | A | B |
| --- | --- | --- |
| 0 | ADD Trans-4-(6-Bromo-3-methyl-2-oxo-2,3-dihydro-benzoimidazol-1-ylmethyl)-cyclohexanecarboxylic acid methyl ester | ADD Trans-4-(6-Bromo-3-methyl-2-oxo-2,3-dihydro-benzoimidazol-1-ylmethyl)-cyclohexanecarboxylic acid methyl ester |
| 1 | ADD bis(pinacolato)diboron | ADD bis(pinacolato)diboron |
| 2 | ADD Pd(dppf)Cl2 | ADD Pd(dppf)Cl2 |
| 3 | ADD AcOK | ADD AcOK |
| 4 | ADD dioxane | ADD dioxane |
| 5 | STIR for 28800 s at 100 °C | STIR for 86400 s at 100 °C |
| 6 | ADD ethyl acetate | SETTEMPERATURE 25 °C |
| 7 | ADD water | ADD ethyl acetate |
| 8 | PHASESEPARATION | ADD water |
| 9 | COLLECTLAYER organic | COLLECTLAYER aqueous |
| 10 | DRYSOLUTION over Na2SO4 | EXTRACT with ethyl acetate |
| 11 | CONCENTRATE | COLLECTLAYER organic |
| 12 | PURIFY | DRYSOLUTION over Na2SO4 |
| 13 | YIELD Trans-4-[3-Methyl-2-oxo-6-(4,4,5,5-tetramethyl-[1,3,2] dioxaborolan-2-yl)-2,3-dihydro-benzoimidazol-1-ylmethyl]-cyclohexanecarboxylic acid methyl ester | FILTER keep filtrate |
| 14 |  | CONCENTRATE |
| 15 |  | PURIFY |
| 16 |  | YIELD Trans-4-[3-Methyl-2-oxo-6-(4,4,5,5-tetramethyl-[1,3,2] dioxaborolan-2-yl)-2,3-dihydro-benzoimidazol-1-ylmethyl]-cyclohexanecarboxylic acid methyl ester |

---

```
Reaction no 62
```

Generated by the Chemistry Development Kit (http://github.com/cdk)

|  | A | B |
| --- | --- | --- |
| 0 | ADD 1-t-butoxy-2-deoxy-L-ribose | ADD 1-t-butoxy-2-deoxy-L-ribose |
| 1 | ADD acetic acid | ADD acetic acid |
| 2 | STIR for 3600 s at 60 °C | STIR for 28800 s at 60 °C |
| 3 | CONCENTRATE | CONCENTRATE |
| 4 | PURIFY | CONCENTRATE |
| 5 | YIELD 2-deoxy-L-ribose | PURIFY |
| 6 |  | YIELD 2-deoxy-L-ribose |

---

```
Reaction no 63
```

Generated by the Chemistry Development Kit (http://github.com/cdk)

|  | A | B |
| --- | --- | --- |
| 0 | ADD 4-(5-((5-chloro-2-oxoindolin-3-ylidene)methyl)furan-2-yl)-3-fluorobenzoic acid | ADD 4-(5-((5-chloro-2-oxoindolin-3-ylidene)methyl)furan-2-yl)-3-fluorobenzoic acid |
| 1 | ADD DMF | ADD DMF |
| 2 | ADD HBTU | ADD HBTU |
| 3 | ADD DIPEA | ADD DIPEA |
| 4 | ADD 1-methyl-1,4-diazepane | STIR at 25 °C |
| 5 | STIR for 86400 s at 25 °C | ADD 1-methyl-1,4-diazepane |
| 6 | CONCENTRATE | SETTEMPERATURE 25 °C |
| 7 | ADD DMSO | FILTER keep precipitate |
| 8 | PURIFY | PURIFY |
| 9 | FILTER keep precipitate | YIELD 5-chloro-3-((5-(2-fluoro-4-(4-methyl-1,4-diazepane-1-carbonyl)phenyl)furan-2-yl)methylene)indolin-2-one |
| 10 | CONCENTRATE |  |
| 11 | YIELD 5-chloro-3-((5-(2-fluoro-4-(4-methyl-1,4-diazepane-1-carbonyl)phenyl)furan-2-yl)methylene)indolin-2-one |  |

---

```
Reaction no 64
```

Generated by the Chemistry Development Kit (http://github.com/cdk)

|  | A | B |
| --- | --- | --- |
| 0 | ADD benzyl ‌(2S)-2-(cyanomethyl)-4-[7-(8-methyl-1-naphthyl)-2-methylsulfinyl-6,8-dihydro-5H-pyrido[3,4-d]pyrimidin-4-yl]piperazine-1-carboxylate | ADD [(3R)-1-methylpyrrolidin-3-yl]methanol |
| 1 | ADD [(3R)-1-methylpyrrolidin-3-yl]methanol | ADD toluene |
| 2 | ADD toluene | ADD sodium tert-butoxide |
| 3 | ADD sodium tert-butoxide | STIR for 3600 s at 0 °C |
| 4 | STIR for 600 s at 0 °C | ADD benzyl ‌(2S)-2-(cyanomethyl)-4-[7-(8-methyl-1-naphthyl)-2-methylsulfinyl-6,8-dihydro-5H-pyrido[3,4-d]pyrimidin-4-yl]piperazine-1-carboxylate |
| 5 | ADD water | STIR for 3600 s at 0 °C |
| 6 | EXTRACT with ethyl acetate | ADD water |
| 7 | COLLECTLAYER organic | EXTRACT with ethyl acetate |
| 8 | DRYSOLUTION over Na2SO4 | COLLECTLAYER organic |
| 9 | CONCENTRATE | DRYSOLUTION over Na2SO4 |
| 10 | PURIFY | FILTER keep filtrate |
| 11 | YIELD benzyl ‌(2S)-2-(cyanomethyl)-4-[7-(8-methyl-1-naphthyl)-2-[[(3R)-1-methylpyrrolidin-3-yl]methoxy]-6,8-dihydro-5H-pyrido[3,4-d]pyrimidin-4-yl]piperazine-1-carboxylate | CONCENTRATE |
| 12 |  | PURIFY |
| 13 |  | YIELD benzyl ‌(2S)-2-(cyanomethyl)-4-[7-(8-methyl-1-naphthyl)-2-[[(3R)-1-methylpyrrolidin-3-yl]methoxy]-6,8-dihydro-5H-pyrido[3,4-d]pyrimidin-4-yl]piperazine-1-carboxylate |

---

```
Reaction no 65
```

Generated by the Chemistry Development Kit (http://github.com/cdk)

|  | A | B |
| --- | --- | --- |
| 0 | ADD {6-[(4-{[(2-phenylethyl)(4-phenyl-1,3-thiazol-2-yl)amino]methyl}benzyl)oxy]-2,3-dihydro-1-benzofuran-3-yl}acetic acid | ADD {6-[(4-{[(2-phenylethyl)(4-phenyl-1,3-thiazol-2-yl)amino]methyl}benzyl)oxy]-2,3-dihydro-1-benzofuran-3-yl}acetic acid |
| 1 | ADD methanol | ADD methanol |
| 2 | ADD NaOH | ADD NaOH |
| 3 | CONCENTRATE | STIR for 3600 s at 25 °C |
| 4 | ADD water | CONCENTRATE |
| 5 | ADD methanol | ADD water |
| 6 | MAKESOLUTION with Calciumchlorid and water | PH with Calciumchlorid to pH neutral |
| 7 | ADD SLN | FILTER keep precipitate |
| 8 | CONCENTRATE | DRYSOLID under vacuum |
| 9 | FILTER keep precipitate | YIELD calcium {6-[(4-{[(2-phenylethyl)(4-phenyl-1,3-thiazol-2-yl)amino]methyl}benzyl)oxy]-2,3-dihydro-1-benzofuran-3-yl}acetate |
| 10 | WASH with water |  |
| 11 | YIELD calcium {6-[(4-{[(2-phenylethyl)(4-phenyl-1,3-thiazol-2-yl)amino]methyl}benzyl)oxy]-2,3-dihydro-1-benzofuran-3-yl}acetate |  |

---

```
Reaction no 66
```

Generated by the Chemistry Development Kit (http://github.com/cdk)

|  | A | B |
| --- | --- | --- |
| 0 | ADD (((9H-fluoren-9-yl)methoxy)carbonyl)-L-threonine | ADD (((9H-fluoren-9-yl)methoxy)carbonyl)-L-threonine |
| 1 | ADD PyBroP | ADD Ala-Sar-NMeH |
| 2 | ADD DIPEA | ADD dichloromethane |
| 3 | ADD dichloromethane | ADD PyBroP at 0 °C |
| 4 | ADD Ala-Sar-NMeH | ADD DIPEA at 0 °C |
| 5 | STIR for 3600 s | SETTEMPERATURE 25 °C |
| 6 | FILTER keep precipitate | STIR for 86400 s |
| 7 | WASH with N-methylpyrrolidine | ADD water |
| 8 | WASH with methanol | EXTRACT with ethyl acetate |
| 9 | WASH with N-methylpyrrolidine | COLLECTLAYER organic |
| 10 | YIELD Ala-Sar-Thr-NHFmoc | WASH with brine |
| 11 |  | DRYSOLUTION over sodium sulfate |
| 12 |  | CONCENTRATE |
| 13 |  | PURIFY |
| 14 |  | YIELD N-methylpyrrolidine |
| 15 |  | YIELD Ala-Sar-Thr-NHFmoc |

---

```
Reaction no 67
```

Generated by the Chemistry Development Kit (http://github.com/cdk)

|  | A | B |
| --- | --- | --- |
| 0 | ADD t-butyl 4-phenylbut-2-enoate | ADD t-butyl 4-phenylbut-2-enoate |
| 1 | ADD dichloromethane | ADD dichloromethane |
| 2 | ADD TFA | ADD TFA |
| 3 | STIR for 3600 s at 25 °C | STIR for 86400 s at 25 °C |
| 4 | CONCENTRATE | CONCENTRATE |
| 5 | YIELD 4-phenylbut-2-enoic acid | YIELD 4-phenylbut-2-enoic acid |

---

```
Reaction no 68
```

Generated by the Chemistry Development Kit (http://github.com/cdk)

|  | A | B |
| --- | --- | --- |
| 0 | ADD 8-(3-bromo-4-fluorophenyl)-3,3-difluoro-8-(4-methoxy-3-methylphenyl)-3,4,7,8-tetrahydroimidazo[1,5-a]pyrimidine-6(2H)-thione | ADD 8-(3-bromo-4-fluorophenyl)-3,3-difluoro-8-(4-methoxy-3-methylphenyl)-3,4,7,8-tetrahydroimidazo[1,5-a]pyrimidine-6(2H)-thione |
| 1 | ADD methanol | ADD methanol |
| 2 | ADD ammonium hydroxide | ADD ammonium hydroxide |
| 3 | ADD tert-butyl hydroperoxide | ADD tert-butyl hydroperoxide |
| 4 | STIR for 86400 s at 25 °C | STIR for 86400 s at 25 °C |
| 5 | CONCENTRATE | CONCENTRATE |
| 6 | ADD dichloromethane | PURIFY |
| 7 | COLLECTLAYER organic | YIELD 8-(3-Bromo-4-fluorophenyl)-3,3-difluoro-8-(4-methoxy-3-methylphenyl)-2,3,4,8-tetrahydroimidazo[1,5-a]pyrimidin-6-amine |
| 8 | WASH with sodium chloride |  |
| 9 | DRYSOLUTION over sodium sulfate |  |
| 10 | FILTER keep filtrate |  |
| 11 | CONCENTRATE |  |
| 12 | ADD silica gel |  |
| 13 | PURIFY |  |
| 14 | YIELD 8-(3-Bromo-4-fluorophenyl)-3,3-difluoro-8-(4-methoxy-3-methylphenyl)-2,3,4,8-tetrahydroimidazo[1,5-a]pyrimidin-6-amine |  |

---

```
Reaction no 69
```

Generated by the Chemistry Development Kit (http://github.com/cdk)

|  | A | B |
| --- | --- | --- |
| 0 | ADD (1RS,2RS)-1-(6-Benzyloxy-1-hydroxy-1,2,3,4-tetrahydro-naphthalen-2-ylmethyl)-4-(4-methyl-benzyl)-piperidin-4-ol | ADD (1RS,2RS)-1-(6-Benzyloxy-1-hydroxy-1,2,3,4-tetrahydro-naphthalen-2-ylmethyl)-4-(4-methyl-benzyl)-piperidin-4-ol |
| 1 | ADD ethyl acetate | ADD ethyl acetate |
| 2 | ADD Pd/C | ADD Pd/C |
| 3 | STIR for 28800 s at 25 °C | STIR for 86400 s at 25 °C |
| 4 | CONCENTRATE | FILTER keep filtrate |
| 5 | YIELD 1-(1,6-Dihydroxy-1,2,3,4-tetrahydro-naphthalen-2-ylmethyl)-4-(4-methyl-benzyl)-piperidin-4-ol | CONCENTRATE |
| 6 |  | PURIFY |
| 7 |  | YIELD 1-(1,6-Dihydroxy-1,2,3,4-tetrahydro-naphthalen-2-ylmethyl)-4-(4-methyl-benzyl)-piperidin-4-ol |

---

```
Reaction no 70
```

Generated by the Chemistry Development Kit (http://github.com/cdk)

|  | A | B |
| --- | --- | --- |
| 0 | ADD 6-bromo-2-chloro-5-phenyl-thieno[2,3-d]pyrimidine | ADD 4-(2-pyrrolidin-1-ylethoxymethyl)piperidine |
| 1 | ADD acetonitrile | ADD 6-bromo-2-chloro-5-phenyl-thieno[2,3-d]pyrimidine |
| 2 | ADD 4-(2-pyrrolidin-1-ylethoxymethyl)piperidine | ADD K2CO3 |
| 3 | ADD K2CO3 | ADD acetonitrile |
| 4 | STIR for 86400 s at 100 °C | SETTEMPERATURE 100 °C |
| 5 | SETTEMPERATURE 25 °C | ADD dichloromethane |
| 6 | ADD dichloromethane | WASH with water |
| 7 | WASH with water | DRYSOLUTION over Na2SO4 |
| 8 | WASH with brine | CONCENTRATE |
| 9 | DRYSOLUTION over Na2SO4 | PURIFY |
| 10 | CONCENTRATE | YIELD 6-Bromo-5-phenyl-2-[4-(2-pyrrolidin-1-ylethoxymethyl)-1-piperidyl]thieno[2,3-d]pyrimidine |
| 11 | PURIFY |  |
| 12 | YIELD 6-Bromo-5-phenyl-2-[4-(2-pyrrolidin-1-ylethoxymethyl)-1-piperidyl]thieno[2,3-d]pyrimidine |  |

---

```
Reaction no 71
```

Generated by the Chemistry Development Kit (http://github.com/cdk)

|  | A | B |
| --- | --- | --- |
| 0 | ADD pyridine | ADD undecaprenol |
| 1 | ADD undecaprenol | ADD pyridine |
| 2 | ADD benzoyl chloride | MAKESOLUTION with benzoyl chloride and pyridine |
| 3 | STIR for 3600 s at 25 °C | ADD SLN dropwise over 3600 s |
| 4 | ADD hexane | STIR for 3600 s at 25 °C |
| 5 | WASH with sodium bicarbonate | ADD hexane |
| 6 | WASH with water | WASH with water |
| 7 | CONCENTRATE | CONCENTRATE |
| 8 | PURIFY | PURIFY |
| 9 | YIELD 3,7,11,15,19,23,27,31,35,39,43-Undecamethyl-2,6,10,14,18,22,26,30,35,38,42-tetratetracontaundecaenyl benzoate | YIELD 3,7,11,15,19,23,27,31,35,39,43-Undecamethyl-2,6,10,14,18,22,26,30,35,38,42-tetratetracontaundecaenyl benzoate |

---

```
Reaction no 72
```

Generated by the Chemistry Development Kit (http://github.com/cdk)

|  | A | B |
| --- | --- | --- |
| 0 | ADD 5-hydroxy-6-methoxy-3-pivaloyloxymethyl-3,4-dihydroquinazolin-4-one | ADD 5-hydroxy-6-methoxy-3-pivaloyloxymethyl-3,4-dihydroquinazolin-4-one |
| 1 | ADD 1-methylpiperidin-4-ol | ADD triphenylphosphine |
| 2 | ADD triphenylphosphine | ADD 1-methylpiperidin-4-ol |
| 3 | ADD dichloromethane | ADD dichloromethane |
| 4 | MAKESOLUTION with tert-butyl azodicarboxylate and dichloromethane | MAKESOLUTION with tert-butyl azodicarboxylate and dichloromethane |
| 5 | ADD SLN dropwise at 0 °C | ADD SLN |
| 6 | STIR for 3600 s at 0 °C | SETTEMPERATURE 0 °C |
| 7 | CONCENTRATE | STIR for 3600 s at 0 °C |
| 8 | ADD methanol | CONCENTRATE |
| 9 | ADD HCl / dioxane | PURIFY |
| 10 | STIR for 3600 s at 25 °C | ADD methanol / ammonia |
| 11 | CONCENTRATE | STIR for 86400 s |
| 12 | PURIFY | CONCENTRATE |
| 13 | YIELD 6-methoxy-5-(N-methylpiperidin-4-yloxy)-3,4-dihydroquinazolin-4-one | TRITURATE with ether |
| 14 |  | WASH with ether |
| 15 |  | DRYSOLID under vacuum |
| 16 |  | YIELD 6-methoxy-5-(N-methylpiperidin-4-yloxy)-3,4-dihydroquinazolin-4-one |

---

```
Reaction no 73
```

Generated by the Chemistry Development Kit (http://github.com/cdk)

|  | A | B |
| --- | --- | --- |
| 0 | ADD [(3S)-5-methyl-4-oxo-2,3-dihydro-1,5-benzoxazepin-3-yl]ammonium chloride | ADD [(3S)-5-methyl-4-oxo-2,3-dihydro-1,5-benzoxazepin-3-yl]ammonium chloride |
| 1 | ADD DMF | ADD DMF |
| 2 | ADD sodium 4-phenylpyrazolo[1,5-a]pyridine-2-carboxylate | ADD sodium 4-phenylpyrazolo[1,5-a]pyridine-2-carboxylate |
| 3 | ADD DIPEA | ADD PyAOP |
| 4 | ADD PyAOP | ADD DIPEA |
| 5 | STIR for 86400 s at 25 °C | STIR for 86400 s at 25 °C |
| 6 | PURIFY | PURIFY |
| 7 | YIELD 4-phenyl-N-[(3S)-5-methyl-4-oxo-2,3-dihydro-1,5-benzoxazepin-3-yl]pyrazolo[1,5-a]pyridine-2-carboxamide | YIELD 4-phenyl-N-[(3S)-5-methyl-4-oxo-2,3-dihydro-1,5-benzoxazepin-3-yl]pyrazolo[1,5-a]pyridine-2-carboxamide |

---

```
Reaction no 74
```

Generated by the Chemistry Development Kit (http://github.com/cdk)

|  | A | B |
| --- | --- | --- |
| 0 | ADD 2,3,5-trimethylphenol | ADD 2,3,5-trimethylphenol |
| 1 | ADD methallyl chloride | ADD methallyl chloride |
| 2 | ADD K2CO3 | ADD K2CO3 |
| 3 | ADD DMF | ADD DMF |
| 4 | STIR for 28800 s at 100 °C | STIR for 86400 s at 60 °C |
| 5 | WAIT for 28800 s at 100 °C | SETTEMPERATURE 25 °C |
| 6 | SETTEMPERATURE 0 °C | FILTER keep filtrate |
| 7 | ADD ice water | CONCENTRATE |
| 8 | EXTRACT with ethyl acetate | ADD ethyl acetate |
| 9 | WASH with water | WASH with water |
| 10 | WASH with brine | WASH with brine |
| 11 | DRYSOLUTION over magnesium sulfate | DRYSOLUTION over magnesium sulfate |
| 12 | CONCENTRATE | CONCENTRATE |
| 13 | PURIFY | YIELD 2, 3, 5-trimethylphenyl 2-methylpropenyl ether |
| 14 | YIELD 2, 3, 5-trimethylphenyl 2-methylpropenyl ether |  |

---

```
Reaction no 75
```

Generated by the Chemistry Development Kit (http://github.com/cdk)

|  | A | B |
| --- | --- | --- |
| 0 | ADD 3-(1,1-dimethylindan-7-yloxy) quinolone | ADD 3-(1,1-dimethylindan-7-yloxy) quinolone |
| 1 | ADD m-chloroperbenzoic acid | ADD chloroform |
| 2 | ADD chloroform | ADD m-chloroperbenzoic acid |
| 3 | STIR for 86400 s at 25 °C | STIR for 86400 s at 25 °C |
| 4 | CONCENTRATE | ADD chloroform |
| 5 | PURIFY | WASH with NaHCO3 aq. |
| 6 | YIELD 3-(1,1-dimethylindan-7-yloxy) quinoline-N-oxide | ADD magnesium sulfate |
| 7 |  | CONCENTRATE |
| 8 |  | YIELD 3-(1,1-dimethylindan-7-yloxy) quinoline-N-oxide |

---

```
Reaction no 76
```

Generated by the Chemistry Development Kit (http://github.com/cdk)

|  | A | B |
| --- | --- | --- |
| 0 | ADD 6-bromo-4-[[(1R)-1-(oxan-4-yl)ethyl]amino]cinnoline-3-carboxamide | ADD 6-bromo-4-[[(1R)-1-(oxan-4-yl)ethyl]amino]cinnoline-3-carboxamide |
| 1 | ADD 6-fluoropyridin-3-ylboronic acid | ADD 6-fluoropyridin-3-ylboronic acid |
| 2 | ADD sodium tetrachloropalladate(II) | ADD K2CO3 |
| 3 | ADD 3-(di-tert-butylphosphino)propane-1-sulfonic acid | ADD dioxane |
| 4 | ADD K2CO3 | ADD water |
| 5 | ADD dioxane | MAKESOLUTION with sodium tetrachloropalladate(II) and 3-(di-tert-butylphosphino)propane-1-sulfonic acid |
| 6 | ADD water | STIR for 86400 s at 100 °C |
| 7 | MICROWAVE for 3600 s at 100 °C | SETTEMPERATURE 25 °C |
| 8 | CONCENTRATE | PARTITION with water and ethyl acetate |
| 9 | PURIFY | COLLECTLAYER organic |
| 10 | YIELD 6-(6-Fluoropyridin-3-yl)-N-methyl-4-[[(1R)-1-(oxan-4-yl)ethyl]amino]cinnoline-3-carboxamide | WASH with water |
| 11 |  | WASH with brine |
| 12 |  | COLLECTLAYER organic |
| 13 |  | DRYSOLUTION over MgSO4 |
| 14 |  | FILTER keep filtrate |
| 15 |  | CONCENTRATE |
| 16 |  | PURIFY |
| 17 |  | YIELD 6-(6-Fluoropyridin-3-yl)-N-methyl-4-[[(1R)-1-(oxan-4-yl)ethyl]amino]cinnoline-3-carboxamide |

---

```
Reaction no 77
```

Generated by the Chemistry Development Kit (http://github.com/cdk)

|  | A | B |
| --- | --- | --- |
| 0 | ADD (R)-(9H-fluoren-9-yl)methyl 2-(4-(((4-fluorophenyl)((2-((S)-1-((S)-3-hydroxy-2-(methoxycarbonylamino)-3-methylbutanoyl)pyrrolidin-2-yl)-1H-benzo[d]imidazol-5-yl)methyl)amino)methyl)phenylcarbamoyl)pyrrolidine-1-carboxylate at 25 °C | ADD (R)-(9H-fluoren-9-yl)methyl 2-(4-(((4-fluorophenyl)((2-((S)-1-((S)-3-hydroxy-2-(methoxycarbonylamino)-3-methylbutanoyl)pyrrolidin-2-yl)-1H-benzo[d]imidazol-5-yl)methyl)amino)methyl)phenylcarbamoyl)pyrrolidine-1-carboxylate |
| 1 | ADD acetonitrile at 25 °C | ADD acetonitrile |
| 2 | ADD diethylamine at 25 °C over 3600 s | ADD diethylamine |
| 3 | CONCENTRATE | STIR for 3600 s at 25 °C |
| 4 | YIELD methyl ‌(S)-1-((S)-2-(5-(((4-fluorophenyl)(4-((R)-pyrrolidine-2-carboxamido)benzyl)amino)methyl)-1H-benzo[d]imidazol-2-yl)pyrrolidin-1-yl)-3-hydroxy-3-methyl-1-oxobutan-2-ylcarbamate | CONCENTRATE |
| 5 |  | PURIFY |
| 6 |  | YIELD methyl ‌(S)-1-((S)-2-(5-(((4-fluorophenyl)(4-((R)-pyrrolidine-2-carboxamido)benzyl)amino)methyl)-1H-benzo[d]imidazol-2-yl)pyrrolidin-1-yl)-3-hydroxy-3-methyl-1-oxobutan-2-ylcarbamate |

---

```
Reaction no 78
```

Generated by the Chemistry Development Kit (http://github.com/cdk)

|  | A | B |
| --- | --- | --- |
| 0 | ADD (3aS,4S,6R,6aR)-6-(4-chloro-7H-pyrrolo[2,3-d]pyrimidin-7-yl)-N-methoxy-N, 2,2,3a-tetramethyltetrahydrofuro[3,4-d][1,3]dioxole-4-carboxamide | ADD (3aS,4S,6R,6aR)-6-(4-chloro-7H-pyrrolo[2,3-d]pyrimidin-7-yl)-N-methoxy-N, 2,2,3a-tetramethyltetrahydrofuro[3,4-d][1,3]dioxole-4-carboxamide |
| 1 | ADD THF | ADD THF |
| 2 | ADD 4-chlorophenylmagnesium bromide at 0 °C | ADD 4-chlorophenylmagnesium bromide at 0 °C |
| 3 | STIR for 86400 s at 25 °C | STIR for 3600 s at 0 °C |
| 4 | QUENCH with ammonium chloride | QUENCH with ammonium chloride |
| 5 | CONCENTRATE | EXTRACT with ethyl acetate |
| 6 | PURIFY | COLLECTLAYER organic |
| 7 | YIELD ((3aS,4S,6R,6aR)-6-(4-chloro-7H-pyrrolo[2,3-d]pyrimidin-7-yl)-2,2,3a-trimethyltetrahydrofuro[3,4-d][1,3]dioxol-4-yl)(4-chlorophenyl)methanone | DRYSOLUTION over Na2SO4 |
| 8 |  | FILTER keep filtrate |
| 9 |  | CONCENTRATE |
| 10 |  | PURIFY |
| 11 |  | YIELD ((3aS,4S,6R,6aR)-6-(4-chloro-7H-pyrrolo[2,3-d]pyrimidin-7-yl)-2,2,3a-trimethyltetrahydrofuro[3,4-d][1,3]dioxol-4-yl)(4-chlorophenyl)methanone |

---

```
Reaction no 79
```

Generated by the Chemistry Development Kit (http://github.com/cdk)

|  | A | B |
| --- | --- | --- |
| 0 | ADD 4-[(6,7-Dimethoxy-4-quinolyl)oxy]aniline | ADD 4-[(6,7-Dimethoxy-4-quinolyl)oxy]aniline |
| 1 | ADD toluene | ADD toluene |
| 2 | ADD triphosgene | ADD triphosgene |
| 3 | REFLUX for 28800 s | REFLUX for 600 s |
| 4 | ADD N-methylaniline | ADD N-methylaniline |
| 5 | REFLUX for 3600 s | REFLUX for 600 s |
| 6 | EXTRACT with NaHCO3 | EXTRACT with NaHCO3 |
| 7 | EXTRACT with ethyl acetate | EXTRACT with ethyl acetate |
| 8 | COLLECTLAYER organic | COLLECTLAYER organic |
| 9 | WASH with brine | WASH with brine |
| 10 | DRYSOLUTION over sodium sulfate | DRYSOLUTION over sodium sulfate |
| 11 | CONCENTRATE | CONCENTRATE |
| 12 | PURIFY | PURIFY |
| 13 | YIELD N-Methyl-N-phenyl-N'-{4-[(6,7-dimethoxy-4-quinolyl)oxy]phenyl}urea | YIELD N-Methyl-N-phenyl-N'-{4-[(6,7-dimethoxy-4-quinolyl)oxy]phenyl}urea |

---

```
Reaction no 80
```

Generated by the Chemistry Development Kit (http://github.com/cdk)

|  | A | B |
| --- | --- | --- |
| 0 | ADD (+)-2-(tert-Buyloxycarbonyl(methyl)amino)-3-methoxy-N-(benzyloxycarbonyl)morphinan | ADD (+)-2-(tert-Buyloxycarbonyl(methyl)amino)-3-methoxy-N-(benzyloxycarbonyl)morphinan |
| 1 | ADD dichloromethane | ADD 1,2-dichloroethane |
| 2 | ADD TFA at 25 °C | ADD TFA |
| 3 | STIR for 3600 s at 25 °C | STIR for 3600 s at 25 °C |
| 4 | CONCENTRATE | CONCENTRATE |
| 5 | ADD 1,2-dichloroethane | ADD NaHCO3 |
| 6 | ADD propionaldehyde | EXTRACT with ethyl acetate |
| 7 | STIR for 600 s at 25 °C | COLLECTLAYER organic |
| 8 | ADD sodium triacetoxyborohydride | DRYSOLUTION over sodium sulfate |
| 9 | STIR for 86400 s | FILTER keep filtrate |
| 10 | WASH with NaHCO3 | CONCENTRATE |
| 11 | COLLECTLAYER organic | ADD 1,2-dichloroethane |
| 12 | EXTRACT with ethyl acetate | ADD propionaldehyde |
| 13 | COLLECTLAYER organic | ADD sodium triacetoxyborohydride |
| 14 | DRYSOLUTION over MgSO4 | STIR for 86400 s at 25 °C |
| 15 | FILTER keep filtrate | ADD dichloromethane |
| 16 | CONCENTRATE | WASH with NaHCO3 |
| 17 | PURIFY | DRYSOLUTION over sodium sulfate |
| 18 | YIELD (+)-3-Methoxy-2-(methylpropylamino)-N-(benzyloxycarbonyl)morphinan | FILTER keep filtrate |
| 19 |  | CONCENTRATE |
| 20 |  | PURIFY |
| 21 |  | YIELD (+)-3-Methoxy-2-(methylpropylamino)-N-(benzyloxycarbonyl)morphinan |

---

```
Reaction no 81
```

Generated by the Chemistry Development Kit (http://github.com/cdk)

|  | A | B |
| --- | --- | --- |
| 0 | ADD 2-chloro-6-methyl-5-nitroquinoline | ADD 2-chloro-6-methyl-5-nitroquinoline |
| 1 | ADD trimethylboroxine | ADD dioxane |
| 2 | ADD Pd(dppf)Cl2 | ADD water |
| 3 | ADD K2CO3 | ADD K2CO3 |
| 4 | ADD dioxane | ADD trimethylboroxine |
| 5 | ADD water | ADD Pd(dppf)Cl2 |
| 6 | STIR for 86400 s at 100 °C | STIR for 86400 s at 100 °C |
| 7 | CONCENTRATE | SETTEMPERATURE 25 °C |
| 8 | PURIFY | FILTER keep precipitate |
| 9 | YIELD 2,6-Dimethyl-5-nitroquinoline | WASH with ethyl acetate |
| 10 |  | DRYSOLUTION |
| 11 |  | PURIFY |
| 12 |  | YIELD 2,6-Dimethyl-5-nitroquinoline |

---

```
Reaction no 82
```

Generated by the Chemistry Development Kit (http://github.com/cdk)

|  | A | B |
| --- | --- | --- |
| 0 | ADD N-[3-(chloromethyl)-5-(pentafluoro-λ6-sulfanyl)phenyl]-4-(2,4-difluorophenyl)-5-fluoropyrimidin-2-amine hydrochloride | ADD N-[3-(chloromethyl)-5-(pentafluoro-λ6-sulfanyl)phenyl]-4-(2,4-difluorophenyl)-5-fluoropyrimidin-2-amine hydrochloride |
| 1 | ADD sodium thiomethoxide | ADD ethanol |
| 2 | ADD ethanol | ADD sodium thiomethoxide at -30 °C |
| 3 | STIR for 86400 s at 25 °C | SETTEMPERATURE 25 °C |
| 4 | CONCENTRATE | STIR for 28800 s at 25 °C |
| 5 | ADD ethyl acetate | ADD sodium chloride |
| 6 | WASH with water | EXTRACT with ethyl acetate |
| 7 | DRYSOLUTION over sodium sulfate | COLLECTLAYER organic |
| 8 | CONCENTRATE | WASH with water |
| 9 | PURIFY | DRYSOLUTION over sodium sulfate |
| 10 | YIELD 4-(2,4-Difluorophenyl)-5-fluoro-N-{3-[(methylsulfanyl)methyl]-5-(pentafluoro-λ6-sulfanyl)phenyl}pyrimidin-2-amine | FILTER keep filtrate |
| 11 |  | CONCENTRATE |
| 12 |  | YIELD 4-(2,4-Difluorophenyl)-5-fluoro-N-{3-[(methylsulfanyl)methyl]-5-(pentafluoro-λ6-sulfanyl)phenyl}pyrimidin-2-amine |

---

```
Reaction no 83
```

Generated by the Chemistry Development Kit (http://github.com/cdk)

|  | A | B |
| --- | --- | --- |
| 0 | ADD (E)-4-((4-aminobut-2-en-1-yl)amino)-3-methoxy-5-nitrobenzamide, hydrochloride | ADD (E)-4-((4-aminobut-2-en-1-yl)amino)-3-methoxy-5-nitrobenzamide, hydrochloride |
| 1 | ADD n-butanol | ADD n-butanol |
| 2 | ADD NaHCO3 | ADD 3-(3-((tert-butyldimethylsilyl)oxy)propoxy)-4-chloro-5-nitrobenzamide |
| 3 | ADD DIPEA | ADD DIPEA |
| 4 | STIR for 600 s at 25 °C | STIR for 86400 s at 100 °C |
| 5 | ADD 3-(3-((tert-butyldimethylsilyl)oxy)propoxy)-4-chloro-5-nitrobenzamide | ADD NaHCO3 |
| 6 | STIR for 86400 s at 100 °C | EXTRACT with ethyl acetate |
| 7 | SETTEMPERATURE 25 °C | COLLECTLAYER organic |
| 8 | FILTER keep precipitate | WASH with water |
| 9 | WASH with ethanol | WASH with brine |
| 10 | ADD water | DRYSOLUTION over sodium sulfate |
| 11 | STIR for 600 s | FILTER keep filtrate |
| 12 | FILTER keep precipitate | CONCENTRATE |
| 13 | WASH with water | PURIFY |
| 14 | WASH with ethyl acetate | YIELD (E)-3-(3-((tert-butyldimethylsilyl)oxy)propoxy)-4-((4-((4-carbamoyl-2-methoxy-6-nitrophenyl)amino)but-2-en-1-yl)amino)-5-nitrobenzamide |
| 15 | WASH with ethanol |  |
| 16 | DRYSOLID under vacuum |  |
| 17 | YIELD (E)-3-(3-((tert-butyldimethylsilyl)oxy)propoxy)-4-((4-((4-carbamoyl-2-methoxy-6-nitrophenyl)amino)but-2-en-1-yl)amino)-5-nitrobenzamide |  |

---

```
Reaction no 84
```

Generated by the Chemistry Development Kit (http://github.com/cdk)

|  | A | B |
| --- | --- | --- |
| 0 | ADD (R)-3-{I-[6-(4-azidomethyl-phenoxy)-2-methyl-pyridin-3-ylmethyl]-piperidin-4-yl}-4-phenyl-oxazolidin-2-one | ADD (R)-3-{I-[6-(4-azidomethyl-phenoxy)-2-methyl-pyridin-3-ylmethyl]-piperidin-4-yl}-4-phenyl-oxazolidin-2-one |
| 1 | ADD THF | ADD THF |
| 2 | ADD triphenylphosphine | ADD triphenylphosphine |
| 3 | STIR for 86400 s at 25 °C | STIR for 86400 s at 25 °C |
| 4 | CONCENTRATE | YIELD (R)-3-{1-[6-(4-aminomethyl-phenoxy)-2-methyl-pyridin-3-ylmethyl]-piperidin-4-yl}-4-phenyl-oxazolidin-2-one |
| 5 | PURIFY |  |
| 6 | YIELD (R)-3-{1-[6-(4-aminomethyl-phenoxy)-2-methyl-pyridin-3-ylmethyl]-piperidin-4-yl}-4-phenyl-oxazolidin-2-one |  |

---

```
Reaction no 85
```

Generated by the Chemistry Development Kit (http://github.com/cdk)

|  | A | B |
| --- | --- | --- |
| 0 | ADD 2-phenylbutanedioic acid | ADD 2-phenylbutanedioic acid |
| 1 | ADD toluene | ADD acetyl chloride |
| 2 | ADD acetyl chloride | ADD toluene |
| 3 | REFLUX for 28800 s | REFLUX for 28800 s |
| 4 | CONCENTRATE | FILTER keep filtrate |
| 5 | YIELD phenylsuccinic anhydride | SETTEMPERATURE 25 °C |
| 6 |  | CONCENTRATE |
| 7 |  | RECRYSTALLIZE from ether |
| 8 |  | YIELD phenylsuccinic anhydride |

---

```
Reaction no 86
```

Generated by the Chemistry Development Kit (http://github.com/cdk)

|  | A | B |
| --- | --- | --- |
| 0 | ADD 3-morpholin-4-yl-5-nitro-benzamide | ADD 3-morpholin-4-yl-5-nitro-benzamide |
| 1 | ADD trimethyl phosphate | ADD trimethyl phosphate |
| 2 | ADD diphosgene | ADD diphosgene dropwise at 0 °C |
| 3 | STIR for 3600 s at 100 °C | STIR for 86400 s at 60 °C |
| 4 | SETTEMPERATURE 25 °C | SETTEMPERATURE 25 °C |
| 5 | ADD water | ADD water |
| 6 | EXTRACT with ethyl acetate | FILTER keep precipitate |
| 7 | COLLECTLAYER organic | WASH with water |
| 8 | WASH with brine | DRYSOLID |
| 9 | DRYSOLUTION over sodium sulfate | PURIFY |
| 10 | CONCENTRATE | YIELD 3-morpholin-4-yl-5-nitro-benzonitrile |
| 11 | PURIFY |  |
| 12 | YIELD 3-morpholin-4-yl-5-nitro-benzonitrile |  |

---

```
Reaction no 87
```

Generated by the Chemistry Development Kit (http://github.com/cdk)

|  | A | B |
| --- | --- | --- |
| 0 | ADD 3-iodo-1-(4-methylbenzenesulfonyl)-1H-pyrrolo[2,3-b]pyridine-5-carboxylic acid | ADD 3-iodo-1-(4-methylbenzenesulfonyl)-1H-pyrrolo[2,3-b]pyridine-5-carboxylic acid |
| 1 | ADD thionyl chloride | ADD thionyl chloride |
| 2 | REFLUX for 3600 s | REFLUX for 3600 s |
| 3 | CONCENTRATE | CONCENTRATE |
| 4 | ADD acetonitrile | ADD dichloromethane |
| 5 | ADD N-methylallylamine dropwise | ADD N-methylallylamine |
| 6 | STIR for 604800 s at 25 °C | STIR for 3600 s at 25 °C |
| 7 | CONCENTRATE | CONCENTRATE |
| 8 | ADD ethyl acetate | PURIFY |
| 9 | WASH with water | YIELD 3-iodo-N-methyl-1-(4-methylbenzenesulfonyl)-N-(prop-2-en-1-yl)-1H-pyrrolo[2,3-b]pyridine-5-carboxamide |
| 10 | COLLECTLAYER organic |  |
| 11 | DRYSOLUTION over Na2SO4 |  |
| 12 | CONCENTRATE |  |
| 13 | YIELD 3-iodo-N-methyl-1-(4-methylbenzenesulfonyl)-N-(prop-2-en-1-yl)-1H-pyrrolo[2,3-b]pyridine-5-carboxamide |  |

---

```
Reaction no 88
```

Generated by the Chemistry Development Kit (http://github.com/cdk)

|  | A | B |
| --- | --- | --- |
| 0 | ADD (COCl)2 | ADD 2-(4-chloro-3-fluorophenyl)-4,5-di(benzylamino)-6-methoxycarbonyl-pyrimidine |
| 1 | ADD dichlorobenzene | ADD dichlorobenzene |
| 2 | MAKESOLUTION with 2-(4-chloro-3-fluorophenyl)-4,5-di(benzylamino)-6-methoxycarbonyl-pyrimidine and dichlorobenzene | ADD (COCl)2 |
| 3 | ADD SLN | SETTEMPERATURE 100 °C |
| 4 | STIR for 3600 s at 100 °C | STIR for 3600 s |
| 5 | SETTEMPERATURE 25 °C | SETTEMPERATURE 25 °C |
| 6 | ADD ethyl acetate | ADD ethyl acetate |
| 7 | WASH with water | WASH with water |
| 8 | WASH with brine | WASH with brine |
| 9 | DRYSOLUTION over magnesium sulfate | DRYSOLUTION over sodium sulfate |
| 10 | FILTER keep filtrate | FILTER keep filtrate |
| 11 | CONCENTRATE | CONCENTRATE |
| 12 | PURIFY | PURIFY |
| 13 | YIELD 2-(4-chloro-3-fluorophenyl)-5,8-dibenzyl-6,7-dioxo-4-methoxycarbonyl-5,6,7,8-tetrahydropteridine | YIELD 2-(4-chloro-3-fluorophenyl)-5,8-dibenzyl-6,7-dioxo-4-methoxycarbonyl-5,6,7,8-tetrahydropteridine |

---

```
Reaction no 89
```

Generated by the Chemistry Development Kit (http://github.com/cdk)

|  | A | B |
| --- | --- | --- |
| 0 | ADD Boc-glycine | ADD Boc-glycine |
| 1 | ADD DMF | ADD DMF |
| 2 | ADD HATU at 25 °C | ADD DIPEA |
| 3 | ADD DIPEA at 25 °C | ADD HATU |
| 4 | STIR for 600 s at 25 °C | STIR for 600 s at 25 °C |
| 5 | ADD ethyl 5-[(2-aminothiazol-5-yl)sulfonylamino]thiazole-4-carboxylate, hydrochloride at 25 °C | ADD ethyl 5-[(2-aminothiazol-5-yl)sulfonylamino]thiazole-4-carboxylate, hydrochloride |
| 6 | STIR for 86400 s at 25 °C | STIR for 86400 s at 25 °C |
| 7 | ADD water | CONCENTRATE |
| 8 | EXTRACT with dichloromethane | TRITURATE with water |
| 9 | COLLECTLAYER organic | FILTER keep precipitate |
| 10 | DRYSOLUTION over Na2SO4 | WASH with water |
| 11 | FILTER keep filtrate | WASH with ether |
| 12 | CONCENTRATE | YIELD ethyl 5-[[2-[[2-(tert-butoxycarbonylamino)acetyl]amino]thiazol-5-yl]sulfonylamino]thiazole-4-carboxylate |
| 13 | PURIFY |  |
| 14 | YIELD ethyl 5-[[2-[[2-(tert-butoxycarbonylamino)acetyl]amino]thiazol-5-yl]sulfonylamino]thiazole-4-carboxylate |  |

---

```
Reaction no 90
```

Generated by the Chemistry Development Kit (http://github.com/cdk)

|  | A | B |
| --- | --- | --- |
| 0 | ADD 3-bromophenylacetonitrile | ADD 3-bromophenylacetonitrile |
| 1 | ADD 1-(2-Chloroethyl)-2-(chloromethyl)benzene | ADD THF |
| 2 | ADD THF | ADD NaH at 0 °C |
| 3 | ADD NaH at 0 °C | STIR for 600 s |
| 4 | STIR for 86400 s at 25 °C | MAKESOLUTION with 1-(2-Chloroethyl)-2-(chloromethyl)benzene and THF |
| 5 | QUENCH with water | ADD SLN |
| 6 | EXTRACT with ethyl acetate | SETTEMPERATURE 25 °C |
| 7 | COLLECTLAYER organic | STIR for 3600 s at 60 °C |
| 8 | DRYSOLUTION over Na2SO4 | QUENCH with ammonium chloride |
| 9 | FILTER keep filtrate | ADD ethyl acetate |
| 10 | CONCENTRATE | WASH with water |
| 11 | PURIFY | WASH with brine |
| 12 | YIELD 2-(3-bromophenyl)-1,2,3,4-tetrahydronaphthalene-2-carbonitrile | DRYSOLUTION over Na2SO4 |
| 13 |  | FILTER keep filtrate |
| 14 |  | CONCENTRATE |
| 15 |  | YIELD 2-(3-bromophenyl)-1,2,3,4-tetrahydronaphthalene-2-carbonitrile |

---

```
Reaction no 91
```

Generated by the Chemistry Development Kit (http://github.com/cdk)

|  | A | B |
| --- | --- | --- |
| 0 | ADD 2-(4-chlorophenyl)-5-hydroxy-3-methoxy-7-(methoxymethoxy)-8-(3-methylbut-2-enyl)-4H-chromen-4-one | ADD 2-(4-chlorophenyl)-5-hydroxy-3-methoxy-7-(methoxymethoxy)-8-(3-methylbut-2-enyl)-4H-chromen-4-one |
| 1 | ADD HCl | ADD HCl |
| 2 | ADD isopropanol | ADD isopropanol |
| 3 | STIR for 3600 s at 60 °C | STIR for 3600 s at 60 °C |
| 4 | SETTEMPERATURE 25 °C | SETTEMPERATURE 25 °C |
| 5 | EXTRACT with ethyl acetate | EXTRACT with ethyl acetate |
| 6 | COLLECTLAYER organic | WASH with ethyl acetate |
| 7 | WASH with brine | WASH with water |
| 8 | DRYSOLUTION over MgSO4 | DRYSOLUTION over Na2SO4 |
| 9 | FILTER keep filtrate | CONCENTRATE |
| 10 | CONCENTRATE | PURIFY |
| 11 | PURIFY | YIELD 2-(4-chlorophenyl)-5,7-dihydroxy-3-methoxy-8-(3-methylbut-2-enyl)-4H-chromen-4-one |
| 12 | YIELD 2-(4-chlorophenyl)-5,7-dihydroxy-3-methoxy-8-(3-methylbut-2-enyl)-4H-chromen-4-one |  |

---

```
Reaction no 92
```

Generated by the Chemistry Development Kit (http://github.com/cdk)

|  | A | B |
| --- | --- | --- |
| 0 | ADD (R)—N-[5-(2-Azido-1-hydroxyethyl)-2-(benzyloxy)phenyl]formamide | ADD (R)—N-[5-(2-Azido-1-hydroxyethyl)-2-(benzyloxy)phenyl]formamide |
| 1 | ADD methanol | ADD dichloromethane |
| 2 | ADD dichloromethane | ADD methanol |
| 3 | ADD Pd/C | ADD Pd/C |
| 4 | STIR for 86400 s at 60 °C | STIR for 3600 s at 25 °C |
| 5 | FILTER keep precipitate | FILTER keep filtrate |
| 6 | WASH with methanol | CONCENTRATE |
| 7 | CONCENTRATE | YIELD N-[5-[(lR)-2-amino-1-hydroxy-ethyl]-2-hydroxy-phenyl]formamide |
| 8 | YIELD N-[5-[(lR)-2-amino-1-hydroxy-ethyl]-2-hydroxy-phenyl]formamide |  |

---

```
Reaction no 93
```

Generated by the Chemistry Development Kit (http://github.com/cdk)

|  | A | B |
| --- | --- | --- |
| 0 | ADD 4-Aminobutyraldehyde diethyl acetal | ADD 4-Aminobutyraldehyde diethyl acetal |
| 1 | ADD pyridine | ADD pyridine |
| 2 | ADD dichloromethane | ADD dichloromethane |
| 3 | ADD ethyl chloroformate at 0 °C | ADD ethyl chloroformate dropwise at 0 °C |
| 4 | STIR for 86400 s at 25 °C | MAKESOLUTION with 4-Aminobutyraldehyde diethyl acetal and pyridine and dichloromethane |
| 5 | ADD water | ADD SLN |
| 6 | EXTRACT with dichloromethane | STIR for 3600 s at 0 °C |
| 7 | COLLECTLAYER organic | ADD water |
| 8 | WASH with brine | EXTRACT with ether |
| 9 | DRYSOLUTION over sodium sulfate | DRYSOLUTION over Na2SO4 |
| 10 | CONCENTRATE | CONCENTRATE |
| 11 | PURIFY | YIELD Ethyl N-(4,4-diethoxybutyl)carbamate |
| 12 | YIELD Ethyl N-(4,4-diethoxybutyl)carbamate |  |

---

```
Reaction no 94
```

Generated by the Chemistry Development Kit (http://github.com/cdk)

|  | A | B |
| --- | --- | --- |
| 0 | ADD Pd(OAc)2 | ADD mono-methyl succinate |
| 1 | ADD triphenylphosphine | ADD (3-{[tert-butyl(dimethyl)silyl]oxy}phenyl)boronic acid |
| 2 | ADD THF | ADD triphenylphosphine |
| 3 | STIR for 600 s at 25 °C | ADD Pd(OAc)2 |
| 4 | ADD (3-{[tert-butyl(dimethyl)silyl]oxy}phenyl)boronic acid | ADD THF |
| 5 | ADD mono-methyl succinate | ADD water |
| 6 | ADD pivalic anhydride | ADD pivalic anhydride |
| 7 | STIR for 86400 s at 60 °C | STIR for 86400 s at 60 °C |
| 8 | SETTEMPERATURE 25 °C | CONCENTRATE |
| 9 | ADD water | ADD water |
| 10 | EXTRACT with ethyl acetate | ADD NaHCO3 |
| 11 | COLLECTLAYER organic | EXTRACT with ethyl acetate |
| 12 | DRYSOLUTION over sodium sulfate | COLLECTLAYER organic |
| 13 | CONCENTRATE | WASH with brine |
| 14 | PURIFY | DRYSOLUTION over sodium sulfate |
| 15 | YIELD 4-(3-tert-butyldimethylsilyloxophenyl)-4-oxobutyric acid methyl ester | CONCENTRATE |
| 16 |  | PURIFY |
| 17 |  | YIELD 4-(3-tert-butyldimethylsilyloxophenyl)-4-oxobutyric acid methyl ester |

---

```
Reaction no 95
```

Generated by the Chemistry Development Kit (http://github.com/cdk)

|  | A | B |
| --- | --- | --- |
| 0 | ADD Methyl ‌(±)-3-(5-methylthiazol-2-yl)-4-(4-hydroxyphenyl)butanoate | ADD Methyl ‌(±)-3-(5-methylthiazol-2-yl)-4-(4-hydroxyphenyl)butanoate |
| 1 | ADD 6-(methylamino)-2-pyridylethanol | ADD 6-(methylamino)-2-pyridylethanol |
| 2 | ADD triphenylphosphine | ADD triphenylphosphine |
| 3 | ADD MTBE | ADD MTBE |
| 4 | ADD diisopropyl azodicarboxylate at 0 °C | ADD diisopropyl azodicarboxylate at 0 °C |
| 5 | STIR for 86400 s at 25 °C | STIR for 604800 s at 25 °C |
| 6 | FILTER keep precipitate | CONCENTRATE |
| 7 | WASH with MTBE | PURIFY |
| 8 | YIELD Methyl(±)-4-[4-[2-(6-Methylaminopyridin-2-yl)-1-ethoxy]phenyl]-3-(5-methylthiazol2-yl)butanoate | COLLECTLAYER organic |
| 9 |  | CONCENTRATE |
| 10 |  | YIELD Methyl(±)-4-[4-[2-(6-Methylaminopyridin-2-yl)-1-ethoxy]phenyl]-3-(5-methylthiazol2-yl)butanoate |

---

```
Reaction no 96
```

Generated by the Chemistry Development Kit (http://github.com/cdk)

|  | A | B |
| --- | --- | --- |
| 0 | ADD Ethyl 3-(3-bromobenzyl)-4-(4-cyanophenyl)-6-methyl-2-oxo-1-[3-(trifluoromethyl)phenyl]-1,2,3,4-tetrahydropyrimidine-5-carboxylate | ADD Ethyl 3-(3-bromobenzyl)-4-(4-cyanophenyl)-6-methyl-2-oxo-1-[3-(trifluoromethyl)phenyl]-1,2,3,4-tetrahydropyrimidine-5-carboxylate |
| 1 | ADD thiophene-2-boronic acid | ADD thiophene-2-boronic acid |
| 2 | ADD palladium bis(triphenylphosphine) dichloride | ADD Na2CO3 |
| 3 | ADD Na2CO3 | ADD palladium bis(triphenylphosphine) dichloride |
| 4 | ADD DMF | ADD DMF |
| 5 | STIR for 86400 s at 25 °C | STIR for 86400 s at 100 °C |
| 6 | PARTITION with ethyl acetate and water | ADD palladium bis(triphenylphosphine) dichloride |
| 7 | COLLECTLAYER organic | STIR for 86400 s at 100 °C |
| 8 | WASH with water | ADD palladium bis(triphenylphosphine) dichloride |
| 9 | WASH with brine | STIR for 28800 s at 100 °C |
| 10 | DRYSOLUTION over MgSO4 | ADD DMF |
| 11 | CONCENTRATE | PURIFY |
| 12 | PURIFY | CONCENTRATE |
| 13 | YIELD Ethyl 4-(4-cyanophenyl)-6-methyl-2-oxo-3-[3-(2-thienyl)benzyl]-1-[3-(trifluoromethyl)phenyl]-1,2,3,4-tetrahydropyrimidine-5-carboxylate | CONCENTRATE |
| 14 |  | YIELD Ethyl 4-(4-cyanophenyl)-6-methyl-2-oxo-3-[3-(2-thienyl)benzyl]-1-[3-(trifluoromethyl)phenyl]-1,2,3,4-tetrahydropyrimidine-5-carboxylate |

---

```
Reaction no 97
```

Generated by the Chemistry Development Kit (http://github.com/cdk)

|  | A | B |
| --- | --- | --- |
| 0 | ADD 2-mercapto-3-methylthieno[3,2-d]-pyrimidin-4(3H)-one | ADD 2-mercapto-3-methylthieno[3,2-d]-pyrimidin-4(3H)-one |
| 1 | ADD 4,4'-bis(bromomethyl)benzophenone | ADD 4,4'-bis(bromomethyl)benzophenone |
| 2 | ADD 1-phenylpiperazine | ADD 1-phenylpiperazine |
| 3 | ADD K2CO3 | ADD K2CO3 |
| 4 | ADD DMF | ADD DMF |
| 5 | STIR for 3600 s at 60 °C | STIR for 86400 s at 25 °C |
| 6 | CONCENTRATE | CONCENTRATE |
| 7 | ADD ethyl acetate | ADD water |
| 8 | WASH with water | EXTRACT with chloroform |
| 9 | DRYSOLUTION | WASH with water |
| 10 | CONCENTRATE | DRYSOLUTION |
| 11 | PURIFY | CONCENTRATE |
| 12 | YIELD 3-Methyl-2-[4-[4-(4-phenylpiperazinylmethyl)benzoyl]benzylthio]thieno[3,2-d]pyrimidin-4(3H)-one | PURIFY |
| 13 |  | YIELD 3-Methyl-2-[4-[4-(4-phenylpiperazinylmethyl)benzoyl]benzylthio]thieno[3,2-d]pyrimidin-4(3H)-one |

---

```
Reaction no 98
```

Generated by the Chemistry Development Kit (http://github.com/cdk)

|  | A | B |
| --- | --- | --- |
| 0 | ADD morpholine | ADD ethyl 2-chloroacetoacetate |
| 1 | ADD acetonitrile | ADD morpholine |
| 2 | ADD K2CO3 | ADD K2CO3 |
| 3 | ADD ethyl 2-chloroacetoacetate dropwise over 3600 s | ADD acetonitrile |
| 4 | CONCENTRATE | STIR for 86400 s at 25 °C |
| 5 | PURIFY | FILTER keep filtrate |
| 6 | YIELD ethyl 2-morpholino-3-oxobutanoate | CONCENTRATE |
| 7 |  | PURIFY |
| 8 |  | YIELD ethyl 2-morpholino-3-oxobutanoate |

---

```
Reaction no 99
```

Generated by the Chemistry Development Kit (http://github.com/cdk)

|  | A | B |
| --- | --- | --- |
| 0 | ADD 4,6-diamino-2-ethoxy-nicotinonitrile | ADD 4,6-diamino-2-ethoxy-nicotinonitrile |
| 1 | ADD THF | ADD DIPEA |
| 2 | ADD DIPEA | ADD THF |
| 3 | ADD methoxyacetyl chloride | ADD methoxyacetyl chloride at 0 °C |
| 4 | STIR for 600 s | STIR for 86400 s at 25 °C |
| 5 | ADD water | ADD water |
| 6 | FILTER keep precipitate | EXTRACT with ethyl acetate |
| 7 | WASH with water | COLLECTLAYER organic |
| 8 | RECRYSTALLIZE from methanol | WASH with brine |
| 9 | YIELD N-(4-amino-5-cyano-6-ethoxypyridin-2-yl)-2-methoxyacetamide | DRYSOLUTION over magnesium sulfate |
| 10 |  | CONCENTRATE |
| 11 |  | PURIFY |
| 12 |  | YIELD N-(4-amino-5-cyano-6-ethoxypyridin-2-yl)-2-methoxyacetamide |

---

```
Reaction no 100
```

Generated by the Chemistry Development Kit (http://github.com/cdk)

|  | A | B |
| --- | --- | --- |
| 0 | ADD N-(6,6-dimethyl-2-hepten-4-ynyl)methylamine | ADD DMF |
| 1 | ADD 3'-bromomethylpropiophenone | ADD N-(6,6-dimethyl-2-hepten-4-ynyl)methylamine |
| 2 | ADD Na2CO3 | ADD Na2CO3 |
| 3 | ADD DMF | SETTEMPERATURE 25 °C |
| 4 | STIR for 86400 s at 25 °C | MAKESOLUTION with 3'-bromomethylpropiophenone and DMF |
| 5 | PARTITION with ether and water | ADD SLN dropwise |
| 6 | COLLECTLAYER organic | STIR for 3600 s at 25 °C |
| 7 | WASH with water | ADD trans-3'-[N-(6,6-dimethyl-2-hepten-4-ynyl)-N-methylaminomethyl]propiophenone |
| 8 | WASH with brine | EXTRACT with ethyl acetate |
| 9 | DRYSOLUTION over MgSO4 | COLLECTLAYER organic |
| 10 | CONCENTRATE | WASH with sodium bicarbonate |
| 11 | PURIFY | WASH with brine |
| 12 | YIELD trans-3'-[N-(6,6-dimethyl-2-hepten-4-ynyl)-N-methylaminomethyl]propiophenone | DRYSOLUTION over sodium sulfate |
| 13 |  | CONCENTRATE |
| 14 |  | PURIFY |

---

```
Reaction no 101
```

Generated by the Chemistry Development Kit (http://github.com/cdk)

|  | A | B |
| --- | --- | --- |
| 0 | ADD ethyl 4-(cyclohexylamino)-3-nitrobenzoate | ADD ethyl 4-(cyclohexylamino)-3-nitrobenzoate |
| 1 | ADD ethanol | ADD ethanol |
| 2 | ADD Pd/C | ADD cyclohexene |
| 3 | ADD carbon | ADD Pd/C |
| 4 | ADD cyclohexene | REFLUX for 3600 s |
| 5 | STIR for 28800 s at 100 °C | FILTER keep filtrate |
| 6 | FILTER keep filtrate | CONCENTRATE |
| 7 | WASH with ethanol | YIELD ethyl 3-amino-4-(cyclohexylamino)benzoate |
| 8 | CONCENTRATE |  |
| 9 | YIELD ethyl 3-amino-4-(cyclohexylamino)benzoate |  |

---

```
Reaction no 102
```

Generated by the Chemistry Development Kit (http://github.com/cdk)

|  | A | B |
| --- | --- | --- |
| 0 | ADD ethyl 5-methoxybenzofuran-2-carboxylic acid | ADD ethyl 5-methoxybenzofuran-2-carboxylic acid |
| 1 | ADD dichloromethane | ADD dichloromethane |
| 2 | ADD boron tribromide at 0 °C | ADD dichloromethane |
| 3 | STIR for 86400 s at 25 °C | ADD boron tribromide at 0 °C |
| 4 | QUENCH with water | STIR for 3600 s at 25 °C |
| 5 | EXTRACT with dichloromethane | ADD water |
| 6 | COLLECTLAYER organic | STIR for 3600 s |
| 7 | WASH with brine | EXTRACT with ethyl acetate |
| 8 | DRYSOLUTION over sodium sulfate | COLLECTLAYER organic |
| 9 | CONCENTRATE | WASH with water |
| 10 | PURIFY | WASH with brine |
| 11 | YIELD 5-hydroxy-benzofuran-2-carboxylic acid | DRYSOLUTION over sodium sulfate |
| 12 |  | CONCENTRATE |
| 13 |  | YIELD 5-hydroxy-benzofuran-2-carboxylic acid |

---

```
Reaction no 103
```

Generated by the Chemistry Development Kit (http://github.com/cdk)

|  | A | B |
| --- | --- | --- |
| 0 | ADD 1-(4-hydroxy-3,5-di-tert.-butylbenzylideneamino)-2-methylmercaptoimidazole | ADD 1-(4-hydroxy-3,5-di-tert.-butylbenzylideneamino)-2-methylmercaptoimidazole |
| 1 | ADD acetic acid | ADD acetic acid |
| 2 | ADD sodium cyanoborohydride | ADD sodium cyanoborohydride over 3600 s |
| 3 | STIR for 3600 s at 25 °C | STIR for 86400 s at 25 °C |
| 4 | CONCENTRATE | CONCENTRATE |
| 5 | ADD ethyl acetate | ADD water |
| 6 | WASH with NaHCO3 | ADD sodium carbonate |
| 7 | WASH with brine | EXTRACT with dichloromethane |
| 8 | DRYSOLUTION over sodium sulfate | COLLECTLAYER organic |
| 9 | CONCENTRATE | DRYSOLUTION over sodium sulfate |
| 10 | PURIFY | FILTER keep filtrate |
| 11 | YIELD 1-(4-hydroxy-3,5-di-tert.-butylbenzylamino)-2-methylmercaptoimidazole | CONCENTRATE |
| 12 |  | RECRYSTALLIZE from methanol / water |
| 13 |  | YIELD 1-(4-hydroxy-3,5-di-tert.-butylbenzylamino)-2-methylmercaptoimidazole |

---

```
Reaction no 104
```

Generated by the Chemistry Development Kit (http://github.com/cdk)

|  | A | B |
| --- | --- | --- |
| 0 | ADD methyl 5-cyano-5-(naphthalen-2-yl)-2-oxocyclohexanecarboxylate | ADD methyl 5-cyano-5-(naphthalen-2-yl)-2-oxocyclohexanecarboxylate |
| 1 | ADD DMSO | ADD brine |
| 2 | ADD water | ADD DMSO |
| 3 | ADD brine | SETTEMPERATURE 100 °C |
| 4 | STIR for 28800 s at 100 °C | STIR for 86400 s |
| 5 | SETTEMPERATURE 25 °C | WAIT for 86400 s |
| 6 | ADD water | SETTEMPERATURE 25 °C |
| 7 | EXTRACT with ether | ADD water |
| 8 | COLLECTLAYER organic | EXTRACT with MTBE |
| 9 | WASH with water | COLLECTLAYER organic |
| 10 | WASH with brine | WASH with brine |
| 11 | DRYSOLUTION over magnesium sulfate | DRYSOLUTION over MgSO4 |
| 12 | CONCENTRATE | FILTER keep filtrate |
| 13 | YIELD heptane ethyl acetate | CONCENTRATE |
| 14 | PURIFY | TRITURATE with heptane ethyl acetate |
| 15 | YIELD 1-(naphthalen-2-yl)-4-oxocyclohexanecarbonitrile | STIR for 3600 s |
| 16 |  | FILTER keep precipitate |
| 17 |  | DRYSOLID for 86400 s under vacuum |
| 18 |  | YIELD 1-(naphthalen-2-yl)-4-oxocyclohexanecarbonitrile |

---

```
Reaction no 105
```

Generated by the Chemistry Development Kit (http://github.com/cdk)

|  | A | B |
| --- | --- | --- |
| 0 | ADD 4-chlorothieno[3,2-d]pyrimidine | ADD 4-chlorothieno[3,2-d]pyrimidine |
| 1 | ADD 5,6-dimethyl-[2,2']bipyridinyl-3-ol | ADD 5,6-dimethyl-[2,2']bipyridinyl-3-ol |
| 2 | ADD DMAP | ADD DMAP |
| 3 | ADD DMSO | ADD DMSO |
| 4 | ADD Cs2CO3 | ADD Cs2CO3 |
| 5 | STIR for 86400 s at 100 °C | STIR for 86400 s at 100 °C |
| 6 | SETTEMPERATURE 25 °C | SETTEMPERATURE 25 °C |
| 7 | ADD water | ADD water |
| 8 | COLLECTLAYER organic | COLLECTLAYER organic |
| 9 | EXTRACT with chloroform | EXTRACT with chloroform |
| 10 | WASH with water | WASH with water |
| 11 | WASH with brine | WASH with brine |
| 12 | DRYSOLUTION over sodium sulfate | DRYSOLUTION over sodium sulfate |
| 13 | CONCENTRATE | CONCENTRATE |
| 14 | PURIFY | PURIFY |
| 15 | YIELD 5,6-Dimethyl-3-(thieno[3,2-d]pyrimidin-4-yloxy)-[2,2']bipyridine | YIELD 5,6-Dimethyl-3-(thieno[3,2-d]pyrimidin-4-yloxy)-[2,2']bipyridine |

---

```
Reaction no 106
```

Generated by the Chemistry Development Kit (http://github.com/cdk)

|  | A | B |
| --- | --- | --- |
| 0 | ADD 4-cyanophenol | ADD NaH |
| 1 | ADD hexamethylphosphorous triamide | ADD hexamethylphosphorous triamide |
| 2 | ADD NaH | MAKESOLUTION with 4-cyanophenol and hexamethylphosphorous triamide |
| 3 | STIR for 600 s at 25 °C | ADD SLN dropwise at 0 °C |
| 4 | ADD 2,2,2-trifluoroethyl methanesulfonate | STIR for 3600 s at 25 °C |
| 5 | STIR for 86400 s at 100 °C | MAKESOLUTION with 2,2,2-trifluoroethyl methanesulfonate and hexamethylphosphorous triamide |
| 6 | SETTEMPERATURE 25 °C | ADD SLN |
| 7 | ADD ice water | STIR for 3600 s at 100 °C |
| 8 | ADD HCl | SETTEMPERATURE 25 °C |
| 9 | EXTRACT with ether | ADD ice water |
| 10 | COLLECTLAYER organic | PH with HCl to pH acidic |
| 11 | WASH with water | EXTRACT with ether |
| 12 | WASH with sodium hydroxide | COLLECTLAYER organic |
| 13 | WASH with brine | WASH with water |
| 14 | DRYSOLUTION over Na2SO4 | DRYSOLUTION over magnesium sulfate |
| 15 | FILTER keep filtrate | CONCENTRATE |
| 16 | CONCENTRATE | YIELD 4-(2,2,2-trifluoroethoxy)benzonitrile |
| 17 | PURIFY |  |
| 18 | YIELD 4-(2,2,2-trifluoroethoxy)benzonitrile |  |

---

```
Reaction no 107
```

Generated by the Chemistry Development Kit (http://github.com/cdk)

|  | A | B |
| --- | --- | --- |
| 0 | ADD (COCl)2 | ADD (COCl)2 |
| 1 | ADD dichloromethane | ADD dichloromethane |
| 2 | MAKESOLUTION with DMSO and dichloromethane | ADD DMSO at -70 °C |
| 3 | ADD SLN at -70 °C | STIR for 600 s |
| 4 | STIR for 3600 s at -70 °C | MAKESOLUTION with (±)-cis-1,3-dihydro-1-(1-{4-[(5-pyrimidinyl)hydroxymethyl]cyclohex-1-yl}piperidin-4-yl)-2H -benzimidazol-2-one and dichloromethane |
| 5 | MAKESOLUTION with (±)-cis-1,3-dihydro-1-(1-{4-[(5-pyrimidinyl)hydroxymethyl]cyclohex-1-yl}piperidin-4-yl)-2H -benzimidazol-2-one and dichloromethane | ADD SLN at -70 °C |
| 6 | ADD SLN | STIR for 600 s at -70 °C |
| 7 | STIR for 3600 s at -70 °C | ADD triethylamine |
| 8 | ADD triethylamine | SETTEMPERATURE 25 °C |
| 9 | STIR for 86400 s at 25 °C | ADD water |
| 10 | ADD water | STIR for 600 s |
| 11 | PH with Na2CO3 to pH basic | ADD Na2CO3 |
| 12 | COLLECTLAYER aqueous | EXTRACT with dichloromethane |
| 13 | EXTRACT with dichloromethane | COLLECTLAYER organic |
| 14 | COLLECTLAYER organic | DRYSOLUTION over MgSO4 |
| 15 | DRYSOLUTION over magnesium sulfate | CONCENTRATE |
| 16 | FILTER keep filtrate | PURIFY |
| 17 | CONCENTRATE | TRITURATE with dichloromethane |
| 18 | PURIFY | TRITURATE with methanol |
| 19 | YIELD trans-1,3-dihydro-1-{1-[4-(5-pyrimidinylcarbonyl)cyclohex-1-yl]piperidin-4-yl}-2H-benzimidazol-2-one | YIELD trans-1,3-dihydro-1-{1-[4-(5-pyrimidinylcarbonyl)cyclohex-1-yl]piperidin-4-yl}-2H-benzimidazol-2-one |
| 20 | YIELD ammonium hydroxide | RECRYSTALLIZE from ethanol / chloroform |
| 21 |  | YIELD ammonium hydroxide |

---

```
Reaction no 108
```

Generated by the Chemistry Development Kit (http://github.com/cdk)

|  | A | B |
| --- | --- | --- |
| 0 | ADD 4-hydroxy-6-methyl-2-pyrone | ADD 4-hydroxy-6-methyl-2-pyrone |
| 1 | ADD 4-(2-aminoethyl)benzoic acid hydrochloride | ADD water |
| 2 | ADD methanol | ADD NaOH |
| 3 | ADD water | STIR for 600 s at 25 °C |
| 4 | ADD NaOH | ADD 4-(2-aminoethyl)benzoic acid hydrochloride |
| 5 | STIR for 86400 s at 60 °C | STIR for 86400 s at 100 °C |
| 6 | SETTEMPERATURE 25 °C | SETTEMPERATURE 25 °C |
| 7 | PH with HCl to pH acidic | ADD HCl |
| 8 | FILTER keep precipitate | ADD methanol |
| 9 | WASH with water | STIR for 3600 s |
| 10 | DRYSOLID under vacuum | FILTER keep precipitate |
| 11 | YIELD 4-[2-(4-hydroxy-6-methyl-2-oxopyridin-1(2H)-yl)ethyl]benzoic acid | YIELD 4-[2-(4-hydroxy-6-methyl-2-oxopyridin-1(2H)-yl)ethyl]benzoic acid |

---

```
Reaction no 109
```

Generated by the Chemistry Development Kit (http://github.com/cdk)

|  | A | B |
| --- | --- | --- |
| 0 | ADD (S)-2-[2-(3-bromophenyl)-[1,4]oxazepan-4-yl]-1-methyl-1H-[4,4′]bipyrimidinyl-6-one | ADD (S)-2-[2-(3-bromophenyl)-[1,4]oxazepan-4-yl]-1-methyl-1H-[4,4′]bipyrimidinyl-6-one |
| 1 | ADD zinc cyanide | ADD DMA |
| 2 | ADD tetrakis(triphenylphosphine) palladium(0) | ADD zinc cyanide at 25 °C |
| 3 | ADD DMA | ADD tetrakis(triphenylphosphine) palladium(0) at 25 °C |
| 4 | STIR for 3600 s at 100 °C | STIR for 28800 s at 100 °C |
| 5 | PURIFY | FILTER keep filtrate |
| 6 | YIELD (R)-2-[2-(3-cyanophenyl)-[1,4]oxazepan-4-yl]-1-methyl-1H-[4,4′]bipyrimidinyl-6-one | PARTITION with NaHCO3 and chloroform |
| 7 |  | COLLECTLAYER organic |
| 8 |  | WASH with water |
| 9 |  | DRYSOLUTION over sodium sulfate |
| 10 |  | CONCENTRATE |
| 11 |  | PURIFY |
| 12 |  | YIELD (R)-2-[2-(3-cyanophenyl)-[1,4]oxazepan-4-yl]-1-methyl-1H-[4,4′]bipyrimidinyl-6-one |

---

```
Reaction no 110
```

Generated by the Chemistry Development Kit (http://github.com/cdk)

|  | A | B |
| --- | --- | --- |
| 0 | ADD 20,20-Ethylenedioxy-3α-hydroxy-6β-methyl-5α-pregnan-11-one | ADD 20,20-Ethylenedioxy-3α-hydroxy-6β-methyl-5α-pregnan-11-one |
| 1 | ADD isopropanol | ADD isopropanol |
| 2 | ADD sodium borohydride | MAKESOLUTION with sodium borohydride and water |
| 3 | STIR for 3600 s at 25 °C | ADD SLN |
| 4 | ADD acetone | STIR for 86400 s at 25 °C |
| 5 | ADD water | ADD water |
| 6 | ADD HCl | COLLECTLAYER organic |
| 7 | CONCENTRATE | WASH with chloroform |
| 8 | ADD water | WASH with acetone |
| 9 | EXTRACT with ethyl acetate | WASH with HCl |
| 10 | COLLECTLAYER organic | WAIT for 3600 s |
| 11 | DRYSOLUTION over magnesium sulfate | ADD water |
| 12 | CONCENTRATE | EXTRACT with chloroform |
| 13 | PURIFY | RECRYSTALLIZE from methanol |
| 14 | YIELD 3α,11β-Dihydroxy-6β-methyl-5α-pregnan-20-one | YIELD 3α,11β-Dihydroxy-6β-methyl-5α-pregnan-20-one |

---

```
Reaction no 111
```

Generated by the Chemistry Development Kit (http://github.com/cdk)

|  | A | B |
| --- | --- | --- |
| 0 | ADD 2,4-difluorophenol | ADD 2,4-difluorophenol |
| 1 | ADD dichloromethane | ADD 2-bromo-1-(4-(methylthio)phenyl)ethanone |
| 2 | ADD K2CO3 | ADD dichloromethane |
| 3 | ADD tetrabutylammonium hydrogen sulfate | MAKESOLUTION with K2CO3 and tetrabutylammonium hydrogen sulfate and water |
| 4 | ADD 2-bromo-1-(4-(methylthio)phenyl)ethanone | ADD SLN |
| 5 | STIR for 86400 s at 25 °C | STIR for 86400 s at 25 °C |
| 6 | ADD water | ADD water |
| 7 | EXTRACT with dichloromethane | COLLECTLAYER organic |
| 8 | COLLECTLAYER organic | EXTRACT with dichloromethane |
| 9 | WASH with brine | DRYSOLUTION over Na2SO4 |
| 10 | DRYSOLUTION over Na2SO4 | CONCENTRATE |
| 11 | FILTER keep filtrate | WASH with ether |
| 12 | CONCENTRATE | YIELD 2-(2,4-Difluorophenoxy)-1-(4-methylsulfanyl phenyl)ethanone |
| 13 | PURIFY |  |
| 14 | YIELD 2-(2,4-Difluorophenoxy)-1-(4-methylsulfanyl phenyl)ethanone |  |

---

```
Reaction no 112
```

Generated by the Chemistry Development Kit (http://github.com/cdk)

|  | A | B |
| --- | --- | --- |
| 0 | ADD (2S,3R)-3-cyclopropyl-3-(3-(2′-fluoro-5′-methoxy-[1,1′-biphenyl]-4-yl)thiochroman-6-yl)-2-methylpropanoic acid | ADD (2S,3R)-3-cyclopropyl-3-(3-(2′-fluoro-5′-methoxy-[1,1′-biphenyl]-4-yl)thiochroman-6-yl)-2-methylpropanoic acid |
| 1 | ADD methanol | ADD methanol |
| 2 | MAKESOLUTION with Oxone and water | ADD water |
| 3 | ADD SLN at 25 °C over 3600 s | ADD Oxone |
| 4 | PARTITION with water and ethyl acetate | STIR for 86400 s at 25 °C |
| 5 | PHASESEPARATION | ADD water |
| 6 | COLLECTLAYER organic | EXTRACT with ethyl acetate |
| 7 | DRYSOLUTION over sodium sulfate | COLLECTLAYER organic |
| 8 | FILTER keep filtrate | WASH with sodium chloride |
| 9 | CONCENTRATE | DRYSOLUTION over magnesium sulfate |
| 10 | PURIFY | FILTER keep filtrate |
| 11 | YIELD (2S,3R)-3-Cyclopropyl-3-(3-(2′-fluoro-5′-methoxy-[1,1′-biphenyl]-4-yl)-1,1-dioxidothiochroman-6-yl)-2-methylpropanoic acid | CONCENTRATE |
| 12 |  | PURIFY |
| 13 |  | YIELD (2S,3R)-3-Cyclopropyl-3-(3-(2′-fluoro-5′-methoxy-[1,1′-biphenyl]-4-yl)-1,1-dioxidothiochroman-6-yl)-2-methylpropanoic acid |

---

```
Reaction no 113
```

Generated by the Chemistry Development Kit (http://github.com/cdk)

|  | A | B |
| --- | --- | --- |
| 0 | ADD 3-cyclopropyl-5-fluorobenzyl methanesulfonate | ADD 3-cyclopropyl-5-fluorobenzyl methanesulfonate |
| 1 | ADD acetonitrile | ADD acetonitrile |
| 2 | ADD NaCN at 25 °C | ADD NaCN |
| 3 | ADD 18-crown-6 at 25 °C | ADD 18-crown-6 |
| 4 | STIR for 28800 s at 60 °C | STIR for 86400 s at 25 °C |
| 5 | FILTER keep filtrate | FILTER keep filtrate |
| 6 | CONCENTRATE | CONCENTRATE |
| 7 | PURIFY | PURIFY |
| 8 | YIELD (3-cyclopropyl-5-fluorophenyl)acetonitrile | YIELD (3-cyclopropyl-5-fluorophenyl)acetonitrile |

---

```
Reaction no 114
```

Generated by the Chemistry Development Kit (http://github.com/cdk)

|  | A | B |
| --- | --- | --- |
| 0 | ADD 3-[(2S,5R)-2-[cyclopropyl(hydroxy)methyl]-1,3-oxathiolan-5-yl]-5-[[(4-methoxyphenyl)-diphenyl-methyl]amino]thiazolo[4, 5-d]pyrimidin-2-one | ADD 3-[(2S,5R)-2-[cyclopropyl(hydroxy)methyl]-1,3-oxathiolan-5-yl]-5-[[(4-methoxyphenyl)-diphenyl-methyl]amino]thiazolo[4, 5-d]pyrimidin-2-one |
| 1 | ADD dichloromethane | ADD dichloromethane |
| 2 | ADD triethylamine at 0 °C | ADD acetic anhydride |
| 3 | ADD DMAP at 0 °C | ADD DMAP |
| 4 | ADD acetic anhydride at 0 °C | WAIT for 3600 s |
| 5 | STIR for 86400 s at 25 °C | CONCENTRATE |
| 6 | QUENCH with water | PURIFY |
| 7 | EXTRACT with dichloromethane | YIELD [cyclopropyl-[(2S,5R)-5-[5-[[(4-methoxyphenyl)-diphenyl-methyl]amino]-2-oxo-thiazolo[4,5-d]pyrimidin-3-yl]-1,3-oxathiolan-2-yl]methyl] acetate |
| 8 | COLLECTLAYER organic |  |
| 9 | DRYSOLUTION |  |
| 10 | CONCENTRATE |  |
| 11 | YIELD [cyclopropyl-[(2S,5R)-5-[5-[[(4-methoxyphenyl)-diphenyl-methyl]amino]-2-oxo-thiazolo[4,5-d]pyrimidin-3-yl]-1,3-oxathiolan-2-yl]methyl] acetate |  |

---

```
Reaction no 115
```

Generated by the Chemistry Development Kit (http://github.com/cdk)

|  | A | B |
| --- | --- | --- |
| 0 | ADD 4-[5-(2-chlorophenyl)-4-(4-{[2-(trimethylsilyl)-ethoxy]methyl}-4H-1,2,4-triazol-3-yl)-1,3-oxazol-2-yl]-5-methylpyridin-2-amine | ADD 4-[5-(2-chlorophenyl)-4-(4-{[2-(trimethylsilyl)-ethoxy]methyl}-4H-1,2,4-triazol-3-yl)-1,3-oxazol-2-yl]-5-methylpyridin-2-amine |
| 1 | ADD dichloromethane | ADD triethylamine |
| 2 | ADD cyclopropanecarbonyl chloride | ADD dichloromethane |
| 3 | STIR for 3600 s at 25 °C | STIR for 600 s at 0 °C |
| 4 | ADD methanol | ADD cyclopropanecarbonyl chloride |
| 5 | CONCENTRATE | STIR for 3600 s at 0 °C |
| 6 | PURIFY | ADD methanol |
| 7 | YIELD N-{4-[5-(2-chlorophenyl)-4-(4H-1,2,4-triazol-3-yl)-1,3-oxazol-2-yl]-5-methylpyridin-2-yl}cyclopropanecarboxamide | STIR for 600 s at 25 °C |
| 8 |  | CONCENTRATE |
| 9 |  | ADD methanol |
| 10 |  | ADD NaHCO3 |
| 11 |  | SETTEMPERATURE 25 °C |
| 12 |  | ADD NaOH |
| 13 |  | STIR for 28800 s at 25 °C |
| 14 |  | CONCENTRATE |
| 15 |  | ADD ethyl acetate |
| 16 |  | STIR for 3600 s at 25 °C |
| 17 |  | FILTER keep filtrate |
| 18 |  | CONCENTRATE |
| 19 |  | ADD dichloromethane |
| 20 |  | ADD TFA |
| 21 |  | STIR for 86400 s at 25 °C |
| 22 |  | CONCENTRATE |
| 23 |  | PURIFY |
| 24 |  | YIELD N-{4-[5-(2-chlorophenyl)-4-(4H-1,2,4-triazol-3-yl)-1,3-oxazol-2-yl]-5-methylpyridin-2-yl}cyclopropanecarboxamide |

---

```
Reaction no 116
```

Generated by the Chemistry Development Kit (http://github.com/cdk)

|  | A | B |
| --- | --- | --- |
| 0 | ADD 7-(Quinolin-3-yl)-2,3-dihydrobenzo[f][1,4]oxazepine hydrochloride | ADD N-methyl-terephthalamic acid |
| 1 | ADD N-methyl-terephthalamic acid | ADD DIPEA |
| 2 | ADD HATU | ADD HATU |
| 3 | ADD DMF | ADD DMF |
| 4 | ADD DIPEA | MAKESOLUTION with 7-(Quinolin-3-yl)-2,3-dihydrobenzo[f][1,4]oxazepine hydrochloride and DIPEA and DMF |
| 5 | STIR for 3600 s at 25 °C | ADD SLN |
| 6 | ADD NaHCO3 | STIR for 600 s at 60 °C |
| 7 | EXTRACT with ethyl acetate | ADD NaHCO3 |
| 8 | COLLECTLAYER organic | FILTER keep precipitate |
| 9 | WASH with brine | WASH with water |
| 10 | DRYSOLUTION over Na2SO4 | PURIFY |
| 11 | FILTER keep filtrate | YIELD N-methyl-4-[(7-quinolin-3-yl-2,3-dihydro-1,4-benzoxazepin-4(5H)-yl)carbonyl]benzamide |
| 12 | CONCENTRATE |  |
| 13 | PURIFY |  |
| 14 | YIELD N-methyl-4-[(7-quinolin-3-yl-2,3-dihydro-1,4-benzoxazepin-4(5H)-yl)carbonyl]benzamide |  |

---

```
Reaction no 117
```

Generated by the Chemistry Development Kit (http://github.com/cdk)

|  | A | B |
| --- | --- | --- |
| 0 | ADD 4-[5-[4-(aminoiminomethyl)phenoxy]pentyloxy]-2-hydroxy-N,N-bis(1-methylethyl)benzamide monohydrochloride | ADD 4-[5-[4-(aminoiminomethyl)phenoxy]pentyloxy]-2-hydroxy-N,N-bis(1-methylethyl)benzamide monohydrochloride |
| 1 | ADD dichloromethane | ADD dichloromethane |
| 2 | ADD phenyl acetate | ADD triethylamine |
| 3 | ADD triethylamine | ADD phenyl acetate |
| 4 | STIR for 86400 s | STIR for 86400 s at 25 °C |
| 5 | CONCENTRATE | CONCENTRATE |
| 6 | PURIFY | ADD ethyl acetate |
| 7 | YIELD 4-[5-[4-(acetyliminoaminomethyl)phenoxy]pentyloxy]-2-hydroxy-N,N-bis(1-methylethyl)benzamide | WASH with water |
| 8 |  | WASH with brine |
| 9 |  | DRYSOLUTION over magnesium sulfate |
| 10 |  | CONCENTRATE |
| 11 |  | PURIFY |
| 12 |  | YIELD 4-[5-[4-(acetyliminoaminomethyl)phenoxy]pentyloxy]-2-hydroxy-N,N-bis(1-methylethyl)benzamide |

---

```
Reaction no 118
```

Generated by the Chemistry Development Kit (http://github.com/cdk)

|  | A | B |
| --- | --- | --- |
| 0 | ADD N-{4-[6-Bromo-3-(5-methoxypyridin-2-yl)-1H-pyrrolo[3,2-b]pyridin-2-yl]pyridin-2-yl}acetamide | ADD N-{4-[6-Bromo-3-(5-methoxypyridin-2-yl)-1H-pyrrolo[3,2-b]pyridin-2-yl]pyridin-2-yl}acetamide |
| 1 | ADD di-tert-butyl dicarbonate | ADD DMAP |
| 2 | ADD triethylamine | ADD THF |
| 3 | ADD DMAP | ADD di-tert-butyl dicarbonate |
| 4 | ADD THF | STIR for 86400 s at 25 °C |
| 5 | STIR for 86400 s at 25 °C | ADD water |
| 6 | ADD water | EXTRACT with ethyl acetate |
| 7 | EXTRACT with ethyl acetate | COLLECTLAYER organic |
| 8 | COLLECTLAYER organic | DRYSOLUTION over sodium sulfate |
| 9 | DRYSOLUTION over Na2SO4 | FILTER keep filtrate |
| 10 | FILTER keep filtrate | CONCENTRATE |
| 11 | CONCENTRATE | YIELD tert-butyl 2-(2-acetamidopyridin-4-yl)-3-(5-methoxypyridin-2-yl)-6-(pyrrolidin-1-yl)-1H-pyrrolo[3,2-b]pyridine-1-carboxylate |
| 12 | PURIFY |  |
| 13 | YIELD tert-butyl 2-(2-acetamidopyridin-4-yl)-3-(5-methoxypyridin-2-yl)-6-(pyrrolidin-1-yl)-1H-pyrrolo[3,2-b]pyridine-1-carboxylate |  |

---

```
Reaction no 119
```

Generated by the Chemistry Development Kit (http://github.com/cdk)

|  | A | B |
| --- | --- | --- |
| 0 | ADD 4-(4-(2-tert-butylphenyl)piperazin-1-yl)-4-oxobutanoic acid | ADD 4-(4-(2-tert-butylphenyl)piperazin-1-yl)-4-oxobutanoic acid |
| 1 | ADD dichloromethane | ADD EDCI |
| 2 | ADD HOBt | ADD HOBt |
| 3 | ADD EDCI | ADD DIPEA |
| 4 | STIR for 3600 s at 25 °C | ADD dichloromethane |
| 5 | ADD DIPEA | ADD methanesulfonamide at 25 °C |
| 6 | ADD methanesulfonamide | WAIT for 86400 s |
| 7 | STIR for 86400 s at 25 °C | ADD ethyl acetate |
| 8 | ADD ethyl acetate | WASH with HCl |
| 9 | WASH with water | WASH with water |
| 10 | COLLECTLAYER organic | DRYSOLUTION over Na2SO4 |
| 11 | DRYSOLUTION over MgSO4 | FILTER keep filtrate |
| 12 | CONCENTRATE | CONCENTRATE |
| 13 | PURIFY | PURIFY |
| 14 | YIELD 4-(4-(2-tert-butylphenyl)piperazin-1-yl)-N-(methylsulfonyl)-4-oxobutanamide | YIELD 4-(4-(2-tert-butylphenyl)piperazin-1-yl)-N-(methylsulfonyl)-4-oxobutanamide |

---

```
Reaction no 120
```

Generated by the Chemistry Development Kit (http://github.com/cdk)

|  | A | B |
| --- | --- | --- |
| 0 | ADD ethyl 7-((6-amino-9H-purin-9-yl)methyl)-6-chloro-1H-indole-2-carboxylate | ADD ethyl 7-((6-amino-9H-purin-9-yl)methyl)-6-chloro-1H-indole-2-carboxylate |
| 1 | ADD THF | ADD THF |
| 2 | ADD LiAlH4 at 0 °C | ADD LiAlH4 at 0 °C |
| 3 | ADD THF at 0 °C | STIR for 3600 s at 0 °C |
| 4 | STIR for 28800 s at 25 °C | QUENCH with water |
| 5 | QUENCH with water | QUENCH with sodium hydroxide |
| 6 | ADD ethyl acetate | QUENCH with water |
| 7 | FILTER keep filtrate | FILTER keep filtrate |
| 8 | EXTRACT with ethyl acetate | CONCENTRATE |
| 9 | COLLECTLAYER organic | YIELD (7-((6-amino-9H-purin-9-yl)methyl)-6-chloro-1H-indol-2-yl)methanol |
| 10 | WASH with brine |  |
| 11 | DRYSOLUTION over sodium sulfate |  |
| 12 | FILTER keep filtrate |  |
| 13 | CONCENTRATE |  |
| 14 | TRITURATE with petroleum ether / ethyl acetate |  |
| 15 | YIELD (7-((6-amino-9H-purin-9-yl)methyl)-6-chloro-1H-indol-2-yl)methanol |  |

---

```
Reaction no 121
```

Generated by the Chemistry Development Kit (http://github.com/cdk)

|  | A | B |
| --- | --- | --- |
| 0 | ADD 4-[(E)-(4-bromophenyl)(ethoxyimino)methyl]-1-(4-methyl-4-piperidinyl)piperidine | ADD 4-[(E)-(4-bromophenyl)(ethoxyimino)methyl]-1-(4-methyl-4-piperidinyl)piperidine |
| 1 | ADD 2-methyl-1,8-naphthyridine-3-carboxylic acid | ADD 2-methyl-1,8-naphthyridine-3-carboxylic acid |
| 2 | ADD triethylamine | ADD triethylamine |
| 3 | ADD DMF | ADD DMF |
| 4 | ADD HATU at 25 °C | ADD HATU |
| 5 | WAIT for 86400 s | WAIT for 86400 s |
| 6 | ADD ice water | ADD ice water |
| 7 | FILTER keep precipitate | EXTRACT with dichloromethane |
| 8 | ADD dichloromethane | COLLECTLAYER organic |
| 9 | DRYSOLUTION over Na2SO4 | DRYSOLUTION over Na2SO4 |
| 10 | PURIFY | CONCENTRATE |
| 11 | YIELD 3-[[4-[4-[(E)-(4-Bromophenyl)(ethoxyimino)methyl]-1-piperidinyl]-4-methyl-1-piperidinyl]carbonyl]-2-methyl-1,8-naphthyridine | PURIFY |
| 12 |  | YIELD 3-[[4-[4-[(E)-(4-Bromophenyl)(ethoxyimino)methyl]-1-piperidinyl]-4-methyl-1-piperidinyl]carbonyl]-2-methyl-1,8-naphthyridine |

---

```
Reaction no 122
```

Generated by the Chemistry Development Kit (http://github.com/cdk)

|  | A | B |
| --- | --- | --- |
| 0 | ADD (methoxymethyl)triphenylphosphonium chloride | ADD (methoxymethyl)triphenylphosphonium chloride |
| 1 | ADD THF | ADD THF |
| 2 | ADD potassium t-butoxide at 0 °C | MAKESOLUTION with potassium t-butoxide and THF |
| 3 | STIR for 3600 s at 0 °C | ADD SLN at 0 °C |
| 4 | MAKESOLUTION with (±)-4-((3R\*,4aR\*,9bS\*)-7-ethoxy-6-fluoro-1,2,3,4,4a,9b-hexahydrodibenzofuran-3-yl)cyclohexanone and THF | WAIT for 3600 s at 0 °C |
| 5 | ADD SLN dropwise at 0 °C | MAKESOLUTION with (±)-4-((3R\*,4aR\*,9bS\*)-7-ethoxy-6-fluoro-1,2,3,4,4a,9b-hexahydrodibenzofuran-3-yl)cyclohexanone and THF |
| 6 | STIR for 3600 s at 25 °C | ADD SLN |
| 7 | ADD water | STIR for 86400 s at 25 °C |
| 8 | PH with HCl to pH acidic | ADD water at 0 °C |
| 9 | EXTRACT with ethyl acetate | PH with HCl to pH acidic |
| 10 | WASH with water | EXTRACT with MTBE |
| 11 | DRYSOLUTION over magnesium sulfate | COLLECTLAYER organic |
| 12 | CONCENTRATE | WASH with sodium chloride |
| 13 | PURIFY | DRYSOLUTION over sodium sulfate |
| 14 | YIELD (±)-(3R\*,4aR\*,9bS\*)-7-ethoxy-6-fluoro-3-(4-methoxymethylene-cyclohexyl)-1,2,3,4,4a,9b-hexahydrodibenzofuran | CONCENTRATE |
| 15 |  | PURIFY |
| 16 |  | YIELD (±)-(3R\*,4aR\*,9bS\*)-7-ethoxy-6-fluoro-3-(4-methoxymethylene-cyclohexyl)-1,2,3,4,4a,9b-hexahydrodibenzofuran |

---

```
Reaction no 123
```

Generated by the Chemistry Development Kit (http://github.com/cdk)

|  | A | B |
| --- | --- | --- |
| 0 | ADD 6-[5H-imidazo[4,3-a]isoindol-5-yl]-5H,6H,7H-cyclopenta[c]pyridin-7-one | ADD 6-[5H-imidazo[4,3-a]isoindol-5-yl]-5H,6H,7H-cyclopenta[c]pyridin-7-one |
| 1 | ADD THF | ADD THF |
| 2 | MAKESOLUTION with L-Selectride and THF | MAKESOLUTION with L-Selectride and THF |
| 3 | ADD SLN at -70 °C | ADD SLN at -70 °C |
| 4 | STIR for 3600 s at -70 °C | STIR for 3600 s at -70 °C |
| 5 | SETTEMPERATURE 25 °C | QUENCH with methanol |
| 6 | QUENCH with water | ADD water |
| 7 | CONCENTRATE | FILTER keep precipitate |
| 8 | PURIFY | WASH with dichloromethane |
| 9 | YIELD 6-(5H-imidazo[5,1-a]isoindol-5-yl)-6,7-dihydro-5H-cyclopenta[c]pyridin-7-ol | EXTRACT with dichloromethane |
| 10 |  | COLLECTLAYER organic |
| 11 |  | DRYSOLUTION over sodium sulfate |
| 12 |  | CONCENTRATE |
| 13 |  | PURIFY |
| 14 |  | YIELD 6-(5H-imidazo[5,1-a]isoindol-5-yl)-6,7-dihydro-5H-cyclopenta[c]pyridin-7-ol |

---

```
Reaction no 124
```

Generated by the Chemistry Development Kit (http://github.com/cdk)

|  | A | B |
| --- | --- | --- |
| 0 | ADD hydroxylamine hydrochloride | ADD CC(=O)CC(=O)N(Cc1cc(C(F)(F)F)cc(C(F)(F)F)c1)C1(Cl)CCCN(C(=O)OC(C)C)c2ccccc21 |
| 1 | ADD sodium acetate | ADD methanol |
| 2 | MAKESOLUTION with CC(=O)CC(=O)N(Cc1cc(C(F)(F)F)cc(C(F)(F)F)c1)C1(Cl)CCCN(C(=O)OC(C)C)c2ccccc21 and methanol | ADD sodium acetate |
| 3 | ADD SLN | ADD hydroxylamine hydrochloride |
| 4 | REFLUX for 86400 s | REFLUX for 3600 s |
| 5 | SETTEMPERATURE 25 °C | SETTEMPERATURE 25 °C |
| 6 | CONCENTRATE | CONCENTRATE |
| 7 | ADD ethyl acetate | ADD ethyl acetate |
| 8 | ADD brine | WASH with water |
| 9 | PHASESEPARATION | WASH with brine |
| 10 | COLLECTLAYER organic | DRYSOLUTION over Na2SO4 |
| 11 | DRYSOLUTION over magnesium sulfate | FILTER keep filtrate |
| 12 | FILTER keep filtrate | CONCENTRATE |
| 13 | CONCENTRATE | PURIFY |
| 14 | PURIFY | YIELD (+/−)-isopropyl 5-[(3,5-bistrifluoromethyl-benzyl)-(5-methyl-isoxazol-5-yl)-amino]-8-chloro-2,3,4,5-tetrahydrobenzo[b]azepine-1-carboxylate |
| 15 | YIELD (+/−)-isopropyl 5-[(3,5-bistrifluoromethyl-benzyl)-(5-methyl-isoxazol-5-yl)-amino]-8-chloro-2,3,4,5-tetrahydrobenzo[b]azepine-1-carboxylate |  |

---

```
Reaction no 125
```

Generated by the Chemistry Development Kit (http://github.com/cdk)

|  | A | B |
| --- | --- | --- |
| 0 | ADD 8-ethyltricyclo[5.2.1.02,6]decan-8-ol | ADD benzene |
| 1 | ADD benzene | ADD 8-ethyltricyclo[5.2.1.02,6]decan-8-ol |
| 2 | ADD p-toluenesulfonic acid monohydrate | ADD p-toluenesulfonic acid monohydrate |
| 3 | REFLUX for 28800 s with Dean-Stark apparatus | REFLUX for 28800 s |
| 4 | SETTEMPERATURE 25 °C | SETTEMPERATURE 25 °C |
| 5 | WASH with sodium bicarbonate | CONCENTRATE |
| 6 | WASH with water | PURIFY |
| 7 | DRYSOLUTION over Na2SO4 | YIELD 8-ethylidenetricyclo[5.2.1.02,6]decane |
| 8 | CONCENTRATE |  |
| 9 | PURIFY |  |
| 10 | YIELD 8-ethylidenetricyclo[5.2.1.02,6]decane |  |

---

```
Reaction no 126
```

Generated by the Chemistry Development Kit (http://github.com/cdk)

|  | A | B |
| --- | --- | --- |
| 0 | ADD tert-butyl ethyl 2-[(tert-butyl)oxycarbonyl]-2-(2-methylpropyl)butane-1,4-dioate | ADD tert-butyl ethyl 2-[(tert-butyl)oxycarbonyl]-2-(2-methylpropyl)butane-1,4-dioate |
| 1 | ADD TFA | ADD TFA |
| 2 | STIR for 3600 s at 25 °C | STIR for 3600 s |
| 3 | CONCENTRATE | CONCENTRATE |
| 4 | YIELD 2-[(ethoxycarbonyl)methyl]-2-(2-methylpropyl)propanedioic acid | YIELD 2-[(ethoxycarbonyl)methyl]-2-(2-methylpropyl)propanedioic acid |

---

```
Reaction no 127
```

Generated by the Chemistry Development Kit (http://github.com/cdk)

|  | A | B |
| --- | --- | --- |
| 0 | ADD 5-Aminomethyl-3-(7-oxo-6,7,8,9-tetrahydro-5H-benzocyclohepten-2-yl)-oxazolidin-2-one | ADD 5-Aminomethyl-3-(7-oxo-6,7,8,9-tetrahydro-5H-benzocyclohepten-2-yl)-oxazolidin-2-one |
| 1 | ADD acetic anhydride | ADD pyridine |
| 2 | ADD pyridine | SETTEMPERATURE 0 °C |
| 3 | STIR for 86400 s at 25 °C | ADD acetic anhydride |
| 4 | CONCENTRATE | STIR for 3600 s at 0 °C |
| 5 | PURIFY | STIR for 86400 s at 25 °C |
| 6 | YIELD N-[2-Oxo-3-(7-oxo-6,7,8,9-tetrahydro-5H-benzocyclohepten-2-yl)-oxazolidin-5-ylmethyl]-acetamide | CONCENTRATE |
| 7 |  | PURIFY |
| 8 |  | YIELD N-[2-Oxo-3-(7-oxo-6,7,8,9-tetrahydro-5H-benzocyclohepten-2-yl)-oxazolidin-5-ylmethyl]-acetamide |

---

```
Reaction no 128
```

Generated by the Chemistry Development Kit (http://github.com/cdk)

|  | A | B |
| --- | --- | --- |
| 0 | ADD 1-tert-butyl 2-methyl 6-(2-(3-chloro-4-fluorophenylamino)-4-(3-methoxypropylamino)pyrimidin-5-yl)-1H-indole-1,2-dicarboxylate | ADD 1-tert-butyl 2-methyl 6-(2-(3-chloro-4-fluorophenylamino)-4-(3-methoxypropylamino)pyrimidin-5-yl)-1H-indole-1,2-dicarboxylate |
| 1 | ADD dichloromethane | ADD dichloromethane |
| 2 | ADD TFA | ADD TFA |
| 3 | STIR for 3600 s at 25 °C | STIR for 3600 s at 25 °C |
| 4 | CONCENTRATE | CONCENTRATE |
| 5 | PURIFY | WASH with ether / hexanes |
| 6 | YIELD methyl 6-(2-(3-chloro-4-fluorophenylamino)-4-(3-methoxypropylamino)pyrimidin-5-yl)-1H-indole-2-carboxylate | YIELD methyl 6-(2-(3-chloro-4-fluorophenylamino)-4-(3-methoxypropylamino)pyrimidin-5-yl)-1H-indole-2-carboxylate |

---

```
Reaction no 129
```

Generated by the Chemistry Development Kit (http://github.com/cdk)

|  | A | B |
| --- | --- | --- |
| 0 | ADD Methyl ‌(S) -4- ‌(5-methylthiazol-2-yl) -3- ‌(tetrahydropyran-2-yl)oxym ethyl-3,4-dihydro-2H-benzo[1,4]oxazine-8-carboxylate | ADD Methyl ‌(S) -4- ‌(5-methylthiazol-2-yl) -3- ‌(tetrahydropyran-2-yl)oxym ethyl-3,4-dihydro-2H-benzo[1,4]oxazine-8-carboxylate |
| 1 | ADD THF | ADD THF |
| 2 | ADD methanol | ADD methanol |
| 3 | ADD NaOH | ADD NaOH |
| 4 | STIR for 3600 s at 100 °C | STIR for 86400 s at 25 °C |
| 5 | PH with HCl to pH neutral | PH with HCl to pH acidic |
| 6 | EXTRACT with ethyl acetate | FILTER keep precipitate |
| 7 | COLLECTLAYER organic | WASH with water |
| 8 | WASH with sodium chloride | DRYSOLID under vacuum |
| 9 | DRYSOLUTION over sodium sulfate | YIELD (S)-4-(5-methylthiazol-2-yl)-3-(tetrahydropyran-2-yl)oxymethyl-3,4-dihydro-2H-benzo[1,4]oxazine-8-carboxylic acid |
| 10 | CONCENTRATE |  |
| 11 | YIELD (S)-4-(5-methylthiazol-2-yl)-3-(tetrahydropyran-2-yl)oxymethyl-3,4-dihydro-2H-benzo[1,4]oxazine-8-carboxylic acid |  |

---

```
Reaction no 130
```

Generated by the Chemistry Development Kit (http://github.com/cdk)

|  | A | B |
| --- | --- | --- |
| 0 | ADD 2-[[4-[4-(trifluoromethoxy)phenoxy]phenoxy]]propanoic acid | ADD 2-[[4-[4-(trifluoromethoxy)phenoxy]phenoxy]]propanoic acid |
| 1 | ADD thionyl chloride | ADD DMF |
| 2 | ADD DMF | ADD thionyl chloride |
| 3 | REFLUX for 3600 s | CONCENTRATE |
| 4 | CONCENTRATE | EXTRACT with dichloromethane |
| 5 | YIELD 2-[[4-[4-(trifluoromethoxy)phenoxy]phenoxy]]propanoyl chloride | WASH with ice water |
| 6 |  | DRYSOLUTION |
| 7 |  | CONCENTRATE |
| 8 |  | YIELD 2-[[4-[4-(trifluoromethoxy)phenoxy]phenoxy]]propanoyl chloride |

---

```
Reaction no 131
```

Generated by the Chemistry Development Kit (http://github.com/cdk)

|  | A | B |
| --- | --- | --- |
| 0 | ADD 2-Bromo-1-(4-bromo-2-fluorophenyl)ethanone | ADD 2-Bromo-1-(4-bromo-2-fluorophenyl)ethanone |
| 1 | ADD (2S,5S)-5-(methoxycarbonylamino)-4-oxo-1,2,4,5,6,7-hexahydroazepino[3,2,1-hi]indole-2-carboxylic acid | ADD (2S,5S)-5-(methoxycarbonylamino)-4-oxo-1,2,4,5,6,7-hexahydroazepino[3,2,1-hi]indole-2-carboxylic acid |
| 2 | ADD acetonitrile | ADD acetonitrile |
| 3 | ADD DIPEA | ADD DIPEA |
| 4 | STIR for 28800 s at 25 °C | STIR for 28800 s at 25 °C |
| 5 | CONCENTRATE | CONCENTRATE |
| 6 | PARTITION with dichloromethane and water | PARTITION with dichloromethane and water |
| 7 | COLLECTLAYER aqueous | COLLECTLAYER organic |
| 8 | EXTRACT with dichloromethane | CONCENTRATE |
| 9 | COLLECTLAYER organic | PURIFY |
| 10 | CONCENTRATE | YIELD (2S,5S)-5-Methoxycarbonylamino-4-oxo-1,2,4,5,6,7-hexahydro-azepino[3,2,1-hi]indole-2-carboxylic acid 2-(4-bromo-phenyl)-2-oxo-ethyl ester |
| 11 | PURIFY |  |
| 12 | YIELD (2S,5S)-5-Methoxycarbonylamino-4-oxo-1,2,4,5,6,7-hexahydro-azepino[3,2,1-hi]indole-2-carboxylic acid 2-(4-bromo-phenyl)-2-oxo-ethyl ester |  |

---

```
Reaction no 132
```

Generated by the Chemistry Development Kit (http://github.com/cdk)

|  | A | B |
| --- | --- | --- |
| 0 | ADD methyl 4-(bromomethyl)benzoate | ADD methyl 4-(bromomethyl)benzoate |
| 1 | ADD DMF | ADD DMF |
| 2 | ADD phenol | ADD phenol |
| 3 | ADD Cs2CO3 | ADD Cs2CO3 |
| 4 | STIR for 86400 s at 25 °C | STIR for 86400 s at 25 °C |
| 5 | ADD water | ADD water |
| 6 | WAIT for 600 s | EXTRACT with ethyl acetate |
| 7 | FILTER keep precipitate | COLLECTLAYER organic |
| 8 | DRYSOLID | WASH with brine |
| 9 | DRYSOLID under vacuum | DRYSOLUTION over Na2SO4 |
| 10 | YIELD methyl 4-phenoxymethylbenzoate | CONCENTRATE |
| 11 |  | PURIFY |
| 12 |  | YIELD methyl 4-phenoxymethylbenzoate |

---

```
Reaction no 133
```

Generated by the Chemistry Development Kit (http://github.com/cdk)

|  | A | B |
| --- | --- | --- |
| 0 | ADD 1-Amino-2-cyclopentyl-N-methyl-1H-imidazole-5-carboxamide | ADD 1-Amino-2-cyclopentyl-N-methyl-1H-imidazole-5-carboxamide |
| 1 | ADD chloroacetyl chloride | ADD chloroacetyl chloride |
| 2 | STIR for 28800 s at 100 °C | STIR for 28800 s at 100 °C |
| 3 | SETTEMPERATURE 25 °C | CONCENTRATE |
| 4 | QUENCH with NaHCO3 aq. | PURIFY |
| 5 | EXTRACT with dichloromethane | YIELD 2-(Chloromethyl)-7-cyclopentyl-3-methylimidazo[5,1-f][1,2,4]triazin-4(3H)-one |
| 6 | COLLECTLAYER organic |  |
| 7 | DRYSOLUTION |  |
| 8 | CONCENTRATE |  |
| 9 | PURIFY |  |
| 10 | YIELD 2-(Chloromethyl)-7-cyclopentyl-3-methylimidazo[5,1-f][1,2,4]triazin-4(3H)-one |  |

---

```
Reaction no 134
```

Generated by the Chemistry Development Kit (http://github.com/cdk)

|  | A | B |
| --- | --- | --- |
| 0 | ADD 2-(3-Methoxy-phenyl)-4,4-dimethyl-cyclohex-2-enone | ADD 2-(3-Methoxy-phenyl)-4,4-dimethyl-cyclohex-2-enone |
| 1 | ADD ethanol | ADD ethanol |
| 2 | ADD nitrogen | ADD nitrogen |
| 3 | ADD Pd/C | ADD Pd/C |
| 4 | STIR for 86400 s | STIR for 86400 s at 25 °C |
| 5 | FILTER keep filtrate | FILTER keep filtrate |
| 6 | CONCENTRATE | CONCENTRATE |
| 7 | YIELD 2-(3-methoxy-phenyl)-4,4-dimethyl-cyclohexanone | YIELD 2-(3-methoxy-phenyl)-4,4-dimethyl-cyclohexanone |

---

```
Reaction no 135
```

Generated by the Chemistry Development Kit (http://github.com/cdk)

|  | A | B |
| --- | --- | --- |
| 0 | ADD bromoacetic anhydride | ADD bromoacetic anhydride |
| 1 | ADD acetonitrile | ADD acetonitrile |
| 2 | MAKESOLUTION with tert-butyl 2-aminoethylcarbamate and triethylamine and THF | MAKESOLUTION with ninhydrin and THF |
| 3 | ADD SLN at 25 °C | ADD SLN at 0 °C |
| 4 | PH with ninhydrin to pH neutral | STIR for 3600 s at 0 °C |
| 5 | CONCENTRATE | ADD tert-butyl 2-aminoethylcarbamate |
| 6 | ADD ethyl acetate | STIR for 3600 s at 25 °C |
| 7 | WASH with sodium bicarbonate | CONCENTRATE |
| 8 | WASH with sulfuric acid | PURIFY |
| 9 | WASH with brine | YIELD N-bromoacetyl-N′-Boc-ethylenediamine |
| 10 | DRYSOLUTION over sodium sulfate |  |
| 11 | CONCENTRATE |  |
| 12 | YIELD N-bromoacetyl-N′-Boc-ethylenediamine |  |

---

```
Reaction no 136
```

Generated by the Chemistry Development Kit (http://github.com/cdk)

|  | A | B |
| --- | --- | --- |
| 0 | ADD (rac.)-(1R\*,5S\*)-7-{4-[3-(2-chloro-3,6-difluoro-phenoxy)-propyl]-phenyl}-3,9-diaza-bicyclo[3.3.1]non-6-ene-3,6,9-tricarboxylic acid 3,9-di-tert-butyl ester | ADD (rac.)-(1R\*,5S\*)-7-{4-[3-(2-chloro-3,6-difluoro-phenoxy)-propyl]-phenyl}-3,9-diaza-bicyclo[3.3.1]non-6-ene-3,6,9-tricarboxylic acid 3,9-di-tert-butyl ester |
| 1 | ADD dichloromethane | ADD 2-amino-4-tert-butylphenol |
| 2 | ADD 1-(3-dimethylaminopropyl)-3-ethylcarbodiimide hydrochloride | ADD DIPEA |
| 3 | ADD HOBt | ADD DMAP |
| 4 | ADD DIPEA | ADD HOBt |
| 5 | ADD DMAP | ADD 1-(3-dimethylaminopropyl)-3-ethylcarbodiimide hydrochloride |
| 6 | STIR for 86400 s at 25 °C | ADD dichloromethane |
| 7 | ADD dichloromethane | STIR for 604800 s at 25 °C |
| 8 | WASH with water | WASH with HCl |
| 9 | WASH with ammonium chloride | WASH with NaHCO3 |
| 10 | WASH with sodium chloride | DRYSOLUTION over MgSO4 |
| 11 | COLLECTLAYER organic | FILTER keep filtrate |
| 12 | DRYSOLUTION over sodium sulfate | CONCENTRATE |
| 13 | FILTER keep filtrate | PURIFY |
| 14 | CONCENTRATE | YIELD (rac.)-(1R\*,5S\*)-7-{4-[3-(2-Chloro-3,6-difluorophenoxy)propyl]phenyl}-6-{cyclopropyl-[2-(2-methoxyethoxy)-3-methylpyridin-4-ylmethyl]carbamoyl}-3,9-diazabicyclo[3.3.1]non-6-ene-3,9-dicarboxylic acid di-tert-butyl ester |
| 15 | PURIFY |  |
| 16 | YIELD (rac.)-(1R\*,5S\*)-7-{4-[3-(2-Chloro-3,6-difluorophenoxy)propyl]phenyl}-6-{cyclopropyl-[2-(2-methoxyethoxy)-3-methylpyridin-4-ylmethyl]carbamoyl}-3,9-diazabicyclo[3.3.1]non-6-ene-3,9-dicarboxylic acid di-tert-butyl ester |  |

---

```
Reaction no 137
```

Generated by the Chemistry Development Kit (http://github.com/cdk)

|  | A | B |
| --- | --- | --- |
| 0 | ADD 2,3-Dichloro-5-methylfuro[3,2-b]pyridine | ADD 2,3-Dichloro-5-methylfuro[3,2-b]pyridine |
| 1 | ADD THF | ADD THF |
| 2 | ADD tert-butyllithium | ADD tert-butyllithium at -70 °C |
| 3 | WAIT for 3600 s | STIR for 3600 s at -70 °C |
| 4 | QUENCH with methanol dropwise at -70 °C | QUENCH with ammonium chloride |
| 5 | SETTEMPERATURE 25 °C | EXTRACT with ethyl acetate |
| 6 | EXTRACT with ethyl acetate | COLLECTLAYER organic |
| 7 | CONCENTRATE | DRYSOLUTION over Na2SO4 |
| 8 | PURIFY | FILTER keep filtrate |
| 9 | YIELD 3-Chloro-5-methylfuro[3,2-b]pyridine | CONCENTRATE |
| 10 |  | PURIFY |
| 11 |  | YIELD 3-Chloro-5-methylfuro[3,2-b]pyridine |

---

```
Reaction no 138
```

Generated by the Chemistry Development Kit (http://github.com/cdk)

|  | A | B |
| --- | --- | --- |
| 0 | ADD 2-(4-fluorobenzyloxy)-6,7-dihydro-5H-benzocycloheptene-8-carboxylate | ADD 2-(4-fluorobenzyloxy)-6,7-dihydro-5H-benzocycloheptene-8-carboxylate |
| 1 | ADD methanol | ADD methanol |
| 2 | ADD THF | ADD THF |
| 3 | ADD NaOH | ADD NaOH |
| 4 | STIR for 3600 s at 60 °C | STIR for 86400 s at 25 °C |
| 5 | ADD HCl at 0 °C | ADD HCl |
| 6 | CONCENTRATE | CONCENTRATE |
| 7 | ADD water | ADD water |
| 8 | FILTER keep precipitate | FILTER keep precipitate |
| 9 | WASH with water | WASH with water |
| 10 | DRYSOLID under vacuum | DRYSOLID under vacuum |
| 11 | YIELD 2-(4-fluorobenzyloxy)-6,7-dihydro-5H-benzocycloheptene-8-carboxylic acid | YIELD 2-(4-fluorobenzyloxy)-6,7-dihydro-5H-benzocycloheptene-8-carboxylic acid |

---

```
Reaction no 139
```

Generated by the Chemistry Development Kit (http://github.com/cdk)

|  | A | B |
| --- | --- | --- |
| 0 | ADD Ethyl 3-amino-2-methyl-3-thioxopropanoate | ADD Ethyl 3-amino-2-methyl-3-thioxopropanoate |
| 1 | ADD 3-bromo-1,1,1-trifluoroacetone | ADD 3-bromo-1,1,1-trifluoroacetone |
| 2 | ADD DMF | ADD DMF |
| 3 | STIR for 3600 s at 100 °C | STIR for 86400 s at 100 °C |
| 4 | SETTEMPERATURE 25 °C | CONCENTRATE |
| 5 | ADD NaHCO3 | PURIFY |
| 6 | EXTRACT with ether | YIELD Ethyl 2-[4-(trifluoromethyl)-1,3-thiazol-2-yl]propanoate |
| 7 | PHASESEPARATION |  |
| 8 | COLLECTLAYER organic |  |
| 9 | WASH with brine |  |
| 10 | DRYSOLUTION over Na2SO4 |  |
| 11 | CONCENTRATE |  |
| 12 | PURIFY |  |
| 13 | YIELD Ethyl 2-[4-(trifluoromethyl)-1,3-thiazol-2-yl]propanoate |  |

---

```
Reaction no 140
```

Generated by the Chemistry Development Kit (http://github.com/cdk)

|  | A | B |
| --- | --- | --- |
| 0 | ADD 5-(1-bromoethyl)-6-methyl-4-(3-nitrophenyl)-2-phenylpyrimidine | ADD 5-(1-bromoethyl)-6-methyl-4-(3-nitrophenyl)-2-phenylpyrimidine |
| 1 | ADD 1-methylpiperazine | ADD 1-methylpiperazine |
| 2 | ADD isopropanol | ADD isopropanol |
| 3 | REFLUX for 28800 s | REFLUX for 3600 s |
| 4 | CONCENTRATE | CONCENTRATE |
| 5 | ADD water | PURIFY |
| 6 | EXTRACT with chloroform | COLLECTLAYER organic |
| 7 | COLLECTLAYER organic | CONCENTRATE |
| 8 | WASH with sodium chloride | RECRYSTALLIZE from hexane |
| 9 | DRYSOLUTION over magnesium sulfate | WASH with ether |
| 10 | CONCENTRATE | YIELD 6-methyl-5-[1-(4-methylpiperazin-1-yl)ethyl]-4-(3-nitrophenyl)-2-phenylpyrimidine |
| 11 | PURIFY |  |
| 12 | COLLECTLAYER organic |  |
| 13 | CONCENTRATE |  |
| 14 | RECRYSTALLIZE from ethyl acetate |  |
| 15 | YIELD 6-methyl-5-[1-(4-methylpiperazin-1-yl)ethyl]-4-(3-nitrophenyl)-2-phenylpyrimidine |  |

---

```
Reaction no 141
```

Generated by the Chemistry Development Kit (http://github.com/cdk)

|  | A | B |
| --- | --- | --- |
| 0 | ADD 6,7-Dichloro-2-[4-(2-morpholin-4-ylethoxy)phenyl]-3H-imidazo[4,5-b]pyridine | ADD 6,7-Dichloro-2-[4-(2-morpholin-4-ylethoxy)phenyl]-3H-imidazo[4,5-b]pyridine |
| 1 | ADD morpholine | ADD morpholine |
| 2 | STIR for 86400 s at 100 °C | MICROWAVE for 600 s at 100 °C |
| 3 | CONCENTRATE | CONCENTRATE |
| 4 | PURIFY | TRITURATE with ethyl acetate |
| 5 | YIELD 6-Chloro-7-{4-morpholinyl)-2-{4-[2-(4-morpholinyl)ethoxy]phenyl}-1H-imidazo[4,5-b]pyridine | FILTER keep precipitate |
| 6 |  | WASH with ethyl acetate / heptane |
| 7 |  | YIELD 6-Chloro-7-{4-morpholinyl)-2-{4-[2-(4-morpholinyl)ethoxy]phenyl}-1H-imidazo[4,5-b]pyridine |

---

```
Reaction no 142
```

Generated by the Chemistry Development Kit (http://github.com/cdk)

|  | A | B |
| --- | --- | --- |
| 0 | ADD HBr | ADD (2,3-dichloro-4-methoxyphenyl)(1-methyl-5-pyrazolyl)methanone |
| 1 | ADD (2,3-dichloro-4-methoxyphenyl)(1-methyl-5-pyrazolyl)methanone | ADD HBr |
| 2 | REFLUX for 28800 s | STIR for 86400 s at 100 °C |
| 3 | CONCENTRATE | SETTEMPERATURE 25 °C |
| 4 | ADD sodium hydroxide | FILTER keep precipitate |
| 5 | FILTER keep filtrate | WASH with water |
| 6 | PH with acetic acid to pH acidic | DRYSOLID under vacuum |
| 7 | FILTER keep precipitate | YIELD 2,3-dichloro-4-(1-methylpyrazolylcarbonyl)phenol |
| 8 | WASH with water |  |
| 9 | DRYSOLID |  |
| 10 | YIELD 2,3-dichloro-4-(1-methylpyrazolylcarbonyl)phenol |  |

---

```
Reaction no 143
```

Generated by the Chemistry Development Kit (http://github.com/cdk)

|  | A | B |
| --- | --- | --- |
| 0 | ADD 1-benzyl-4-(3-methyl-1-(pyridin-2-yl)-1H-pyrazol-5-yl)piperazine | ADD 1-benzyl-4-(3-methyl-1-(pyridin-2-yl)-1H-pyrazol-5-yl)piperazine |
| 1 | ADD ethanol | ADD ethanol |
| 2 | ADD formic acid | SETTEMPERATURE 25 °C |
| 3 | ADD ammonium formate | ADD HCl |
| 4 | ADD Pd/C | CONCENTRATE |
| 5 | REFLUX for 3600 s | ADD Pd/C |
| 6 | SETTEMPERATURE 25 °C | ADD formic acid |
| 7 | FILTER keep filtrate | STIR for 3600 s at 60 °C |
| 8 | WASH with methanol | STIR for 3600 s at 60 °C |
| 9 | CONCENTRATE | ADD Pd/C |
| 10 | ADD HCl | ADD ammonium formate |
| 11 | WASH with methanol | STIR for 28800 s at 60 °C |
| 12 | WASH with ammonia / methanol | SETTEMPERATURE 25 °C |
| 13 | CONCENTRATE | FILTER keep filtrate |
| 14 | YIELD 1-(3-methyl-1-(pyridin-2-yl)-1H-pyrazol-5-yl)piperazine | WASH with ethanol |
| 15 |  | PH with NaOH to pH basic |
| 16 |  | ADD water |
| 17 |  | STIR for 3600 s |
| 18 |  | CONCENTRATE |
| 19 |  | COLLECTLAYER aqueous |
| 20 |  | EXTRACT with isopropanol / ethyl acetate |
| 21 |  | COLLECTLAYER organic |
| 22 |  | DRYSOLUTION over Na2SO4 |
| 23 |  | CONCENTRATE |
| 24 |  | YIELD 1-(3-methyl-1-(pyridin-2-yl)-1H-pyrazol-5-yl)piperazine |

---

```
Reaction no 144
```

Generated by the Chemistry Development Kit (http://github.com/cdk)

|  | A | B |
| --- | --- | --- |
| 0 | ADD (4-amino-2,2-dimethyl-1-piperidyl)-[7-tert-butyl-5-(4-chloro-3-fluoro-phenyl)furo[3,2-b]pyridin-2-yl]methanone | ADD methanesulfonamide |
| 1 | ADD triethylamine | ADD acetonitrile |
| 2 | ADD acetonitrile | ADD NMP |
| 3 | ADD CDI at 25 °C | ADD CDI |
| 4 | STIR for 28800 s | STIR for 3600 s at 25 °C |
| 5 | ADD methanesulfonamide | ADD (4-amino-2,2-dimethyl-1-piperidyl)-[7-tert-butyl-5-(4-chloro-3-fluoro-phenyl)furo[3,2-b]pyridin-2-yl]methanone |
| 6 | ADD DBU | ADD acetonitrile |
| 7 | SETTEMPERATURE 60 °C | ADD DBU |
| 8 | STIR for 86400 s at 60 °C | STIR for 86400 s at 25 °C |
| 9 | ADD NMP | PURIFY |
| 10 | PURIFY | YIELD 1-[1-[7-tert-butyl-5-(4-chloro-3-fluoro-phenyl)furo[3,2-b]pyridine-2-carbonyl]-2,2-dimethyl-4-piperidyl]-3-methylsulfonyl-urea |
| 11 | YIELD 1-[1-[7-tert-butyl-5-(4-chloro-3-fluoro-phenyl)furo[3,2-b]pyridine-2-carbonyl]-2,2-dimethyl-4-piperidyl]-3-methylsulfonyl-urea |  |

---

```
Reaction no 145
```

Generated by the Chemistry Development Kit (http://github.com/cdk)

|  | A | B |
| --- | --- | --- |
| 0 | ADD aluminum chloride | ADD aluminum chloride |
| 1 | ADD NaN3 | ADD THF |
| 2 | ADD THF | ADD NaN3 |
| 3 | REFLUX for 3600 s | REFLUX for 3600 s |
| 4 | SETTEMPERATURE 0 °C | ADD 1,6-Dihydro-6-oxo-2-(2-methoxyphenyl)pyrimidine-5-carbonitrile |
| 5 | ADD 1,6-Dihydro-6-oxo-2-(2-methoxyphenyl)pyrimidine-5-carbonitrile | REFLUX for 86400 s |
| 6 | REFLUX for 86400 s | ADD water |
| 7 | SETTEMPERATURE 25 °C | PH with HCl to pH acidic |
| 8 | ADD water | FILTER |
| 9 | PH with HCl to pH acidic | RECRYSTALLIZE from acetic acid |
| 10 | EXTRACT with ethyl acetate | YIELD 2-(2-Methoxyphenyl)-5-(5-1H-tetrazolyl)pyrimidin-4(3H)-one |
| 11 | COLLECTLAYER organic |  |
| 12 | WASH with water |  |
| 13 | WASH with brine |  |
| 14 | DRYSOLUTION over magnesium sulfate |  |
| 15 | CONCENTRATE |  |
| 16 | YIELD 2-(2-Methoxyphenyl)-5-(5-1H-tetrazolyl)pyrimidin-4(3H)-one |  |

---

```
Reaction no 146
```

Generated by the Chemistry Development Kit (http://github.com/cdk)

|  | A | B |
| --- | --- | --- |
| 0 | ADD dimethylamine | ADD dimethylamine |
| 1 | ADD THF | ADD THF |
| 2 | ADD dichloromethane | ADD methyl-2-(chlorosulfonyl)acetate at 0 °C |
| 3 | ADD methyl-2-(chlorosulfonyl)acetate | STIR for 3600 s at 25 °C |
| 4 | SETTEMPERATURE 0 °C | ADD dichloromethane |
| 5 | ADD dichloromethane | ADD brine |
| 6 | STIR for 3600 s at 0 °C | PHASESEPARATION |
| 7 | ADD brine | COLLECTLAYER organic |
| 8 | COLLECTLAYER aqueous | DRYSOLUTION over MgSO4 |
| 9 | EXTRACT with dichloromethane | FILTER keep filtrate |
| 10 | COLLECTLAYER organic | CONCENTRATE |
| 11 | DRYSOLUTION over Na2SO4 | YIELD methyl 2-(N,N-dimethylsulfamoyl)acetate |
| 12 | FILTER keep filtrate |  |
| 13 | CONCENTRATE |  |
| 14 | PURIFY |  |
| 15 | YIELD methyl 2-(N,N-dimethylsulfamoyl)acetate |  |

---

```
Reaction no 147
```

Generated by the Chemistry Development Kit (http://github.com/cdk)

|  | A | B |
| --- | --- | --- |
| 0 | ADD 3-(3-aminomethyl-1,2,4-oxadiazol-5-yl)-5,6-dihydro-5-methyl-4H-imidazo[1,5-a][1,4]benzodiazepin-6-one | ADD 3-(3-aminomethyl-1,2,4-oxadiazol-5-yl)-5,6-dihydro-5-methyl-4H-imidazo[1,5-a][1,4]benzodiazepin-6-one |
| 1 | ADD DMF | ADD 5-bromo-1-pentene |
| 2 | ADD DIPEA | ADD DIPEA |
| 3 | ADD 5-bromo-1-pentene | ADD DMF |
| 4 | STIR for 3600 s at 60 °C | STIR for 86400 s at 100 °C |
| 5 | CONCENTRATE | PURIFY |
| 6 | PURIFY | YIELD 3-{3-[bis-(pent-4-enyl)aminomethyl]-1,2,4-oxadiazol-5-yl)-5-methyl-5,6-dihydro-4H-imidazo[1,5-a][1,4]benzodiazepin-6-one |
| 7 | YIELD 3-{3-[bis-(pent-4-enyl)aminomethyl]-1,2,4-oxadiazol-5-yl)-5-methyl-5,6-dihydro-4H-imidazo[1,5-a][1,4]benzodiazepin-6-one |  |

---

```
Reaction no 148
```

Generated by the Chemistry Development Kit (http://github.com/cdk)

|  | A | B |
| --- | --- | --- |
| 0 | ADD N-{3-[5-(2-Chloro-4-pyrimidinyl)-2-methyl-1,3-oxazol-4-yl]phenyl}-2,5-difluorobenzamide | ADD N-{3-[5-(2-Chloro-4-pyrimidinyl)-2-methyl-1,3-oxazol-4-yl]phenyl}-2,5-difluorobenzamide |
| 1 | ADD {2-[(4-amino-2-chlorophenyl)oxy]ethyl}dimethylamine hydrochloride | ADD {2-[(4-amino-2-chlorophenyl)oxy]ethyl}dimethylamine hydrochloride |
| 2 | ADD isopropanol | ADD isopropanol |
| 3 | ADD HCl | ADD HCl |
| 4 | MICROWAVE for 600 s at 100 °C | STIR for 600 s at 100 °C |
| 5 | ADD dichloromethane | ADD dichloromethane |
| 6 | WASH with NaHCO3 | CONCENTRATE |
| 7 | DRYSOLUTION over MgSO4 | PURIFY |
| 8 | FILTER keep filtrate | YIELD N-[3-(5-{2-[(3-Chloro-4-{[2-(dimethylamino)ethyl]oxy}phenyl)amino]-4-pyrimidinyl}-2-methyl-1,3-oxazol-4-yl)phenyl]-2,5-difluorobenzamide |
| 9 | CONCENTRATE |  |
| 10 | PURIFY |  |
| 11 | YIELD N-[3-(5-{2-[(3-Chloro-4-{[2-(dimethylamino)ethyl]oxy}phenyl)amino]-4-pyrimidinyl}-2-methyl-1,3-oxazol-4-yl)phenyl]-2,5-difluorobenzamide |  |

---

```
Reaction no 149
```

Generated by the Chemistry Development Kit (http://github.com/cdk)

|  | A | B |
| --- | --- | --- |
| 0 | ADD 2,6-di-tert-butylphenol | ADD 2,6-di-tert-butylphenol |
| 1 | ADD 5-methylisoxazole-3-carbonyl chloride | ADD 5-methylisoxazole-3-carbonyl chloride |
| 2 | ADD carbon disulfide | ADD carbon disulfide |
| 3 | ADD aluminum chloride at 0 °C | ADD aluminum chloride at 0 °C |
| 4 | REFLUX for 86400 s | STIR for 3600 s at 25 °C |
| 5 | SETTEMPERATURE 25 °C | STIR for 3600 s at 25 °C |
| 6 | ADD ice water | ADD HCl |
| 7 | EXTRACT with ethyl acetate | EXTRACT with ether |
| 8 | WASH with water | WASH with NaHCO3 |
| 9 | DRYSOLUTION over magnesium sulfate | WASH with brine |
| 10 | CONCENTRATE | DRYSOLUTION over Na2SO4 |
| 11 | PURIFY | CONCENTRATE |
| 12 | YIELD [3,5-Bis(1,1-dimethylethyl)-4-hydroxyphenyl](5-methyl-3-isoxazolyl)methanon | PHASESEPARATION |
| 13 |  | COLLECTLAYER organic |
| 14 |  | PURIFY |
| 15 |  | YIELD [3,5-Bis(1,1-dimethylethyl)-4-hydroxyphenyl](5-methyl-3-isoxazolyl)methanon |

---

```
Reaction no 150
```

Generated by the Chemistry Development Kit (http://github.com/cdk)

|  | A | B |
| --- | --- | --- |
| 0 | ADD dimethyl 3,3′-((((2,2′-dimethyl-[1,1′-biphenyl]-3,3′-diyl)bis(methylene))bis(oxy))bis(5-chloro-2-methoxypyridine-6,3-diyl))bis(3-(dimethylamino)propanoate) | ADD dimethyl 3,3′-((((2,2′-dimethyl-[1,1′-biphenyl]-3,3′-diyl)bis(methylene))bis(oxy))bis(5-chloro-2-methoxypyridine-6,3-diyl))bis(3-(dimethylamino)propanoate) |
| 1 | ADD H2O THF DMSO | ADD H2O THF DMSO |
| 2 | ADD LiOH | ADD LiOH |
| 3 | STIR for 3600 s | STIR for 86400 s at 25 °C |
| 4 | PURIFY | CONCENTRATE |
| 5 | YIELD 3,3′-((((2,2′-dimethyl-[1,1′-biphenyl]-3,3′-diyl)bis(methylene))bis(oxy))bis(5-chloro-2-methoxypyridine-6,3-diyl))bis(3-(dimethylamino)propanoic acid) | PH with HCl to pH acidic |
| 6 |  | EXTRACT with dichloromethane |
| 7 |  | COLLECTLAYER organic |
| 8 |  | CONCENTRATE |
| 9 |  | YIELD 3,3′-((((2,2′-dimethyl-[1,1′-biphenyl]-3,3′-diyl)bis(methylene))bis(oxy))bis(5-chloro-2-methoxypyridine-6,3-diyl))bis(3-(dimethylamino)propanoic acid) |

---

```
Reaction no 151
```

Generated by the Chemistry Development Kit (http://github.com/cdk)

|  | A | B |
| --- | --- | --- |
| 0 | ADD N-(1-methylethyl)-1,3-propanediamine | ADD N-(1-methylethyl)-1,3-propanediamine |
| 1 | ADD isopropanol | ADD isopropanol |
| 2 | ADD formaldehyde dropwise | ADD formaldehyde at 25 °C |
| 3 | WAIT for 3600 s | STIR for 86400 s at 25 °C |
| 4 | CONCENTRATE | CONCENTRATE |
| 5 | CONCENTRATE | YIELD N-isopropylhexahydropyrimidine |
| 6 | YIELD N-isopropylhexahydropyrimidine |  |

---

```
Reaction no 152
```

Generated by the Chemistry Development Kit (http://github.com/cdk)

|  | A | B |
| --- | --- | --- |
| 0 | ADD 2-(2-Chlorophenyl)-N-{3-[(2,4-dimethoxybenzyl)sulfamoyl]-4-(2H-pyrazolo[4,3-b]pyridin-2-yl)phenyl}acetamide | ADD 2-(2-Chlorophenyl)-N-{3-[(2,4-dimethoxybenzyl)sulfamoyl]-4-(2H-pyrazolo[4,3-b]pyridin-2-yl)phenyl}acetamide |
| 1 | ADD dichloromethane | ADD dichloromethane |
| 2 | ADD TFA | ADD TFA |
| 3 | STIR for 86400 s at 25 °C | STIR for 86400 s at 25 °C |
| 4 | CONCENTRATE | CONCENTRATE |
| 5 | PURIFY | PURIFY |
| 6 | YIELD 2-(2-Chlorophenyl)-N-[4-(2H-pyrazolo[4,3-b]pyridin-2-yl)-3-sulfamoylphenyl]-acetamide | YIELD 2-(2-Chlorophenyl)-N-[4-(2H-pyrazolo[4,3-b]pyridin-2-yl)-3-sulfamoylphenyl]-acetamide |

---

```
Reaction no 153
```

Generated by the Chemistry Development Kit (http://github.com/cdk)

|  | A | B |
| --- | --- | --- |
| 0 | ADD 2-(4-Bromo-3-chloro-phenyl)-1-propyl-1H-benzoimidazole | ADD 2-(4-Bromo-3-chloro-phenyl)-1-propyl-1H-benzoimidazole |
| 1 | ADD 6-methylpyridin-3-amine | ADD 6-methylpyridin-3-amine |
| 2 | ADD Pd(OAc)2 | ADD BINAP |
| 3 | ADD BINAP | ADD Pd(OAc)2 |
| 4 | ADD K2CO3 | ADD K2CO3 |
| 5 | ADD toluene | ADD toluene |
| 6 | STIR for 86400 s at 100 °C | STIR for 86400 s at 100 °C |
| 7 | ADD ethyl acetate | SETTEMPERATURE 25 °C |
| 8 | WASH with water | ADD ethyl acetate |
| 9 | DRYSOLUTION over Na2SO4 | WASH with NaHCO3 |
| 10 | CONCENTRATE | WASH with water |
| 11 | PURIFY | COLLECTLAYER aqueous |
| 12 | YIELD [2-Chloro-4-(1-propyl-1H-benzoimidazol-2-yl)-phenyl]-(6-methyl-pyridin-3-yl)-amine | EXTRACT with ethyl acetate |
| 13 |  | COLLECTLAYER organic |
| 14 |  | DRYSOLUTION over Na2SO4 |
| 15 |  | FILTER keep filtrate |
| 16 |  | CONCENTRATE |
| 17 |  | PURIFY |
| 18 |  | YIELD [2-Chloro-4-(1-propyl-1H-benzoimidazol-2-yl)-phenyl]-(6-methyl-pyridin-3-yl)-amine |

---

```
Reaction no 154
```

Generated by the Chemistry Development Kit (http://github.com/cdk)

|  | A | B |
| --- | --- | --- |
| 0 | ADD (3-Chloro-5-methyl-phenyl)-acetonitrile | ADD (3-Chloro-5-methyl-phenyl)-acetonitrile |
| 1 | ADD KOH | ADD KOH |
| 2 | ADD isopropanol water | ADD isopropanol water |
| 3 | REFLUX for 86400 s | STIR for 86400 s at 100 °C |
| 4 | CONCENTRATE | STIR for 86400 s at 25 °C |
| 5 | ADD water | CONCENTRATE |
| 6 | PH with HCl to pH acidic | PH with HCl to pH acidic |
| 7 | EXTRACT with ethyl acetate | EXTRACT with ether |
| 8 | COLLECTLAYER organic | COLLECTLAYER organic |
| 9 | WASH with brine | WASH with water |
| 10 | DRYSOLUTION over MgSO4 | DRYSOLUTION over Na2SO4 |
| 11 | CONCENTRATE | CONCENTRATE |
| 12 | YIELD 2-(3-chloro-5-methylphenyl)acetic acid | YIELD 2-(3-chloro-5-methylphenyl)acetic acid |

---

```
Reaction no 155
```

Generated by the Chemistry Development Kit (http://github.com/cdk)

|  | A | B |
| --- | --- | --- |
| 0 | ADD (S)-6-chloro-N-(1-(3-fluoro-4-(trifluoromethoxy)phenyl)-2-methoxyethyl)pyrazine-2-carboxamide | ADD (S)-6-chloro-N-(1-(3-fluoro-4-(trifluoromethoxy)phenyl)-2-methoxyethyl)pyrazine-2-carboxamide |
| 1 | ADD (S)-3-methoxypyrrolidine hydrochloride | ADD DMA |
| 2 | ADD K2CO3 | ADD (S)-3-methoxypyrrolidine hydrochloride |
| 3 | ADD DMA | ADD K2CO3 |
| 4 | STIR for 86400 s at 100 °C | STIR for 3600 s at 100 °C |
| 5 | ADD water | ADD water |
| 6 | FILTER keep precipitate | EXTRACT with ethyl acetate |
| 7 | WASH with ethyl acetate / hexane | COLLECTLAYER organic |
| 8 | YIELD N-((1S)-1-(3-fluoro-4-(trifluoromethoxy)phenyl)-2-methoxyethyl)-6-((3S)-3-methoxypyrrolidin-1-yl)pyrazine-2-carboxamide | WASH with brine |
| 9 |  | DRYSOLUTION over magnesium sulfate |
| 10 |  | CONCENTRATE |
| 11 |  | PURIFY |
| 12 |  | YIELD N-((1S)-1-(3-fluoro-4-(trifluoromethoxy)phenyl)-2-methoxyethyl)-6-((3S)-3-methoxypyrrolidin-1-yl)pyrazine-2-carboxamide |

---

```
Reaction no 156
```

Generated by the Chemistry Development Kit (http://github.com/cdk)

|  | A | B |
| --- | --- | --- |
| 0 | ADD 2,3,5,6,7,11c-hexahydro-1H-pyrido[3′,2′:4,5]pyrrolo[2,3-g]indolizine | ADD 2,3,5,6,7,11c-hexahydro-1H-pyrido[3′,2′:4,5]pyrrolo[2,3-g]indolizine |
| 1 | ADD KOH | ADD KOH |
| 2 | ADD NMP | ADD NMP |
| 3 | STIR for 600 s at 25 °C | STIR for 600 s at 25 °C |
| 4 | ADD 2-(trifluoromethyl)-5-vinylpyridine | ADD 2-(trifluoromethyl)-5-vinylpyridine |
| 5 | STIR for 86400 s at 25 °C | STIR for 86400 s at 25 °C |
| 6 | ADD water | ADD water |
| 7 | EXTRACT with ethyl acetate | EXTRACT with ethyl acetate |
| 8 | COLLECTLAYER organic | COLLECTLAYER organic |
| 9 | WASH with water | WASH with water |
| 10 | DRYSOLUTION over sodium sulfate | DRYSOLUTION over sodium sulfate |
| 11 | CONCENTRATE | CONCENTRATE |
| 12 | PURIFY | PURIFY |
| 13 | YIELD 7-(2-(6-(trifluoromethyl)pyridin-3-yl)ethyl)-2,3,5,6,7,11c-hexahydro-1H-pyrido[3′,2′:4,5]pyrrolo[2,3-g]indolizine | YIELD 7-(2-(6-(trifluoromethyl)pyridin-3-yl)ethyl)-2,3,5,6,7,11c-hexahydro-1H-pyrido[3′,2′:4,5]pyrrolo[2,3-g]indolizine |

---

```
Reaction no 157
```

Generated by the Chemistry Development Kit (http://github.com/cdk)

|  | A | B |
| --- | --- | --- |
| 0 | ADD 3-cyano-17β-(phenethylcarbonyl)-androsta-3,5-diene | ADD 3-cyano-17β-(phenethylcarbonyl)-androsta-3,5-diene |
| 1 | ADD ethanol | ADD ethanol |
| 2 | REFLUX | MICROWAVE for 28800 s at 100 °C |
| 3 | QUENCH with HCl | CONCENTRATE |
| 4 | EXTRACT with dichloromethane | PURIFY |
| 5 | YIELD 17β-(phenethylcarbonyl)-androsta-3,5-diene-3-carboxylic acid | YIELD 17β-(phenethylcarbonyl)-androsta-3,5-diene-3-carboxylic acid |

---

```
Reaction no 158
```

Generated by the Chemistry Development Kit (http://github.com/cdk)

|  | A | B |
| --- | --- | --- |
| 0 | ADD 4-[7-[3-cyano-5-fluoro-4-([5-methoxy-4-oxaspiro[2.5]octan-7-yl]oxy)phenyl]furo[3,2-b]pyridin-2-yl]-N,N-dimethylbenzamide | ADD 4-[7-[3-cyano-5-fluoro-4-([5-methoxy-4-oxaspiro[2.5]octan-7-yl]oxy)phenyl]furo[3,2-b]pyridin-2-yl]-N,N-dimethylbenzamide |
| 1 | ADD dichloromethane | ADD dichloromethane |
| 2 | ADD triethylsilane at 0 °C | ADD triethylsilane at 0 °C |
| 3 | ADD BF3.Et2O at 0 °C | ADD BF3.Et2O at 0 °C |
| 4 | STIR for 3600 s at 0 °C | STIR for 600 s at 0 °C |
| 5 | QUENCH with water | QUENCH with water |
| 6 | EXTRACT with dichloromethane | CONCENTRATE |
| 7 | COLLECTLAYER organic | PURIFY |
| 8 | WASH with brine | YIELD 4-[7-(3-cyano-5-fluoro-4-[4-oxaspiro[2.5]octan-7-yloxy]phenyl)furo[3,2-b]pyridin-2-yl]-N,N-dimethylbenzamide hydrochloride |
| 9 | DRYSOLUTION over sodium sulfate |  |
| 10 | CONCENTRATE |  |
| 11 | PURIFY |  |
| 12 | YIELD 4-[7-(3-cyano-5-fluoro-4-[4-oxaspiro[2.5]octan-7-yloxy]phenyl)furo[3,2-b]pyridin-2-yl]-N,N-dimethylbenzamide hydrochloride |  |

---

```
Reaction no 159
```

Generated by the Chemistry Development Kit (http://github.com/cdk)

|  | A | B |
| --- | --- | --- |
| 0 | ADD 1-(3-Aminopropyl)-2-(ethoxymethyl)-1H-imidazo[4,5-c]quinolin-4-amine | ADD 1-(3-Aminopropyl)-2-(ethoxymethyl)-1H-imidazo[4,5-c]quinolin-4-amine |
| 1 | ADD DMF | ADD DMF |
| 2 | SETTEMPERATURE 25 °C | ADD N,N′-bis(tert-butoxycarbonyl)-1H-pyrazole-1-carboxamidine |
| 3 | ADD N,N′-bis(tert-butoxycarbonyl)-1H-pyrazole-1-carboxamidine | STIR for 86400 s at 25 °C |
| 4 | STIR for 28800 s | CONCENTRATE |
| 5 | CONCENTRATE | PURIFY |
| 6 | ADD chloroform | YIELD tert-butyl-N-[[3-(4-amino-2-(ethoxymethyl)imidazo[4,5-c]quinolin-1-yl)propylamino]-(tert-butoxycarbonylamino)methylene]carbamate |
| 7 | WASH with water |  |
| 8 | WASH with brine |  |
| 9 | COLLECTLAYER organic |  |
| 10 | DRYSOLUTION over Na2SO4 |  |
| 11 | FILTER keep filtrate |  |
| 12 | CONCENTRATE |  |
| 13 | RECRYSTALLIZE from acetonitrile |  |
| 14 | YIELD tert-butyl-N-[[3-(4-amino-2-(ethoxymethyl)imidazo[4,5-c]quinolin-1-yl)propylamino]-(tert-butoxycarbonylamino)methylene]carbamate |  |

---

```
Reaction no 160
```

Generated by the Chemistry Development Kit (http://github.com/cdk)

|  | A | B |
| --- | --- | --- |
| 0 | ADD 2-(difluoromethoxy)-4-fluoro-5-nitroaniline | ADD 2-(difluoromethoxy)-4-fluoro-5-nitroaniline |
| 1 | ADD N,N,N′-trimethylethylenediamine | ADD N,N,N′-trimethylethylenediamine |
| 2 | ADD acetonitrile | ADD K2CO3 |
| 3 | ADD K2CO3 at 25 °C | ADD acetonitrile |
| 4 | STIR for 28800 s at 100 °C | STIR for 86400 s at 100 °C |
| 5 | SETTEMPERATURE 25 °C | SETTEMPERATURE 25 °C |
| 6 | FILTER keep filtrate | FILTER keep filtrate |
| 7 | CONCENTRATE | CONCENTRATE |
| 8 | PHASESEPARATION | PURIFY |
| 9 | YIELD 5-(difluoromethoxy)-N1-(2-(dimethylamino)ethyl)-N1-methyl-2-nitrobenzene-1,4-diamine | YIELD 5-(difluoromethoxy)-N1-(2-(dimethylamino)ethyl)-N1-methyl-2-nitrobenzene-1,4-diamine |

---

```
Reaction no 161
```

Generated by the Chemistry Development Kit (http://github.com/cdk)

|  | A | B |
| --- | --- | --- |
| 0 | ADD cis-N-(2-{[7-Amino-8-(2-fluorobenzyl)-5,6,7,8-tetrahydronaphthalen-2-yl]oxy}ethyl)-1-methyl-1H-imidazole-4-sulfonamide | ADD cis-N-(2-{[7-Amino-8-(2-fluorobenzyl)-5,6,7,8-tetrahydronaphthalen-2-yl]oxy}ethyl)-1-methyl-1H-imidazole-4-sulfonamide |
| 1 | ADD acetyl chloride | ADD acetyl chloride |
| 2 | REFLUX for 3600 s | STIR for 600 s at 100 °C |
| 3 | CONCENTRATE | PURIFY |
| 4 | ADD dichloromethane | YIELD cis-N-(2-{[7-(2,4-Dioxo-3-azabicyclo[3.1.0]hex-3-yl)-8-(2-fluorobenzyl)-5,6,7,8-tetrahydronaphthalen-2-yl]oxy}ethyl)-1-methyl-1H-imidazole-4-sulfonamide |
| 5 | WASH with sodium bicarbonate |  |
| 6 | WASH with sodium chloride |  |
| 7 | DRYSOLUTION over magnesium sulfate |  |
| 8 | YIELD cis-N-(2-{[7-(2,4-Dioxo-3-azabicyclo[3.1.0]hex-3-yl)-8-(2-fluorobenzyl)-5,6,7,8-tetrahydronaphthalen-2-yl]oxy}ethyl)-1-methyl-1H-imidazole-4-sulfonamide |  |

---

```
Reaction no 162
```

Generated by the Chemistry Development Kit (http://github.com/cdk)

|  | A | B |
| --- | --- | --- |
| 0 | ADD dimethyl 4-chloropyridine-2,6-dicarboxylate | ADD dimethyl 4-chloropyridine-2,6-dicarboxylate |
| 1 | ADD Natriumiodid | ADD Natriumiodid |
| 2 | ADD acetonitrile | ADD acetonitrile |
| 3 | ADD acetyl chloride | ADD acetyl chloride at 0 °C |
| 4 | STIR for 3600 s at 60 °C | STIR for 28800 s at 60 °C |
| 5 | SETTEMPERATURE 25 °C | CONCENTRATE |
| 6 | QUENCH with sodium thiosulfate | PH with sodium carbonate to pH neutral |
| 7 | EXTRACT with ethyl acetate | EXTRACT with dichloromethane |
| 8 | COLLECTLAYER organic | COLLECTLAYER organic |
| 9 | WASH with brine | WASH with sodium thiosulfate |
| 10 | DRYSOLUTION over sodium sulfate | WASH with water |
| 11 | CONCENTRATE | DRYSOLUTION over Na2SO4 |
| 12 | PURIFY | CONCENTRATE |
| 13 | YIELD dimethyl 4-iodopyridine-2,6-dicarboxylate | YIELD dimethyl 4-iodopyridine-2,6-dicarboxylate |

---

```
Reaction no 163
```

Generated by the Chemistry Development Kit (http://github.com/cdk)

|  | A | B |
| --- | --- | --- |
| 0 | ADD [(2S)-8-methoxy-1,2,3,4-tetrahydro-naphthalen-2-yl]-dimethyl-amine | ADD [(2S)-8-methoxy-1,2,3,4-tetrahydro-naphthalen-2-yl]-dimethyl-amine |
| 1 | ADD TFA | ADD TFA |
| 2 | ADD nitrate de sodium at 0 °C | ADD nitrate de sodium at 0 °C |
| 3 | STIR for 86400 s at 25 °C | STIR for 3600 s at 25 °C |
| 4 | ADD ice water | PH with ammonia to pH acidic |
| 5 | PH with ammonia to pH basic | COLLECTLAYER aqueous |
| 6 | EXTRACT with ethyl acetate | EXTRACT with dichloromethane |
| 7 | COLLECTLAYER organic | COLLECTLAYER organic |
| 8 | DRYSOLUTION over Na2SO4 | WASH with sodium chloride |
| 9 | CONCENTRATE | DRYSOLUTION over Na2SO4 |
| 10 | PURIFY | FILTER keep filtrate |
| 11 | YIELD (2S)-8-Methoxy-N,N-dimethyl-5-nitro-1,2,3,4-tetrahydronaphthalen-2-amine | CONCENTRATE |
| 12 |  | PURIFY |
| 13 |  | FILTER keep precipitate |
| 14 |  | CONCENTRATE |
| 15 |  | PURIFY |
| 16 |  | YIELD (2S)-8-Methoxy-N,N-dimethyl-5-nitro-1,2,3,4-tetrahydronaphthalen-2-amine |

---

```
Reaction no 164
```

Generated by the Chemistry Development Kit (http://github.com/cdk)

|  | A | B |
| --- | --- | --- |
| 0 | ADD methanol | ADD (8R,13R)-13-[[(1,1-dimethylethoxy)carbonyl]amino]-6,14-dioxo-10,11-dithia-7,15-diazaspiro[4.12]heptadecane-8-carboxylic acid 1,1-dimethylethyl ester |
| 1 | ADD ethyl acetate | ADD ethyl acetate |
| 2 | ADD acetyl chloride at 0 °C | ADD methanol |
| 3 | STIR for 600 s at 0 °C | ADD acetyl chloride at 0 °C |
| 4 | STIR for 3600 s at 25 °C | STIR for 3600 s at 25 °C |
| 5 | ADD (8R,13R)-13-[[(1,1-dimethylethoxy)carbonyl]amino]-6,14-dioxo-10,11-dithia-7,15-diazaspiro[4.12]heptadecane-8-carboxylic acid 1,1-dimethylethyl ester | CONCENTRATE |
| 6 | STIR for 28800 s | YIELD (8R, 13R)-13-amino-6,14-dioxo-10,11-dithia-7,15-diazaspiro[4.12]heptadecane-8-carboxylic acid 1,1-dimethylethyl ester |
| 7 | FILTER keep precipitate |  |
| 8 | WASH with ether |  |
| 9 | DRYSOLID under vacuum |  |
| 10 | YIELD (8R, 13R)-13-amino-6,14-dioxo-10,11-dithia-7,15-diazaspiro[4.12]heptadecane-8-carboxylic acid 1,1-dimethylethyl ester |  |

---

```
Reaction no 165
```

Generated by the Chemistry Development Kit (http://github.com/cdk)

|  | A | B |
| --- | --- | --- |
| 0 | ADD 2-Fluoro-3,4-dimethoxy-6-nitro-benzamide | ADD 2-Fluoro-3,4-dimethoxy-6-nitro-benzamide |
| 1 | ADD acetic acid | ADD acetic acid |
| 2 | ADD iron at 100 °C | ADD iron |
| 3 | STIR for 600 s at 100 °C | STIR for 3600 s at 100 °C |
| 4 | FILTER | FILTER keep filtrate |
| 5 | SETTEMPERATURE 25 °C | CONCENTRATE |
| 6 | QUENCH with water | PURIFY |
| 7 | EXTRACT with dichloromethane | YIELD 6-Amino-2-fluoro-3,4-dimethoxy-benzamide |
| 8 | COLLECTLAYER organic |  |
| 9 | DRYSOLUTION |  |
| 10 | CONCENTRATE |  |
| 11 | YIELD 6-Amino-2-fluoro-3,4-dimethoxy-benzamide |  |

---

```
Reaction no 166
```

Generated by the Chemistry Development Kit (http://github.com/cdk)

|  | A | B |
| --- | --- | --- |
| 0 | ADD 3-Acetyl-4-methoxy-1H-pyrrolo[2,3-b]pyridine | ADD 3-Acetyl-4-methoxy-1H-pyrrolo[2,3-b]pyridine |
| 1 | ADD dimethyl sulfate | ADD acetone |
| 2 | ADD K2CO3 | ADD K2CO3 |
| 3 | ADD acetone | ADD dimethyl sulfate |
| 4 | REFLUX for 28800 s | STIR for 86400 s at 25 °C |
| 5 | SETTEMPERATURE 25 °C | ADD water |
| 6 | CONCENTRATE | ADD ethyl acetate |
| 7 | ADD water | PHASESEPARATION |
| 8 | ADD ethyl acetate | COLLECTLAYER aqueous |
| 9 | PHASESEPARATION | EXTRACT with ethyl acetate |
| 10 | COLLECTLAYER organic | COLLECTLAYER organic |
| 11 | DRYSOLUTION over MgSO4 | DRYSOLUTION over MgSO4 |
| 12 | CONCENTRATE | FILTER keep filtrate |
| 13 | PURIFY | CONCENTRATE |
| 14 | YIELD 3-Acetyl-4-methoxy-1-methyl-1H-pyrrolo[2,3-b]pyridine | PURIFY |
| 15 |  | YIELD 3-Acetyl-4-methoxy-1-methyl-1H-pyrrolo[2,3-b]pyridine |

---

```
Reaction no 167
```

Generated by the Chemistry Development Kit (http://github.com/cdk)

|  | A | B |
| --- | --- | --- |
| 0 | ADD methyl 3-(4-methoxymethoxyphenyl)-2-methanesulfonyloxypropionate | ADD DMF |
| 1 | ADD DMF | ADD methyl 3-(4-methoxymethoxyphenyl)-2-methanesulfonyloxypropionate |
| 2 | ADD thiophenol | ADD thiophenol |
| 3 | ADD K2CO3 | ADD K2CO3 |
| 4 | STIR for 86400 s at 25 °C | STIR for 3600 s at 60 °C |
| 5 | ADD water | ADD ethyl acetate |
| 6 | EXTRACT with ethyl acetate | ADD water |
| 7 | COLLECTLAYER organic | PHASESEPARATION |
| 8 | WASH with brine | COLLECTLAYER organic |
| 9 | DRYSOLUTION over sodium sulfate | DRYSOLUTION over magnesium sulfate |
| 10 | CONCENTRATE | CONCENTRATE |
| 11 | PURIFY | PURIFY |
| 12 | YIELD Methyl 3-(4-methoxymethoxyphenyl)-2-(phenylthio)propionate | YIELD Methyl 3-(4-methoxymethoxyphenyl)-2-(phenylthio)propionate |

---

```
Reaction no 168
```

Generated by the Chemistry Development Kit (http://github.com/cdk)

|  | A | B |
| --- | --- | --- |
| 0 | ADD 5-bromo-2-chlorobenzoic acid | ADD 5-bromo-2-chlorobenzoic acid |
| 1 | ADD dichloromethane | ADD dichloromethane |
| 2 | ADD water | ADD (COCl)2 |
| 3 | ADD DMF | ADD DMF |
| 4 | ADD (COCl)2 at 25 °C | STIR for 86400 s at 25 °C |
| 5 | ADD HCl | ADD dry ice |
| 6 | ADD dry ice | ADD HCl |
| 7 | STIR for 86400 s at 25 °C | CONCENTRATE |
| 8 | CONCENTRATE | YIELD 5-bromo-2-chlorobenzoyl chloride |
| 9 | YIELD 5-bromo-2-chlorobenzoyl chloride |  |

---

```
Reaction no 169
```

Generated by the Chemistry Development Kit (http://github.com/cdk)

|  | A | B |
| --- | --- | --- |
| 0 | ADD 1,1,1-trifluoropropan-2-amine | ADD 1,1,1-trifluoropropan-2-amine |
| 1 | ADD K2CO3 | ADD 1-bromo-3-fluoropropane |
| 2 | ADD acetonitrile | ADD K2CO3 |
| 3 | ADD 1-bromo-3-fluoropropane at 25 °C over 3600 s | ADD acetonitrile |
| 4 | STIR for 86400 s at 25 °C | STIR for 86400 s at 100 °C |
| 5 | FILTER keep filtrate | SETTEMPERATURE 25 °C |
| 6 | CONCENTRATE | FILTER keep filtrate |
| 7 | CONCENTRATE | CONCENTRATE |
| 8 | YIELD (3-fluoropropyl)-(2,2,2-trifluoro-1-methyl-ethyl)-amine | YIELD (3-fluoropropyl)-(2,2,2-trifluoro-1-methyl-ethyl)-amine |

---

```
Reaction no 170
```

Generated by the Chemistry Development Kit (http://github.com/cdk)

|  | A | B |
| --- | --- | --- |
| 0 | ADD potassium t-butoxide | ADD 2,2,2-trifluoroethanol |
| 1 | ADD 2,2,2-trifluoroethanol | ADD 5-bromo-2-fluoropyridine |
| 2 | ADD MTBE | ADD MTBE |
| 3 | STIR for 3600 s at 25 °C | ADD potassium t-butoxide at 0 °C |
| 4 | ADD 5-bromo-2-fluoropyridine | STIR for 28800 s at 25 °C |
| 5 | STIR for 86400 s at 25 °C | ADD water |
| 6 | ADD water | EXTRACT with ethyl acetate |
| 7 | EXTRACT with MTBE | COLLECTLAYER organic |
| 8 | COLLECTLAYER organic | WASH with brine |
| 9 | DRYSOLUTION over sodium sulfate | DRYSOLUTION over Na2SO4 |
| 10 | FILTER keep filtrate | FILTER keep filtrate |
| 11 | CONCENTRATE | CONCENTRATE |
| 12 | PURIFY | PURIFY |
| 13 | YIELD 5-bromo-2-(2,2,2-trifluoroethoxy) pyridine | YIELD 5-bromo-2-(2,2,2-trifluoroethoxy) pyridine |

---

```
Reaction no 171
```

Generated by the Chemistry Development Kit (http://github.com/cdk)

|  | A | B |
| --- | --- | --- |
| 0 | ADD phenyl ‌(5-(tert-butyl)-2-methoxy-3-(methylsulfinyl)phenyl)carbamate | ADD phenyl ‌(5-(tert-butyl)-2-methoxy-3-(methylsulfinyl)phenyl)carbamate |
| 1 | ADD 4-((4-((4-aminonaphthalen-1-yl)oxy)pyridin-2-yl)amino)-2-methoxy-N-(2-morpholinoethyl)benzamide | ADD 4-((4-((4-aminonaphthalen-1-yl)oxy)pyridin-2-yl)amino)-2-methoxy-N-(2-morpholinoethyl)benzamide |
| 2 | ADD isopropyl acetate | ADD triethylamine |
| 3 | ADD triethylamine | ADD isopropyl acetate |
| 4 | STIR for 86400 s at 60 °C | STIR for 86400 s at 60 °C |
| 5 | CONCENTRATE | CONCENTRATE |
| 6 | PURIFY | PURIFY |
| 7 | PURIFY | YIELD 4-((4-((4-(3-(5-(tert-Butyl)-2-methoxy-3-(methylsulfinyl)phenyl)ureido)naphthalen-1-yl)oxy)pyridin-2-yl)amino)-2-methoxy-N-(2-morpholinoethyl)benzamide |
| 8 | YIELD 4-((4-((4-(3-(5-(tert-Butyl)-2-methoxy-3-(methylsulfinyl)phenyl)ureido)naphthalen-1-yl)oxy)pyridin-2-yl)amino)-2-methoxy-N-(2-morpholinoethyl)benzamide |  |

---

```
Reaction no 172
```

Generated by the Chemistry Development Kit (http://github.com/cdk)

|  | A | B |
| --- | --- | --- |
| 0 | ADD spiro[2.5]octan-6-ylmethanol | ADD spiro[2.5]octan-6-ylmethanol |
| 1 | ADD acetic acid ethyl acetate | ADD acetic acid ethyl acetate |
| 2 | ADD PtO2 | ADD PtO2 at 25 °C |
| 3 | STIR for 86400 s at 25 °C | STIR for 86400 s |
| 4 | FILTER keep filtrate | FILTER keep filtrate |
| 5 | CONCENTRATE | WASH with ethyl acetate |
| 6 | YIELD (4,4-dimethylcyclohexyl)methanol | CONCENTRATE |
| 7 |  | PURIFY |
| 8 |  | YIELD (4,4-dimethylcyclohexyl)methanol |

---

```
Reaction no 173
```

Generated by the Chemistry Development Kit (http://github.com/cdk)

|  | A | B |
| --- | --- | --- |
| 0 | ADD 4-benzyloxy-2-nitro-m-xylene | ADD 4-benzyloxy-2-nitro-m-xylene |
| 1 | ADD acetic acid | ADD acetic acid |
| 2 | ADD zinc | ADD zinc |
| 3 | STIR for 3600 s at 25 °C | FILTER keep filtrate |
| 4 | FILTER keep filtrate | WASH with acetic acid |
| 5 | CONCENTRATE | CONCENTRATE |
| 6 | ADD ethyl acetate | ADD water |
| 7 | WASH with NaHCO3 | PH with ammonium hydroxide to pH basic |
| 8 | WASH with brine | EXTRACT with chloroform |
| 9 | DRYSOLUTION over MgSO4 | COLLECTLAYER organic |
| 10 | CONCENTRATE | DRYSOLUTION |
| 11 | YIELD 3-benzyloxy-2,6-dimethylaniline | CONCENTRATE |
| 12 |  | YIELD 3-benzyloxy-2,6-dimethylaniline |

---

```
Reaction no 174
```

Generated by the Chemistry Development Kit (http://github.com/cdk)

|  | A | B |
| --- | --- | --- |
| 0 | ADD 2-Methylsulfanyl-5,6,7,8-tetrahydro-3H-quinazolin-4-one | ADD 2-Methylsulfanyl-5,6,7,8-tetrahydro-3H-quinazolin-4-one |
| 1 | ADD 1-(4-fluorophenyl)piperazine | ADD 1-(4-fluorophenyl)piperazine |
| 2 | ADD isoamyl alcohol | ADD isoamyl alcohol |
| 3 | STIR for 86400 s at 100 °C | MICROWAVE for 3600 s at 100 °C |
| 4 | SETTEMPERATURE 25 °C | FILTER keep precipitate |
| 5 | FILTER keep precipitate | WASH with ethanol |
| 6 | WASH with isoamyl alcohol | WASH with ether |
| 7 | DRYSOLID | FILTER |
| 8 | YIELD 2-[4-(4-Fluorophenyl)piperazin-1-yl]-5,6,7,8-tetrahydro-3H-quinazolin-4-one | WASH with ethanol |
| 9 |  | WASH with ether |
| 10 |  | FILTER keep precipitate |
| 11 |  | DRYSOLID at 60 °C under vacuum |
| 12 |  | YIELD 2-[4-(4-Fluorophenyl)piperazin-1-yl]-5,6,7,8-tetrahydro-3H-quinazolin-4-one |

---

```
Reaction no 175
```

Generated by the Chemistry Development Kit (http://github.com/cdk)

|  | A | B |
| --- | --- | --- |
| 0 | ADD diisopropylamine | ADD diisopropylamine |
| 1 | ADD THF | ADD THF |
| 2 | SETTEMPERATURE -70 °C | ADD n-butyllithium at -70 °C |
| 3 | MAKESOLUTION with n-butyllithium and hexanes | STIR for 3600 s at -70 °C |
| 4 | ADD SLN | MAKESOLUTION with chlorotitanium triisopropoxide and THF |
| 5 | STIR for 3600 s | ADD SLN |
| 6 | SETTEMPERATURE -70 °C | STIR for 3600 s at -70 °C |
| 7 | ADD methyl acetate | MAKESOLUTION with (R,E)-2-Methyl-N-(1-(8-(prop-1-yn-1-yl)dibenzo[b,d]thiophen-2-yl)ethylidene)propane-2-sulfinamide and THF |
| 8 | STIR for 3600 s at -70 °C | ADD SLN |
| 9 | MAKESOLUTION with chlorotitanium triisopropoxide and THF | STIR for 3600 s at -70 °C |
| 10 | ADD SLN | ADD methyl acetate |
| 11 | STIR for 3600 s | WASH with NaHCO3 |
| 12 | SETTEMPERATURE -70 °C | WASH with brine |
| 13 | MAKESOLUTION with (R,E)-2-Methyl-N-(1-(8-(prop-1-yn-1-yl)dibenzo[b,d]thiophen-2-yl)ethylidene)propane-2-sulfinamide and THF | DRYSOLUTION over MgSO4 |
| 14 | ADD SLN | FILTER keep filtrate |
| 15 | STIR for 3600 s | CONCENTRATE |
| 16 | WAIT for 3600 s | PURIFY |
| 17 | QUENCH with water | YIELD (S)-Methyl 3-((R)-1,1-dimethylethylsulfinamido)-3-(8-(prop-1-yn-1-yl) dibenzo[b,d]thiophen-2-yl)butanoate |
| 18 | EXTRACT with dichloromethane |  |
| 19 | FILTER keep precipitate |  |
| 20 | COLLECTLAYER organic |  |
| 21 | DRYSOLUTION over MgSO4 |  |
| 22 | CONCENTRATE |  |
| 23 | PURIFY |  |
| 24 | YIELD (S)-Methyl 3-((R)-1,1-dimethylethylsulfinamido)-3-(8-(prop-1-yn-1-yl) dibenzo[b,d]thiophen-2-yl)butanoate |  |

---

```
Reaction no 176
```

Generated by the Chemistry Development Kit (http://github.com/cdk)

|  | A | B |
| --- | --- | --- |
| 0 | ADD (1S,2S,6R)-2-azido-6-(3,6-difluoro-9H-carbazol-9-yl)cyclohexanol | ADD (1S,2S,6R)-2-azido-6-(3,6-difluoro-9H-carbazol-9-yl)cyclohexanol |
| 1 | ADD THF | ADD THF |
| 2 | SETTEMPERATURE 0 °C | SETTEMPERATURE 0 °C |
| 3 | ADD triphenylphosphine | ADD triphenylphosphine |
| 4 | ADD water | ADD water |
| 5 | STIR for 86400 s at 25 °C | STIR for 86400 s at 25 °C |
| 6 | CONCENTRATE | CONCENTRATE |
| 7 | ADD dichloromethane | ADD dichloromethane |
| 8 | PURIFY | PURIFY |
| 9 | COLLECTLAYER organic | YIELD (1R,2S,6R)-2-amino-6-(3,6-difluoro-9H-carbazol-9-yl)cyclohexanol |
| 10 | CONCENTRATE |  |
| 11 | DRYSOLUTION over toluene |  |
| 12 | YIELD (1R,2S,6R)-2-amino-6-(3,6-difluoro-9H-carbazol-9-yl)cyclohexanol |  |

---

```
Reaction no 177
```

Generated by the Chemistry Development Kit (http://github.com/cdk)

|  | A | B |
| --- | --- | --- |
| 0 | ADD 3,4-diaminobenzoic acid | ADD 3,4-diaminobenzoic acid |
| 1 | ADD TFA | ADD TFA |
| 2 | REFLUX for 86400 s | STIR for 86400 s at 60 °C |
| 3 | CONCENTRATE | CONCENTRATE |
| 4 | ADD water | PURIFY |
| 5 | FILTER keep precipitate | YIELD 2-(trifluoromethyl)-1H-benzimidazole-6-carboxylic acid |
| 6 | DRYSOLID under vacuum |  |
| 7 | YIELD 2-(trifluoromethyl)-1H-benzimidazole-6-carboxylic acid |  |

---

```
Reaction no 178
```

Generated by the Chemistry Development Kit (http://github.com/cdk)

|  | A | B |
| --- | --- | --- |
| 0 | ADD 5-bromo-3-fluoropyridin-2-amine | ADD 5-bromo-3-fluoropyridin-2-amine |
| 1 | ADD pyridine | ADD pyridine |
| 2 | ADD 2-chlorobenzenesulfonyl chloride | ADD 2-chlorobenzenesulfonyl chloride |
| 3 | PURIFY | STIR for 86400 s at 60 °C |
| 4 | YIELD N-(5-bromo-3-fluoropyridin-2-yl)-2-chlorobenzenesulfonamide | CONCENTRATE |
| 5 |  | PURIFY |
| 6 |  | YIELD N-(5-bromo-3-fluoropyridin-2-yl)-2-chlorobenzenesulfonamide |

---

```
Reaction no 179
```

Generated by the Chemistry Development Kit (http://github.com/cdk)

|  | A | B |
| --- | --- | --- |
| 0 | ADD Ethyl 2-(3,5-dimethyl-1H-pyrazol-4-yl)acetate | ADD Ethyl 2-(3,5-dimethyl-1H-pyrazol-4-yl)acetate |
| 1 | ADD 1-(Bromomethyl)-2-chloro-4-nitro-benzene | ADD 1-(Bromomethyl)-2-chloro-4-nitro-benzene |
| 2 | ADD K2CO3 | ADD acetonitrile |
| 3 | ADD acetonitrile | ADD K2CO3 |
| 4 | STIR for 86400 s at 25 °C | STIR for 86400 s at 25 °C |
| 5 | FILTER keep filtrate | CONCENTRATE |
| 6 | CONCENTRATE | ADD dichloromethane / water |
| 7 | PURIFY | COLLECTLAYER organic |
| 8 | YIELD [3,5-dimethyl-1-(2-chloro-4-nitro-benzyl)-1H-pyrazol-4-yl]-acetic acid ethyl ester | DRYSOLUTION over Na2SO4 |
| 9 |  | CONCENTRATE |
| 10 |  | YIELD [3,5-dimethyl-1-(2-chloro-4-nitro-benzyl)-1H-pyrazol-4-yl]-acetic acid ethyl ester |

---

```
Reaction no 180
```

Generated by the Chemistry Development Kit (http://github.com/cdk)

|  | A | B |
| --- | --- | --- |
| 0 | ADD 2-fluorobenzaldehyde | ADD 2-fluorobenzaldehyde |
| 1 | ADD tert-butylamine | ADD benzene |
| 2 | ADD benzene | ADD tert-butylamine |
| 3 | REFLUX for 86400 s with Dean-Stark apparatus | REFLUX for 86400 s |
| 4 | CONCENTRATE | SETTEMPERATURE 25 °C |
| 5 | YIELD tert-Butyl-(2-fluoro-benzylidene)-amine | CONCENTRATE |
| 6 |  | YIELD tert-Butyl-(2-fluoro-benzylidene)-amine |

---

```
Reaction no 181
```

Generated by the Chemistry Development Kit (http://github.com/cdk)

|  | A | B |
| --- | --- | --- |
| 0 | ADD methyl 3-bromo-4-hydroxybenzoate | ADD methyl 3-bromo-4-hydroxybenzoate |
| 1 | ADD CuCN | ADD DMF |
| 2 | ADD CuI | ADD CuI |
| 3 | ADD DMF | ADD CuCN |
| 4 | STIR for 86400 s at 100 °C | STIR for 86400 s at 100 °C |
| 5 | ADD water | SETTEMPERATURE 100 °C |
| 6 | EXTRACT with ethyl acetate | ADD water |
| 7 | COLLECTLAYER organic | EXTRACT with dichloromethane |
| 8 | WASH with brine | COLLECTLAYER organic |
| 9 | DRYSOLUTION over sodium sulfate | DRYSOLUTION over sodium sulfate |
| 10 | CONCENTRATE | CONCENTRATE |
| 11 | PURIFY | PURIFY |
| 12 | YIELD Methyl 3-cyano-4-hydroxybenzoate | YIELD Methyl 3-cyano-4-hydroxybenzoate |

---

```
Reaction no 182
```

Generated by the Chemistry Development Kit (http://github.com/cdk)

|  | A | B |
| --- | --- | --- |
| 0 | ADD aniline | ADD aniline |
| 1 | ADD acetonitrile | ADD K2CO3 |
| 2 | ADD K2CO3 at 25 °C | ADD acetonitrile |
| 3 | ADD cyclopropanecarbonyl chloride at 25 °C | ADD cyclopropanecarbonyl chloride at 0 °C |
| 4 | STIR for 3600 s at 25 °C | STIR for 3600 s at 25 °C |
| 5 | CONCENTRATE | QUENCH with water |
| 6 | ADD water | EXTRACT with ethyl acetate |
| 7 | EXTRACT with dichloromethane | COLLECTLAYER organic |
| 8 | COLLECTLAYER organic | DRYSOLUTION over Na2SO4 |
| 9 | WASH with water | FILTER keep filtrate |
| 10 | DRYSOLUTION over MgSO4 | CONCENTRATE |
| 11 | FILTER keep filtrate | YIELD N-phenylcyclopropanamide |
| 12 | CONCENTRATE |  |
| 13 | RECRYSTALLIZE from ethyl acetate / hexanes |  |
| 14 | YIELD N-phenylcyclopropanamide |  |

---

```
Reaction no 183
```

Generated by the Chemistry Development Kit (http://github.com/cdk)

|  | A | B |
| --- | --- | --- |
| 0 | ADD 4-chloro-1-(5,6-dihydropyrazolo[4,3-b][1,4]oxazin-7(1H)-yl)butan-1-one | ADD 4-chloro-1-(5,6-dihydropyrazolo[4,3-b][1,4]oxazin-7(1H)-yl)butan-1-one |
| 1 | ADD Cs2CO3 | ADD DMF |
| 2 | ADD DMF | ADD Cs2CO3 |
| 3 | STIR for 86400 s at 25 °C | STIR for 28800 s at 25 °C |
| 4 | FILTER keep filtrate | CONCENTRATE |
| 5 | CONCENTRATE | PURIFY |
| 6 | PURIFY | YIELD 4,5,8,9-tetrahydro-3-oxa-1,5a,9a-triazabenzo[cd]azulen-6(7H)-one |
| 7 | YIELD 4,5,8,9-tetrahydro-3-oxa-1,5a,9a-triazabenzo[cd]azulen-6(7H)-one |  |

---

```
Reaction no 184
```

Generated by the Chemistry Development Kit (http://github.com/cdk)

|  | A | B |
| --- | --- | --- |
| 0 | ADD 2-[(4-nitrophenyl)sulfonyl]benzonitrile | ADD 2-[(4-nitrophenyl)sulfonyl]benzonitrile |
| 1 | ADD ethanol | ADD ethanol |
| 2 | ADD iron | ADD iron |
| 3 | ADD HCl | REFLUX |
| 4 | REFLUX for 3600 s | MAKESOLUTION with HCl and ethanol |
| 5 | FILTER keep filtrate | ADD SLN dropwise |
| 6 | CONCENTRATE | REFLUX for 28800 s |
| 7 | PURIFY | ADD ethanol |
| 8 | YIELD 4-[(2-Cyanophenyl)sulfonyl]benzeneamine | FILTER keep filtrate |
| 9 |  | CONCENTRATE |
| 10 |  | STIR for 86400 s |
| 11 |  | FILTER keep filtrate |
| 12 |  | YIELD 4-[(2-Cyanophenyl)sulfonyl]benzeneamine |

---

```
Reaction no 185
```

Generated by the Chemistry Development Kit (http://github.com/cdk)

|  | A | B |
| --- | --- | --- |
| 0 | ADD 3-aminopyrrolidine | ADD 3-aminopyrrolidine |
| 1 | ADD toluene | ADD toluene |
| 2 | ADD benzaldehyde dropwise | ADD benzaldehyde dropwise |
| 3 | STIR for 28800 s at 25 °C | STIR for 3600 s at 25 °C |
| 4 | CONCENTRATE | CONCENTRATE |
| 5 | ADD toluene | YIELD N-(phenylmethylene)pyrrolidine-3-amine |
| 6 | YIELD N-(phenylmethylene)pyrrolidine-3-amine |  |

---

```
Reaction no 186
```

Generated by the Chemistry Development Kit (http://github.com/cdk)

|  | A | B |
| --- | --- | --- |
| 0 | ADD 1,3,8-triaza-3-butyl-7,7,9,9-tetramethyl-8-nitroso-spiro[4.5]decane-2,4-dione | ADD 1,3,8-triaza-3-butyl-7,7,9,9-tetramethyl-8-nitroso-spiro[4.5]decane-2,4-dione |
| 1 | ADD zinc | ADD acetic acid |
| 2 | ADD water | ADD zinc |
| 3 | ADD acetic acid | STIR for 86400 s at 25 °C |
| 4 | STIR for 3600 s at 60 °C | ADD water |
| 5 | SETTEMPERATURE 25 °C | PH with NaOH to pH basic |
| 6 | ADD NaOH | EXTRACT with dichloromethane |
| 7 | EXTRACT with benzene | COLLECTLAYER organic |
| 8 | COLLECTLAYER organic | DRYSOLUTION over Na2SO4 |
| 9 | DRYSOLUTION over sodium sulfate | CONCENTRATE |
| 10 | CONCENTRATE | YIELD 8-amino-1,3,8-triaza-3-butyl-7,7,9,9-tetramethyl-spiro[4.5]-decane-2,4-dione |
| 11 | RECRYSTALLIZE from benzene |  |
| 12 | YIELD 8-amino-1,3,8-triaza-3-butyl-7,7,9,9-tetramethyl-spiro[4.5]-decane-2,4-dione |  |

---

```
Reaction no 187
```

Generated by the Chemistry Development Kit (http://github.com/cdk)

|  | A | B |
| --- | --- | --- |
| 0 | ADD N-iodosuccinimide | ADD 1-methoxy-3,4-methylenedioxybenzene |
| 1 | ADD 1-methoxy-3,4-methylenedioxybenzene | ADD acetonitrile |
| 2 | ADD acetonitrile | ADD N-iodosuccinimide |
| 3 | ADD TFA | ADD TFA |
| 4 | STIR for 86400 s at 25 °C | STIR for 86400 s at 25 °C |
| 5 | CONCENTRATE | CONCENTRATE |
| 6 | PURIFY | PURIFY |
| 7 | YIELD 5-Iodo-6-methoxybenzo[d][1,3]dioxole | YIELD 5-Iodo-6-methoxybenzo[d][1,3]dioxole |

---

```
Reaction no 188
```

Generated by the Chemistry Development Kit (http://github.com/cdk)

|  | A | B |
| --- | --- | --- |
| 0 | ADD methyl 3-amino-4-chloro-2-[(4-hydroxybutyl)amino]benzoate | ADD methyl 3-amino-4-chloro-2-[(4-hydroxybutyl)amino]benzoate |
| 1 | ADD 2-Chloro-1-isothiocyanato-4-methoxybenzene | ADD 2-Chloro-1-isothiocyanato-4-methoxybenzene |
| 2 | ADD THF | ADD THF |
| 3 | STIR for 86400 s at 60 °C | STIR for 86400 s at 60 °C |
| 4 | CONCENTRATE | CONCENTRATE |
| 5 | PURIFY | PURIFY |
| 6 | CONCENTRATE | YIELD methyl 4-chloro-3-{[(2-chloro-4-methoxyphenyl)carbamothioyl]amino}-2-[(4-hydroxybutyl)amino]benzoate |
| 7 | YIELD methyl 4-chloro-3-{[(2-chloro-4-methoxyphenyl)carbamothioyl]amino}-2-[(4-hydroxybutyl)amino]benzoate |  |

---

```
Reaction no 189
```

Generated by the Chemistry Development Kit (http://github.com/cdk)

|  | A | B |
| --- | --- | --- |
| 0 | ADD 5-amino-2-fluoro-benzamide | ADD 5-amino-2-fluoro-benzamide |
| 1 | MAKESOLUTION with DIPEA and THF | ADD DIPEA |
| 2 | ADD SLN | ADD dichloromethane |
| 3 | MAKESOLUTION with 2-cyclopropyl-3-fluoro-5-[2-methoxy-4-(trifluoromethoxy)phenoxy]pyridine-4-carbonyl chloride and dichloromethane | ADD THF |
| 4 | ADD SLN dropwise at 0 °C | ADD 2-cyclopropyl-3-fluoro-5-[2-methoxy-4-(trifluoromethoxy)phenoxy]pyridine-4-carbonyl chloride |
| 5 | SETTEMPERATURE 25 °C | STIR for 86400 s at 25 °C |
| 6 | STIR for 86400 s | CONCENTRATE |
| 7 | QUENCH with water | ADD DMSO |
| 8 | COLLECTLAYER aqueous | PURIFY |
| 9 | EXTRACT with dichloromethane | YIELD N-(3-carbamoyl-4-fluoro-phenyl)-2-cyclopropyl-3-fluoro-5-[2-methoxy-4-(trifluoromethoxy)phenoxy]pyridine-4-carboxamide |
| 10 | COLLECTLAYER organic |  |
| 11 | DRYSOLUTION over MgSO4 |  |
| 12 | FILTER keep filtrate |  |
| 13 | CONCENTRATE |  |
| 14 | ADD DMF / methanol |  |
| 15 | FILTER |  |
| 16 | PURIFY |  |
| 17 | YIELD N-(3-carbamoyl-4-fluoro-phenyl)-2-cyclopropyl-3-fluoro-5-[2-methoxy-4-(trifluoromethoxy)phenoxy]pyridine-4-carboxamide |  |

---

```
Reaction no 190
```

Generated by the Chemistry Development Kit (http://github.com/cdk)

|  | A | B |
| --- | --- | --- |
| 0 | ADD tert-butyl 4-oxoazepane-1-carboxylate | ADD tert-butyl 4-oxoazepane-1-carboxylate |
| 1 | ADD dichloromethane | ADD dichloromethane |
| 2 | ADD TFA | ADD TFA dropwise at 25 °C |
| 3 | STIR for 3600 s at 25 °C | STIR for 28800 s |
| 4 | CONCENTRATE | CONCENTRATE |
| 5 | DRYSOLID under vacuum | ADD DMF |
| 6 | ADD dichloromethane | ADD K2CO3 |
| 7 | ADD K2CO3 | ADD benzyl bromide |
| 8 | ADD benzyl bromide | STIR for 28800 s at 60 °C |
| 9 | STIR for 86400 s at 25 °C | SETTEMPERATURE 25 °C |
| 10 | FILTER keep filtrate | FILTER keep filtrate |
| 11 | CONCENTRATE | CONCENTRATE |
| 12 | PURIFY | PURIFY |
| 13 | YIELD 1-benzylazepan-4-one | YIELD 1-benzylazepan-4-one |

---

```
Reaction no 191
```

Generated by the Chemistry Development Kit (http://github.com/cdk)

|  | A | B |
| --- | --- | --- |
| 0 | ADD 6-bromo-1H-indole-3-carboxylic acid methyl ester | ADD 6-bromo-1H-indole-3-carboxylic acid methyl ester |
| 1 | ADD K2CO3 | ADD acetonitrile |
| 2 | ADD acetonitrile | ADD K2CO3 |
| 3 | ADD methyl iodide at 25 °C | ADD methyl iodide |
| 4 | STIR for 604800 s at 25 °C | STIR for 86400 s at 25 °C |
| 5 | CONCENTRATE | FILTER keep filtrate |
| 6 | ADD water | CONCENTRATE |
| 7 | COLLECTLAYER organic | PURIFY |
| 8 | EXTRACT with ethyl acetate | YIELD 6-Bromo-1-methyl-1H-indole-3-carboxylic acid methyl ester |
| 9 | COLLECTLAYER organic |  |
| 10 | DRYSOLUTION over magnesium sulfate |  |
| 11 | CONCENTRATE |  |
| 12 | PURIFY |  |
| 13 | YIELD 6-Bromo-1-methyl-1H-indole-3-carboxylic acid methyl ester |  |

---

```
Reaction no 192
```

Generated by the Chemistry Development Kit (http://github.com/cdk)

|  | A | B |
| --- | --- | --- |
| 0 | ADD cyclopropylamine | ADD cyclopropylamine |
| 1 | ADD DMA | ADD DIPEA |
| 2 | ADD DIPEA | ADD DMA |
| 3 | ADD 3-(6-amino-5-(3-methylisoxazol-5-yl)pyridin-3-yl)-4-methylbenzene-1-sulfonyl chloride | ADD 3-(6-amino-5-(3-methylisoxazol-5-yl)pyridin-3-yl)-4-methylbenzene-1-sulfonyl chloride |
| 4 | STIR for 86400 s at 25 °C | STIR for 86400 s at 25 °C |
| 5 | FILTER keep precipitate | PARTITION with dichloromethane and sodium bicarbonate |
| 6 | PURIFY | PHASESEPARATION |
| 7 | YIELD 3-(6-Amino-5-(3-methylisoxazol-5-yl)pyridin-3-yl)-N-cyclopropyl-4-methylbenzenesulfonamide | PURIFY |
| 8 |  | WASH with dichloromethane |
| 9 |  | COLLECTLAYER organic |
| 10 |  | CONCENTRATE |
| 11 |  | PURIFY |
| 12 |  | COLLECTLAYER organic |
| 13 |  | CONCENTRATE |
| 14 |  | DRYSOLID under vacuum |
| 15 |  | TRITURATE with methanol |
| 16 |  | DRYSOLID under vacuum |
| 17 |  | YIELD 3-(6-Amino-5-(3-methylisoxazol-5-yl)pyridin-3-yl)-N-cyclopropyl-4-methylbenzenesulfonamide |

---

```
Reaction no 193
```

Generated by the Chemistry Development Kit (http://github.com/cdk)

|  | A | B |
| --- | --- | --- |
| 0 | ADD 2-(4-chloro-benzenesulfonyl)-2-methyl-propionic acid 2-(5-tert-butyl-2-methyl-2H-pyrazol-3-yl)-2-oxo-ethyl ester | ADD 2-(4-chloro-benzenesulfonyl)-2-methyl-propionic acid 2-(5-tert-butyl-2-methyl-2H-pyrazol-3-yl)-2-oxo-ethyl ester |
| 1 | ADD xylene | ADD acetamide |
| 2 | ADD acetamide | ADD xylene |
| 3 | ADD BF3.Et2O | ADD BF3.Et2O |
| 4 | MICROWAVE for 28800 s at 100 °C | REFLUX for 86400 s |
| 5 | CONCENTRATE | CONCENTRATE |
| 6 | ADD dichloromethane | PURIFY |
| 7 | WASH with sodium bicarbonate | YIELD 4-(5-tert-butyl-2-methyl-2H-pyrazol-3-yl)-2-[1-(4-chlorobenzenesulfonyl)-1-methyl-ethyl]-oxazole |
| 8 | DRYSOLUTION over Na2SO4 |  |
| 9 | FILTER keep filtrate |  |
| 10 | CONCENTRATE |  |
| 11 | PURIFY |  |
| 12 | YIELD 4-(5-tert-butyl-2-methyl-2H-pyrazol-3-yl)-2-[1-(4-chlorobenzenesulfonyl)-1-methyl-ethyl]-oxazole |  |

---

```
Reaction no 194
```

Generated by the Chemistry Development Kit (http://github.com/cdk)

|  | A | B |
| --- | --- | --- |
| 0 | ADD methyl 2-({4-[(tert-butoxycarbonyl)amino]phenyl}sulfonyl)-4-(4-oxo-1,2,3-benzotriazin-3(4H)-yl)butanoate | ADD methyl 2-({4-[(tert-butoxycarbonyl)amino]phenyl}sulfonyl)-4-(4-oxo-1,2,3-benzotriazin-3(4H)-yl)butanoate |
| 1 | ADD dichloromethane | ADD dichloromethane |
| 2 | ADD TFA | ADD TFA at 0 °C |
| 3 | STIR for 3600 s at 25 °C | STIR for 28800 s at 25 °C |
| 4 | CONCENTRATE | CONCENTRATE |
| 5 | YIELD methyl 2-[(4-aminophenyl)sulfonyl]-4-(4-oxo-1,2,3-benzotriazin-3(4H)-yl)butanoate | ADD sodium bicarbonate |
| 6 |  | EXTRACT with ethyl acetate |
| 7 |  | WASH with water |
| 8 |  | COLLECTLAYER organic |
| 9 |  | DRYSOLUTION over Na2SO4 |
| 10 |  | CONCENTRATE |
| 11 |  | YIELD methyl 2-[(4-aminophenyl)sulfonyl]-4-(4-oxo-1,2,3-benzotriazin-3(4H)-yl)butanoate |

---

```
Reaction no 195
```

Generated by the Chemistry Development Kit (http://github.com/cdk)

|  | A | B |
| --- | --- | --- |
| 0 | ADD tert-butyl 2-oxopiperidine-1-carboxylate | ADD tert-butyl 2-oxopiperidine-1-carboxylate |
| 1 | ADD cycloheptylamine | ADD dichloromethane |
| 2 | ADD dichloromethane | ADD cycloheptylamine |
| 3 | ADD sodium triacetoxyborohydride | ADD sodium triacetoxyborohydride |
| 4 | STIR for 86400 s at 25 °C | STIR for 86400 s at 25 °C |
| 5 | ADD NaOH | ADD NaOH |
| 6 | EXTRACT with ethyl acetate | EXTRACT with ethyl acetate |
| 7 | COLLECTLAYER organic | DRYSOLUTION over MgSO4 |
| 8 | WASH with brine | CONCENTRATE |
| 9 | DRYSOLUTION over magnesium sulfate | YIELD 4-cycloheptylamino-piperidine-1-carboxylic acid tert-butyl ester |
| 10 | CONCENTRATE |  |
| 11 | PURIFY |  |
| 12 | YIELD 4-cycloheptylamino-piperidine-1-carboxylic acid tert-butyl ester |  |

---

```
Reaction no 196
```

Generated by the Chemistry Development Kit (http://github.com/cdk)

|  | A | B |
| --- | --- | --- |
| 0 | ADD 5-(chloromethyl)-3-methyl-1H-pyrazole | ADD 5-(chloromethyl)-3-methyl-1H-pyrazole |
| 1 | ADD acetonitrile | ADD water |
| 2 | MAKESOLUTION with KCN and water | ADD acetonitrile |
| 3 | ADD SLN | ADD KCN at 25 °C |
| 4 | STIR for 86400 s at 25 °C | STIR for 86400 s at 60 °C |
| 5 | ADD water | EXTRACT with ethyl acetate |
| 6 | EXTRACT with ethyl acetate | COLLECTLAYER organic |
| 7 | COLLECTLAYER organic | DRYSOLUTION over sodium sulfate |
| 8 | WASH with brine | CONCENTRATE |
| 9 | DRYSOLUTION over Na2SO4 | PURIFY |
| 10 | CONCENTRATE | YIELD 2-(3-methyl-1H-pyrazol-5-yl)acetonitrile |
| 11 | PURIFY |  |
| 12 | YIELD 2-(3-methyl-1H-pyrazol-5-yl)acetonitrile |  |

---

```
Reaction no 197
```

Generated by the Chemistry Development Kit (http://github.com/cdk)

|  | A | B |
| --- | --- | --- |
| 0 | ADD (2-chloropyridin-4-yl)methanol | ADD (2-chloropyridin-4-yl)methanol |
| 1 | ADD tert-butyldimethylsilyl chloride | ADD DMF |
| 2 | ADD imidazole | ADD imidazole |
| 3 | ADD DMF | ADD tert-butyldimethylsilyl chloride |
| 4 | STIR for 86400 s at 25 °C | STIR for 3600 s at 25 °C |
| 5 | ADD brine | ADD brine |
| 6 | EXTRACT with ethyl acetate | EXTRACT with ethyl acetate |
| 7 | COLLECTLAYER organic | WASH with brine |
| 8 | DRYSOLUTION over Na2SO4 | DRYSOLUTION over sodium sulfate |
| 9 | FILTER keep filtrate | PURIFY |
| 10 | CONCENTRATE | YIELD 4-[[(tert-butyldimethylsilyl)oxy]methyl]-2-chloropyridine |
| 11 | PURIFY |  |
| 12 | YIELD 4-[[(tert-butyldimethylsilyl)oxy]methyl]-2-chloropyridine |  |

---

```
Reaction no 198
```

Generated by the Chemistry Development Kit (http://github.com/cdk)

|  | A | B |
| --- | --- | --- |
| 0 | ADD 1-(6-{4-[3-(4-fluoro-phenyl)-5-trifluoromethyl-isoxazol-4-yl]-imidazol-1-yl}-pyridin-3-yl)-ethanone | ADD 1-(6-{4-[3-(4-fluoro-phenyl)-5-trifluoromethyl-isoxazol-4-yl]-imidazol-1-yl}-pyridin-3-yl)-ethanone |
| 1 | ADD THF | ADD THF |
| 2 | ADD methylmagnesium bromide at 25 °C | ADD methylmagnesium bromide at 0 °C |
| 3 | STIR for 86400 s | STIR for 3600 s at 0 °C |
| 4 | ADD HCl | ADD HCl |
| 5 | EXTRACT with ethyl acetate | EXTRACT with ethyl acetate |
| 6 | COLLECTLAYER organic | COLLECTLAYER organic |
| 7 | DRYSOLUTION over Na2SO4 | DRYSOLUTION over MgSO4 |
| 8 | CONCENTRATE | FILTER keep filtrate |
| 9 | PURIFY | CONCENTRATE |
| 10 | YIELD 2-(6-{4-[3-(4-Fluoro-phenyl)-5-trifluoromethyl-isoxazol-4-yl]-imidazol-1-yl}-pyridin-3-yl)-propan-2-ol | PURIFY |
| 11 |  | YIELD 2-(6-{4-[3-(4-Fluoro-phenyl)-5-trifluoromethyl-isoxazol-4-yl]-imidazol-1-yl}-pyridin-3-yl)-propan-2-ol |

---

```
Reaction no 199
```

Generated by the Chemistry Development Kit (http://github.com/cdk)

|  | A | B |
| --- | --- | --- |
| 0 | ADD 4-chloro-3-[(2-methoxy-7-oxo-7,8-dihydro-pyrido[2,3-d]pyrimidine-6-carbonyl)-amino]-benzoic acid | ADD 4-chloro-3-[(2-methoxy-7-oxo-7,8-dihydro-pyrido[2,3-d]pyrimidine-6-carbonyl)-amino]-benzoic acid |
| 1 | ADD HATU | ADD HATU |
| 2 | ADD DMF | ADD DMF |
| 3 | ADD DIPEA | ADD DIPEA |
| 4 | STIR for 3600 s at 25 °C | STIR for 3600 s at 25 °C |
| 5 | ADD 3-(trifluoromethyl)benzylamine | ADD 3-(trifluoromethyl)benzylamine |
| 6 | STIR for 86400 s | STIR for 86400 s |
| 7 | PURIFY | FILTER keep precipitate |
| 8 | YIELD 2-methoxy-7-oxo-7,8-dihydro-pyrido[2,3-d]pyrimidine-6-carboxylic acid [2-chloro-5-(3-trifluoromethyl-benzylcarbamoyl)-phenyl]amide | WASH with methanol |
| 9 |  | DRYSOLID under vacuum |
| 10 |  | YIELD 2-methoxy-7-oxo-7,8-dihydro-pyrido[2,3-d]pyrimidine-6-carboxylic acid [2-chloro-5-(3-trifluoromethyl-benzylcarbamoyl)-phenyl]amide |

---

```
Reaction no 200
```

Generated by the Chemistry Development Kit (http://github.com/cdk)

|  | A | B |
| --- | --- | --- |
| 0 | ADD (2-chloro-9-methyl-6-morpholin-4-yl-9H-purin-8-yl)acetaldehyde | ADD (2-chloro-9-methyl-6-morpholin-4-yl-9H-purin-8-yl)acetaldehyde |
| 1 | ADD 1,2-dichloroethane | ADD 1,2-dichloroethane |
| 2 | ADD 2-(piperidin-4-yl)propan-2-ol | ADD 2-(piperidin-4-yl)propan-2-ol |
| 3 | ADD trimethyl orthoformate | ADD trimethyl orthoformate |
| 4 | ADD acetic acid | ADD acetic acid |
| 5 | STIR for 28800 s at 25 °C | STIR for 28800 s at 25 °C |
| 6 | ADD sodium triacetoxyborohydride | ADD sodium triacetoxyborohydride |
| 7 | STIR for 86400 s | STIR for 3600 s |
| 8 | WASH with methanol | WASH with methanol |
| 9 | WASH with ammonia / methanol | WASH with ammonia / methanol |
| 10 | PURIFY | PURIFY |
| 11 | YIELD 2-{1-[2-(2-chloro-9-methyl-6-morpholin-4-yl-9H-purin-8-yl)ethyl]-piperidin-4-yl}propan-2-ol | YIELD 2-{1-[2-(2-chloro-9-methyl-6-morpholin-4-yl-9H-purin-8-yl)ethyl]-piperidin-4-yl}propan-2-ol |

---

```
Reaction no 201
```

Generated by the Chemistry Development Kit (http://github.com/cdk)

|  | A | B |
| --- | --- | --- |
| 0 | ADD 4-pentylcyclohexyl-p-(3-bromopropyl)phenyl ketone | ADD 4-pentylcyclohexyl-p-(3-bromopropyl)phenyl ketone |
| 1 | ADD methanol | ADD sodium methoxide methanol |
| 2 | STIR | REFLUX for 86400 s |
| 3 | ADD sodium methoxide methanol | SETTEMPERATURE 25 °C |
| 4 | ADD sodium | FILTER keep filtrate |
| 5 | ADD methanol | CONCENTRATE |
| 6 | REFLUX for 28800 s | PURIFY |
| 7 | CONCENTRATE | YIELD 4-pentylcyclohexyl-p-(3-methoxypropyl)phenyl ketone |
| 8 | ADD water |  |
| 9 | COLLECTLAYER organic |  |
| 10 | EXTRACT with benzene |  |
| 11 | CONCENTRATE |  |
| 12 | CONCENTRATE |  |
| 13 | YIELD 4-pentylcyclohexyl-p-(3-methoxypropyl)phenyl ketone |  |

---

```
Reaction no 202
```

Generated by the Chemistry Development Kit (http://github.com/cdk)

|  | A | B |
| --- | --- | --- |
| 0 | ADD 9,10-difluoro-2,3-dihydro-3-methyl-7-oxo-7H-pyrido[1,2,3-de]-1,4-benzoxazine-6-carboxylic acid | ADD 9,10-difluoro-2,3-dihydro-3-methyl-7-oxo-7H-pyrido[1,2,3-de]-1,4-benzoxazine-6-carboxylic acid |
| 1 | ADD thiomorpholine | ADD thiomorpholine |
| 2 | ADD pyridine | ADD pyridine |
| 3 | REFLUX for 86400 s | REFLUX for 86400 s |
| 4 | CONCENTRATE | CONCENTRATE |
| 5 | TRITURATE with methanol | RECRYSTALLIZE from DMF |
| 6 | FILTER keep precipitate | YIELD 9-Fluoro-2,3-dihydro-3-methyl-7-oxo-10-(4-thiomorpholinyl)-7H-pyrido[1,2,3-de]-1,4-benzoxazine-6-carboxylic acid |
| 7 | YIELD 9-Fluoro-2,3-dihydro-3-methyl-7-oxo-10-(4-thiomorpholinyl)-7H-pyrido[1,2,3-de]-1,4-benzoxazine-6-carboxylic acid |  |

---

```
Reaction no 203
```

Generated by the Chemistry Development Kit (http://github.com/cdk)

|  | A | B |
| --- | --- | --- |
| 0 | ADD (R)-tert-butyl 3-((R)-1-(3-chlorophenyl)-1-(2-hydroxyethoxy)ethyl)piperidine-1-carboxylate | ADD (R)-tert-butyl 3-((R)-1-(3-chlorophenyl)-1-(2-hydroxyethoxy)ethyl)piperidine-1-carboxylate |
| 1 | ADD phthalimide | ADD phthalimide |
| 2 | ADD triphenylphosphine | ADD triphenylphosphine |
| 3 | ADD THF | ADD DIAD |
| 4 | ADD DIAD | ADD THF |
| 5 | STIR for 86400 s at 25 °C | STIR for 86400 s at 25 °C |
| 6 | CONCENTRATE | CONCENTRATE |
| 7 | PURIFY | PURIFY |
| 8 | YIELD (R)-tert-butyl 3-((R)-1-(3-chlorophenyl)-1-(2-(1,3-dioxoisoindolin-2-yl)ethoxy)ethyl)piperidine-1-carboxylate | YIELD (R)-tert-butyl 3-((R)-1-(3-chlorophenyl)-1-(2-(1,3-dioxoisoindolin-2-yl)ethoxy)ethyl)piperidine-1-carboxylate |

---

```
Reaction no 204
```

Generated by the Chemistry Development Kit (http://github.com/cdk)

|  | A | B |
| --- | --- | --- |
| 0 | ADD 2-methyl-1,6-naphthyridine | ADD 2-methyl-1,6-naphthyridine |
| 1 | ADD carbon tetrachloride | ADD N-chlorosuccinimide |
| 2 | ADD N-chlorosuccinimide | ADD benzoyl peroxide |
| 3 | ADD benzoyl peroxide | ADD carbon tetrachloride |
| 4 | REFLUX for 28800 s | REFLUX for 86400 s |
| 5 | SETTEMPERATURE 25 °C | SETTEMPERATURE 25 °C |
| 6 | CONCENTRATE | FILTER keep filtrate |
| 7 | PURIFY | CONCENTRATE |
| 8 | YIELD 2-Chloromethyl-1,6-naphthyridine | PURIFY |
| 9 |  | YIELD 2-Chloromethyl-1,6-naphthyridine |

---

```
Reaction no 205
```

Generated by the Chemistry Development Kit (http://github.com/cdk)

|  | A | B |
| --- | --- | --- |
| 0 | ADD methyl 6-O-tert-butyldiphenylsilyl-2,3,4-tri-O-benzyl-β-D-glucopyranoside | ADD methyl 6-O-tert-butyldiphenylsilyl-2,3,4-tri-O-benzyl-β-D-glucopyranoside |
| 1 | ADD dichloromethane | ADD dichloromethane |
| 2 | ADD 2,6-di-tert-butyl-4-methylpyridine at 25 °C | ADD 2,6-di-tert-butyl-4-methylpyridine at -70 °C |
| 3 | ADD trifluoromethanesulfonic anhydride at 25 °C | ADD trifluoromethanesulfonic anhydride at -70 °C |
| 4 | STIR for 86400 s at 25 °C | STIR for 600 s at -70 °C |
| 5 | QUENCH with methanol | STIR for 600 s at 25 °C |
| 6 | CONCENTRATE | ADD Methyl 2,3,4-tri-O-benzyl-6-O-trifluoromethylsulfonyl-β-D-glucopyranoside |
| 7 | PURIFY | EXTRACT with ethyl acetate |
| 8 | YIELD Methyl 2,3,4-tri-O-benzyl-6-O-trifluoromethylsulfonyl-β-D-glucopyranoside | COLLECTLAYER organic |
| 9 |  | WASH with NaHCO3 |
| 10 |  | WASH with sodium chloride |
| 11 |  | DRYSOLUTION over magnesium sulfate |
| 12 |  | CONCENTRATE |

---

```
Reaction no 206
```

Generated by the Chemistry Development Kit (http://github.com/cdk)

|  | A | B |
| --- | --- | --- |
| 0 | ADD 3-Pyridin-2-yl-isoxazole-4-carboxylic acid | ADD 3-Pyridin-2-yl-isoxazole-4-carboxylic acid |
| 1 | ADD triethylamine | ADD THF |
| 2 | ADD THF | ADD triethylamine at 0 °C |
| 3 | ADD ethyl chloroformate at 0 °C | MAKESOLUTION with ethyl chloroformate and THF |
| 4 | STIR for 3600 s at 0 °C | ADD SLN at 0 °C |
| 5 | FILTER keep filtrate | WAIT for 3600 s |
| 6 | MAKESOLUTION with sodium borohydride and water | FILTER keep precipitate |
| 7 | ADD SLN | SETTEMPERATURE 0 °C |
| 8 | STIR for 3600 s at 25 °C | MAKESOLUTION with sodium borohydride and water |
| 9 | ADD NaOH | ADD SLN at 0 °C over 600 s |
| 10 | EXTRACT with ethyl acetate | STIR for 3600 s at 25 °C |
| 11 | COLLECTLAYER organic | ADD NaOH |
| 12 | WASH with brine | EXTRACT with ether |
| 13 | DRYSOLUTION over Na2SO4 | COLLECTLAYER organic |
| 14 | CONCENTRATE | WASH with water |
| 15 | PURIFY | WASH with brine |
| 16 | YIELD (3-pyridin-2-yl-isoxazol-4-yl)-methanol | DRYSOLUTION over sodium sulfate |
| 17 |  | CONCENTRATE |
| 18 |  | YIELD (3-pyridin-2-yl-isoxazol-4-yl)-methanol |

---

```
Reaction no 207
```

Generated by the Chemistry Development Kit (http://github.com/cdk)

|  | A | B |
| --- | --- | --- |
| 0 | ADD 4-[(Pyridazin-4-ylamino)-methyl]-piperidine | ADD 4-[(Pyridazin-4-ylamino)-methyl]-piperidine |
| 1 | ADD DMF | ADD DMF |
| 2 | ADD N-(benzyloxycarbonyloxy)succinimide | ADD N-(benzyloxycarbonyloxy)succinimide |
| 3 | STIR for 3600 s at 25 °C | STIR for 3600 s at 25 °C |
| 4 | CONCENTRATE | CONCENTRATE |
| 5 | PURIFY | PURIFY |
| 6 | YIELD 4-[(Pyridazin-4-ylamino)-methyl]-piperidine-1-carboxylic acid benzyl ester | YIELD 4-[(Pyridazin-4-ylamino)-methyl]-piperidine-1-carboxylic acid benzyl ester |

---

```
Reaction no 208
```

Generated by the Chemistry Development Kit (http://github.com/cdk)

|  | A | B |
| --- | --- | --- |
| 0 | ADD [5-(4-{1-ethyl-1-[4-(2-hydroxy-3,3-dimethyl-butoxy)-3-methyl-phenyl]-propyl}-2-methyl-phenyl)-pyridin-3-yl]-acetic acid methyl ester | ADD [5-(4-{1-ethyl-1-[4-(2-hydroxy-3,3-dimethyl-butoxy)-3-methyl-phenyl]-propyl}-2-methyl-phenyl)-pyridin-3-yl]-acetic acid methyl ester |
| 1 | ADD methanol | ADD methanol |
| 2 | ADD NaOH | ADD NaOH |
| 3 | STIR for 3600 s | STIR for 28800 s |
| 4 | ADD ammonium chloride | ADD ammonium chloride |
| 5 | EXTRACT with ethyl acetate | EXTRACT with ethyl acetate |
| 6 | COLLECTLAYER organic | COLLECTLAYER organic |
| 7 | WASH with water | WASH with water |
| 8 | DRYSOLUTION over sodium sulfate | DRYSOLUTION over sodium sulfate |
| 9 | CONCENTRATE | CONCENTRATE |
| 10 | PURIFY | YIELD [5-(4-{1-ethyl-1-[4-(2-hydroxy-3,3-dimethyl-butoxy)-3-methyl-phenyl]-propyl}-2-methyl-phenyl)-pyridin-3-yl]-acetic Acid |
| 11 | YIELD [5-(4-{1-ethyl-1-[4-(2-hydroxy-3,3-dimethyl-butoxy)-3-methyl-phenyl]-propyl}-2-methyl-phenyl)-pyridin-3-yl]-acetic Acid |  |

---

```
Reaction no 209
```

Generated by the Chemistry Development Kit (http://github.com/cdk)

|  | A | B |
| --- | --- | --- |
| 0 | ADD 5-{2-Fluoro-4-methyl-5-[(2,2,2-trifluoroethyl)sulphanyl]phenyl}-1-methyl-6-(trifluoromethyl)-1,5-dihydro-4H-pyrazolo[3,4-d]pyrimidin-4-one | ADD 5-{2-Fluoro-4-methyl-5-[(2,2,2-trifluoroethyl)sulphanyl]phenyl}-1-methyl-6-(trifluoromethyl)-1,5-dihydro-4H-pyrazolo[3,4-d]pyrimidin-4-one |
| 1 | ADD dichloromethane | ADD dichloromethane |
| 2 | ADD m-chloroperbenzoic acid at 0 °C | ADD m-chloroperbenzoic acid at 0 °C |
| 3 | STIR for 3600 s at 25 °C | STIR for 3600 s at 25 °C |
| 4 | ADD bisulfite | ADD bisulfite |
| 5 | EXTRACT with dichloromethane | EXTRACT with dichloromethane |
| 6 | COLLECTLAYER organic | COLLECTLAYER organic |
| 7 | WASH with sodium bicarbonate | WASH with sodium hydroxide |
| 8 | DRYSOLUTION over Na2SO4 | DRYSOLUTION over sodium sulfate |
| 9 | FILTER keep filtrate | CONCENTRATE |
| 10 | CONCENTRATE | PURIFY |
| 11 | YIELD 5-{2-Fluoro-4-methyl-5-[(2,2,2-trifluoroethyl)sulphinyl]phenyl}-1-methyl-6-(trifluoromethyl)-1,5-dihydro-4H-pyrazolo[3,4-d]pyrimidin-4-one | YIELD 5-{2-Fluoro-4-methyl-5-[(2,2,2-trifluoroethyl)sulphinyl]phenyl}-1-methyl-6-(trifluoromethyl)-1,5-dihydro-4H-pyrazolo[3,4-d]pyrimidin-4-one |

---

```
Reaction no 210
```

Generated by the Chemistry Development Kit (http://github.com/cdk)

|  | A | B |
| --- | --- | --- |
| 0 | ADD THF | ADD 1-(2-(2-(2-azidoethoxy)ethoxy)ethoxy)-4-(tert-butyl)benzene |
| 1 | ADD 1-(2-(2-(2-azidoethoxy)ethoxy)ethoxy)-4-(tert-butyl)benzene | ADD THF |
| 2 | ADD triphenylphosphine | ADD water |
| 3 | WAIT for 3600 s | ADD triphenylphosphine |
| 4 | ADD water | STIR for 86400 s at 25 °C |
| 5 | STIR for 86400 s at 25 °C | CONCENTRATE |
| 6 | CONCENTRATE | PURIFY |
| 7 | PURIFY | YIELD 2-(2-(2-(4-(tert-Buty)phenoxy)ethoxy)ethoxy)ethanamine |
| 8 | YIELD 2-(2-(2-(4-(tert-Buty)phenoxy)ethoxy)ethoxy)ethanamine |  |

---

```
Reaction no 211
```

Generated by the Chemistry Development Kit (http://github.com/cdk)

|  | A | B |
| --- | --- | --- |
| 0 | ADD 4-aminomethyl benzonitrile | ADD CDI at 0 °C |
| 1 | ADD dichloromethane | ADD dichloromethane at 0 °C |
| 2 | ADD CDI at 25 °C | ADD 4-aminomethyl benzonitrile at 0 °C |
| 3 | ADD DIPEA at 25 °C | ADD DIPEA at 0 °C |
| 4 | STIR for 3600 s at 25 °C | STIR for 3600 s at 25 °C |
| 5 | ADD 4-piperidin-4-ylmethylpyridine dihydrochloride | SETTEMPERATURE 0 °C |
| 6 | STIR for 86400 s at 25 °C | MAKESOLUTION with 4-piperidin-4-ylmethylpyridine dihydrochloride and DIPEA and dichloromethane |
| 7 | PURIFY | ADD SLN |
| 8 | YIELD N-[(4-Cyanophenyl)methyl]-4-(pyridin-4-ylmethyl)piperidine-1-carboxamide | STIR for 86400 s |
| 9 |  | CONCENTRATE |
| 10 |  | PURIFY |
| 11 |  | YIELD N-[(4-Cyanophenyl)methyl]-4-(pyridin-4-ylmethyl)piperidine-1-carboxamide |

---

```
Reaction no 212
```

Generated by the Chemistry Development Kit (http://github.com/cdk)

|  | A | B |
| --- | --- | --- |
| 0 | ADD 5-(2-chlorophenyl)-7-[4-(4-isobutylphenyl)butyl]-1,3-dihydro-2H-thieno[2,3-e]-1,4-diazepin-2-one | ADD 5-(2-chlorophenyl)-7-[4-(4-isobutylphenyl)butyl]-1,3-dihydro-2H-thieno[2,3-e]-1,4-diazepin-2-one |
| 1 | ADD toluene | ADD Lawesson's reagent |
| 2 | ADD Lawesson's reagent | ADD toluene |
| 3 | STIR for 3600 s at 100 °C | STIR for 3600 s at 25 °C |
| 4 | CONCENTRATE | CONCENTRATE |
| 5 | PURIFY | PURIFY |
| 6 | YIELD 5-(2-chlorophenyl)-7-[4-(4-isobutylphenyl)butyl]-1,3-dihydro-2H-thieno[2,3-e]-1,4-diazepine-2-thione | CONCENTRATE |
| 7 |  | YIELD 5-(2-chlorophenyl)-7-[4-(4-isobutylphenyl)butyl]-1,3-dihydro-2H-thieno[2,3-e]-1,4-diazepine-2-thione |

---

```
Reaction no 213
```

Generated by the Chemistry Development Kit (http://github.com/cdk)

|  | A | B |
| --- | --- | --- |
| 0 | ADD copper(II) acetate | ADD 10-ethynyl-8-chloro-dibenzo[b,f]-thiepin |
| 1 | ADD methanesulfonic acid | ADD dioxane |
| 2 | ADD formaldehyde | ADD formaldehyde |
| 3 | ADD dimethylamine | ADD dimethylamine |
| 4 | ADD dioxane | ADD dioxane |
| 5 | ADD 10-ethynyl-8-chloro-dibenzo[b,f]-thiepin | ADD copper(II) acetate |
| 6 | STIR for 86400 s at 25 °C | STIR for 3600 s at 100 °C |
| 7 | EXTRACT with ether | SETTEMPERATURE 25 °C |
| 8 | COLLECTLAYER organic | ADD water |
| 9 | WASH with water | PH with methanesulfonic acid to pH acidic |
| 10 | DRYSOLUTION over sodium sulfate | PH with ether to pH neutral |
| 11 | CONCENTRATE | COLLECTLAYER aqueous |
| 12 | PURIFY | PH with ammonia to pH basic |
| 13 | YIELD N,N-dimethyl-3-(8-chloro-dibenzo [b,f]thiepin-10-yl)-2-propynylamine | EXTRACT with ether |
| 14 |  | YIELD N,N-dimethyl-3-(8-chloro-dibenzo [b,f]thiepin-10-yl)-2-propynylamine |

---

```
Reaction no 214
```

Generated by the Chemistry Development Kit (http://github.com/cdk)

|  | A | B |
| --- | --- | --- |
| 0 | ADD 1-[2-(4-chloro-5-methyl-3-trifluoromethyl-pyrazol-1-yl)-acetyl]-4-(4-chloro-phenyl)-piperidine-4-carboxylic acid | ADD 1-[2-(4-chloro-5-methyl-3-trifluoromethyl-pyrazol-1-yl)-acetyl]-4-(4-chloro-phenyl)-piperidine-4-carboxylic acid |
| 1 | ADD piperidine | ADD piperidine |
| 2 | ADD triethylamine | ADD triethylamine |
| 3 | ADD NMP | ADD BOP |
| 4 | ADD BOP | ADD NMP |
| 5 | STIR for 86400 s at 25 °C | STIR for 86400 s at 25 °C |
| 6 | PURIFY | PURIFY |
| 7 | YIELD 2-(4-chloro-5-methyl-3-trifluoromethyl-pyrazol-1-yl)-1-[4-(4-chloro-phenyl)-4-(piperidine-1-carbonyl)-piperidin-1-yl]-ethanone | YIELD 2-(4-chloro-5-methyl-3-trifluoromethyl-pyrazol-1-yl)-1-[4-(4-chloro-phenyl)-4-(piperidine-1-carbonyl)-piperidin-1-yl]-ethanone |

---

```
Reaction no 215
```

Generated by the Chemistry Development Kit (http://github.com/cdk)

|  | A | B |
| --- | --- | --- |
| 0 | ADD N-(5-cyanopyridin-3-yl)-4-{4-[(3-fluorobenzyl)oxy]phenoxy}piperidine-1-carboxamide | ADD N-(5-cyanopyridin-3-yl)-4-{4-[(3-fluorobenzyl)oxy]phenoxy}piperidine-1-carboxamide |
| 1 | ADD ethanol | ADD ethanol |
| 2 | ADD NaOH | ADD NaOH |
| 3 | STIR for 86400 s at 100 °C | STIR for 86400 s at 100 °C |
| 4 | CONCENTRATE | CONCENTRATE |
| 5 | ADD water | ADD water |
| 6 | PH with HCl to pH acidic | ADD methanol |
| 7 | FILTER keep precipitate | FILTER keep filtrate |
| 8 | WASH with water | ADD HCl at 0 °C |
| 9 | DRYSOLID under vacuum | FILTER keep precipitate |
| 10 | YIELD 5-{[(4-{4-[(3-fluorobenzyl)oxy]phenoxy}piperidin-1-yl)carbonyl]amino}nicotinic acid | DRYSOLID |
| 11 |  | YIELD 5-{[(4-{4-[(3-fluorobenzyl)oxy]phenoxy}piperidin-1-yl)carbonyl]amino}nicotinic acid |

---

```
Reaction no 216
```

Generated by the Chemistry Development Kit (http://github.com/cdk)

|  | A | B |
| --- | --- | --- |
| 0 | ADD ethyl 2-(1-methyl-4-phenyl-1H-imidazol-2-yl)cyclopropanecarboxylate | ADD ethyl 2-(1-methyl-4-phenyl-1H-imidazol-2-yl)cyclopropanecarboxylate |
| 1 | ADD THF | ADD THF |
| 2 | ADD methanol | MAKESOLUTION with lithium hydroxide monohydrate and water |
| 3 | ADD water | ADD SLN at 25 °C |
| 4 | ADD lithium hydroxide monohydrate | ADD methanol |
| 5 | STIR for 86400 s at 25 °C | STIR for 86400 s at 25 °C |
| 6 | PH with HCl to pH acidic | CONCENTRATE |
| 7 | ADD toluene | FILTER keep filtrate |
| 8 | PHASESEPARATION | ADD water |
| 9 | COLLECTLAYER aqueous | PH with HCl to pH neutral |
| 10 | EXTRACT with toluene | CONCENTRATE |
| 11 | COLLECTLAYER organic | TRITURATE with toluene |
| 12 | DRYSOLUTION over sodium sulfate | DRYSOLID for 86400 s |
| 13 | FILTER keep filtrate | YIELD 2-(1-methyl-4-phenyl-1H-imidazol-2-yl)cyclopropanecarboxylic acid |
| 14 | CONCENTRATE |  |
| 15 | YIELD 2-(1-methyl-4-phenyl-1H-imidazol-2-yl)cyclopropanecarboxylic acid |  |

---

```
Reaction no 217
```

Generated by the Chemistry Development Kit (http://github.com/cdk)

|  | A | B |
| --- | --- | --- |
| 0 | ADD deoxycholic acid | ADD deoxycholic acid |
| 1 | ADD DMF | ADD K2CO3 |
| 2 | ADD K2CO3 | ADD DMF |
| 3 | ADD methyl iodide | ADD methyl iodide |
| 4 | STIR for 86400 s at 25 °C | STIR for 28800 s at 25 °C |
| 5 | ADD ethyl acetate | ADD ethyl acetate |
| 6 | WASH with water | WASH with water |
| 7 | WASH with brine | WASH with sodium chloride |
| 8 | DRYSOLUTION over sodium sulfate | DRYSOLUTION over magnesium sulfate |
| 9 | CONCENTRATE | FILTER keep filtrate |
| 10 | PURIFY | CONCENTRATE |
| 11 | YIELD methyl deoxycholate | PURIFY |
| 12 |  | YIELD methyl deoxycholate |

---

```
Reaction no 218
```

Generated by the Chemistry Development Kit (http://github.com/cdk)

|  | A | B |
| --- | --- | --- |
| 0 | ADD benzylmagnesium chloride | ADD N-(3-chloro-4-cyanophenyl)-2-oxo-2-cyclohexylacetamide |
| 1 | ADD THF | ADD THF |
| 2 | ADD THF | MAKESOLUTION with benzylmagnesium chloride and THF |
| 3 | SETTEMPERATURE -70 °C | ADD SLN at 0 °C |
| 4 | MAKESOLUTION with N-(3-chloro-4-cyanophenyl)-2-oxo-2-cyclohexylacetamide and THF | STIR for 3600 s at 25 °C |
| 5 | ADD SLN | QUENCH with ammonium chloride |
| 6 | STIR for 3600 s at -70 °C | EXTRACT with ethyl acetate |
| 7 | ADD ammonium chloride | COLLECTLAYER organic |
| 8 | EXTRACT with ethyl acetate | DRYSOLUTION over Na2SO4 |
| 9 | COLLECTLAYER organic | CONCENTRATE |
| 10 | WASH with sodium chloride | PURIFY |
| 11 | DRYSOLUTION over Na2SO4 | YIELD rac- N-(3-Chlor-4-cyanophenyl)-2-hydroxy-2-cyclohexyl-3-phenylpropanamid |
| 12 | PURIFY |  |
| 13 | YIELD rac- N-(3-Chlor-4-cyanophenyl)-2-hydroxy-2-cyclohexyl-3-phenylpropanamid |  |

---

```
Reaction no 219
```

Generated by the Chemistry Development Kit (http://github.com/cdk)

|  | A | B |
| --- | --- | --- |
| 0 | ADD 4-{2-[(2R)-4-[(Benzyloxy)carbonyl]-1-({1-[(1R,2S)-2-hydroxy-2-(methoxymethyl)cyclohexyl]-5-phenyl-1H-imidazol-4-yl}carbonyl)piperazin-2-yl]ethoxy}benzoic acid | ADD 4-{2-[(2R)-4-[(Benzyloxy)carbonyl]-1-({1-[(1R,2S)-2-hydroxy-2-(methoxymethyl)cyclohexyl]-5-phenyl-1H-imidazol-4-yl}carbonyl)piperazin-2-yl]ethoxy}benzoic acid |
| 1 | ADD cyclopropylamine | ADD DMF |
| 2 | ADD 1-(3-dimethylaminopropyl)-3-ethylcarbodiimide hydrochloride | ADD cyclopropylamine |
| 3 | ADD HOBt | ADD 1-(3-dimethylaminopropyl)-3-ethylcarbodiimide hydrochloride |
| 4 | ADD DMF | ADD HOBt |
| 5 | STIR for 86400 s at 25 °C | STIR for 86400 s at 25 °C |
| 6 | ADD NaHCO3 | ADD NaHCO3 |
| 7 | EXTRACT with ethyl acetate | EXTRACT with ethyl acetate |
| 8 | WASH with water | WASH with water |
| 9 | WASH with brine | WASH with brine |
| 10 | DRYSOLUTION over magnesium sulfate | DRYSOLUTION over sodium sulfate |
| 11 | CONCENTRATE | CONCENTRATE |
| 12 | PURIFY | PURIFY |
| 13 | CONCENTRATE | CONCENTRATE |
| 14 | YIELD Benzyl ‌(3R)-3-(2-{4-[(cyclopropylamino)carbonyl]phenoxy}ethyl)-4-({1-[(1R,2S)-2-hydroxy-2-(methoxymethyl)cyclohexyl]-5-phenyl-1H-imidazol-4-yl}carbonyl)piperazine-1-carboxylate | YIELD Benzyl ‌(3R)-3-(2-{4-[(cyclopropylamino)carbonyl]phenoxy}ethyl)-4-({1-[(1R,2S)-2-hydroxy-2-(methoxymethyl)cyclohexyl]-5-phenyl-1H-imidazol-4-yl}carbonyl)piperazine-1-carboxylate |

---

```
Reaction no 220
```

Generated by the Chemistry Development Kit (http://github.com/cdk)

|  | A | B |
| --- | --- | --- |
| 0 | ADD 5-(4-Chlorophenyl)-1-(2,4-dichlorophenyl)-4-methyl-1H-pyrazole-3-carboxamide | ADD 5-(4-Chlorophenyl)-1-(2,4-dichlorophenyl)-4-methyl-1H-pyrazole-3-carboxamide |
| 1 | ADD THF | ADD THF |
| 2 | ADD NaHMDS at -70 °C | ADD NaHMDS at 0 °C |
| 3 | STIR for 600 s | STIR for 600 s at 0 °C |
| 4 | ADD benzyl chloroformate | ADD benzyl chloroformate |
| 5 | ADD THF dropwise | STIR for 3600 s at 0 °C |
| 6 | STIR for 3600 s | ADD NaHCO3 |
| 7 | STIR for 86400 s at 25 °C | EXTRACT with ethyl acetate |
| 8 | ADD NaHCO3 | COLLECTLAYER organic |
| 9 | EXTRACT with ethyl acetate | DRYSOLUTION over Na2SO4 |
| 10 | COLLECTLAYER organic | FILTER keep filtrate |
| 11 | WASH with water | CONCENTRATE |
| 12 | DRYSOLUTION over MgSO4 | PURIFY |
| 13 | CONCENTRATE | YIELD Benzyl 5-(4-chlorophenyl)-1-(2,4-dichlorophenyl)-4-methyl-1H-pyrazole-3-carbonylcarbamate |
| 14 | PURIFY |  |
| 15 | YIELD Benzyl 5-(4-chlorophenyl)-1-(2,4-dichlorophenyl)-4-methyl-1H-pyrazole-3-carbonylcarbamate |  |

---

```
Reaction no 221
```

Generated by the Chemistry Development Kit (http://github.com/cdk)

|  | A | B |
| --- | --- | --- |
| 0 | ADD (3,5-dichloropyrazin-2-yl)(2-(4-methoxybenzylamino)-5-methyl pyridin-4-yl)-methanone | ADD (3,5-dichloropyrazin-2-yl)(2-(4-methoxybenzylamino)-5-methyl pyridin-4-yl)-methanone |
| 1 | ADD (R)—N—((R)-1-(4-methoxyphenyl)ethyl)-8-azaspiro[4.5]decan-1-amine | ADD DMF |
| 2 | ADD DMF | ADD (R)—N—((R)-1-(4-methoxyphenyl)ethyl)-8-azaspiro[4.5]decan-1-amine |
| 3 | ADD K2CO3 | ADD K2CO3 |
| 4 | STIR for 86400 s at 25 °C | STIR for 3600 s at 25 °C |
| 5 | PURIFY | CONCENTRATE |
| 6 | YIELD (3-Chloro-5-((R)-1-((R)-1-(4-methoxyphenyl)ethylamino)-8-azaspiro[4.5]decan-8-yl)pyrazin-2-yl)(2-(4-methoxybenzylamino)-5-methylpyridin-4-yl)methanone | YIELD (3-Chloro-5-((R)-1-((R)-1-(4-methoxyphenyl)ethylamino)-8-azaspiro[4.5]decan-8-yl)pyrazin-2-yl)(2-(4-methoxybenzylamino)-5-methylpyridin-4-yl)methanone |

---

```
Reaction no 222
```

Generated by the Chemistry Development Kit (http://github.com/cdk)

|  | A | B |
| --- | --- | --- |
| 0 | ADD 5-[3-(Aminomethyl)-1,2,4-oxadiazol-5-yl]-1-ethyl-N-(tetrahydro-2H-pyran-4-yl)-1H-pyrazolo[3,4-b]pyridin-4-amine | ADD 5-[3-(Aminomethyl)-1,2,4-oxadiazol-5-yl]-1-ethyl-N-(tetrahydro-2H-pyran-4-yl)-1H-pyrazolo[3,4-b]pyridin-4-amine |
| 1 | ADD DIPEA | ADD chloroform |
| 2 | ADD chloroform | ADD DIPEA at 0 °C |
| 3 | ADD 4-chlorobutyryl chloride at 25 °C | ADD 4-chlorobutyryl chloride at 0 °C |
| 4 | STIR for 86400 s at 25 °C | STIR for 86400 s at 25 °C |
| 5 | PURIFY | CONCENTRATE |
| 6 | COLLECTLAYER organic | PURIFY |
| 7 | CONCENTRATE | YIELD 4-Chloro-N-({5-[1-ethyl-4-(tetrahydro-2H-pyran-4-ylamino)-1H-pyrazolo[3,4-b]pyridin-5-yl]-1,2,4-oxadiazol-3-yl}methyl)butanamide |
| 8 | PURIFY |  |
| 9 | YIELD 4-Chloro-N-({5-[1-ethyl-4-(tetrahydro-2H-pyran-4-ylamino)-1H-pyrazolo[3,4-b]pyridin-5-yl]-1,2,4-oxadiazol-3-yl}methyl)butanamide |  |

---

```
Reaction no 223
```

Generated by the Chemistry Development Kit (http://github.com/cdk)

|  | A | B |
| --- | --- | --- |
| 0 | ADD 3-methylene-1-methylcyclopentanecarboxylic acid | ADD 3-methylene-1-methylcyclopentanecarboxylic acid |
| 1 | ADD 3,5-bis(trifluoromethyl)benzylamine hydrochloride | ADD 3,5-bis(trifluoromethyl)benzylamine hydrochloride |
| 2 | ADD HOAt | ADD HOAt |
| 3 | ADD 1-(3-dimethylaminopropyl)-3-ethylcarbodiimide hydrochloride | ADD DIPEA |
| 4 | ADD DIPEA | ADD 1-(3-dimethylaminopropyl)-3-ethylcarbodiimide hydrochloride |
| 5 | ADD dichloromethane | ADD dichloromethane |
| 6 | STIR for 86400 s at 25 °C | STIR for 3600 s at 25 °C |
| 7 | CONCENTRATE | ADD dichloromethane |
| 8 | PURIFY | WASH with water |
| 9 | YIELD 3,5-Bis(trifluoromethyl)benzyl 3-methylene-1-methylcyclopentanecarboxamide | WASH with brine |
| 10 |  | DRYSOLUTION |
| 11 |  | CONCENTRATE |
| 12 |  | PURIFY |
| 13 |  | YIELD 3,5-Bis(trifluoromethyl)benzyl 3-methylene-1-methylcyclopentanecarboxamide |

---

```
Reaction no 224
```

Generated by the Chemistry Development Kit (http://github.com/cdk)

|  | A | B |
| --- | --- | --- |
| 0 | ADD 6-ethylpyridin-2-amine | ADD 6-ethylpyridin-2-amine |
| 1 | ADD sulfuric acid | ADD 6-ethyl-5-nitro-2-pyridinamine |
| 2 | ADD nitric acid at 0 °C | ADD sulfuric acid |
| 3 | SETTEMPERATURE 60 °C | ADD nitric acid dropwise at 0 °C |
| 4 | STIR for 86400 s at 25 °C | STIR for 3600 s at 0 °C |
| 5 | WAIT for 86400 s | ADD ice water |
| 6 | SETTEMPERATURE 25 °C | EXTRACT with ethyl acetate |
| 7 | PH with sodium hydroxide to pH acidic | COLLECTLAYER organic |
| 8 | FILTER keep precipitate | DRYSOLUTION over sodium sulfate |
| 9 | WASH with water | CONCENTRATE |
| 10 | DRYSOLID | PURIFY |
| 11 | PURIFY | YIELD 6-ethyl-3-nitro-2-pyridinamine |
| 12 | YIELD 6-ethyl-3-nitro-2-pyridinamine |  |
| 13 | YIELD 6-ethyl-5-nitro-2-pyridinamine |  |

---

```
Reaction no 225
```

Generated by the Chemistry Development Kit (http://github.com/cdk)

|  | A | B |
| --- | --- | --- |
| 0 | ADD 4-chloro-1-(tetrahydro-pyran-4-yl)-1H-pyrazolo[3,4-b]pyridine-5-carboxylic acid ethyl ester | ADD 4-chloro-1-(tetrahydro-pyran-4-yl)-1H-pyrazolo[3,4-b]pyridine-5-carboxylic acid ethyl ester |
| 1 | ADD DMF | ADD DMF |
| 2 | ADD N-chlorosuccinimide | ADD N-chlorosuccinimide |
| 3 | STIR for 86400 s at 25 °C | STIR for 86400 s at 100 °C |
| 4 | ADD water | ADD water |
| 5 | EXTRACT with ethyl acetate | EXTRACT with ethyl acetate |
| 6 | COLLECTLAYER organic | COLLECTLAYER organic |
| 7 | WASH with brine | CONCENTRATE |
| 8 | DRYSOLUTION over sodium sulfate | PURIFY |
| 9 | CONCENTRATE | YIELD 3,4-dichloro-1-(tetrahydro-pyran-4-yl)-1H-pyrazolo[3,4-b]pyridine-5-carboxylic acid ethyl ester |
| 10 | PURIFY |  |
| 11 | YIELD 3,4-dichloro-1-(tetrahydro-pyran-4-yl)-1H-pyrazolo[3,4-b]pyridine-5-carboxylic acid ethyl ester |  |

---

```
Reaction no 226
```

Generated by the Chemistry Development Kit (http://github.com/cdk)

|  | A | B |
| --- | --- | --- |
| 0 | ADD (S,S) [1-[5-(4-Amino-phenoxy)-1H-indol-3-ylmethyl]-2-(2cyano-pyrrolidin-1-yl)-2-oxo-ethyl]-carbamic acid tert-butyl ester | ADD (S,S) [1-[5-(4-Amino-phenoxy)-1H-indol-3-ylmethyl]-2-(2cyano-pyrrolidin-1-yl)-2-oxo-ethyl]-carbamic acid tert-butyl ester |
| 1 | ADD dichloromethane | ADD TFA |
| 2 | ADD TFA | ADD dichloromethane |
| 3 | STIR for 3600 s at 25 °C | STIR for 3600 s at 0 °C |
| 4 | CONCENTRATE | CONCENTRATE |
| 5 | PURIFY | RECRYSTALLIZE from 2-amino-3-[5-(4-amino-phenoxy)-1H-indol-3-yl]-propionyl-pyrrolidine-2-carbonitrile |
| 6 | YIELD 2-amino-3-[5-(4-amino-phenoxy)-1H-indol-3-yl]-propionyl-pyrrolidine-2-carbonitrile | PURIFY |

---

```
Reaction no 227
```

Generated by the Chemistry Development Kit (http://github.com/cdk)

|  | A | B |
| --- | --- | --- |
| 0 | ADD propargyl alcohol | ADD propargyl alcohol |
| 1 | ADD THF | ADD THF |
| 2 | ADD lithium | ADD lithium at -30 °C |
| 3 | ADD ammonia | STIR for 3600 s at -30 °C |
| 4 | ADD ferric chloride | ADD hexadecyl bromide |
| 5 | STIR for 3600 s | ADD ferric chloride |
| 6 | ADD hexadecyl bromide | STIR for 3600 s at -30 °C |
| 7 | REFLUX for 28800 s | STIR for 86400 s at 25 °C |
| 8 | CONCENTRATE | ADD ammonia |
| 9 | ADD ether | EXTRACT with ether |
| 10 | WASH with HCl | COLLECTLAYER organic |
| 11 | DRYSOLUTION | WASH with water |
| 12 | PURIFY | WASH with brine |
| 13 | YIELD 2-nonadecyn-1-ol | DRYSOLUTION over magnesium sulfate |
| 14 |  | CONCENTRATE |
| 15 |  | PURIFY |
| 16 |  | YIELD 2-nonadecyn-1-ol |

---

```
Reaction no 228
```

Generated by the Chemistry Development Kit (http://github.com/cdk)

|  | A | B |
| --- | --- | --- |
| 0 | ADD 2-(R)-(7-methoxy-2,3-dihydro-isoindol-1-one-2-ylmethyl)-4,4-diethoxy-1-(1-(S)-phenyl-ethyl)-piperidine | ADD 2-(R)-(7-methoxy-2,3-dihydro-isoindol-1-one-2-ylmethyl)-4,4-diethoxy-1-(1-(S)-phenyl-ethyl)-piperidine |
| 1 | ADD TFA | ADD TFA |
| 2 | STIR for 3600 s at 100 °C | ADD water |
| 3 | ADD water | STIR for 3600 s at 0 °C |
| 4 | PH with Na2CO3 to pH basic | ADD Na2CO3 |
| 5 | EXTRACT with dichloromethane | EXTRACT with dichloromethane |
| 6 | COLLECTLAYER organic | COLLECTLAYER organic |
| 7 | DRYSOLUTION over Na2SO4 | DRYSOLUTION over MgSO4 |
| 8 | FILTER keep filtrate | FILTER keep filtrate |
| 9 | CONCENTRATE | CONCENTRATE |
| 10 | PURIFY | YIELD 2-(R)-(7-methoxy-2,3-dihydro-isoindol-1-one-2-ylmethyl)-4-oxo-1(1-(S)-phenyl-ethyl)-piperidine |
| 11 | YIELD 2-(R)-(7-methoxy-2,3-dihydro-isoindol-1-one-2-ylmethyl)-4-oxo-1(1-(S)-phenyl-ethyl)-piperidine |  |

---

```
Reaction no 229
```

Generated by the Chemistry Development Kit (http://github.com/cdk)

|  | A | B |
| --- | --- | --- |
| 0 | ADD 3-Amino-5-nitro-1H-pyrazole | ADD 3-Amino-5-nitro-1H-pyrazole |
| 1 | ADD methyl acetoacetate | ADD methyl acetoacetate |
| 2 | ADD methanol | ADD methanol |
| 3 | REFLUX for 3600 s | REFLUX for 86400 s |
| 4 | SETTEMPERATURE 25 °C | SETTEMPERATURE 25 °C |
| 5 | FILTER keep precipitate | CONCENTRATE |
| 6 | YIELD 7-methyl-2-nitro-pyrazolo[1,5-a]pyrimidine-5-carboxylic acid methyl ester | PURIFY |
| 7 |  | YIELD 7-methyl-2-nitro-pyrazolo[1,5-a]pyrimidine-5-carboxylic acid methyl ester |

---

```
Reaction no 230
```

Generated by the Chemistry Development Kit (http://github.com/cdk)

|  | A | B |
| --- | --- | --- |
| 0 | ADD 1-methyl-1H-benzimidazole-6-carbohydrazide | ADD 1-methyl-1H-benzimidazole-6-carbohydrazide |
| 1 | ADD carbon disulfide | ADD triethylamine |
| 2 | ADD KOH | ADD carbon disulfide |
| 3 | ADD ethanol | ADD ethanol |
| 4 | REFLUX for 3600 s | REFLUX for 86400 s |
| 5 | SETTEMPERATURE 25 °C | SETTEMPERATURE 0 °C |
| 6 | CONCENTRATE | ADD KOH |
| 7 | ADD 3-(trifluoromethyl)benzyl chloride | ADD 3-(trifluoromethyl)benzyl chloride |
| 8 | ADD triethylamine | STIR for 86400 s at 25 °C |
| 9 | REFLUX for 3600 s | ADD ethyl acetate |
| 10 | SETTEMPERATURE 25 °C | WASH with water |
| 11 | ADD ethyl acetate | WASH with brine |
| 12 | WASH with water | DRYSOLUTION over magnesium sulfate |
| 13 | WASH with brine | CONCENTRATE |
| 14 | DRYSOLUTION over magnesium sulfate | PURIFY |
| 15 | CONCENTRATE | YIELD 1-methyl-6-[5-[[3-(trifluoromethyl)benzyl]thio]-1,3,4-oxadiazol-2-yl]-1H-benzimidazole |
| 16 | PURIFY |  |
| 17 | YIELD 1-methyl-6-[5-[[3-(trifluoromethyl)benzyl]thio]-1,3,4-oxadiazol-2-yl]-1H-benzimidazole |  |

---

```
Reaction no 231
```

Generated by the Chemistry Development Kit (http://github.com/cdk)

|  | A | B |
| --- | --- | --- |
| 0 | ADD tert-butyl 6-[2-(4-bromo-2-formylphenoxymethyl)pyridin-5-yl]piperidine-1-carboxylate | ADD tert-butyl 6-[2-(4-bromo-2-formylphenoxymethyl)pyridin-5-yl]piperidine-1-carboxylate |
| 1 | ADD dichloromethane | ADD dichloromethane |
| 2 | ADD TFA | ADD TFA |
| 3 | STIR for 3600 s at 25 °C | STIR for 28800 s at 25 °C |
| 4 | CONCENTRATE | CONCENTRATE |
| 5 | DRYSOLID under vacuum | ADD DMF |
| 6 | ADD dichloromethane | ADD benzyl bromide |
| 7 | ADD K2CO3 | ADD K2CO3 |
| 8 | ADD benzyl bromide | STIR for 86400 s at 100 °C |
| 9 | STIR for 86400 s at 25 °C | SETTEMPERATURE 25 °C |
| 10 | ADD water | ADD water |
| 11 | EXTRACT with dichloromethane | EXTRACT with ethyl acetate |
| 12 | COLLECTLAYER organic | COLLECTLAYER organic |
| 13 | DRYSOLUTION over Na2SO4 | WASH with brine |
| 14 | FILTER keep filtrate | DRYSOLUTION over sodium sulfate |
| 15 | CONCENTRATE | CONCENTRATE |
| 16 | PURIFY | ADD acetic acid |
| 17 | YIELD 5-(1-Benzylpiperidin-4-yl)-2-(5-bromobenzofuran-2-yl)pyridine | REFLUX for 86400 s |
| 18 |  | SETTEMPERATURE 25 °C |
| 19 |  | ADD water |
| 20 |  | EXTRACT with ethyl acetate |
| 21 |  | COLLECTLAYER organic |
| 22 |  | WASH with brine |
| 23 |  | DRYSOLUTION over sodium sulfate |
| 24 |  | CONCENTRATE |
| 25 |  | PURIFY |
| 26 |  | YIELD 5-(1-Benzylpiperidin-4-yl)-2-(5-bromobenzofuran-2-yl)pyridine |

---

```
Reaction no 232
```

Generated by the Chemistry Development Kit (http://github.com/cdk)

|  | A | B |
| --- | --- | --- |
| 0 | ADD 4-amino-2-benzyl-6-(4-methoxyphenyl)-2H-pyridazin-3-one | ADD 4-amino-2-benzyl-6-(4-methoxyphenyl)-2H-pyridazin-3-one |
| 1 | ADD triethylamine | ADD triethylamine |
| 2 | ADD toluene | ADD chloroform |
| 3 | ADD methanesulfonyl chloride | ADD methanesulfonyl chloride |
| 4 | STIR for 3600 s at 60 °C | STIR for 86400 s at 25 °C |
| 5 | ADD chloroform | ADD toluene |
| 6 | COLLECTLAYER organic | WASH with water |
| 7 | WASH with water | WASH with brine |
| 8 | WASH with brine | DRYSOLUTION over magnesium sulfate |
| 9 | DRYSOLUTION over sodium sulfate | CONCENTRATE |
| 10 | CONCENTRATE | PURIFY |
| 11 | PURIFY | YIELD 2-benzyl-4-dimethanesulfonylamino-6-(4-methoxyphenyl)-2H-pyridazin-3-one |
| 12 | YIELD 2-benzyl-4-dimethanesulfonylamino-6-(4-methoxyphenyl)-2H-pyridazin-3-one |  |

---

```
Reaction no 233
```

Generated by the Chemistry Development Kit (http://github.com/cdk)

|  | A | B |
| --- | --- | --- |
| 0 | ADD tert-Butyl [2-{[(6-Chloropyridin-3-yl)carbonyl]amino}-4-(2-thienyl)phenyl]-carbamate | ADD tert-Butyl [2-{[(6-Chloropyridin-3-yl)carbonyl]amino}-4-(2-thienyl)phenyl]-carbamate |
| 1 | ADD 1-methylpiperazine | ADD 1-methylpiperazine |
| 2 | STIR for 3600 s at 100 °C | STIR for 86400 s at 100 °C |
| 3 | ADD ethyl acetate | ADD ethyl acetate |
| 4 | WASH with water | WASH with NaHCO3 |
| 5 | WASH with brine | WASH with brine |
| 6 | DRYSOLUTION over Na2SO4 | DRYSOLUTION over MgSO4 |
| 7 | CONCENTRATE | FILTER keep filtrate |
| 8 | PURIFY | CONCENTRATE |
| 9 | YIELD CN1CCN(c2ccc(C(=O)Nc3cc(-c4cccs4)ccc3NC(=O)OC(C)(C)C)cn2)CC1 | PURIFY |
| 10 |  | YIELD CN1CCN(c2ccc(C(=O)Nc3cc(-c4cccs4)ccc3NC(=O)OC(C)(C)C)cn2)CC1 |

---

```
Reaction no 234
```

Generated by the Chemistry Development Kit (http://github.com/cdk)

|  | A | B |
| --- | --- | --- |
| 0 | ADD methyl 1-(2-chloro-6-formylphenyl)-4-methylpiperidin-3-ylcarbamate | ADD methyl 1-(2-chloro-6-formylphenyl)-4-methylpiperidin-3-ylcarbamate |
| 1 | ADD methanol | ADD methanol |
| 2 | ADD KOH | ADD water |
| 3 | REFLUX for 86400 s | ADD KOH |
| 4 | SETTEMPERATURE 25 °C | REFLUX for 86400 s |
| 5 | ADD water | CONCENTRATE |
| 6 | EXTRACT with dichloromethane | ADD water |
| 7 | COLLECTLAYER organic | EXTRACT with ethyl acetate |
| 8 | DRYSOLUTION over Na2SO4 | COLLECTLAYER organic |
| 9 | FILTER keep filtrate | WASH with brine |
| 10 | PURIFY | DRYSOLUTION over Na2SO4 |
| 11 | YIELD 2-(3-amino-4-methylpiperidin-1-yl)-3-chlorobenzaldehyde | CONCENTRATE |
| 12 |  | YIELD 2-(3-amino-4-methylpiperidin-1-yl)-3-chlorobenzaldehyde |

---

```
Reaction no 235
```

Generated by the Chemistry Development Kit (http://github.com/cdk)

|  | A | B |
| --- | --- | --- |
| 0 | ADD toluene triethylamine | ADD toluene triethylamine |
| 1 | ADD 4-[(6,7-Dimethoxy-4-quinolyl)oxy]-2,3-dimethylaniline | ADD 4-[(6,7-Dimethoxy-4-quinolyl)oxy]-2,3-dimethylaniline |
| 2 | REFLUX | REFLUX |
| 3 | MAKESOLUTION with triphosgene and dichloromethane | MAKESOLUTION with triphosgene and dichloromethane |
| 4 | ADD SLN | ADD SLN |
| 5 | REFLUX for 600 s | REFLUX for 600 s |
| 6 | ADD (2,5-dimethylphenyl sulfanyl]-1-propanol | ADD (2,5-dimethylphenyl sulfanyl]-1-propanol |
| 7 | REFLUX for 3600 s | REFLUX for 3600 s |
| 8 | SETTEMPERATURE 25 °C | SETTEMPERATURE 25 °C |
| 9 | ADD water | ADD water |
| 10 | QUENCH with chloroform | QUENCH with chloroform |
| 11 | WASH with HCl | WASH with HCl |
| 12 | WASH with brine | WASH with brine |
| 13 | DRYSOLUTION over sodium sulfate | DRYSOLUTION over sodium sulfate |
| 14 | CONCENTRATE | CONCENTRATE |
| 15 | PURIFY | PURIFY |
| 16 | YIELD 3-[(2,5-Dimethylphenyl)sulfanyl]propyl N-{4-[(6,7-dimethoxy-4-quinolyl)oxy]-2,3-dimethylphenyl}carbamate | YIELD 3-[(2,5-Dimethylphenyl)sulfanyl]propyl N-{4-[(6,7-dimethoxy-4-quinolyl)oxy]-2,3-dimethylphenyl}carbamate |

---

```
Reaction no 236
```

Generated by the Chemistry Development Kit (http://github.com/cdk)

|  | A | B |
| --- | --- | --- |
| 0 | ADD 1-[4-(4-Chlorophenyl)piperazine-1-sulfonylmethyl]-4-oxo-cyclohexanecarboxylic Acid N-Hydroxy Amide | ADD 1-[4-(4-Chlorophenyl)piperazine-1-sulfonylmethyl]-4-oxo-cyclohexanecarboxylic Acid N-Hydroxy Amide |
| 1 | ADD O-methylhydroxylamine hydrochloride | ADD ethanol |
| 2 | ADD sodium acetate | ADD O-methylhydroxylamine hydrochloride |
| 3 | ADD ethanol | ADD sodium acetate |
| 4 | REFLUX for 28800 s | STIR for 28800 s |
| 5 | CONCENTRATE | CONCENTRATE |
| 6 | ADD water | ADD water |
| 7 | EXTRACT with ethyl acetate | EXTRACT with dichloromethane |
| 8 | WASH with sodium chloride | WASH with water |
| 9 | DRYSOLUTION over MgSO4 | WASH with brine |
| 10 | CONCENTRATE | DRYSOLUTION |
| 11 | PURIFY | CONCENTRATE |
| 12 | YIELD 1-[4-(4-Chlorophenyl)piperazine-1-sulphonylmethyl]-4-methoxyiminocylohexanecarboxylic Acid N-Hydroxy Amide | YIELD 1-[4-(4-Chlorophenyl)piperazine-1-sulphonylmethyl]-4-methoxyiminocylohexanecarboxylic Acid N-Hydroxy Amide |

---

```
Reaction no 237
```

Generated by the Chemistry Development Kit (http://github.com/cdk)

|  | A | B |
| --- | --- | --- |
| 0 | ADD methyl 4-(2-(2-((2-chloro-2′-methyl-3′-(1-methyl-4,5,6,7-tetrahydro-1H-imidazo[4,5-c]pyridine-2-carboxamido)-[1,1′-biphenyl]-3-yl)carbamoyl)-1-methyl-1,4,6,7-tetrahydro-5H-imidazo[4,5-c]pyridin-5-yl)ethyl)cyclohexane-1-carboxylate | ADD methyl 4-(2-(2-((2-chloro-2′-methyl-3′-(1-methyl-4,5,6,7-tetrahydro-1H-imidazo[4,5-c]pyridine-2-carboxamido)-[1,1′-biphenyl]-3-yl)carbamoyl)-1-methyl-1,4,6,7-tetrahydro-5H-imidazo[4,5-c]pyridin-5-yl)ethyl)cyclohexane-1-carboxylate |
| 1 | ADD 4-hydroxycyclohexanone | ADD 4-hydroxycyclohexanone |
| 2 | ADD dichloromethane | ADD dichloromethane |
| 3 | STIR for 3600 s at 25 °C | STIR for 3600 s at 25 °C |
| 4 | ADD sodium triacetoxyborohydride | ADD sodium triacetoxyborohydride |
| 5 | STIR for 3600 s at 25 °C | STIR for 86400 s at 25 °C |
| 6 | WASH with NaHCO3 | PURIFY |
| 7 | CONCENTRATE | YIELD methyl 4-(2-(2-((2′-chloro-3′-(5-(4-hydroxycyclohexyl)-1-methyl-4,5,6,7-tetrahydro-1H-imidazo[4,5-c]pyridine-2-carboxamido)-2-methyl-[1,1′-biphenyl]-3-yl)carbamoyl)-1-methyl-1,4,6,7-tetrahydro-5H-imidazo[4,5-c]pyridin-5-yl)ethyl)cyclohexane-1-carboxylate |
| 8 | PURIFY |  |
| 9 | YIELD methyl 4-(2-(2-((2′-chloro-3′-(5-(4-hydroxycyclohexyl)-1-methyl-4,5,6,7-tetrahydro-1H-imidazo[4,5-c]pyridine-2-carboxamido)-2-methyl-[1,1′-biphenyl]-3-yl)carbamoyl)-1-methyl-1,4,6,7-tetrahydro-5H-imidazo[4,5-c]pyridin-5-yl)ethyl)cyclohexane-1-carboxylate |  |

---

```
Reaction no 238
```

Generated by the Chemistry Development Kit (http://github.com/cdk)

|  | A | B |
| --- | --- | --- |
| 0 | ADD 3-methylbenzophenone | ADD 3-methylbenzophenone |
| 1 | ADD N-bromosuccinimide | ADD carbon tetrachloride |
| 2 | ADD benzoyl peroxide | ADD N-bromosuccinimide |
| 3 | ADD carbon tetrachloride | ADD benzoyl peroxide |
| 4 | REFLUX for 3600 s | REFLUX for 3600 s |
| 5 | SETTEMPERATURE 25 °C | REFLUX for 3600 s |
| 6 | FILTER keep filtrate | ADD benzoyl peroxide |
| 7 | CONCENTRATE | REFLUX for 3600 s |
| 8 | YIELD 3-bromomethylbenzophenone | ADD benzoyl peroxide |
| 9 |  | REFLUX for 3600 s |
| 10 |  | SETTEMPERATURE 25 °C |
| 11 |  | FILTER keep filtrate |
| 12 |  | CONCENTRATE |
| 13 |  | YIELD 3-bromomethylbenzophenone |

---

```
Reaction no 239
```

Generated by the Chemistry Development Kit (http://github.com/cdk)

|  | A | B |
| --- | --- | --- |
| 0 | ADD 8-{2-[3-(1-methylhydrazino)-1-pyrrolidinyl]ethyl}-2-(methyloxy)-1,5-naphthyridine | ADD 8-{2-[3-(1-methylhydrazino)-1-pyrrolidinyl]ethyl}-2-(methyloxy)-1,5-naphthyridine |
| 1 | ADD DCM DMF | ADD 3-oxo-3,4-dihydro-2H-pyrido[3,2-b][1,4]thiazine-6-carboxylic acid |
| 2 | ADD 3-oxo-3,4-dihydro-2H-pyrido[3,2-b][1,4]thiazine-6-carboxylic acid | ADD 1,2-dichloroethane |
| 3 | ADD 1,2-dichloroethane | ADD HOBt |
| 4 | ADD HOBt | ADD DCM DMF |
| 5 | WAIT for 86400 s | STIR for 86400 s at 25 °C |
| 6 | CONCENTRATE | CONCENTRATE |
| 7 | PURIFY | PURIFY |
| 8 | YIELD N′-methyl-N′-(1-{2-[6-(methyloxy)-1,5-naphthyridin-4-yl]ethyl}-3-pyrrolidinyl)-3-oxo-3,4-dihydro-2H-pyrido[3,2-b][1,4]thiazine-6-carbohydrazide | YIELD N′-methyl-N′-(1-{2-[6-(methyloxy)-1,5-naphthyridin-4-yl]ethyl}-3-pyrrolidinyl)-3-oxo-3,4-dihydro-2H-pyrido[3,2-b][1,4]thiazine-6-carbohydrazide |

---

```
Reaction no 240
```

Generated by the Chemistry Development Kit (http://github.com/cdk)

|  | A | B |
| --- | --- | --- |
| 0 | ADD (9H-fluoren-9-yl)methyl ‌(2-((1R,2R,3S,3aR,8b S)-6-(((2S,3R,6R)-6-((R)-1,2-dihydroxyethyl)-3-methoxy-1,4-dioxan-2-yl)oxy)-1,8b-dihydroxy-8-methoxy-3a-(4-methoxyphenyl)-3-phenyl-2,3,3a,8b-tetrahydro-1H-cyclopenta[b]benzofuran-2-carboxamido)ethyl)carbamate | ADD (9H-fluoren-9-yl)methyl ‌(2-((1R,2R,3S,3aR,8b S)-6-(((2S,3R,6R)-6-((R)-1,2-dihydroxyethyl)-3-methoxy-1,4-dioxan-2-yl)oxy)-1,8b-dihydroxy-8-methoxy-3a-(4-methoxyphenyl)-3-phenyl-2,3,3a,8b-tetrahydro-1H-cyclopenta[b]benzofuran-2-carboxamido)ethyl)carbamate |
| 1 | ADD DMF | ADD DMF |
| 2 | ADD piperidine | ADD piperidine |
| 3 | STIR for 3600 s at 25 °C | STIR for 3600 s at 25 °C |
| 4 | PURIFY | CONCENTRATE |
| 5 | YIELD (1R,2R,3S,3aR,8bS)—N-(2-aminoethyl)-6-(((2S,3R,6R)-6-((R)-1,2-dihydroxyethyl)-3-methoxy-1,4-dioxan-2-yl)oxy)-1,8b-dihydroxy-8-methoxy-3a-(4-methoxyphenyl)-3-phenyl-2,3,3a,8b-tetrahydro-1H-cyclopenta[b]benzofuran-2-carboxamide | PURIFY |
| 6 |  | YIELD (1R,2R,3S,3aR,8bS)—N-(2-aminoethyl)-6-(((2S,3R,6R)-6-((R)-1,2-dihydroxyethyl)-3-methoxy-1,4-dioxan-2-yl)oxy)-1,8b-dihydroxy-8-methoxy-3a-(4-methoxyphenyl)-3-phenyl-2,3,3a,8b-tetrahydro-1H-cyclopenta[b]benzofuran-2-carboxamide |

---

```
Reaction no 241
```

Generated by the Chemistry Development Kit (http://github.com/cdk)

|  | A | B |
| --- | --- | --- |
| 0 | ADD 4-phenoxymethyl-benzaldehyde | ADD 4-phenoxymethyl-benzaldehyde |
| 1 | ADD Na2S2O4 | ADD 4-methyl-3-nitropyridine-2,6-diamine |
| 2 | ADD ethanol | ADD ethanol |
| 3 | REFLUX for 3600 s | ADD Na2S2O4 |
| 4 | SETTEMPERATURE 25 °C | MICROWAVE for 600 s at 100 °C |
| 5 | ADD 4-methyl-3-nitropyridine-2,6-diamine | SETTEMPERATURE 25 °C |
| 6 | STIR for 86400 s at 100 °C | ADD ammonia |
| 7 | SETTEMPERATURE 25 °C | STIR for 600 s |
| 8 | ADD ammonia | FILTER keep precipitate |
| 9 | STIR for 3600 s at 100 °C | PURIFY |
| 10 | SETTEMPERATURE 25 °C | YIELD 7-Methyl-2-(4-(phenoxymethyl)phenyl)-3H-imidazo[4,5-b]pyridin-5-amine |
| 11 | CONCENTRATE |  |
| 12 | PURIFY |  |
| 13 | YIELD 7-Methyl-2-(4-(phenoxymethyl)phenyl)-3H-imidazo[4,5-b]pyridin-5-amine |  |

---

```
Reaction no 242
```

Generated by the Chemistry Development Kit (http://github.com/cdk)

|  | A | B |
| --- | --- | --- |
| 0 | ADD rac-((1R,5S)-3-oxa-8-azabicyclo[3.2.1]octan-8-yl)((4bS,5R,6R,7S,7aR)-7a-(4-bromophenyl)-4b,5-dihydroxy-4-methoxy-7-phenyl-4b,6,7,7a-tetrahydro-5H-cyclopenta[4,5]furo[2,3-c]pyridin-6-yl)methanone | ADD rac-((1R,5S)-3-oxa-8-azabicyclo[3.2.1]octan-8-yl)((4bS,5R,6R,7S,7aR)-7a-(4-bromophenyl)-4b,5-dihydroxy-4-methoxy-7-phenyl-4b,6,7,7a-tetrahydro-5H-cyclopenta[4,5]furo[2,3-c]pyridin-6-yl)methanone |
| 1 | ADD methanol | ADD methanol |
| 2 | ADD water | ADD water |
| 3 | ADD LiOH | ADD LiOH |
| 4 | REFLUX for 28800 s | STIR for 28800 s at 25 °C |
| 5 | SETTEMPERATURE 0 °C | SETTEMPERATURE 0 °C |
| 6 | ADD $3$ / $4$ | PH with HCl to pH acidic |
| 7 | ADD HCl | FILTER keep precipitate |
| 8 | CONCENTRATE | DRYSOLID under vacuum |
| 9 | EXTRACT with dichloromethane | YIELD rac-(5aR,6S,7R,8R,8aS)-5a-(4-bromophenyl)-3-chloro-8,8a-dihydroxy-1-methoxy-6-phenyl-5a,7,8,8a-tetrahydro-6H-cyclopenta[4,5]furo[3,2-c]pyridine-7-carboxylic acid |
| 10 | COLLECTLAYER organic |  |
| 11 | WASH with brine |  |
| 12 | DRYSOLUTION over magnesium sulfate |  |
| 13 | FILTER keep filtrate |  |
| 14 | CONCENTRATE |  |
| 15 | DRYSOLID at 60 °C under vacuum |  |
| 16 | YIELD rac-(5aR,6S,7R,8R,8aS)-5a-(4-bromophenyl)-3-chloro-8,8a-dihydroxy-1-methoxy-6-phenyl-5a,7,8,8a-tetrahydro-6H-cyclopenta[4,5]furo[3,2-c]pyridine-7-carboxylic acid |  |

---

```
Reaction no 243
```

Generated by the Chemistry Development Kit (http://github.com/cdk)

|  | A | B |
| --- | --- | --- |
| 0 | ADD N-p-methoxybenzyl-N-tert.-butylthiomethyl-2-(S)-bromo-3-(R)-hydroxybutyramide | ADD m-chloroperbenzoic acid |
| 1 | ADD dichloromethane | MAKESOLUTION with N-p-methoxybenzyl-N-tert.-butylthiomethyl-2-(S)-bromo-3-(R)-hydroxybutyramide and dichloromethane |
| 2 | ADD m-chloroperbenzoic acid at 0 °C | ADD SLN |
| 3 | STIR for 3600 s at 0 °C | STIR |
| 4 | FILTER keep precipitate | STIR for 3600 s at 0 °C |
| 5 | WASH with NaHCO3 | FILTER keep filtrate |
| 6 | WASH with water | ADD dichloromethane |
| 7 | DRYSOLUTION over Na2SO4 | WASH with sodium bisulfite |
| 8 | CONCENTRATE | WASH with sodium bicarbonate |
| 9 | YIELD (2S,3R)-N-p-methoxybenzyl-N-tert.-butylsulphonylmethyl-2-bromo-3-hydroxybutyramide | COLLECTLAYER organic |
| 10 |  | DRYSOLUTION over Na2SO4 |
| 11 |  | CONCENTRATE |
| 12 |  | PURIFY |
| 13 |  | YIELD (2S,3R)-N-p-methoxybenzyl-N-tert.-butylsulphonylmethyl-2-bromo-3-hydroxybutyramide |

---

```
Reaction no 244
```

Generated by the Chemistry Development Kit (http://github.com/cdk)

|  | A | B |
| --- | --- | --- |
| 0 | ADD 1-tert-butyl 6-methyl 1H-indole-1,6-dicarboxylate | ADD 1-tert-butyl 6-methyl 1H-indole-1,6-dicarboxylate |
| 1 | ADD THF | ADD THF |
| 2 | ADD LDA at -70 °C | ADD LDA at -70 °C |
| 3 | STIR for 3600 s at -70 °C | STIR for 3600 s at -70 °C |
| 4 | ADD trimethyltin chloride | MAKESOLUTION with trimethyltin chloride and THF |
| 5 | STIR for 3600 s at -70 °C | ADD SLN |
| 6 | STIR for 3600 s at 25 °C | STIR for 600 s at 0 °C |
| 7 | QUENCH with ammonium chloride | PARTITION with ethyl acetate and ammonium chloride |
| 8 | EXTRACT with ethyl acetate | COLLECTLAYER organic |
| 9 | COLLECTLAYER organic | DRYSOLUTION over sodium sulfate |
| 10 | DRYSOLUTION over Na2SO4 | CONCENTRATE |
| 11 | FILTER keep filtrate | PURIFY |
| 12 | CONCENTRATE | YIELD 1-tert-butyl 6-methyl 2-(trimethylstannyl)-1H-indole-1,6-dicarboxylate |
| 13 | PURIFY |  |
| 14 | YIELD 1-tert-butyl 6-methyl 2-(trimethylstannyl)-1H-indole-1,6-dicarboxylate |  |

---

```
Reaction no 245
```

Generated by the Chemistry Development Kit (http://github.com/cdk)

|  | A | B |
| --- | --- | --- |
| 0 | ADD DCC | ADD 4,4-dimethyl-1,2,3,4-tetrahydro-2-oxo-7-quinolinecarboxylic acid |
| 1 | ADD aniline | ADD aniline |
| 2 | ADD dichloromethane | ADD DCC |
| 3 | ADD 4,4-dimethyl-1,2,3,4-tetrahydro-2-oxo-7-quinolinecarboxylic acid | ADD dichloromethane |
| 4 | STIR for 28800 s at 25 °C | STIR for 86400 s at 25 °C |
| 5 | FILTER keep precipitate | FILTER keep filtrate |
| 6 | RECRYSTALLIZE from ethanol | CONCENTRATE |
| 7 | YIELD 4,4-Dimethyl-1,2,3,4-tetrahydro-N-phenyl-2-oxo-7-quinolinecarboxamide | PURIFY |
| 8 |  | YIELD 4,4-Dimethyl-1,2,3,4-tetrahydro-N-phenyl-2-oxo-7-quinolinecarboxamide |

---

```
Reaction no 246
```

Generated by the Chemistry Development Kit (http://github.com/cdk)

|  | A | B |
| --- | --- | --- |
| 0 | ADD 4-Bromo-3-chloro-N-methoxy-N-methylbenzamide at -70 °C | ADD 4-Bromo-3-chloro-N-methoxy-N-methylbenzamide |
| 1 | ADD THF at -70 °C | ADD THF |
| 2 | ADD diisobutylaluminum hydride dropwise | ADD diisobutylaluminum hydride at -70 °C |
| 3 | STIR for 3600 s at -70 °C | STIR for 3600 s at -70 °C |
| 4 | QUENCH with water | QUENCH with ammonium chloride |
| 5 | COLLECTLAYER aqueous | EXTRACT with ethyl acetate |
| 6 | EXTRACT with ethyl acetate | COLLECTLAYER organic |
| 7 | COLLECTLAYER organic | WASH with brine |
| 8 | WASH with HCl | DRYSOLUTION over Na2SO4 |
| 9 | WASH with water | CONCENTRATE |
| 10 | WASH with brine | YIELD 4-bromo-3-chlorobenzaldehyde |
| 11 | DRYSOLUTION over magnesium sulfate |  |
| 12 | CONCENTRATE |  |
| 13 | DRYSOLID under vacuum |  |
| 14 | YIELD 4-bromo-3-chlorobenzaldehyde |  |

---

```
Reaction no 247
```

Generated by the Chemistry Development Kit (http://github.com/cdk)

|  | A | B |
| --- | --- | --- |
| 0 | ADD 1-phenyl-2-(1-(tetrahydro-2H-pyran-2-yl)-1H-indol-3-yl)ethan-1-one | ADD 1-phenyl-2-(1-(tetrahydro-2H-pyran-2-yl)-1H-indol-3-yl)ethan-1-one |
| 1 | ADD ammonium acetate | ADD methanol |
| 2 | ADD methanol | ADD ammonium acetate |
| 3 | STIR for 3600 s at 25 °C | STIR for 600 s at 0 °C |
| 4 | ADD sodium cyanoborohydride | ADD sodium cyanoborohydride |
| 5 | STIR for 86400 s at 25 °C | STIR for 28800 s at 60 °C |
| 6 | CONCENTRATE | QUENCH with water |
| 7 | PURIFY | EXTRACT with methanol |
| 8 | YIELD 1-phenyl-2-(1-(tetrahydro-2H-pyran-2-yl)-1H-indol-3-yl)ethan-1-amine | PHASESEPARATION |
| 9 |  | COLLECTLAYER organic |
| 10 |  | DRYSOLUTION over Na2SO4 |
| 11 |  | CONCENTRATE |
| 12 |  | PURIFY |
| 13 |  | YIELD 1-phenyl-2-(1-(tetrahydro-2H-pyran-2-yl)-1H-indol-3-yl)ethan-1-amine |

---

```
Reaction no 248
```

Generated by the Chemistry Development Kit (http://github.com/cdk)

|  | A | B |
| --- | --- | --- |
| 0 | ADD 5-chloro-1H-pyrrolo[2,3-c]pyridine | ADD 5-chloro-1H-pyrrolo[2,3-c]pyridine |
| 1 | ADD Cs2CO3 | ADD bromocyclobutane |
| 2 | ADD bromocyclobutane | ADD Cs2CO3 |
| 3 | ADD DMF | ADD DMF |
| 4 | STIR for 86400 s at 100 °C | STIR for 86400 s at 25 °C |
| 5 | SETTEMPERATURE 25 °C | ADD water |
| 6 | ADD water | EXTRACT with ethyl acetate |
| 7 | EXTRACT with ethyl acetate | COLLECTLAYER organic |
| 8 | COLLECTLAYER organic | WASH with brine |
| 9 | DRYSOLUTION over MgSO4 | DRYSOLUTION over magnesium sulfate |
| 10 | CONCENTRATE | CONCENTRATE |
| 11 | YIELD 5-chloro-1-cyclobutyl-1H-pyrrolo[2,3-c]pyridine-3-carbonitrile | PURIFY |
| 12 |  | YIELD 5-chloro-1-cyclobutyl-1H-pyrrolo[2,3-c]pyridine-3-carbonitrile |

---

```
Reaction no 249
```

Generated by the Chemistry Development Kit (http://github.com/cdk)

|  | A | B |
| --- | --- | --- |
| 0 | ADD amino-acridine 9 carboxylic acid | ADD amino-acridine 9 carboxylic acid |
| 1 | ADD N-phenyl-5-amino isatin | ADD N-phenyl-5-amino isatin |
| 2 | ADD KOH | ADD KOH |
| 3 | STIR for 86400 s at 100 °C | REFLUX for 86400 s |
| 4 | FILTER keep filtrate | SETTEMPERATURE 25 °C |
| 5 | PH with sulfuric acid to pH acidic | FILTER |
| 6 | FILTER keep precipitate | PH with sulfuric acid to pH acidic |
| 7 |  | FILTER keep precipitate |

---

```
Reaction no 250
```

Generated by the Chemistry Development Kit (http://github.com/cdk)

|  | A | B |
| --- | --- | --- |
| 0 | ADD sodium ‌(2-chloro-2,2-difluoro-acetyl)oxide | ADD sodium ‌(2-chloro-2,2-difluoro-acetyl)oxide |
| 1 | ADD NMP | ADD (5S,7S)-7-fluoro-5-(2-fluorophenyl)-6,7-dihydro-5H-pyrrolo[1,2-b][1,2,4]triazole-2-thiol |
| 2 | ADD (5S,7S)-7-fluoro-5-(2-fluorophenyl)-6,7-dihydro-5H-pyrrolo[1,2-b][1,2,4]triazole-2-thiol | ADD Cs2CO3 |
| 3 | ADD Cs2CO3 | ADD NMP |
| 4 | STIR for 3600 s at 100 °C | STIR for 28800 s at 100 °C |
| 5 | ADD water | ADD water |
| 6 | EXTRACT with ethyl acetate | EXTRACT with ethyl acetate |
| 7 | COLLECTLAYER organic | COLLECTLAYER organic |
| 8 | WASH with water | CONCENTRATE |
| 9 | WASH with brine | PURIFY |
| 10 | DRYSOLUTION over sodium sulfate | YIELD (5S,7S)-2-(difluoromethylsulfanyl)-7-fluoro-5-(2-fluorophenyl)-6,7-dihydro-5H-pyrrolo[1,2-b][1,2,4]triazole |
| 11 | CONCENTRATE |  |
| 12 | PURIFY |  |
| 13 | YIELD (5S,7S)-2-(difluoromethylsulfanyl)-7-fluoro-5-(2-fluorophenyl)-6,7-dihydro-5H-pyrrolo[1,2-b][1,2,4]triazole |  |

---

```
Reaction no 251
```

Generated by the Chemistry Development Kit (http://github.com/cdk)

|  | A | B |
| --- | --- | --- |
| 0 | ADD 7-hydroxy-2-naphthonitrile | ADD 7-hydroxy-2-naphthonitrile |
| 1 | ADD Na2CO3 | ADD THF |
| 2 | ADD iodine | ADD water |
| 3 | ADD water | ADD Na2CO3 at 0 °C |
| 4 | ADD THF | ADD iodine at 0 °C |
| 5 | STIR for 28800 s at 0 °C | STIR for 3600 s at 0 °C |
| 6 | PH with HCl to pH acidic | ADD HCl |
| 7 | EXTRACT with ethyl acetate | EXTRACT with ethyl acetate |
| 8 | WASH with sodium thiosulfate | COLLECTLAYER organic |
| 9 | WASH with brine | DRYSOLUTION over sodium sulfate |
| 10 | DRYSOLUTION over Na2SO4 | FILTER keep filtrate |
| 11 | CONCENTRATE | CONCENTRATE |
| 12 | RECRYSTALLIZE from ethyl acetate | PURIFY |
| 13 | YIELD 7-hydroxy-8-iodo-2-naphthalenecarbonitrile | YIELD 7-hydroxy-8-iodo-2-naphthalenecarbonitrile |

---

```
Reaction no 252
```

Generated by the Chemistry Development Kit (http://github.com/cdk)

|  | A | B |
| --- | --- | --- |
| 0 | ADD 4-(bromomethyl)benzene-1-sulfonyl chloride | ADD 4-(bromomethyl)benzene-1-sulfonyl chloride |
| 1 | ADD dichloromethane | ADD dichloromethane |
| 2 | ADD dimethylamine at 0 °C | ADD dimethylamine at 0 °C |
| 3 | STIR for 3600 s at 0 °C | STIR for 86400 s at 25 °C |
| 4 | PARTITION with HCl and dichloromethane | QUENCH with water |
| 5 | COLLECTLAYER organic | EXTRACT with dichloromethane |
| 6 | WASH with HCl | COLLECTLAYER organic |
| 7 | WASH with water | WASH with brine |
| 8 | DRYSOLUTION over Na2SO4 | DRYSOLUTION over Na2SO4 |
| 9 | CONCENTRATE | CONCENTRATE |
| 10 | YIELD 4-(bromomethyl)-N,N-dimethylbenzenesulfonamide | YIELD 4-(bromomethyl)-N,N-dimethylbenzenesulfonamide |

---

```
Reaction no 253
```

Generated by the Chemistry Development Kit (http://github.com/cdk)

|  | A | B |
| --- | --- | --- |
| 0 | ADD 2,6-Dimethoxy-5-nitro-4-pyrimidinamine | ADD 2,6-Dimethoxy-5-nitro-4-pyrimidinamine |
| 1 | ADD 2-methoxycarbonylbenzenesulfonylisocyanate | ADD dichloromethane |
| 2 | ADD dichloromethane | MAKESOLUTION with 2-methoxycarbonylbenzenesulfonylisocyanate and dichloromethane |
| 3 | STIR for 86400 s at 25 °C | ADD SLN |
| 4 | CONCENTRATE | STIR for 86400 s at 25 °C |
| 5 | ADD 1-chlorobutane | FILTER keep precipitate |
| 6 | FILTER keep precipitate | WASH with 1-chlorobutane |
| 7 | WASH with ether | YIELD 2-[[(2,6-Dimethoxy-5-nitropyrimidin-4-yl)aminocarbonyl]aminosulfonyl]benzoic acid, methyl ester |
| 8 | DRYSOLID |  |
| 9 | YIELD 2-[[(2,6-Dimethoxy-5-nitropyrimidin-4-yl)aminocarbonyl]aminosulfonyl]benzoic acid, methyl ester |  |

---

```
Reaction no 254
```

Generated by the Chemistry Development Kit (http://github.com/cdk)

|  | A | B |
| --- | --- | --- |
| 0 | ADD 2-fluorophenylacetic acid | ADD 2-fluorophenylacetic acid |
| 1 | ADD sulfuric acid | ADD sulfuric acid |
| 2 | MAKESOLUTION with nitric acid and sulfuric acid | SETTEMPERATURE 0 °C |
| 3 | ADD SLN dropwise at 0 °C | MAKESOLUTION with nitric acid and sulfuric acid |
| 4 | STIR for 3600 s at 0 °C | ADD SLN dropwise at 0 °C |
| 5 | ADD ice water | STIR for 600 s at 0 °C |
| 6 | FILTER keep precipitate | ADD water |
| 7 | WASH with water | FILTER keep precipitate |
| 8 | DRYSOLID | DRYSOLID under vacuum |
| 9 | YIELD 2-(2-fluoro-5-nitrophenyl)acetic acid | YIELD 2-(2-fluoro-5-nitrophenyl)acetic acid |

---

```
Reaction no 255
```

Generated by the Chemistry Development Kit (http://github.com/cdk)

|  | A | B |
| --- | --- | --- |
| 0 | ADD 2-Chloromethyl-3-methoxy-4-(2-thienylmethylthio)pyridine hydrochloride | ADD 2-mercaptobenzimidazole |
| 1 | ADD 2-mercaptobenzimidazole | ADD ethanol |
| 2 | ADD NaOH | ADD NaOH |
| 3 | ADD ethanol | ADD 2-Chloromethyl-3-methoxy-4-(2-thienylmethylthio)pyridine hydrochloride |
| 4 | STIR for 86400 s at 25 °C | STIR for 86400 s at 25 °C |
| 5 | RECRYSTALLIZE from toluene / methanol | CONCENTRATE |
| 6 | YIELD 2-{[[3-Methoxy-4-(2-thienylmethylthio]-2-pyridinyl]methyl]thio}-1H-benzimidazole | PURIFY |
| 7 |  | YIELD 2-{[[3-Methoxy-4-(2-thienylmethylthio]-2-pyridinyl]methyl]thio}-1H-benzimidazole |

---

```
Reaction no 256
```

Generated by the Chemistry Development Kit (http://github.com/cdk)

|  | A | B |
| --- | --- | --- |
| 0 | ADD Ethyl 2-(2-{[(allyloxy)carbonyl]amino}-1,3-thiazol-4-yl)-3-oxopropanoate | ADD Ethyl 2-(2-{[(allyloxy)carbonyl]amino}-1,3-thiazol-4-yl)-3-oxopropanoate |
| 1 | ADD THF | ADD THF |
| 2 | ADD ethanol | ADD ethanol |
| 3 | ADD water | ADD water |
| 4 | ADD sodium borohydride at 0 °C | ADD sodium borohydride at 0 °C |
| 5 | ADD sodium borohydride over 3600 s | STIR for 3600 s at 0 °C |
| 6 | STIR | ADD water |
| 7 | ADD sodium borohydride over 3600 s | EXTRACT with ethyl acetate |
| 8 | STIR for 86400 s | WASH with sodium chloride |
| 9 | CONCENTRATE | DRYSOLUTION over magnesium sulfate |
| 10 | PURIFY | CONCENTRATE |
| 11 | YIELD allyl {4-[2-hydroxy-1-(hydroxymethyl)ethyl]-1,3-thiazol-2-yl}carbamate | PURIFY |
| 12 |  | YIELD allyl {4-[2-hydroxy-1-(hydroxymethyl)ethyl]-1,3-thiazol-2-yl}carbamate |

---

```
Reaction no 257
```

Generated by the Chemistry Development Kit (http://github.com/cdk)

|  | A | B |
| --- | --- | --- |
| 0 | ADD 4-(4,4,5,5-tetramethyl-1,3,2-dioxaborolan-2-yl)benzoic acid | ADD 4-(4,4,5,5-tetramethyl-1,3,2-dioxaborolan-2-yl)benzoic acid |
| 1 | ADD thionyl chloride | ADD thionyl chloride |
| 2 | REFLUX for 3600 s | REFLUX for 3600 s |
| 3 | CONCENTRATE | CONCENTRATE |
| 4 | ADD dichloromethane | ADD dichloromethane |
| 5 | ADD tetrahydro-2H-pyran-4-amine | ADD tetrahydro-2H-pyran-4-amine |
| 6 | ADD triethylamine | ADD triethylamine |
| 7 | STIR for 3600 s at 25 °C | STIR for 28800 s at 25 °C |
| 8 | ADD water | ADD water |
| 9 | EXTRACT with dichloromethane | EXTRACT with dichloromethane |
| 10 | COLLECTLAYER organic | COLLECTLAYER organic |
| 11 | DRYSOLUTION over Na2SO4 | WASH with water |
| 12 | CONCENTRATE | WASH with brine |
| 13 | YIELD 4-(4,4,5,5-tetramethyl-[1,3,2]dioxaborolan-2-yl)-N-(tetrahydropyran-4-yl)benzamide | DRYSOLUTION over magnesium sulfate |
| 14 |  | CONCENTRATE |
| 15 |  | PURIFY |
| 16 |  | YIELD 4-(4,4,5,5-tetramethyl-[1,3,2]dioxaborolan-2-yl)-N-(tetrahydropyran-4-yl)benzamide |

---

```
Reaction no 258
```

Generated by the Chemistry Development Kit (http://github.com/cdk)

|  | A | B |
| --- | --- | --- |
| 0 | ADD 6-(1,5-dimethyl-6-oxo-1,6-dihydropyridin-3-yl)imidazo[1,2-a]pyridine-3-carboxylic Acid | ADD 6-(1,5-dimethyl-6-oxo-1,6-dihydropyridin-3-yl)imidazo[1,2-a]pyridine-3-carboxylic Acid |
| 1 | ADD DMF | ADD DMF |
| 2 | ADD cyclohexylamine | ADD HATU at 25 °C |
| 3 | ADD HATU | STIR for 3600 s at 25 °C |
| 4 | ADD DIPEA | MAKESOLUTION with cyclohexylamine and DIPEA and DMF |
| 5 | STIR for 86400 s at 25 °C | ADD SLN dropwise |
| 6 | ADD water | STIR for 86400 s at 25 °C |
| 7 | PURIFY | ADD water |
| 8 | YIELD N-cyclohexyl-6-(1,5-dimethyl-6-oxo-1,6-dihydropyridin-3-yl)imidazo[1,2-a] pyridine-3-carboxamide | FILTER keep precipitate |
| 9 |  | DRYSOLID under vacuum |
| 10 |  | PURIFY |
| 11 |  | YIELD N-cyclohexyl-6-(1,5-dimethyl-6-oxo-1,6-dihydropyridin-3-yl)imidazo[1,2-a] pyridine-3-carboxamide |

---

```
Reaction no 259
```

Generated by the Chemistry Development Kit (http://github.com/cdk)

|  | A | B |
| --- | --- | --- |
| 0 | ADD (2S,5S)-5-(methoxycarbonylamino)-4-oxo-1,2,4,5,6,7-hexahydroazepino[3,2,1-hi]indole-2-carboxylic acid | ADD (2S,5S)-5-(methoxycarbonylamino)-4-oxo-1,2,4,5,6,7-hexahydroazepino[3,2,1-hi]indole-2-carboxylic acid |
| 1 | ADD 2-amino-1-(4-(6-bromoquinoxalin-2-yl)phenyl)ethanone 2,2,2-trifluoroacetate | ADD 2-amino-1-(4-(6-bromoquinoxalin-2-yl)phenyl)ethanone 2,2,2-trifluoroacetate |
| 2 | ADD HATU | ADD HATU |
| 3 | ADD DMF | ADD DMF |
| 4 | ADD (2S,5S)-5-(methoxycarbonylamino)-4-oxo-1,2,4,5,6,7-hexahydroazepino[3,2,1-hi]indole-2-carboxylic acid at 25 °C | ADD triethylamine at 0 °C |
| 5 | ADD dichloromethane dropwise | STIR for 3600 s at 25 °C |
| 6 | STIR at 25 °C | ADD dichloromethane |
| 7 | CONCENTRATE | WASH with water |
| 8 | TRITURATE with methanol | WASH with NaHCO3 |
| 9 | FILTER keep precipitate | COLLECTLAYER organic |
| 10 | WASH with ether | DRYSOLUTION over Na2SO4 |
| 11 | DRYSOLID | FILTER keep filtrate |
| 12 | YIELD methyl ‌(2S,5S)-2-(2-(4-(6-bromoquinoxalin-2-yl)phenyl)-2-oxoethylcarbamoyl)-4-oxo-1,2,4,5,6,7-hexahydroazepino[3,2,1-hi]indol-5-ylcarbamate | CONCENTRATE |
| 13 |  | PURIFY |
| 14 |  | YIELD methyl ‌(2S,5S)-2-(2-(4-(6-bromoquinoxalin-2-yl)phenyl)-2-oxoethylcarbamoyl)-4-oxo-1,2,4,5,6,7-hexahydroazepino[3,2,1-hi]indol-5-ylcarbamate |

---

```
Reaction no 260
```

Generated by the Chemistry Development Kit (http://github.com/cdk)

|  | A | B |
| --- | --- | --- |
| 0 | ADD tert-butyl ‌(1S,4S)-7-benzamido-4-(3,4-dichlorophenyl)-1,2,3,4-tetrahydrona-phthalen-1-yl(methyl)carbamate | ADD tert-butyl ‌(1S,4S)-7-benzamido-4-(3,4-dichlorophenyl)-1,2,3,4-tetrahydrona-phthalen-1-yl(methyl)carbamate |
| 1 | ADD dichloromethane | ADD dichloromethane |
| 2 | ADD TFA | ADD TFA |
| 3 | STIR for 3600 s at 25 °C | STIR for 28800 s |
| 4 | CONCENTRATE | ADD dichloromethane |
| 5 | PURIFY | WASH with sodium bicarbonate |
| 6 | YIELD N-((5S,8S)-5-(3,4-dichlorophenyl)-8-(methylamino)-5,6,7,8-tetrahydronaphthalen-2-yl)benzamide | WASH with brine |
| 7 |  | PHASESEPARATION |
| 8 |  | COLLECTLAYER organic |
| 9 |  | DRYSOLUTION over Na2SO4 |
| 10 |  | CONCENTRATE |
| 11 |  | PURIFY |
| 12 |  | YIELD N-((5S,8S)-5-(3,4-dichlorophenyl)-8-(methylamino)-5,6,7,8-tetrahydronaphthalen-2-yl)benzamide |

---

```
Reaction no 261
```

Generated by the Chemistry Development Kit (http://github.com/cdk)

|  | A | B |
| --- | --- | --- |
| 0 | MAKESOLUTION with 3-hydroxy-5-n-butoxy-1,2,4-thiadiazole and THF | ADD 3-hydroxy-5-n-butoxy-1,2,4-thiadiazole |
| 1 | ADD triethylamine | ADD triethylamine |
| 2 | ADD SLN | ADD THF |
| 3 | ADD ethyl isocyanate dropwise | ADD ethyl isocyanate |
| 4 | STIR for 3600 s at 25 °C | STIR for 86400 s at 25 °C |
| 5 | CONCENTRATE | CONCENTRATE |
| 6 | PURIFY | PURIFY |
| 7 | YIELD 2-(N-ethylcarbamoyl)-5-n-butoxy-1,2,4-thiadiazole-3-one | YIELD 2-(N-ethylcarbamoyl)-5-n-butoxy-1,2,4-thiadiazole-3-one |

---

```
Reaction no 262
```

Generated by the Chemistry Development Kit (http://github.com/cdk)

|  | A | B |
| --- | --- | --- |
| 0 | ADD Methyl 4-(4-t-Butylbenzoyl)aminopyridine-3-carboxylate | ADD Methyl 4-(4-t-Butylbenzoyl)aminopyridine-3-carboxylate |
| 1 | ADD THF | ADD THF |
| 2 | ADD methanol | ADD methanol |
| 3 | ADD LiOH | ADD LiOH |
| 4 | STIR for 86400 s at 25 °C | WAIT for 3600 s |
| 5 | CONCENTRATE | CONCENTRATE |
| 6 | PH with HCl to pH acidic | PARTITION with water and ether |
| 7 | FILTER keep precipitate | PHASESEPARATION |
| 8 | WASH with water | COLLECTLAYER aqueous |
| 9 | DRYSOLID under vacuum | PH with citric acid to pH acidic |
| 10 | YIELD 4-(4-t-Butylbenzoyl)aminopyridine-3-carboxylic Acid | FILTER keep precipitate |
| 11 |  | WASH with water |
| 12 |  | WASH with isopropanol / chloroform |
| 13 |  | WASH with ether |
| 14 |  | DRYSOLID under vacuum |
| 15 |  | YIELD 4-(4-t-Butylbenzoyl)aminopyridine-3-carboxylic Acid |

---

```
Reaction no 263
```

Generated by the Chemistry Development Kit (http://github.com/cdk)

|  | A | B |
| --- | --- | --- |
| 0 | ADD ethanol | ADD ethanol |
| 1 | ADD NaHCO3 | ADD NaHCO3 |
| 2 | ADD hydroxylamine hydrochloride | ADD hydroxylamine hydrochloride |
| 3 | REFLUX for 3600 s | REFLUX for 3600 s |
| 4 | SETTEMPERATURE 25 °C | SETTEMPERATURE 25 °C |
| 5 | ADD 4-(2,2,2-trifluoroethyl)pyridine-2-carbonitrile at 0 °C | ADD 4-(2,2,2-trifluoroethyl)pyridine-2-carbonitrile at 0 °C |
| 6 | STIR for 28800 s | STIR for 28800 s |
| 7 | CONCENTRATE | CONCENTRATE |
| 8 | ADD water | ADD water |
| 9 | EXTRACT with ethyl acetate | EXTRACT with ethyl acetate |
| 10 | COLLECTLAYER organic | COLLECTLAYER organic |
| 11 | WASH with sodium chloride | WASH with sodium chloride |
| 12 | DRYSOLUTION over magnesium sulfate | DRYSOLUTION over magnesium sulfate |
| 13 | CONCENTRATE | CONCENTRATE |
| 14 | PURIFY | PURIFY |
| 15 | YIELD 4-(2,2,2-trifluoroethyl)pyridine-2-carboxamide | YIELD 4-(2,2,2-trifluoroethyl)pyridine-2-carboxamide |

---

```
Reaction no 264
```

Generated by the Chemistry Development Kit (http://github.com/cdk)

|  | A | B |
| --- | --- | --- |
| 0 | ADD tert-Butyl N-[2-[(2-hydroxy-3,3-diphenyl-propyl)-(2-methoxy-1-methyl-ethyl)amino]-2-oxo-ethyl]carbamate | ADD tert-Butyl N-[2-[(2-hydroxy-3,3-diphenyl-propyl)-(2-methoxy-1-methyl-ethyl)amino]-2-oxo-ethyl]carbamate |
| 1 | ADD dichloromethane | ADD dichloromethane |
| 2 | MAKESOLUTION with Dess-Martin periodinane and dichloromethane | ADD isopropanol |
| 3 | ADD SLN at 0 °C | ADD Dess-Martin periodinane |
| 4 | STIR for 86400 s at 25 °C | STIR for 3600 s at 25 °C |
| 5 | ADD isopropanol | QUENCH with Na2S2O4 |
| 6 | STIR for 3600 s | ADD dichloromethane |
| 7 | WASH with sodium bicarbonate | WASH with NaHCO3 |
| 8 | COLLECTLAYER organic | WASH with brine |
| 9 | DRYSOLUTION | DRYSOLUTION over Na2SO4 |
| 10 | CONCENTRATE | FILTER keep filtrate |
| 11 | PURIFY | CONCENTRATE |
| 12 | YIELD tert-Butyl N-[2-[(2-methoxy-1-methyl-ethyl)-(2-oxo-3,3-diphenyl-propyl)amino]-2-oxo-ethyl]carbamate | PURIFY |
| 13 |  | YIELD tert-Butyl N-[2-[(2-methoxy-1-methyl-ethyl)-(2-oxo-3,3-diphenyl-propyl)amino]-2-oxo-ethyl]carbamate |

---

```
Reaction no 265
```

Generated by the Chemistry Development Kit (http://github.com/cdk)

|  | A | B |
| --- | --- | --- |
| 0 | ADD 2,6-dichloro-3-nitropyridine | ADD methanol |
| 1 | ADD methanol | ADD 2,6-dichloro-3-nitropyridine |
| 2 | ADD triethylamine | ADD triethylamine |
| 3 | ADD p-toluidine | ADD p-toluidine |
| 4 | STIR for 3600 s at 25 °C | STIR for 28800 s at 25 °C |
| 5 | ADD water | ADD water |
| 6 | FILTER keep precipitate | STIR for 3600 s at 25 °C |
| 7 | WASH with water | FILTER keep precipitate |
| 8 | DRYSOLID | WASH with methanol |
| 9 | YIELD 6-chloro-3-nitro-pyridin-2-yl-p-tolyl-amine | WASH with water |
| 10 |  | DRYSOLID at 60 °C |
| 11 |  | YIELD 6-chloro-3-nitro-pyridin-2-yl-p-tolyl-amine |

---

```
Reaction no 266
```

Generated by the Chemistry Development Kit (http://github.com/cdk)

|  | A | B |
| --- | --- | --- |
| 0 | ADD [1-benzyl-4-(6-bromo-2-pyridinyl)-4-piperidinyl]-phenyl-amine | ADD [1-benzyl-4-(6-bromo-2-pyridinyl)-4-piperidinyl]-phenyl-amine |
| 1 | ADD acetic anhydride | ADD acetic anhydride |
| 2 | REFLUX for 86400 s | STIR for 86400 s at 100 °C |
| 3 | CONCENTRATE | CONCENTRATE |
| 4 | ADD sodium bicarbonate | ADD sodium bicarbonate |
| 5 | EXTRACT with ethyl acetate | EXTRACT with ethyl acetate |
| 6 | WASH with brine | COLLECTLAYER organic |
| 7 | DRYSOLUTION over magnesium sulfate | WASH with sodium chloride |
| 8 | CONCENTRATE | DRYSOLUTION over magnesium sulfate |
| 9 | PURIFY | CONCENTRATE |
| 10 | FILTER keep precipitate | PURIFY |
| 11 | CONCENTRATE | FILTER keep precipitate |
| 12 | YIELD N-[1-benzyl-4-(6-bromo-2-pyridinyl)-4-piperidinyl]-N-phenylacetamide | CONCENTRATE |
| 13 |  | YIELD N-[1-benzyl-4-(6-bromo-2-pyridinyl)-4-piperidinyl]-N-phenylacetamide |

---

```
Reaction no 267
```

Generated by the Chemistry Development Kit (http://github.com/cdk)

|  | A | B |
| --- | --- | --- |
| 0 | ADD 4-[1-(4-Amino-3-methyl-1H-pyrazolo[3,4-d]pyrimidin-1-yl)ethyl]-2-azetidin-3-yl-6-chloro-3-methoxybenzonitrile | ADD 4-[1-(4-Amino-3-methyl-1H-pyrazolo[3,4-d]pyrimidin-1-yl)ethyl]-2-azetidin-3-yl-6-chloro-3-methoxybenzonitrile |
| 1 | ADD methanol | ADD methanol |
| 2 | ADD hydroxyacetone | ADD sodium cyanoborohydride |
| 3 | ADD acetic acid | ADD hydroxyacetone |
| 4 | ADD sodium cyanoborohydride | STIR for 3600 s at 25 °C |
| 5 | STIR for 86400 s at 25 °C | QUENCH with acetic acid |
| 6 | CONCENTRATE | ADD methanol |
| 7 | PURIFY | PURIFY |
| 8 | YIELD 4-[1-(4-amino-3-methyl-1H-pyrazolo[3,4-d]pyrimidin-1-yl)ethyl]-6-chloro-2-[1-(2-hydroxy-1-methylethyl)azetidin-3-yl]-3-methoxybenzonitrile | YIELD 4-[1-(4-amino-3-methyl-1H-pyrazolo[3,4-d]pyrimidin-1-yl)ethyl]-6-chloro-2-[1-(2-hydroxy-1-methylethyl)azetidin-3-yl]-3-methoxybenzonitrile |
| 9 |  | PHASESEPARATION |
| 10 |  | COLLECTLAYER organic |
| 11 |  | PURIFY |

---

```
Reaction no 268
```

Generated by the Chemistry Development Kit (http://github.com/cdk)

|  | A | B |
| --- | --- | --- |
| 0 | ADD 3β-acetoxy-5α,25-dihydroxy cholestane | ADD 3β-acetoxy-5α,25-dihydroxy cholestane |
| 1 | ADD methanol | ADD dioxane |
| 2 | ADD dioxane | ADD methanol |
| 3 | ADD NaOH | ADD NaOH |
| 4 | REFLUX for 28800 s | STIR for 86400 s at 25 °C |
| 5 | PH with HCl to pH neutral | ADD HCl |
| 6 | EXTRACT with ether | EXTRACT with ethyl acetate |
| 7 | WASH with water | COLLECTLAYER organic |
| 8 | CONCENTRATE | WASH with brine |
| 9 | RECRYSTALLIZE from ether | DRYSOLUTION over sodium sulfate |
| 10 | YIELD 3β, 5α,25-tri-hydroxy-cholestane | CONCENTRATE |
| 11 |  | YIELD 3β, 5α,25-tri-hydroxy-cholestane |

---

```
Reaction no 269
```

Generated by the Chemistry Development Kit (http://github.com/cdk)

|  | A | B |
| --- | --- | --- |
| 0 | ADD 7-[1-(tert-butoxycarbonyl)piperidin-4-yl]-5-oxo-4,5-dihydropyrazolo[1,5-a]pyrimidine-3-carboxylic acid | ADD 7-[1-(tert-butoxycarbonyl)piperidin-4-yl]-5-oxo-4,5-dihydropyrazolo[1,5-a]pyrimidine-3-carboxylic acid |
| 1 | ADD 2-[4-(trifluoromethoxy)phenyl]acetohydrazide hydrochloride | ADD 2-[4-(trifluoromethoxy)phenyl]acetohydrazide hydrochloride |
| 2 | ADD DMF | ADD DMF |
| 3 | ADD DIPEA | ADD DIPEA |
| 4 | ADD HATU | ADD HATU |
| 5 | STIR for 86400 s at 25 °C | STIR for 86400 s at 25 °C |
| 6 | PURIFY | PURIFY |
| 7 | DRYSOLID under vacuum | DRYSOLID under vacuum |
| 8 | YIELD Tert-butyl 4-{5-oxo-3-[(2-{[4-(trifluoromethoxy)phenyl]acetyl}hydrazinyl)carbonyl]-4,5-dihydropyrazolo[1,5-a]pyrimidin-7-yl}piperidine-1-carboxylate | YIELD Tert-butyl 4-{5-oxo-3-[(2-{[4-(trifluoromethoxy)phenyl]acetyl}hydrazinyl)carbonyl]-4,5-dihydropyrazolo[1,5-a]pyrimidin-7-yl}piperidine-1-carboxylate |

---

```
Reaction no 270
```

Generated by the Chemistry Development Kit (http://github.com/cdk)

|  | A | B |
| --- | --- | --- |
| 0 | ADD 5-(1-Methylcyclopropyl)-1,3,4-oxadiazol-2(3H)-one | ADD 5-(1-Methylcyclopropyl)-1,3,4-oxadiazol-2(3H)-one |
| 1 | ADD triphenyltin chloride | ADD triphenyltin chloride |
| 2 | ADD dichloromethane | ADD triethylamine |
| 3 | ADD triethylamine | ADD dichloromethane |
| 4 | ADD dichloromethane | STIR for 86400 s at 25 °C |
| 5 | STIR for 86400 s at 25 °C | CONCENTRATE |
| 6 | REFLUX for 28800 s | PURIFY |
| 7 | SETTEMPERATURE 25 °C | YIELD 2-(1-Methylcyclopropyl)-4-Triphenylstannyl-5-Oxo-1,3,4-Oxadiazoline |
| 8 | STIR for 3600 s at 25 °C |  |
| 9 | WASH with water |  |
| 10 | EXTRACT with dichloromethane |  |
| 11 | COLLECTLAYER organic |  |
| 12 | DRYSOLUTION |  |
| 13 | WASH with hexane |  |
| 14 | YIELD 2-(1-Methylcyclopropyl)-4-Triphenylstannyl-5-Oxo-1,3,4-Oxadiazoline |  |

---

```
Reaction no 271
```

Generated by the Chemistry Development Kit (http://github.com/cdk)

|  | A | B |
| --- | --- | --- |
| 0 | ADD N-(2,4-Dimethoxybenzyl)-4-methyl-N-(pyridin-2-yl)benzenesulfonamide | ADD N-(2,4-Dimethoxybenzyl)-4-methyl-N-(pyridin-2-yl)benzenesulfonamide |
| 1 | ADD triphenylphosphine | ADD acetonitrile |
| 2 | ADD acetonitrile | ADD triphenylphosphine |
| 3 | ADD HCl | ADD HCl |
| 4 | STIR for 3600 s at 60 °C | STIR for 86400 s at 25 °C |
| 5 | SETTEMPERATURE 25 °C | CONCENTRATE |
| 6 | CONCENTRATE | PURIFY |
| 7 | ADD water | YIELD 4-Methyl-N-(pyridin-2-yl)benzenesulfonamide |
| 8 | PH with sodium hydroxide to pH acidic |  |
| 9 | STIR for 3600 s |  |
| 10 | FILTER keep precipitate |  |
| 11 | DRYSOLID under vacuum |  |
| 12 | YIELD 4-Methyl-N-(pyridin-2-yl)benzenesulfonamide |  |

---

```
Reaction no 272
```

Generated by the Chemistry Development Kit (http://github.com/cdk)

|  | A | B |
| --- | --- | --- |
| 0 | ADD N-Boc-(R)-2-(3-(2-(3-pyridinyl)vinyl)-benzyl)morpholine | ADD NaOH |
| 1 | ADD N-Boc-(R)-2-(5-bromo-2-hydroxybenzyl)morpholine | ADD water |
| 2 | ADD dioxane | ADD tetrabutylammonium bromide |
| 3 | ADD NaOH | MAKESOLUTION with N-Boc-(R)-2-(5-bromo-2-hydroxybenzyl)morpholine and N-Boc-(R)-2-(3-(2-(3-pyridinyl)vinyl)-benzyl)morpholine and dioxane |
| 4 | ADD tetrabutylammonium bromide | ADD SLN |
| 5 | STIR for 28800 s at 25 °C | SETTEMPERATURE 25 °C |
| 6 | ADD chlorodifluoromethane | ADD chlorodifluoromethane |
| 7 | ADD water | WAIT for 600 s |
| 8 | COLLECTLAYER organic | STIR for 3600 s at 25 °C |
| 9 | DRYSOLUTION over Na2SO4 | STIR for 86400 s |
| 10 | CONCENTRATE | CONCENTRATE |
| 11 | PURIFY | PARTITION with ethyl acetate and water |
| 12 | YIELD N-Boc-(R)-2-(5-bromo-2-difluoromethoxy-benzyl)-morpholine | PURIFY |
| 13 |  | YIELD N-Boc-(R)-2-(5-bromo-2-difluoromethoxy-benzyl)-morpholine |

---

```
Reaction no 273
```

Generated by the Chemistry Development Kit (http://github.com/cdk)

|  | A | B |
| --- | --- | --- |
| 0 | ADD 4-bromo-1,3-dimethyl-1H-pyrazole-5-carboxylic acid | ADD 4-bromo-1,3-dimethyl-1H-pyrazole-5-carboxylic acid |
| 1 | ADD DMF | ADD DMF |
| 2 | ADD ammonium chloride | ADD HOBt |
| 3 | ADD HOBt | ADD 1,2-dichloroethane |
| 4 | ADD 1,2-dichloroethane | STIR for 600 s at 25 °C |
| 5 | ADD DIPEA | ADD ammonium chloride |
| 6 | STIR for 86400 s at 25 °C | ADD DIPEA |
| 7 | ADD ethyl acetate | STIR for 86400 s at 25 °C |
| 8 | WASH with brine | ADD ethyl acetate |
| 9 | DRYSOLUTION over Na2SO4 | WASH with brine |
| 10 | CONCENTRATE | DRYSOLUTION over Na2SO4 |
| 11 | YIELD 4-bromo-1,3-dimethyl-1H-pyrazole-5-carboxamide | FILTER keep filtrate |
| 12 |  | CONCENTRATE |
| 13 |  | YIELD 4-bromo-1,3-dimethyl-1H-pyrazole-5-carboxamide |

---

```
Reaction no 274
```

Generated by the Chemistry Development Kit (http://github.com/cdk)

|  | A | B |
| --- | --- | --- |
| 0 | ADD 3-(2-cyclopropyl-4-iodo-1H-imidazol-1-yl)bicyclo[1.1.1]pentan-1-amine bis(2,2,2-trifluoroacetate) | ADD 3-(2-cyclopropyl-4-iodo-1H-imidazol-1-yl)bicyclo[1.1.1]pentan-1-amine bis(2,2,2-trifluoroacetate) |
| 1 | ADD 1,5-dibromopentan-3-one | ADD acetonitrile |
| 2 | ADD K2CO3 | ADD K2CO3 |
| 3 | ADD acetonitrile | ADD 1,5-dibromopentan-3-one |
| 4 | STIR for 86400 s at 25 °C | STIR for 86400 s at 60 °C |
| 5 | FILTER keep filtrate | FILTER keep filtrate |
| 6 | CONCENTRATE | CONCENTRATE |
| 7 | PURIFY | PURIFY |
| 8 | YIELD 1-(3-(2-cyclopropyl-4-iodo-1H-imidazol-1-yl)bicyclo[1.1.1]pentan-1-yl)piperidin-4-one | YIELD 1-(3-(2-cyclopropyl-4-iodo-1H-imidazol-1-yl)bicyclo[1.1.1]pentan-1-yl)piperidin-4-one |

---

```
Reaction no 275
```

Generated by the Chemistry Development Kit (http://github.com/cdk)

|  | A | B |
| --- | --- | --- |
| 0 | ADD (5R)-5-(2,2-dimethyl-4H-1,3-benzodioxin-6-yl)-3-{2-[3-(hydroxymethyl)phenyl]ethyl}-1,3-oxazolidin-2-one | ADD (5R)-5-(2,2-dimethyl-4H-1,3-benzodioxin-6-yl)-3-{2-[3-(hydroxymethyl)phenyl]ethyl}-1,3-oxazolidin-2-one |
| 1 | ADD DMF | ADD 2-(3-bromopropyl)-1,3-dichlorobenzene |
| 2 | ADD NaH | ADD DMF |
| 3 | MAKESOLUTION with 2-(3-bromopropyl)-1,3-dichlorobenzene and DMF | ADD NaH |
| 4 | ADD SLN | STIR for 86400 s at 25 °C |
| 5 | STIR for 604800 s at 25 °C | ADD dichloromethane |
| 6 | ADD water | ADD water |
| 7 | ADD dichloromethane | COLLECTLAYER organic |
| 8 | PHASESEPARATION | CONCENTRATE |
| 9 | COLLECTLAYER organic | PURIFY |
| 10 | CONCENTRATE | YIELD (5R)-3-[2-(3-{[3-(2,6-Dichlorophenyl)propoxy]methyl}phenyl)ethyl]-5-(2,2-dimethyl-4H-1,3-benzodioxin-6-yl)-1,3-oxazolidin-2-one |
| 11 | YIELD (5R)-3-[2-(3-{[3-(2,6-Dichlorophenyl)propoxy]methyl}phenyl)ethyl]-5-(2,2-dimethyl-4H-1,3-benzodioxin-6-yl)-1,3-oxazolidin-2-one |  |

---

```
Reaction no 276
```

Generated by the Chemistry Development Kit (http://github.com/cdk)

|  | A | B |
| --- | --- | --- |
| 0 | ADD (2-{[2-(aminomethyl)-6-chloro-4-(2-methoxypyridin-4-yl)phenyl]sulfanyl}pyridin-3-yl)methanol HCl salt | ADD (2-{[2-(aminomethyl)-6-chloro-4-(2-methoxypyridin-4-yl)phenyl]sulfanyl}pyridin-3-yl)methanol HCl salt |
| 1 | ADD NaHCO3 | ADD NaHCO3 |
| 2 | MAKESOLUTION with Fmoc-OSu and acetonitrile | MAKESOLUTION with Fmoc-OSu and acetonitrile |
| 3 | ADD SLN at 25 °C | ADD SLN at 25 °C |
| 4 | STIR for 3600 s at 25 °C | STIR for 3600 s at 25 °C |
| 5 | ADD water | ADD water |
| 6 | EXTRACT with ethyl acetate | EXTRACT with ethyl acetate |
| 7 | COLLECTLAYER organic | COLLECTLAYER organic |
| 8 | WASH with brine | WASH with brine |
| 9 | DRYSOLUTION over sodium sulfate | DRYSOLUTION over sodium sulfate |
| 10 | CONCENTRATE | CONCENTRATE |
| 11 | YIELD 9H-fluoren-9-ylmethyl N-[(3-chloro-2-{[3-(hydroxymethyl)pyridin-2-yl]sulfanyl}-5-(2-methoxypyridin-4-yl)phenyl)methyl]carbamate | YIELD 9H-fluoren-9-ylmethyl N-[(3-chloro-2-{[3-(hydroxymethyl)pyridin-2-yl]sulfanyl}-5-(2-methoxypyridin-4-yl)phenyl)methyl]carbamate |

---

```
Reaction no 277
```

Generated by the Chemistry Development Kit (http://github.com/cdk)

|  | A | B |
| --- | --- | --- |
| 0 | ADD 2-(3-Carboxy-propyl)benzoic acid | ADD 2-(3-Carboxy-propyl)benzoic acid |
| 1 | ADD DIPEA | ADD DIPEA |
| 2 | ADD HATU | ADD HATU |
| 3 | ADD dichloromethane | ADD dichloromethane |
| 4 | STIR for 600 s at 25 °C | ADD (R)-3-Amino-4-(2-chloro-phenyl)-butyric Acid Ethyl Ester |
| 5 | ADD (R)-3-Amino-4-(2-chloro-phenyl)-butyric Acid Ethyl Ester | STIR for 3600 s |
| 6 | STIR for 3600 s | ADD ammonium chloride |
| 7 | QUENCH with ammonium chloride | EXTRACT with dichloromethane |
| 8 | EXTRACT with dichloromethane | DRYSOLUTION |
| 9 | DRYSOLUTION | CONCENTRATE |
| 10 | CONCENTRATE | PURIFY |
| 11 | ADD THF | YIELD 2-{3-[(R)-1-Carboxymethyl-2-(2-chlorophenyl)-ethylcarbamoyl]-propyl}-benzoic Acid |
| 12 | ADD NaOH |  |
| 13 | STIR for 3600 s |  |
| 14 | ADD acetic acid |  |
| 15 | PURIFY |  |
| 16 | YIELD 2-{3-[(R)-1-Carboxymethyl-2-(2-chlorophenyl)-ethylcarbamoyl]-propyl}-benzoic Acid |  |

---

```
Reaction no 278
```

Generated by the Chemistry Development Kit (http://github.com/cdk)

|  | A | B |
| --- | --- | --- |
| 0 | ADD 5-(4-(4-aminophenyl)-1H-pyrazol-1-yl)-1-propylpyridin-2(1H)-one | ADD 5-(4-(4-aminophenyl)-1H-pyrazol-1-yl)-1-propylpyridin-2(1H)-one |
| 1 | ADD dichloromethane | ADD dichloromethane |
| 2 | ADD pyridine | ADD pyridine at 0 °C |
| 3 | ADD methanesulfonyl chloride | ADD methanesulfonyl chloride at 0 °C |
| 4 | STIR for 86400 s at 25 °C | STIR for 3600 s at 0 °C |
| 5 | CONCENTRATE | QUENCH with NaHCO3 |
| 6 | PURIFY | EXTRACT with dichloromethane |
| 7 | YIELD N-(4-(1-(6-oxo-1-propyl-1,6-dihydropyridin-3-yl)-1H-pyrazol-4-yl)phenyl)methanesulfonamide | COLLECTLAYER organic |
| 8 |  | WASH with water |
| 9 |  | WASH with brine |
| 10 |  | DRYSOLUTION over MgSO4 |
| 11 |  | CONCENTRATE |
| 12 |  | PURIFY |
| 13 |  | YIELD N-(4-(1-(6-oxo-1-propyl-1,6-dihydropyridin-3-yl)-1H-pyrazol-4-yl)phenyl)methanesulfonamide |

---

```
Reaction no 279
```

Generated by the Chemistry Development Kit (http://github.com/cdk)

|  | A | B |
| --- | --- | --- |
| 0 | ADD diethyl [(6S\*,9aR\*)-6-(2-fluorophenyl)-4-oxooctahydroquinolizin-3-yl]phosphonate | ADD diethyl [(6S\*,9aR\*)-6-(2-fluorophenyl)-4-oxooctahydroquinolizin-3-yl]phosphonate |
| 1 | ADD 3-methoxy-4-(4-methyl-1H-imidazol-1-yl)benzaldehyde | ADD 3-methoxy-4-(4-methyl-1H-imidazol-1-yl)benzaldehyde |
| 2 | ADD THF | ADD THF |
| 3 | ADD ethanol | ADD ethanol |
| 4 | ADD lithium hydroxide monohydrate at 25 °C | ADD lithium hydroxide monohydrate at 25 °C |
| 5 | STIR for 3600 s at 25 °C | STIR for 3600 s at 25 °C |
| 6 | ADD ethyl acetate | ADD ethyl acetate |
| 7 | WASH with NaHCO3 aq. | WASH with NaHCO3 aq. |
| 8 | WASH with brine | WASH with brine |
| 9 | COLLECTLAYER organic | COLLECTLAYER organic |
| 10 | DRYSOLUTION over magnesium sulfate | DRYSOLUTION over sodium sulfate |
| 11 | CONCENTRATE | CONCENTRATE |
| 12 | PURIFY | PURIFY |
| 13 | YIELD 6-(2-fluorophenyl)-3-[3-methoxy-4-(4-methyl-1H-imidazol-1-yl)benzylidene]octahydroquinolizin-4-one | YIELD 6-(2-fluorophenyl)-3-[3-methoxy-4-(4-methyl-1H-imidazol-1-yl)benzylidene]octahydroquinolizin-4-one |

---

```
Reaction no 280
```

Generated by the Chemistry Development Kit (http://github.com/cdk)

|  | A | B |
| --- | --- | --- |
| 0 | ADD 7-azaindole-3-carboxaldehyde | ADD 7-azaindole-3-carboxaldehyde |
| 1 | ADD dichloromethane | ADD dichloromethane |
| 2 | ADD tetrabutylammonium hydrogen sulfate | ADD tetrabutylammonium hydrogen sulfate |
| 3 | ADD benzenesulfonyl chloride | ADD benzenesulfonyl chloride |
| 4 | ADD NaOH | ADD NaOH |
| 5 | STIR for 3600 s at 25 °C | STIR for 86400 s at 25 °C |
| 6 | ADD water | ADD water |
| 7 | EXTRACT with dichloromethane | EXTRACT with dichloromethane |
| 8 | COLLECTLAYER organic | COLLECTLAYER organic |
| 9 | WASH with brine | DRYSOLUTION over Na2SO4 |
| 10 | DRYSOLUTION over Na2SO4 | FILTER keep filtrate |
| 11 | FILTER keep filtrate | CONCENTRATE |
| 12 | CONCENTRATE | PURIFY |
| 13 | YIELD 1-(phenylsulfonyl)-1H-pyrrolo[2,3-b]pyridine-3-carbaldehyde | YIELD 1-(phenylsulfonyl)-1H-pyrrolo[2,3-b]pyridine-3-carbaldehyde |

---

```
Reaction no 281
```

Generated by the Chemistry Development Kit (http://github.com/cdk)

|  | A | B |
| --- | --- | --- |
| 0 | ADD 3-bromo-2,6-difluoro-5-nitrotoluene | ADD iron |
| 1 | ADD ethanol | ADD water |
| 2 | ADD water | ADD HCl at 60 °C |
| 3 | ADD iron | ADD ethanol |
| 4 | ADD HCl | ADD 3-bromo-2,6-difluoro-5-nitrotoluene dropwise at 100 °C over 3600 s |
| 5 | REFLUX for 3600 s | REFLUX for 3600 s |
| 6 | SETTEMPERATURE 25 °C | FILTER keep filtrate |
| 7 | FILTER keep filtrate | WASH with ethanol |
| 8 | CONCENTRATE | ADD ice water |
| 9 | PURIFY | RECRYSTALLIZE from hexane |
| 10 | YIELD 5-Bromo-2,4-difluoro-3-methylaniline | YIELD 5-Bromo-2,4-difluoro-3-methylaniline |

---

```
Reaction no 282
```

Generated by the Chemistry Development Kit (http://github.com/cdk)

|  | A | B |
| --- | --- | --- |
| 0 | ADD methyl 2-(4-fluoro-3-nitrophenyl)acetate | ADD methyl 2-(4-fluoro-3-nitrophenyl)acetate |
| 1 | ADD glycine methyl ester hydrochloride | ADD DMF |
| 2 | ADD DIPEA | ADD glycine methyl ester hydrochloride |
| 3 | ADD DMF | ADD DIPEA |
| 4 | STIR for 86400 s at 25 °C | STIR for 86400 s at 25 °C |
| 5 | ADD brine | ADD brine |
| 6 | EXTRACT with ethyl acetate | EXTRACT with ethyl acetate |
| 7 | COLLECTLAYER organic | COLLECTLAYER organic |
| 8 | WASH with brine | WASH with brine |
| 9 | DRYSOLUTION over sodium sulfate | DRYSOLUTION over Na2SO4 |
| 10 | CONCENTRATE | FILTER keep filtrate |
| 11 | YIELD methyl 2-(4-(2-methoxy-2-oxoethyl)-2-nitrophenylamino)acetate | CONCENTRATE |
| 12 |  | PURIFY |
| 13 |  | YIELD methyl 2-(4-(2-methoxy-2-oxoethyl)-2-nitrophenylamino)acetate |

---

```
Reaction no 283
```

Generated by the Chemistry Development Kit (http://github.com/cdk)

|  | A | B |
| --- | --- | --- |
| 0 | ADD 1-{4-[2-(2,1,3-benzoxadiazol-5-yl)-2-fluoroethyl]piperazin-1-yl}-2-[4-(1H-tetrazol-1-yl)phenyl]ethanone 1-{4-[2-(2,1,3-benzoxadiazol-5-yl)-2-hydroxyethyl]piperazin-1-yl}-2-[4-(1H-tetrazol-1-yl)phenyl]ethanone | ADD 1-{4-[2-(2,1,3-benzoxadiazol-5-yl)-2-fluoroethyl]piperazin-1-yl}-2-[4-(1H-tetrazol-1-yl)phenyl]ethanone 1-{4-[2-(2,1,3-benzoxadiazol-5-yl)-2-hydroxyethyl]piperazin-1-yl}-2-[4-(1H-tetrazol-1-yl)phenyl]ethanone |
| 1 | ADD dichloromethane | ADD dichloromethane |
| 2 | ADD DAST | ADD 1-{4-[2-(2,1,3-benzoxadiazol-5-yl)-2-fluoroethyl]piperazin-1-yl}-2-[4-(1H-tetrazol-1-yl)phenyl]ethanone |
| 3 | STIR for 3600 s at 25 °C | ADD DAST |
| 4 | QUENCH with NaHCO3 | STIR for 3600 s at 0 °C |
| 5 | EXTRACT with dichloromethane | STIR for 3600 s at 25 °C |
| 6 | COLLECTLAYER organic | QUENCH with methanol |
| 7 | DRYSOLUTION over MgSO4 | CONCENTRATE |
| 8 | FILTER keep filtrate | PARTITION with ethyl acetate and water |
| 9 | CONCENTRATE | COLLECTLAYER organic |
| 10 | PURIFY | DRYSOLUTION over MgSO4 |
| 11 | YIELD 1-{4-[2-(2,1,3-benzoxadiazol-5-yl)-2-fluoroethyl]piperazin-1-yl}-2-[4-(1H-tetrazol-1-yl)phenyl]ethanone | PURIFY |

---

```
Reaction no 284
```

Generated by the Chemistry Development Kit (http://github.com/cdk)

|  | A | B |
| --- | --- | --- |
| 0 | ADD 5-amino-3-tert-butylisoxazole | ADD 5-amino-3-tert-butylisoxazole |
| 1 | ADD pyridine | ADD pyridine |
| 2 | ADD ethyl acetate | ADD ethyl acetate |
| 3 | ADD ethyl 2-chloro-2-oxoacetate at 0 °C | SETTEMPERATURE 0 °C |
| 4 | STIR for 3600 s at 25 °C | ADD ethyl 2-chloro-2-oxoacetate dropwise at 25 °C over 3600 s |
| 5 | WASH with water | SETTEMPERATURE 25 °C |
| 6 | WASH with HCl | STIR for 3600 s |
| 7 | WASH with water | WASH with water |
| 8 | WASH with brine | WASH with HCl |
| 9 | DRYSOLUTION over Na2SO4 | WASH with brine |
| 10 | CONCENTRATE | DRYSOLUTION over Na2SO4 |
| 11 | YIELD Ethyl 2-((3-(tert-butyl)isoxazol-5-yl)amino)-2-oxoacetate | CONCENTRATE |
| 12 |  | TRITURATE with isohexane |
| 13 |  | YIELD Ethyl 2-((3-(tert-butyl)isoxazol-5-yl)amino)-2-oxoacetate |

---

```
Reaction no 285
```

Generated by the Chemistry Development Kit (http://github.com/cdk)

|  | A | B |
| --- | --- | --- |
| 0 | ADD 4-(4-piperidin-4-ylbutyl)pyridine | ADD 4-(4-piperidin-4-ylbutyl)pyridine |
| 1 | ADD 2-fluoropyridine | ADD 2-fluoropyridine |
| 2 | ADD DBU | ADD DBU |
| 3 | STIR for 28800 s at 100 °C | STIR for 86400 s at 60 °C |
| 4 | CONCENTRATE | CONCENTRATE |
| 5 | PURIFY | PURIFY |
| 6 | YIELD 4-(4-Pyridin-4-ylbutyl)-3,4,5,6-tetrahydro-2H-[1,2′]bipyridinyl | YIELD 4-(4-Pyridin-4-ylbutyl)-3,4,5,6-tetrahydro-2H-[1,2′]bipyridinyl |

---

```
Reaction no 286
```

Generated by the Chemistry Development Kit (http://github.com/cdk)

|  | A | B |
| --- | --- | --- |
| 0 | ADD 4-oxo-5-(4-phenoxyphenyl)-N-(piperidin-4-yl)-4,5-dihydro-3H-1-thia-3,5,8-triazaacenaphthylene-2-carboxamide | ADD 4-oxo-5-(4-phenoxyphenyl)-N-(piperidin-4-yl)-4,5-dihydro-3H-1-thia-3,5,8-triazaacenaphthylene-2-carboxamide |
| 1 | ADD 4-hydroxy-but-2-enoic acid | ADD 4-hydroxy-but-2-enoic acid |
| 2 | ADD HATU | ADD DMF |
| 3 | ADD triethylamine | ADD HATU |
| 4 | ADD DMF | MICROWAVE for 600 s at 100 °C |
| 5 | STIR for 86400 s at 25 °C | ADD dichloromethane |
| 6 | ADD dichloromethane | WASH with water |
| 7 | WASH with water | PURIFY |
| 8 | WASH with brine | YIELD (E)-N-(1-(4-Hydroxybut-2-enoyl)piperidin-4-yl)-4-oxo-5-(4-phenoxyphenyl)-4,5-dihydro-3H-1-thia-3,5,8-triazaacenaphthylene-2-carboxamide |
| 9 | DRYSOLUTION over Na2SO4 |  |
| 10 | FILTER keep filtrate |  |
| 11 | CONCENTRATE |  |
| 12 | PURIFY |  |
| 13 | YIELD (E)-N-(1-(4-Hydroxybut-2-enoyl)piperidin-4-yl)-4-oxo-5-(4-phenoxyphenyl)-4,5-dihydro-3H-1-thia-3,5,8-triazaacenaphthylene-2-carboxamide |  |

---

```
Reaction no 287
```

Generated by the Chemistry Development Kit (http://github.com/cdk)

|  | A | B |
| --- | --- | --- |
| 0 | ADD 4-cyano-1H-imidazole-2-carboxylic acid [2-(4,4-dimethyl-cyclohex-1-enyl)-4-piperidin-4-yl-phenyl]-amide | ADD N,N-dimethylglycine |
| 1 | ADD N,N-dimethylglycine | ADD dichloromethane |
| 2 | ADD BOP-Cl | ADD BOP-Cl |
| 3 | ADD triethylamine | ADD triethylamine |
| 4 | ADD dichloromethane | STIR for 3600 s at 25 °C |
| 5 | STIR for 86400 s at 25 °C | MAKESOLUTION with 4-cyano-1H-imidazole-2-carboxylic acid [2-(4,4-dimethyl-cyclohex-1-enyl)-4-piperidin-4-yl-phenyl]-amide and dichloromethane |
| 6 | PURIFY | ADD SLN |
| 7 | YIELD 4-Cyano-1H-imidazole-2-carboxylic acid [4-[1-(2-dimethylamino-acetyl)-piperidin-4-yl]-2-(4,4-dimethyl-cyclohex-1-enyl)-phenyl]-amide trifluoroacetic acid salt | STIR for 86400 s at 25 °C |
| 8 |  | ADD dichloromethane |
| 9 |  | WASH with NaHCO3 |
| 10 |  | COLLECTLAYER organic |
| 11 |  | DRYSOLUTION over MgSO4 |
| 12 |  | CONCENTRATE |
| 13 |  | PURIFY |
| 14 |  | YIELD 4-Cyano-1H-imidazole-2-carboxylic acid [4-[1-(2-dimethylamino-acetyl)-piperidin-4-yl]-2-(4,4-dimethyl-cyclohex-1-enyl)-phenyl]-amide trifluoroacetic acid salt |

---

```
Reaction no 288
```

Generated by the Chemistry Development Kit (http://github.com/cdk)

|  | A | B |
| --- | --- | --- |
| 0 | ADD 4-(4-chloro-7H-pyrrolo[2,3-d]pyrimidin-6-yl)-3,6-dihydro-2H-pyridine-1-carboxylic acid tert-butyl ester | ADD 4-(4-chloro-7H-pyrrolo[2,3-d]pyrimidin-6-yl)-3,6-dihydro-2H-pyridine-1-carboxylic acid tert-butyl ester |
| 1 | ADD 1-Phenethyl-1H-pyrazol-4-ylamine | ADD 1-Phenethyl-1H-pyrazol-4-ylamine |
| 2 | ADD n-butanol | ADD n-butanol |
| 3 | STIR for 86400 s at 100 °C | STIR for 86400 s at 100 °C |
| 4 | CONCENTRATE | WAIT for 86400 s |
| 5 | PURIFY | SETTEMPERATURE 25 °C |
| 6 | YIELD 4-[4-(1-Phenethyl-1H-pyrazol-4-ylamino)-7H-pyrrolo[2,3-d]pyrimidin-6-yl]-3,6-dihydro-2H-pyridine-1-carboxylic acid tert-butyl ester | ADD dichloromethane |
| 7 |  | ADD DIPEA |
| 8 |  | ADD di-tert-butyl dicarbonate |
| 9 |  | STIR for 3600 s at 25 °C |
| 10 |  | ADD ethyl acetate |
| 11 |  | WASH with brine |
| 12 |  | DRYSOLUTION over sodium sulfate |
| 13 |  | CONCENTRATE |
| 14 |  | RECRYSTALLIZE from ethyl acetate |
| 15 |  | YIELD 4-[4-(1-Phenethyl-1H-pyrazol-4-ylamino)-7H-pyrrolo[2,3-d]pyrimidin-6-yl]-3,6-dihydro-2H-pyridine-1-carboxylic acid tert-butyl ester |

---

```
Reaction no 289
```

Generated by the Chemistry Development Kit (http://github.com/cdk)

|  | A | B |
| --- | --- | --- |
| 0 | ADD N-chlorosuccinimide | ADD dimethyl sulfide |
| 1 | ADD toluene | ADD toluene |
| 2 | ADD dimethyl sulfide at 0 °C | ADD N-chlorosuccinimide at 0 °C |
| 3 | SETTEMPERATURE -30 °C | STIR for 3600 s at 0 °C |
| 4 | ADD 5-chlorobenzothiophene-2-methanol | MAKESOLUTION with 5-chlorobenzothiophene-2-methanol and toluene |
| 5 | STIR for 3600 s | ADD SLN |
| 6 | ADD triethylamine | STIR for 3600 s at 0 °C |
| 7 | SETTEMPERATURE 25 °C | ADD triethylamine |
| 8 | ADD ether | STIR for 3600 s at 25 °C |
| 9 | WASH with HCl | ADD ether |
| 10 | WASH with water | WASH with water |
| 11 | WASH with brine | WASH with sodium chloride |
| 12 | DRYSOLUTION over sodium sulfate | DRYSOLUTION over sodium sulfate |
| 13 | CONCENTRATE | CONCENTRATE |
| 14 | YIELD 5-chlorobenzo[b]thiophene-2-carboxaldehyde | PURIFY |
| 15 |  | YIELD 5-chlorobenzo[b]thiophene-2-carboxaldehyde |

---

```
Reaction no 290
```

Generated by the Chemistry Development Kit (http://github.com/cdk)

|  | A | B |
| --- | --- | --- |
| 0 | ADD 7-ADCA | ADD 7-ADCA |
| 1 | ADD dichloromethane | ADD dichloromethane |
| 2 | ADD triethylamine at 0 °C | ADD NMP |
| 3 | WAIT for 3600 s | ADD dichlorodimethylsilane at 0 °C |
| 4 | ADD dichlorodimethylsilane dropwise | STIR for 3600 s at 0 °C |
| 5 | STIR for 3600 s at 25 °C | ADD triethylamine |
| 6 | SETTEMPERATURE 0 °C | ADD D(-)-p-hydroxyphenylglycyl chloride hydrochloride |
| 7 | ADD NMP | STIR for 3600 s at 0 °C |
| 8 | ADD D(-)-p-hydroxyphenylglycyl chloride hydrochloride | ADD water |
| 9 | STIR for 3600 s at 0 °C | STIR for 3600 s at 0 °C |
| 10 | ADD water | FILTER keep precipitate |
| 11 | PHASESEPARATION | WASH with water |
| 12 | COLLECTLAYER aqueous | WASH with acetone |
| 13 | PH with ammonia to pH acidic | WASH with ether |
| 14 | STIR for 86400 s | YIELD cephadroxyl |
| 15 | FILTER keep precipitate |  |
| 16 | YIELD cephadroxyl |  |

---

```
Reaction no 291
```

Generated by the Chemistry Development Kit (http://github.com/cdk)

|  | A | B |
| --- | --- | --- |
| 0 | ADD (2S,3R,4S,5S)-ethyl 4-((5-bromo-2-methoxypyridin-3-yl)methoxy)-3-(tert-butyl)-5-(2-cyclopropylphenyl)pyrrolidine-2-carboxylate | ADD (2S,3R,4S,5S)-ethyl 4-((5-bromo-2-methoxypyridin-3-yl)methoxy)-3-(tert-butyl)-5-(2-cyclopropylphenyl)pyrrolidine-2-carboxylate |
| 1 | ADD dichloromethane | ADD dichloromethane |
| 2 | ADD (S)-tetrahydrofuran-2-carbonyl chloride | ADD pyridine |
| 3 | ADD pyridine | ADD (S)-tetrahydrofuran-2-carbonyl chloride |
| 4 | STIR for 3600 s at 25 °C | STIR for 3600 s at 25 °C |
| 5 | ADD ethyl acetate | ADD ethyl acetate |
| 6 | WASH with water | WASH with NaHCO3 |
| 7 | WASH with brine | WASH with brine |
| 8 | COLLECTLAYER organic | COLLECTLAYER organic |
| 9 | DRYSOLUTION over Na2SO4 | DRYSOLUTION over MgSO4 |
| 10 | FILTER keep filtrate | FILTER keep filtrate |
| 11 | CONCENTRATE | CONCENTRATE |
| 12 | YIELD (2S,3R,4S,5S)-ethyl 4-((5-bromo-2-methoxypyridin-3-yl)methoxy)-3-(tert-butyl)-5-(2-cyclopropylphenyl)-1-((S)-tetrahydrofuran-2-carbonyl)pyrrolidine-2-carboxylate | PURIFY |
| 13 |  | YIELD (2S,3R,4S,5S)-ethyl 4-((5-bromo-2-methoxypyridin-3-yl)methoxy)-3-(tert-butyl)-5-(2-cyclopropylphenyl)-1-((S)-tetrahydrofuran-2-carbonyl)pyrrolidine-2-carboxylate |

---

```
Reaction no 292
```

Generated by the Chemistry Development Kit (http://github.com/cdk)

|  | A | B |
| --- | --- | --- |
| 0 | ADD 3-(3-cyano-2,4,6-tribromophenyl)propionic acid | ADD 3-(3-cyano-2,4,6-tribromophenyl)propionic acid |
| 1 | ADD sulfuric acid | ADD sulfuric acid |
| 2 | STIR for 3600 s at 100 °C | STIR for 3600 s at 60 °C |
| 3 | SETTEMPERATURE 25 °C | STIR for 3600 s at 100 °C |
| 4 | ADD ice water | ADD water |
| 5 | FILTER keep precipitate | STIR for 28800 s at 0 °C |
| 6 | WASH with water | FILTER keep precipitate |
| 7 | DRYSOLID under vacuum | WASH with ice water |
| 8 | YIELD 3-(3-carbamoyl-2,4,6-tribromophenyl)propionic acid | DRYSOLID at 60 °C under vacuum |
| 9 |  | YIELD 3-(3-carbamoyl-2,4,6-tribromophenyl)propionic acid |

---

```
Reaction no 293
```

Generated by the Chemistry Development Kit (http://github.com/cdk)

|  | A | B |
| --- | --- | --- |
| 0 | ADD N-(dibenzo[b,d]furan-4-yl)acetamide | ADD N-(dibenzo[b,d]furan-4-yl)acetamide |
| 1 | ADD HCl | ADD HCl |
| 2 | ADD methanol | ADD methanol |
| 3 | REFLUX for 3600 s | REFLUX for 86400 s |
| 4 | SETTEMPERATURE 25 °C | SETTEMPERATURE 25 °C |
| 5 | PH with Na2CO3 to pH neutral | PH with Na2CO3 to pH basic |
| 6 | FILTER keep precipitate | EXTRACT with dichloromethane |
| 7 | ADD dichloromethane | COLLECTLAYER organic |
| 8 | DRYSOLUTION over sodium sulfate | WASH with water |
| 9 | CONCENTRATE | DRYSOLUTION over Na2SO4 |
| 10 | YIELD dibenzo[b,d]furan-4-amine | CONCENTRATE |
| 11 |  | YIELD dibenzo[b,d]furan-4-amine |

---

```
Reaction no 294
```

Generated by the Chemistry Development Kit (http://github.com/cdk)

|  | A | B |
| --- | --- | --- |
| 0 | ADD 7-Methoxy-3-(4-methoxy-phenyl)-chroman-4-one | ADD 7-Methoxy-3-(4-methoxy-phenyl)-chroman-4-one |
| 1 | ADD hydroxylamine hydrochloride | ADD hydroxylamine hydrochloride |
| 2 | ADD pyridine | ADD pyridine |
| 3 | ADD ethanol | ADD ethanol |
| 4 | REFLUX for 3600 s | REFLUX for 3600 s |
| 5 | SETTEMPERATURE 25 °C | ADD water |
| 6 | CONCENTRATE | FILTER keep precipitate |
| 7 | PARTITION with ethyl acetate and water | YIELD 7-Methoxy-3-(3-methoxy-phenyl)-chroman-4-one oxime |
| 8 | PHASESEPARATION |  |
| 9 | COLLECTLAYER organic |  |
| 10 | DRYSOLUTION over MgSO4 |  |
| 11 | CONCENTRATE |  |
| 12 | YIELD 7-Methoxy-3-(3-methoxy-phenyl)-chroman-4-one oxime |  |

---

```
Reaction no 295
```

Generated by the Chemistry Development Kit (http://github.com/cdk)

|  | A | B |
| --- | --- | --- |
| 0 | ADD 6-cyano-7-fluorochroman-4-carboxylic acid | ADD 6-cyano-7-fluorochroman-4-carboxylic acid |
| 1 | ADD THF | ADD THF |
| 2 | ADD (Z)-tert-butyl N,N′-diisopropylcarbamimidate | ADD (Z)-tert-butyl N,N′-diisopropylcarbamimidate |
| 3 | STIR for 28800 s | REFLUX for 86400 s |
| 4 | ADD (Z)-tert-butyl N,N′-diisopropylcarbamimidate | SETTEMPERATURE 25 °C |
| 5 | STIR for 86400 s | ADD ether |
| 6 | ADD ether | FILTER keep filtrate |
| 7 | FILTER keep filtrate | CONCENTRATE |
| 8 | CONCENTRATE | PURIFY |
| 9 | PURIFY | YIELD tert-butyl 6-cyano-7-fluorochroman-4-carboxylate |
| 10 | YIELD tert-butyl 6-cyano-7-fluorochroman-4-carboxylate |  |

---

```
Reaction no 296
```

Generated by the Chemistry Development Kit (http://github.com/cdk)

|  | A | B |
| --- | --- | --- |
| 0 | ADD 2-((3-bromobenzyl)(methyl)amino)-1-(naphthalen-2-yl)ethanone at 0 °C | ADD 2-((3-bromobenzyl)(methyl)amino)-1-(naphthalen-2-yl)ethanone |
| 1 | ADD methanol at 0 °C | ADD methanol |
| 2 | ADD sodium borohydride at 0 °C | ADD sodium borohydride at 0 °C |
| 3 | STIR for 86400 s at 25 °C | STIR for 3600 s at 25 °C |
| 4 | CONCENTRATE | CONCENTRATE |
| 5 | ADD water | ADD water |
| 6 | EXTRACT with dichloromethane | EXTRACT with ethyl acetate |
| 7 | COLLECTLAYER organic | COLLECTLAYER organic |
| 8 | DRYSOLUTION over sodium sulfate | WASH with brine |
| 9 | FILTER keep filtrate | DRYSOLUTION over Na2SO4 |
| 10 | CONCENTRATE | CONCENTRATE |
| 11 | YIELD 2-((3-bromobenzyl)(methyl)amino)-1-(naphthalen-2-yl)ethanol | YIELD 2-((3-bromobenzyl)(methyl)amino)-1-(naphthalen-2-yl)ethanol |

---

```
Reaction no 297
```

Generated by the Chemistry Development Kit (http://github.com/cdk)

|  | A | B |
| --- | --- | --- |
| 0 | ADD thiophene-2,5-diyldimethanol | ADD thiophene-2,5-diyldimethanol |
| 1 | ADD chloroform | ADD chloroform |
| 2 | ADD DMF | ADD thionyl chloride |
| 3 | ADD thionyl chloride | ADD DMF |
| 4 | STIR for 86400 s at 25 °C | REFLUX for 3600 s |
| 5 | CONCENTRATE | CONCENTRATE |
| 6 | PURIFY | YIELD 2,5-bis-chloromethyl-thiophene |
| 7 | YIELD 2,5-bis-chloromethyl-thiophene |  |

---

```
Reaction no 298
```

Generated by the Chemistry Development Kit (http://github.com/cdk)

|  | A | B |
| --- | --- | --- |
| 0 | ADD indole | ADD NaH |
| 1 | ADD THF | ADD THF |
| 2 | ADD NaH at 0 °C | MAKESOLUTION with indole and THF |
| 3 | STIR for 3600 s at 0 °C | ADD SLN at 25 °C over 600 s |
| 4 | ADD 2-bromo-5-(chlorosulfonyl)benzoic acid | ADD THF |
| 5 | STIR for 86400 s at 25 °C | STIR for 3600 s at 25 °C |
| 6 | QUENCH with water | MAKESOLUTION with 2-bromo-5-(chlorosulfonyl)benzoic acid and THF |
| 7 | PH with HCl to pH acidic | ADD SLN dropwise over 600 s |
| 8 | EXTRACT with ethyl acetate | QUENCH with water |
| 9 | COLLECTLAYER organic | PH with HCl to pH acidic |
| 10 | DRYSOLUTION over Na2SO4 | EXTRACT with ethyl acetate |
| 11 | CONCENTRATE | COLLECTLAYER organic |
| 12 | PURIFY | WASH with brine |
| 13 | YIELD 2-Bromo-5-(indole-1-sulfonyl) benzoic acid | WASH with water |
| 14 |  | DRYSOLUTION over Na2SO4 |
| 15 |  | CONCENTRATE |
| 16 |  | YIELD 2-Bromo-5-(indole-1-sulfonyl) benzoic acid |

---

```
Reaction no 299
```

Generated by the Chemistry Development Kit (http://github.com/cdk)

|  | A | B |
| --- | --- | --- |
| 0 | ADD 5-(trifluoromethyl)nicotinic acid | ADD 5-(trifluoromethyl)nicotinic acid |
| 1 | ADD 3-aminopyridine | ADD THF |
| 2 | ADD 1-hydroxybenzotriazole hydrate | ADD TBTU |
| 3 | ADD triethylamine | ADD 1-hydroxybenzotriazole hydrate |
| 4 | ADD THF | ADD triethylamine |
| 5 | ADD TBTU at 0 °C | STIR for 600 s at 25 °C |
| 6 | STIR for 600 s at 25 °C | ADD 3-aminopyridine |
| 7 | STIR for 86400 s at 25 °C | STIR for 86400 s at 25 °C |
| 8 | CONCENTRATE | CONCENTRATE |
| 9 | ADD dichloromethane | PURIFY |
| 10 | WASH with NaHCO3 | YIELD N-Pyridin-3-yl-5-trifluoromethylnicotinamide |
| 11 | DRYSOLUTION over Na2SO4 |  |
| 12 | PURIFY |  |
| 13 | YIELD N-Pyridin-3-yl-5-trifluoromethylnicotinamide |  |

---

```
Reaction no 300
```

Generated by the Chemistry Development Kit (http://github.com/cdk)

|  | A | B |
| --- | --- | --- |
| 0 | ADD tert-butyl 3-(3-(4-(3-(2-chloroethyl)ureido)butyl)-2-oxoimidazolidin-1-yl)-3-(3-(trifluoromethyl)phenyl)propanoate | ADD tert-butyl 3-(3-(4-(3-(2-chloroethyl)ureido)butyl)-2-oxoimidazolidin-1-yl)-3-(3-(trifluoromethyl)phenyl)propanoate |
| 1 | ADD water at 25 °C | ADD ethyl acetate |
| 2 | STIR for 3600 s at 100 °C | MAKESOLUTION with K2CO3 and water |
| 3 | SETTEMPERATURE 25 °C | ADD SLN |
| 4 | ADD HCl | STIR for 86400 s at 25 °C |
| 5 | STIR for 600 s at 25 °C | PH with HCl to pH acidic |
| 6 | ADD ethyl acetate | CONCENTRATE |
| 7 | ADD K2CO3 | PURIFY |
| 8 | PHASESEPARATION | YIELD 3-(3-(4-((4,5-dihydrooxazol-2-yl)amino)butyl)-2-oxoimidazolidin-1-yl)-3-(3-(trifluoromethyl) phenyl)propanoic acid |
| 9 | COLLECTLAYER aqueous |  |
| 10 | EXTRACT with ethyl acetate |  |
| 11 | COLLECTLAYER organic |  |
| 12 | WASH with brine |  |
| 13 | DRYSOLUTION over MgSO4 |  |
| 14 | FILTER keep filtrate |  |
| 15 | CONCENTRATE |  |
| 16 | PURIFY |  |
| 17 | YIELD 3-(3-(4-((4,5-dihydrooxazol-2-yl)amino)butyl)-2-oxoimidazolidin-1-yl)-3-(3-(trifluoromethyl) phenyl)propanoic acid |  |

---

```
Reaction no 301
```

Generated by the Chemistry Development Kit (http://github.com/cdk)

|  | A | B |
| --- | --- | --- |
| 0 | MAKESOLUTION with 1-fluoro-4-nitrobenzene and 2-hydroxypyridine and DMF | ADD 2-hydroxypyridine |
| 1 | ADD Cs2CO3 | ADD 1-fluoro-4-nitrobenzene |
| 2 | ADD SLN | ADD Cs2CO3 |
| 3 | STIR for 86400 s at 100 °C | ADD DMF |
| 4 | SETTEMPERATURE 25 °C | STIR for 86400 s at 100 °C |
| 5 | ADD water | SETTEMPERATURE 25 °C |
| 6 | FILTER keep filtrate | ADD water |
| 7 | DRYSOLUTION | FILTER keep precipitate |
| 8 | RECRYSTALLIZE from ethyl acetate | WASH with water |
| 9 | YIELD 1-(4-nitro-phenyl)-1H-pyridin-2-one | DRYSOLID under vacuum |
| 10 |  | YIELD 1-(4-nitro-phenyl)-1H-pyridin-2-one |

---

```
Reaction no 302
```

Generated by the Chemistry Development Kit (http://github.com/cdk)

|  | A | B |
| --- | --- | --- |
| 0 | ADD [1-(3-methanesulfonyloxypropyl)-3-(2-piperidinoethyl) imidazolidin-2-ylidene]malononitrile | ADD [1-(3-methanesulfonyloxypropyl)-3-(2-piperidinoethyl) imidazolidin-2-ylidene]malononitrile |
| 1 | ADD dioxane | ADD dioxane |
| 2 | ADD diethylamine | ADD diethylamine |
| 3 | ADD Natriumiodid | ADD Natriumiodid |
| 4 | STIR for 86400 s at 100 °C | STIR for 86400 s at 100 °C |
| 5 | ADD water | ADD diethylamine |
| 6 | EXTRACT with ethyl acetate | STIR for 28800 s |
| 7 | COLLECTLAYER organic | ADD water |
| 8 | WASH with water | EXTRACT with ethyl acetate |
| 9 | WASH with brine | COLLECTLAYER organic |
| 10 | DRYSOLUTION over magnesium sulfate | WASH with brine |
| 11 | CONCENTRATE | DRYSOLUTION over magnesium sulfate |
| 12 | PURIFY | CONCENTRATE |
| 13 | YIELD [1-(3-Diethylaminopropyl)-3-(2-piperidinoethyl) imidazolidin-2-ylidene]malononitrile | PURIFY |
| 14 |  | WASH with hexane |
| 15 |  | YIELD [1-(3-Diethylaminopropyl)-3-(2-piperidinoethyl) imidazolidin-2-ylidene]malononitrile |

---

```
Reaction no 303
```

Generated by the Chemistry Development Kit (http://github.com/cdk)

|  | A | B |
| --- | --- | --- |
| 0 | ADD sodium | ADD sodium |
| 1 | ADD ethanol | ADD ethanol |
| 2 | ADD 2,3-dichlorobenzenethiol | ADD 2,3-dichlorobenzenethiol |
| 3 | ADD Ethyl 5-chloro-4-nitrothiophene-2-carboxylate | ADD Ethyl 5-chloro-4-nitrothiophene-2-carboxylate |
| 4 | REFLUX for 3600 s | STIR for 3600 s at 25 °C |
| 5 | SETTEMPERATURE 25 °C | WAIT for 86400 s |
| 6 | ADD water | ADD water |
| 7 | YIELD Ethyl 5-(2,3-dichlorophenylsulfanyl)-4-nitrothiophene-2-carboxylate | FILTER keep precipitate |
| 8 |  | DRYSOLID for 3600 s at 60 °C under vacuum |
| 9 |  | ADD ethanol |
| 10 |  | STIR for 3600 s |
| 11 |  | FILTER keep precipitate |
| 12 |  | DRYSOLID for 3600 s at 60 °C under vacuum |
| 13 |  | YIELD Ethyl 5-(2,3-dichlorophenylsulfanyl)-4-nitrothiophene-2-carboxylate |

---

```
Reaction no 304
```

Generated by the Chemistry Development Kit (http://github.com/cdk)

|  | A | B |
| --- | --- | --- |
| 0 | ADD 6-Bromo-3-methanesulfonyl-2-methyl-4-morpholin-4-yl-quinoline | ADD 6-Bromo-3-methanesulfonyl-2-methyl-4-morpholin-4-yl-quinoline |
| 1 | ADD trans-2-phenylcyclopropylboronic acid | ADD trans-2-phenylcyclopropylboronic acid |
| 2 | ADD K3PO4 | ADD Pd(dppf)Cl2 |
| 3 | ADD Pd(PPh3)4 | ADD K3PO4 |
| 4 | REFLUX for 28800 s | PURIFY |
| 5 | PURIFY | YIELD 3-Methanesulfonyl-2-methyl-4-morpholin-4-yl-6-((1R,2R)-2-phenyl-cyclopropyl)-quinoline |
| 6 | YIELD 3-Methanesulfonyl-2-methyl-4-morpholin-4-yl-6-((1R,2R)-2-phenyl-cyclopropyl)-quinoline |  |

---

```
Reaction no 305
```

Generated by the Chemistry Development Kit (http://github.com/cdk)

|  | A | B |
| --- | --- | --- |
| 0 | ADD 4-fluoroacetophenone | ADD 4-fluoroacetophenone |
| 1 | ADD chlorotrimethylsilane | ADD triethylamine |
| 2 | ADD triethylamine | ADD DMF |
| 3 | ADD DMF | MAKESOLUTION with chlorotrimethylsilane and pentane |
| 4 | REFLUX for 86400 s | ADD SLN at 25 °C |
| 5 | SETTEMPERATURE 25 °C | STIR for 86400 s at 25 °C |
| 6 | ADD pentane | ADD pentane |
| 7 | WASH with sodium bicarbonate | WASH with water |
| 8 | WASH with HCl | WASH with brine |
| 9 | WASH with sodium bicarbonate | DRYSOLUTION over magnesium sulfate |
| 10 | DRYSOLUTION | CONCENTRATE |
| 11 | CONCENTRATE | YIELD 1-fluoro-4-[1[(trimethylsilyl)oxy]ethenyl]benzene |
| 12 | YIELD 1-fluoro-4-[1[(trimethylsilyl)oxy]ethenyl]benzene |  |

---

```
Reaction no 306
```

Generated by the Chemistry Development Kit (http://github.com/cdk)

|  | A | B |
| --- | --- | --- |
| 0 | ADD 2-(4-Methyl-1-piperazinyl)-7-methylbenzoxazole Phosphorus pentachloride | ADD 2-(4-Methyl-1-piperazinyl)-7-methylbenzoxazole Phosphorus pentachloride |
| 1 | ADD 2-mercapto-7-methylbenzoxazole | ADD toluene |
| 2 | ADD toluene | ADD 2-mercapto-7-methylbenzoxazole |
| 3 | REFLUX for 3600 s | STIR for 3600 s at 100 °C |
| 4 | SETTEMPERATURE 0 °C | SETTEMPERATURE 0 °C |
| 5 | ADD 1-methylpiperazine | ADD 1-methylpiperazine dropwise |
| 6 | STIR for 3600 s at 25 °C | STIR for 600 s |
| 7 | EXTRACT with ethyl acetate | EXTRACT with ethyl acetate |
| 8 | COLLECTLAYER organic | COLLECTLAYER organic |
| 9 | WASH with water | WASH with sodium bicarbonate |
| 10 | WASH with brine | WASH with brine |
| 11 | DRYSOLUTION over magnesium sulfate | DRYSOLUTION over magnesium sulfate |
| 12 | CONCENTRATE | CONCENTRATE |
| 13 | PURIFY | PURIFY |
| 14 | YIELD 2-(4-methyl-1-piperazinyl)-7-methylbenzoxazole | YIELD 2-(4-methyl-1-piperazinyl)-7-methylbenzoxazole |

---

```
Reaction no 307
```

Generated by the Chemistry Development Kit (http://github.com/cdk)

|  | A | B |
| --- | --- | --- |
| 0 | PH with sulfuric acid to pH acidic | ADD ethyl chrysanthemate |
| 1 | EXTRACT with toluene | ADD sulfuric acid |
| 2 | COLLECTLAYER organic | STIR for 86400 s at 25 °C |
| 3 | WASH with ethyl chrysanthemate | EXTRACT with ethyl acetate |
| 4 | CONCENTRATE | COLLECTLAYER organic |
| 5 | CONCENTRATE | WASH with brine |
| 6 | YIELD trans-chrysanthemic acid | DRYSOLUTION over sodium sulfate |
| 7 |  | CONCENTRATE |
| 8 |  | PURIFY |
| 9 |  | YIELD trans-chrysanthemic acid |

---

```
Reaction no 308
```

Generated by the Chemistry Development Kit (http://github.com/cdk)

|  | A | B |
| --- | --- | --- |
| 0 | ADD 4-piperidone monohydrate hydrochloride | ADD 4-piperidone monohydrate hydrochloride |
| 1 | ADD K2CO3 | ADD water |
| 2 | ADD water | ADD K2CO3 |
| 3 | MAKESOLUTION with N-(benzyloxycarbonyloxy)succinimide and acetonitrile | MAKESOLUTION with N-(benzyloxycarbonyloxy)succinimide and acetonitrile |
| 4 | ADD SLN at 0 °C | ADD SLN at 25 °C |
| 5 | STIR for 3600 s at 25 °C | STIR for 86400 s |
| 6 | EXTRACT with ethyl acetate | CONCENTRATE |
| 7 | COLLECTLAYER organic | EXTRACT with ethyl acetate |
| 8 | WASH with brine | WASH with citric acid |
| 9 | DRYSOLUTION over sodium sulfate | WASH with NaHCO3 |
| 10 | CONCENTRATE | WASH with brine |
| 11 | PURIFY | DRYSOLUTION over MgSO4 |
| 12 | YIELD benzyl 4-oxopiperidine-1-carboxylate | FILTER keep filtrate |
| 13 |  | CONCENTRATE |
| 14 |  | YIELD benzyl 4-oxopiperidine-1-carboxylate |

---

```
Reaction no 309
```

Generated by the Chemistry Development Kit (http://github.com/cdk)

|  | A | B |
| --- | --- | --- |
| 0 | ADD 3,3,9,9-tetramethyl-4,8-diazaundecane-2,10-dione 2-hydrazone 10-oxime | ADD N-(2-aminoethyl)-1-amino-1,1-dimethyl-2-butanone oxime |
| 1 | ADD N-(2-aminoethyl)-1-amino-1,1-dimethyl-2-butanone oxime | ADD DMF |
| 2 | ADD K2CO3 | ADD 3,3,9,9-tetramethyl-4,8-diazaundecane-2,10-dione 2-hydrazone 10-oxime |
| 3 | ADD DMF | ADD K2CO3 |
| 4 | STIR for 86400 s at 60 °C | STIR for 86400 s at 25 °C |
| 5 | SETTEMPERATURE 25 °C | ADD dichloromethane |
| 6 | ADD dichloromethane | STIR for 600 s |
| 7 | FILTER keep filtrate | FILTER keep filtrate |
| 8 | CONCENTRATE | YIELD 4,7-Diaza-3,3,8,8-tetramethyldecane-2,9-dione |
| 9 | PURIFY |  |
| 10 | YIELD 4,7-Diaza-3,3,8,8-tetramethyldecane-2,9-dione |  |

---

```
Reaction no 310
```

Generated by the Chemistry Development Kit (http://github.com/cdk)

|  | A | B |
| --- | --- | --- |
| 0 | ADD THF | ADD ethyl 8-hydroxy-3-oxo-2-(2,2,2-trifluoroethyl)-2,3,4,5-tetrahydro-1H-2-benzazepin-4-acetate |
| 1 | ADD 2-(imidazo[1,2-a]pyrimidin-2-yl)ethanol | ADD 2-(imidazo[1,2-a]pyrimidin-2-yl)ethanol |
| 2 | ADD ethyl 8-hydroxy-3-oxo-2-(2,2,2-trifluoroethyl)-2,3,4,5-tetrahydro-1H-2-benzazepin-4-acetate | ADD triphenylphosphine |
| 3 | ADD triphenylphosphine | ADD THF |
| 4 | SETTEMPERATURE 0 °C | ADD diisopropyl azodicarboxylate at 0 °C |
| 5 | ADD THF | STIR for 86400 s at 25 °C |
| 6 | ADD diisopropyl azodicarboxylate | CONCENTRATE |
| 7 | STIR for 86400 s | PURIFY |
| 8 | CONCENTRATE | YIELD Ethyl 8-[2-(imidazo[1,2-a]pyrimidin-2-yl)ethoxy]-3-oxo-2-(2,2,2-trifluoroethyl)-2,3,4,5-tetrahydro-1H-2-benzazepin-4-acetate |
| 9 | PURIFY |  |
| 10 | COLLECTLAYER organic |  |
| 11 | CONCENTRATE |  |
| 12 | YIELD Ethyl 8-[2-(imidazo[1,2-a]pyrimidin-2-yl)ethoxy]-3-oxo-2-(2,2,2-trifluoroethyl)-2,3,4,5-tetrahydro-1H-2-benzazepin-4-acetate |  |

---

```
Reaction no 311
```

Generated by the Chemistry Development Kit (http://github.com/cdk)

|  | A | B |
| --- | --- | --- |
| 0 | ADD tert-butyl 6-oxo-1,4-oxazepane-4-carboxylate | ADD tert-butyl 6-oxo-1,4-oxazepane-4-carboxylate |
| 1 | ADD dichloromethane | ADD dichloromethane |
| 2 | ADD N-methylbenzylamine | ADD N-methylbenzylamine |
| 3 | STIR for 3600 s at 25 °C | ADD sodium triacetoxyborohydride |
| 4 | ADD sodium triacetoxyborohydride | STIR for 86400 s at 25 °C |
| 5 | STIR for 86400 s at 25 °C | ADD dichloromethane |
| 6 | QUENCH with water | WASH with brine |
| 7 | EXTRACT with ethyl acetate | COLLECTLAYER organic |
| 8 | COLLECTLAYER organic | DRYSOLUTION over Na2SO4 |
| 9 | DRYSOLUTION over MgSO4 | CONCENTRATE |
| 10 | FILTER keep filtrate | PURIFY |
| 11 | CONCENTRATE | YIELD tert-butyl 6-(benzyl(methyl)amino)-1,4-oxazepane-4-carboxylate |
| 12 | PURIFY |  |
| 13 | YIELD tert-butyl 6-(benzyl(methyl)amino)-1,4-oxazepane-4-carboxylate |  |

---

```
Reaction no 312
```

Generated by the Chemistry Development Kit (http://github.com/cdk)

|  | A | B |
| --- | --- | --- |
| 0 | ADD 5-methylmercapto-3-(o-nitrobenzyl)-1,2,4-triazole | ADD 5-methylmercapto-3-(o-nitrobenzyl)-1,2,4-triazole |
| 1 | ADD THF | ADD THF |
| 2 | ADD titanium trichloride at 0 °C | ADD titanium trichloride |
| 3 | STIR for 86400 s at 25 °C | ADD water |
| 4 | ADD water | STIR for 86400 s at 25 °C |
| 5 | SETTEMPERATURE 0 °C | PH with ammonium hydroxide to pH basic |
| 6 | PH with ammonium hydroxide to pH basic | EXTRACT with ethyl acetate |
| 7 | EXTRACT with dichloromethane | COLLECTLAYER organic |
| 8 | DRYSOLUTION | WASH with brine |
| 9 | CONCENTRATE | DRYSOLUTION over sodium sulfate |
| 10 | RECRYSTALLIZE from toluene | CONCENTRATE |
| 11 | YIELD 3-(o-aminobenzyl)-5-methylmercapto-1,2,4-triazole | YIELD 3-(o-aminobenzyl)-5-methylmercapto-1,2,4-triazole |

---

```
Reaction no 313
```

Generated by the Chemistry Development Kit (http://github.com/cdk)

|  | A | B |
| --- | --- | --- |
| 0 | ADD ethyl 4-{[(benzyloxy)carbonyl]amino}-2-methyl-3-oxobutanoate | ADD ethyl 4-{[(benzyloxy)carbonyl]amino}-2-methyl-3-oxobutanoate |
| 1 | ADD methanol | ADD methanol |
| 2 | ADD sodium borohydride at -70 °C | ADD sodium borohydride at 0 °C |
| 3 | SETTEMPERATURE -30 °C | STIR for 3600 s at 0 °C |
| 4 | ADD ammonium chloride at -30 °C | ADD ammonium chloride |
| 5 | EXTRACT with ethyl acetate | EXTRACT with ethyl acetate |
| 6 | COLLECTLAYER organic | WASH with brine |
| 7 | WASH with sodium chloride | DRYSOLUTION over magnesium sulfate |
| 8 | DRYSOLUTION over Na2SO4 | CONCENTRATE |
| 9 | FILTER keep filtrate | PURIFY |
| 10 | CONCENTRATE | YIELD Ethyl 4-{[(benzyloxy)carbonyl]amino}-3-hydroxy-2-methylbutanoate |
| 11 | ADD acetonitrile |  |
| 12 | PURIFY |  |
| 13 | YIELD Ethyl 4-{[(benzyloxy)carbonyl]amino}-3-hydroxy-2-methylbutanoate |  |

---

```
Reaction no 314
```

Generated by the Chemistry Development Kit (http://github.com/cdk)

|  | A | B |
| --- | --- | --- |
| 0 | ADD NaOH | ADD hydroxylamine hydrochloride |
| 1 | ADD methanol | ADD NaOH |
| 2 | ADD water | ADD methanol |
| 3 | SETTEMPERATURE -30 °C | ADD Methyl 7-(2,3-di-p-tolyl-7,8-dihydropyrido[2,3-b]pyrazin-5(6H)-yl)-3-oxoheptanoate |
| 4 | MAKESOLUTION with Methyl 7-(2,3-di-p-tolyl-7,8-dihydropyrido[2,3-b]pyrazin-5(6H)-yl)-3-oxoheptanoate and methanol | STIR for 86400 s at 25 °C |
| 5 | ADD SLN | ADD water |
| 6 | WAIT for 600 s | PH with HCl to pH acidic |
| 7 | MAKESOLUTION with hydroxylamine hydrochloride and NaOH and methanol and water | EXTRACT with ethyl acetate |
| 8 | ADD SLN | COLLECTLAYER organic |
| 9 | STIR for 3600 s at -30 °C | DRYSOLUTION over Na2SO4 |
| 10 | ADD HCl | CONCENTRATE |
| 11 | ADD methanol | YIELD 5-(4-(2,3-Di-p-tolyl-7,8-dihydropyrido[2,3-b]pyrazin-5(6H)-yl)butyl)isoxazol-3-ol |
| 12 | ADD water |  |
| 13 | STIR for 3600 s at 100 °C |  |
| 14 | SETTEMPERATURE 25 °C |  |
| 15 | CONCENTRATE |  |
| 16 | PURIFY |  |
| 17 | YIELD brine |  |
| 18 | PURIFY |  |
| 19 | YIELD 5-(4-(2,3-Di-p-tolyl-7,8-dihydropyrido[2,3-b]pyrazin-5(6H)-yl)butyl)isoxazol-3-ol |  |

---

```
Reaction no 315
```

Generated by the Chemistry Development Kit (http://github.com/cdk)

|  | A | B |
| --- | --- | --- |
| 0 | ADD 2,3-dihydro-benzo[1,4]dioxine-6-carbaldehyde | ADD 2,3-dihydro-benzo[1,4]dioxine-6-carbaldehyde |
| 1 | ADD malonic acid | ADD malonic acid |
| 2 | ADD pyridine | ADD piperidine |
| 3 | ADD piperidine | ADD pyridine |
| 4 | REFLUX for 86400 s | STIR for 3600 s at 100 °C |
| 5 | ADD malonic acid | CONCENTRATE |
| 6 | REFLUX for 86400 s | ADD water |
| 7 | CONCENTRATE | FILTER keep precipitate |
| 8 | ADD sodium hydroxide | DRYSOLID under vacuum |
| 9 | COLLECTLAYER aqueous | YIELD (E)-3-(2,3-dihydrobenzo[b][1,4]dioxin-6-yl)acrylic acid |
| 10 | WASH with dichloromethane |  |
| 11 | PH with HCl to pH acidic |  |
| 12 | FILTER keep precipitate |  |
| 13 | WASH with water |  |
| 14 | WASH with hexane |  |
| 15 | YIELD (E)-3-(2,3-dihydrobenzo[b][1,4]dioxin-6-yl)acrylic acid |  |

---

```
Reaction no 316
```

Generated by the Chemistry Development Kit (http://github.com/cdk)

|  | A | B |
| --- | --- | --- |
| 0 | ADD morpholine-3-carboxylic acid | ADD 4-chloro-2-fluoro-5-isopropoxyaniline |
| 1 | ADD dichloromethane | ADD dichloromethane |
| 2 | ADD trimethylaluminum at 0 °C | ADD trimethylaluminum dropwise at 0 °C |
| 3 | STIR for 600 s at 0 °C | STIR for 86400 s at 25 °C |
| 4 | MAKESOLUTION with 4-chloro-2-fluoro-5-isopropoxyaniline and dichloromethane | ADD morpholine-3-carboxylic acid at 25 °C |
| 5 | ADD SLN | STIR for 86400 s at 25 °C |
| 6 | STIR for 86400 s at 25 °C | ADD HCl dropwise at 0 °C |
| 7 | ADD HCl | FILTER keep filtrate |
| 8 | STIR for 3600 s at 25 °C | ADD water |
| 9 | EXTRACT with dichloromethane | PH with NaOH to pH basic |
| 10 | COLLECTLAYER organic | ADD dichloromethane |
| 11 | DRYSOLUTION over Na2SO4 | PHASESEPARATION |
| 12 | FILTER keep filtrate | COLLECTLAYER organic |
| 13 | CONCENTRATE | DRYSOLUTION over MgSO4 |
| 14 | PURIFY | CONCENTRATE |
| 15 | YIELD N-[4-Chloro-2-fluoro-5-(1-methylethoxy)phenyl]morpholine-3-carboxamide | YIELD N-[4-Chloro-2-fluoro-5-(1-methylethoxy)phenyl]morpholine-3-carboxamide |

---

```
Reaction no 317
```

Generated by the Chemistry Development Kit (http://github.com/cdk)

|  | A | B |
| --- | --- | --- |
| 0 | ADD 2-[2-(3-Phenyl-5-chloro-1-methyl-1H-pyrazol-4-yl)vinyl]benzoic acid | ADD 2-[2-(3-Phenyl-5-chloro-1-methyl-1H-pyrazol-4-yl)vinyl]benzoic acid |
| 1 | ADD ethanol | ADD ethanol |
| 2 | ADD Pd/C | ADD Pd/C at 25 °C |
| 3 | STIR for 86400 s at 25 °C | FILTER keep filtrate |
| 4 | FILTER keep filtrate | CONCENTRATE |
| 5 | CONCENTRATE | YIELD 2-[2-(3-Phenyl-5-chloro-1-methyl-1H-pyrazol-4-yl)ethyl]benzoic acid |
| 6 | YIELD 2-[2-(3-Phenyl-5-chloro-1-methyl-1H-pyrazol-4-yl)ethyl]benzoic acid |  |

---

```
Reaction no 318
```

Generated by the Chemistry Development Kit (http://github.com/cdk)

|  | A | B |
| --- | --- | --- |
| 0 | ADD methyl 2-chloro-6-formylbenzoate | ADD methyl 2-chloro-6-formylbenzoate |
| 1 | ADD tert-butyl piperazine-1-carboxylate | ADD tert-butyl piperazine-1-carboxylate |
| 2 | ADD 1,2-dichloroethane | ADD 1,2-dichloroethane |
| 3 | STIR for 3600 s at 25 °C | STIR for 3600 s at 25 °C |
| 4 | ADD sodium triacetoxyborohydride | ADD sodium triacetoxyborohydride |
| 5 | ADD water | STIR for 86400 s at 25 °C |
| 6 | EXTRACT with dichloromethane | ADD water |
| 7 | COLLECTLAYER organic | EXTRACT with dichloromethane |
| 8 | WASH with brine | COLLECTLAYER organic |
| 9 | DRYSOLUTION over sodium sulfate | WASH with brine |
| 10 | FILTER keep filtrate | DRYSOLUTION over Na2SO4 |
| 11 | CONCENTRATE | FILTER keep filtrate |
| 12 | PURIFY | CONCENTRATE |
| 13 | YIELD 2-((4-(tert-butoxycarbonyl)piperazin-1-yl)methyl)-6-chlorobenzoic acid | PURIFY |
| 14 |  | YIELD 2-((4-(tert-butoxycarbonyl)piperazin-1-yl)methyl)-6-chlorobenzoic acid |

---

```
Reaction no 319
```

Generated by the Chemistry Development Kit (http://github.com/cdk)

|  | A | B |
| --- | --- | --- |
| 0 | ADD 7-[α-(2-t-Butoxycarbonylaminomethyl-1-cyclohexenyl)acetamido]-3-(5-methyl-1,3,4-thiadiazol-2-ylthiomethyl)-3-cephem-4-carboxylic acid | ADD TFA |
| 1 | ADD TFA | ADD 7-[α-(2-t-Butoxycarbonylaminomethyl-1-cyclohexenyl)acetamido]-3-(5-methyl-1,3,4-thiadiazol-2-ylthiomethyl)-3-cephem-4-carboxylic acid |
| 2 | STIR for 3600 s at 25 °C | STIR for 3600 s at 25 °C |
| 3 | ADD ether | ADD ether |
| 4 | FILTER keep precipitate | ADD 7-[α-(2-aminomethyl-1-cyclohexenyl)acetamido]-3-(5-methyl-1,3,4-thiadiazol-2-ylthiomethyl)-3-cephem-4-carboxylic acid |
| 5 | WASH with ether | PH with ammonium hydroxide to pH acidic |
| 6 | DRYSOLID | ADD acetonitrile |
| 7 | YIELD 7-[α-(2-aminomethyl-1-cyclohexenyl)acetamido]-3-(5-methyl-1,3,4-thiadiazol-2-ylthiomethyl)-3-cephem-4-carboxylic acid |  |

---

```
Reaction no 320
```

Generated by the Chemistry Development Kit (http://github.com/cdk)

|  | A | B |
| --- | --- | --- |
| 0 | ADD 1-benzylpiperidine-4-carboxylic acid | ADD 1-benzylpiperidine-4-carboxylic acid |
| 1 | ADD dichloromethane | ADD dichloromethane |
| 2 | ADD 1-(3-dimethylaminopropyl)-3-ethylcarbodiimide hydrochloride | ADD 1-hydroxybenzotriazole hydrate |
| 3 | ADD 1-hydroxybenzotriazole hydrate | ADD 1-(3-dimethylaminopropyl)-3-ethylcarbodiimide hydrochloride |
| 4 | STIR for 600 s | ADD 4-(aminomethyl)benzonitrile hydrochloride |
| 5 | SETTEMPERATURE 0 °C | ADD DIPEA |
| 6 | ADD 4-(aminomethyl)benzonitrile hydrochloride | STIR for 86400 s at 25 °C |
| 7 | ADD DIPEA | WASH with water |
| 8 | STIR for 86400 s | COLLECTLAYER organic |
| 9 | CONCENTRATE | DRYSOLUTION over sodium sulfate |
| 10 | PARTITION with ethyl acetate and NaHCO3 | FILTER keep filtrate |
| 11 | COLLECTLAYER aqueous | CONCENTRATE |
| 12 | EXTRACT with ethyl acetate | PURIFY |
| 13 | COLLECTLAYER organic | YIELD 1-Benzyl-N-[(4-cyanophenyl)methyl]piperidine-4-carboxamide |
| 14 | WASH with brine |  |
| 15 | DRYSOLUTION over MgSO4 |  |
| 16 | CONCENTRATE |  |
| 17 | YIELD 1-Benzyl-N-[(4-cyanophenyl)methyl]piperidine-4-carboxamide |  |

---

```
Reaction no 321
```

Generated by the Chemistry Development Kit (http://github.com/cdk)

|  | A | B |
| --- | --- | --- |
| 0 | ADD ethanethiol | ADD carbon tetrachloride |
| 1 | ADD carbon tetrachloride | ADD chloroform |
| 2 | ADD triethylamine | ADD ethanethiol at 0 °C |
| 3 | ADD tributyltin chloride dropwise over 600 s | ADD triethylamine at 0 °C |
| 4 | STIR for 604800 s at 25 °C | ADD tributyltin chloride at 0 °C |
| 5 | ADD chloroform | STIR for 3600 s at 0 °C |
| 6 | FILTER keep filtrate | STIR for 3600 s at 25 °C |
| 7 | WASH with acetic acid | CONCENTRATE |
| 8 | WASH with water | YIELD tributyl(ethylthio)stannane |
| 9 | CONCENTRATE |  |
| 10 | YIELD tributyl(ethylthio)stannane |  |

---

```
Reaction no 322
```

Generated by the Chemistry Development Kit (http://github.com/cdk)

|  | A | B |
| --- | --- | --- |
| 0 | ADD Dimethyl ‌((methylsulfonyl)methyl)phosphonate | ADD Dimethyl ‌((methylsulfonyl)methyl)phosphonate |
| 1 | ADD THF | ADD THF |
| 2 | SETTEMPERATURE 0 °C | ADD NaOtBu |
| 3 | ADD NaOtBu | STIR for 600 s at 25 °C |
| 4 | STIR for 3600 s at 0 °C | MAKESOLUTION with (S)-tert-butyl 2-formylpyrrolidine-1-carboxylate and THF |
| 5 | MAKESOLUTION with (S)-tert-butyl 2-formylpyrrolidine-1-carboxylate and THF | ADD SLN |
| 6 | ADD SLN | STIR for 3600 s at 25 °C |
| 7 | STIR for 3600 s at 0 °C | QUENCH with water |
| 8 | SETTEMPERATURE 25 °C | EXTRACT with ethyl acetate |
| 9 | STIR for 3600 s at 25 °C | COLLECTLAYER organic |
| 10 | QUENCH with sodium bicarbonate | WASH with brine |
| 11 | STIR for 3600 s | DRYSOLUTION over sodium sulfate |
| 12 | EXTRACT with dichloromethane | FILTER keep filtrate |
| 13 | COLLECTLAYER organic | CONCENTRATE |
| 14 | DRYSOLUTION over sodium sulfate | PURIFY |
| 15 | FILTER keep filtrate | YIELD Tert-butyl ‌(2S)-2-(1-hydroxy-2-(methylsulfonyl)ethyl)pyrrolidine-1-carboxylate |
| 16 | CONCENTRATE |  |
| 17 | PURIFY |  |
| 18 | YIELD Tert-butyl ‌(2S)-2-(1-hydroxy-2-(methylsulfonyl)ethyl)pyrrolidine-1-carboxylate |  |

---

```
Reaction no 323
```

Generated by the Chemistry Development Kit (http://github.com/cdk)

|  | A | B |
| --- | --- | --- |
| 0 | ADD 3-phenoxybenzyl chloride | ADD 3-phenoxybenzyl chloride |
| 1 | ADD acetic acid | ADD acetic acid |
| 2 | ADD water | ADD hexamethylenetetramine |
| 3 | ADD hexamethylenetetramine | STIR for 28800 s at 100 °C |
| 4 | REFLUX for 3600 s | SETTEMPERATURE 25 °C |
| 5 | ADD HCl | ADD water |
| 6 | REFLUX for 600 s | PH with HCl to pH acidic |
| 7 | SETTEMPERATURE 25 °C | EXTRACT with ethyl acetate |
| 8 | EXTRACT with ether | COLLECTLAYER organic |
| 9 | WASH with water | WASH with water |
| 10 | WASH with sodium bicarbonate | DRYSOLUTION over magnesium sulfate |
| 11 | WASH with water | CONCENTRATE |
| 12 | DRYSOLUTION over magnesium sulfate | PURIFY |
| 13 | CONCENTRATE | YIELD 3-phenoxybenzaldehyde |
| 14 | YIELD 3-phenoxybenzaldehyde |  |

---

```
Reaction no 324
```

Generated by the Chemistry Development Kit (http://github.com/cdk)

|  | A | B |
| --- | --- | --- |
| 0 | ADD 2-{2-[6-amino-3-(3-bromophenyl)-7-chloro-2-oxo-1,2-dihydroquinolin-4-yloxy]-1-ethyl}-piperidine-1-carboxylic acid tert-butyl ester | ADD 2-{2-[6-amino-3-(3-bromophenyl)-7-chloro-2-oxo-1,2-dihydroquinolin-4-yloxy]-1-ethyl}-piperidine-1-carboxylic acid tert-butyl ester |
| 1 | ADD pyridine | ADD dichloromethane |
| 2 | ADD triphosgene at 0 °C | ADD pyridine at 0 °C |
| 3 | STIR for 3600 s at 25 °C | ADD triphosgene at 0 °C |
| 4 | SETTEMPERATURE 0 °C | STIR for 3600 s at 0 °C |
| 5 | ADD cyclopropylamine | ADD cyclopropylamine |
| 6 | STIR for 86400 s at 25 °C | STIR for 3600 s at 25 °C |
| 7 | ADD ethyl acetate | ADD ethyl acetate |
| 8 | WASH with water | WASH with ammonium chloride |
| 9 | WASH with brine | WASH with brine |
| 10 | DRYSOLUTION over sodium sulfate | DRYSOLUTION over magnesium sulfate |
| 11 | CONCENTRATE | CONCENTRATE |
| 12 | PURIFY | PURIFY |
| 13 | YIELD 2-{2-[3-(3-bromophenyl)-7-chloro-6-(3-cyclopropyl-ureido)-2-oxo-1,2-dihydroquinolin-4-yloxy]-ethyl}-piperidine-1-carboxylic acid tert-butyl ester | YIELD 2-{2-[3-(3-bromophenyl)-7-chloro-6-(3-cyclopropyl-ureido)-2-oxo-1,2-dihydroquinolin-4-yloxy]-ethyl}-piperidine-1-carboxylic acid tert-butyl ester |

---

```
Reaction no 325
```

Generated by the Chemistry Development Kit (http://github.com/cdk)

|  | A | B |
| --- | --- | --- |
| 0 | ADD (5R)-4-[3,5-bis(trifluoromethyl)benzoyl]-1-(carboxymethyl)-5-(1H-indol-3-ylmethyl)piperazin-2-one | ADD (5R)-4-[3,5-bis(trifluoromethyl)benzoyl]-1-(carboxymethyl)-5-(1H-indol-3-ylmethyl)piperazin-2-one |
| 1 | ADD HOBt | ADD dichloromethane |
| 2 | ADD dichloromethane | ADD HOBt |
| 3 | ADD 1-(3-dimethylaminopropyl)-3-ethylcarbodiimide hydrochloride at 0 °C | ADD 1-(3-dimethylaminopropyl)-3-ethylcarbodiimide hydrochloride |
| 4 | STIR for 3600 s | STIR for 3600 s at 25 °C |
| 5 | ADD ammonia at 0 °C | ADD ammonia |
| 6 | STIR for 3600 s | STIR for 86400 s at 25 °C |
| 7 | STIR for 600 s at 25 °C | CONCENTRATE |
| 8 | EXTRACT with ethyl acetate | PURIFY |
| 9 | COLLECTLAYER organic | YIELD (5R)-4-[3,5-bis(trifluoromethyl)benzoyl]-1-(carbamoylmethyl)-5-(1H-indol-3-ylmethyl)piperazin-2-one |
| 10 | WASH with sodium bicarbonate |  |
| 11 | WASH with water |  |
| 12 | WASH with brine |  |
| 13 | DRYSOLUTION over magnesium sulfate |  |
| 14 | CONCENTRATE |  |
| 15 | PURIFY |  |
| 16 | ADD diisopropyl ether |  |
| 17 | FILTER keep precipitate |  |
| 18 | WASH with diisopropyl ether |  |
| 19 | DRYSOLID |  |
| 20 | YIELD (5R)-4-[3,5-bis(trifluoromethyl)benzoyl]-1-(carbamoylmethyl)-5-(1H-indol-3-ylmethyl)piperazin-2-one |  |

---

```
Reaction no 326
```

Generated by the Chemistry Development Kit (http://github.com/cdk)

|  | A | B |
| --- | --- | --- |
| 0 | ADD 5-hexyl-2-[p-(trans-4-(p-anisyloxymethyl)cyclohexyl)phenyl]pyridine | ADD 5-hexyl-2-[p-(trans-4-(p-anisyloxymethyl)cyclohexyl)phenyl]pyridine |
| 1 | ADD acetonitrile | ADD acetonitrile |
| 2 | ADD water | ADD water |
| 3 | ADD ammonium nitrate | ADD ammonium nitrate at 0 °C |
| 4 | STIR for 86400 s at 25 °C | WAIT for 600 s |
| 5 | ADD water | ADD water |
| 6 | EXTRACT with ethyl acetate | EXTRACT with ether |
| 7 | COLLECTLAYER organic | COLLECTLAYER organic |
| 8 | WASH with brine | WASH with water |
| 9 | DRYSOLUTION over MgSO4 | DRYSOLUTION over magnesium sulfate |
| 10 | FILTER keep filtrate | CONCENTRATE |
| 11 | CONCENTRATE | PURIFY |
| 12 | PURIFY | YIELD 5-hexyl-2-[p-(trans-4-(hydroxymethyl)-cyclohexyl)phenyl]pyridine |
| 13 | YIELD 5-hexyl-2-[p-(trans-4-(hydroxymethyl)-cyclohexyl)phenyl]pyridine |  |

---

```
Reaction no 327
```

Generated by the Chemistry Development Kit (http://github.com/cdk)

|  | A | B |
| --- | --- | --- |
| 0 | ADD (5R,7S)-5-[4-(Trifluoromethoxy)benzyl]-3-(2,6-dichloro-4-pyridyl)-7-amino-1,3-diazabicyclo[3.3.0]octane-2,4-dione | ADD (5R,7S)-5-[4-(Trifluoromethoxy)benzyl]-3-(2,6-dichloro-4-pyridyl)-7-amino-1,3-diazabicyclo[3.3.0]octane-2,4-dione |
| 1 | ADD THF | ADD THF |
| 2 | ADD DIPEA | ADD DIPEA at 25 °C |
| 3 | ADD methyl chloroformate | ADD methyl chloroformate at 25 °C |
| 4 | WAIT for 28800 s | STIR for 86400 s at 25 °C |
| 5 | CONCENTRATE | CONCENTRATE |
| 6 | PURIFY | PURIFY |
| 7 | YIELD (5R, 7S)-5-[4-(Trifluoromethoxy)benzyl]-3-(2,6-dichloro-4-pyridyl)-7-methoxycarbonylamino-1,3-diazabicyclo[3.3.0]octane-2,4-dione | YIELD (5R, 7S)-5-[4-(Trifluoromethoxy)benzyl]-3-(2,6-dichloro-4-pyridyl)-7-methoxycarbonylamino-1,3-diazabicyclo[3.3.0]octane-2,4-dione |

---

```
Reaction no 328
```

Generated by the Chemistry Development Kit (http://github.com/cdk)

|  | A | B |
| --- | --- | --- |
| 0 | ADD phenylacetonitrile | ADD phenylacetonitrile |
| 1 | ADD N,N-dimethylformamide dimethyl acetal | ADD toluene |
| 2 | ADD toluene | ADD N,N-dimethylformamide dimethyl acetal |
| 3 | REFLUX for 28800 s | REFLUX for 86400 s |
| 4 | CONCENTRATE | CONCENTRATE |
| 5 | YIELD (E)-3-(dimethylamino)-2-phenylacrylonitrile | PURIFY |
| 6 |  | YIELD (E)-3-(dimethylamino)-2-phenylacrylonitrile |

---

```
Reaction no 329
```

Generated by the Chemistry Development Kit (http://github.com/cdk)

|  | A | B |
| --- | --- | --- |
| 0 | ADD (S)-1-(5-Amino-pyridin-2-yl)-pyrrolidin-3-ol | ADD (S)-1-(5-Amino-pyridin-2-yl)-pyrrolidin-3-ol |
| 1 | ADD pyridine | ADD acetonitrile |
| 2 | ADD acetonitrile | ADD pyridine |
| 3 | ADD phenyl chloroformate | ADD phenyl chloroformate |
| 4 | STIR for 3600 s at 25 °C | STIR for 3600 s at 25 °C |
| 5 | ADD water | ADD water |
| 6 | EXTRACT with ethyl acetate | EXTRACT with ethyl acetate |
| 7 | COLLECTLAYER organic | COLLECTLAYER organic |
| 8 | CONCENTRATE | CONCENTRATE |
| 9 | PURIFY | PURIFY |
| 10 | YIELD (S)-Phenyl 6-(3-hydroxypyrrolidin-1-yl)pyridin-3-ylcarbamate | YIELD (S)-Phenyl 6-(3-hydroxypyrrolidin-1-yl)pyridin-3-ylcarbamate |

---

```
Reaction no 330
```

Generated by the Chemistry Development Kit (http://github.com/cdk)

|  | A | B |
| --- | --- | --- |
| 0 | ADD 8-cyano-1,3-dihydro-5-[p-(trifluoromethyl)phenyl]-2H-1,4-benzodiazepine-2-thione | ADD 8-cyano-1,3-dihydro-5-[p-(trifluoromethyl)phenyl]-2H-1,4-benzodiazepine-2-thione |
| 1 | ADD ethanol | ADD ethanol |
| 2 | ADD butyric acid hydrazide | ADD butyric acid hydrazide |
| 3 | STIR | REFLUX for 28800 s |
| 4 | YIELD 9-cyano-1-propyl-6-[p-(trifluoromethyl)phenyl]-4H-s-triazolo[4,3-a][1,4]benzodiazepine | CONCENTRATE |
| 5 |  | PURIFY |
| 6 |  | YIELD 9-cyano-1-propyl-6-[p-(trifluoromethyl)phenyl]-4H-s-triazolo[4,3-a][1,4]benzodiazepine |

---

```
Reaction no 331
```

Generated by the Chemistry Development Kit (http://github.com/cdk)

|  | A | B |
| --- | --- | --- |
| 0 | ADD 6-N-[5-cyclopropyl-1-[2-(oxan-2-yloxy)ethyl]-1H-pyrazol-3-yl]-1-N,1-N-bis[(2,4-dimethoxyphenyl)methyl]-3-(4-methylpyridin-3-yl)-2,7-naphthyridine-1,6-diamine | ADD 6-N-[5-cyclopropyl-1-[2-(oxan-2-yloxy)ethyl]-1H-pyrazol-3-yl]-1-N,1-N-bis[(2,4-dimethoxyphenyl)methyl]-3-(4-methylpyridin-3-yl)-2,7-naphthyridine-1,6-diamine |
| 1 | ADD TFA | ADD TFA |
| 2 | STIR for 3600 s at 60 °C | STIR for 3600 s at 60 °C |
| 3 | CONCENTRATE | WAIT for 3600 s at 60 °C |
| 4 | PURIFY | CONCENTRATE |
| 5 | YIELD 2-(3-[[8-amino-6-(4-methylpyridin-3-yl)-2,7-naphthyridin-3-yl]amino]-5-cyclopropyl-1H-pyrazol-1-yl)ethan-1-ol | PURIFY |
| 6 |  | YIELD 2-(3-[[8-amino-6-(4-methylpyridin-3-yl)-2,7-naphthyridin-3-yl]amino]-5-cyclopropyl-1H-pyrazol-1-yl)ethan-1-ol |

---

```
Reaction no 332
```

Generated by the Chemistry Development Kit (http://github.com/cdk)

|  | A | B |
| --- | --- | --- |
| 0 | ADD methyl 4-iodobenzoate | ADD methyl 4-iodobenzoate |
| 1 | ADD NaHCO3 | ADD Pd(OAc)2 |
| 2 | ADD allyl alcohol | ADD NaHCO3 |
| 3 | ADD tetrabutylammonium bromide | ADD tetrabutylammonium bromide |
| 4 | ADD DMF | ADD DMF |
| 5 | DEGAS with $6$ for 600 s | DEGAS with $6$ for 600 s |
| 6 | ADD Pd(OAc)2 | ADD allyl alcohol |
| 7 | STIR for 604800 s at 25 °C | STIR for 86400 s at 60 °C |
| 8 | FILTER keep filtrate | QUENCH with water |
| 9 | ADD water | EXTRACT with ethyl acetate |
| 10 | EXTRACT with ethyl acetate | COLLECTLAYER organic |
| 11 | COLLECTLAYER organic | WASH with water |
| 12 | DRYSOLUTION over Na2SO4 | WASH with brine |
| 13 | CONCENTRATE | DRYSOLUTION over Na2SO4 |
| 14 | PURIFY | FILTER keep filtrate |
| 15 | YIELD 4-(3-Oxo-propyl)-benzoic acid methyl ester | CONCENTRATE |
| 16 |  | PURIFY |
| 17 |  | YIELD 4-(3-Oxo-propyl)-benzoic acid methyl ester |

---

```
Reaction no 333
```

Generated by the Chemistry Development Kit (http://github.com/cdk)

|  | A | B |
| --- | --- | --- |
| 0 | ADD tributylphosphine | ADD tributylphosphine |
| 1 | ADD methyl 4-hydroxyphenylacetate | ADD methyl 4-hydroxyphenylacetate |
| 2 | ADD (Z)-3-(4-bromo-phenyl)-3-phenyl-prop-2-en-1-ol | ADD (Z)-3-(4-bromo-phenyl)-3-phenyl-prop-2-en-1-ol |
| 3 | ADD THF | ADD THF |
| 4 | ADD (E)-diazene-1,2-diylbis(piperidin-1-ylmethanone) at 0 °C | ADD (E)-diazene-1,2-diylbis(piperidin-1-ylmethanone) at 0 °C |
| 5 | STIR for 3600 s | STIR for 3600 s |
| 6 | FILTER keep filtrate | FILTER keep filtrate |
| 7 | CONCENTRATE | CONCENTRATE |
| 8 | PURIFY | PURIFY |
| 9 | YIELD (Z)-{4-[3-(4-Bromo-phenyl)-3-phenyl-allyloxy]-phenyl}-acetic acid methyl ester | YIELD (Z)-{4-[3-(4-Bromo-phenyl)-3-phenyl-allyloxy]-phenyl}-acetic acid methyl ester |

---

```
Reaction no 334
```

Generated by the Chemistry Development Kit (http://github.com/cdk)

|  | A | B |
| --- | --- | --- |
| 0 | ADD 2-(6-phenyl-1,2,3,4-tetrahydronaphthalene-2-carbonyl)oxazole-5-carboxamide | ADD 2-(6-phenyl-1,2,3,4-tetrahydronaphthalene-2-carbonyl)oxazole-5-carboxamide |
| 1 | ADD pyridine | ADD dioxane |
| 2 | ADD dioxane | ADD pyridine |
| 3 | ADD trifluoroacetic anhydride at 0 °C | ADD trifluoroacetic anhydride |
| 4 | STIR for 3600 s at 25 °C | STIR for 3600 s at 25 °C |
| 5 | ADD dichloromethane | ADD dichloromethane |
| 6 | WASH with NaHCO3 | COLLECTLAYER organic |
| 7 | WASH with brine | WASH with sodium chloride |
| 8 | DRYSOLUTION over Na2SO4 | DRYSOLUTION over Na2SO4 |
| 9 | CONCENTRATE | PURIFY |
| 10 | PURIFY | YIELD 2-(6-Phenyl-1,2,3,4-tetrahydronaphthalene-2-carbonyl)oxazole-5-carbonitrile |
| 11 | YIELD 2-(6-Phenyl-1,2,3,4-tetrahydronaphthalene-2-carbonyl)oxazole-5-carbonitrile | PHASESEPARATION |

---

```
Reaction no 335
```

Generated by the Chemistry Development Kit (http://github.com/cdk)

|  | A | B |
| --- | --- | --- |
| 0 | ADD n-Propanesulfonic acid [6-(2-hydroxy-ethoxy)-5-(2-methoxy-phenoxy)-[2,2′]bipyrimidinyl-4-yl]-amide | ADD n-Propanesulfonic acid [6-(2-hydroxy-ethoxy)-5-(2-methoxy-phenoxy)-[2,2′]bipyrimidinyl-4-yl]-amide |
| 1 | ADD THF | ADD THF |
| 2 | ADD NaH | ADD NaH |
| 3 | ADD 5-bromo-2-chloropyrimidine | ADD 5-bromo-2-chloropyrimidine |
| 4 | STIR for 86400 s at 60 °C | STIR for 86400 s at 60 °C |
| 5 | ADD water | ADD water |
| 6 | PH with citric acid to pH acidic | PH with citric acid to pH acidic |
| 7 | FILTER | FILTER |
| 8 | PURIFY | PURIFY |
| 9 | YIELD n-propanesulfonic acid [6-[2-(5-bromo-pyrimidin-2-yloxy)-ethoxy]-5-(2-methoxy-phenoxy)-[2,2′]bipyridinyl-4-yl]-amide | YIELD n-propanesulfonic acid [6-[2-(5-bromo-pyrimidin-2-yloxy)-ethoxy]-5-(2-methoxy-phenoxy)-[2,2′]bipyridinyl-4-yl]-amide |

---

```
Reaction no 336
```

Generated by the Chemistry Development Kit (http://github.com/cdk)

|  | A | B |
| --- | --- | --- |
| 0 | ADD 2-amino-6-methylpyridine | ADD 2-amino-6-methylpyridine |
| 1 | ADD tert-butanol | ADD tert-butanol |
| 2 | ADD di-tert-butyl dicarbonate | ADD di-tert-butyl dicarbonate |
| 3 | ADD DMAP | ADD DMAP |
| 4 | STIR for 86400 s at 100 °C | STIR for 28800 s at 25 °C |
| 5 | CONCENTRATE | CONCENTRATE |
| 6 | PURIFY | ADD ethyl acetate |
| 7 | YIELD (6-methyl-pyridin-2-yl)-carbamic acid tert-butyl ester | WASH with NaHCO3 |
| 8 |  | COLLECTLAYER organic |
| 9 |  | DRYSOLUTION over MgSO4 |
| 10 |  | CONCENTRATE |
| 11 |  | PURIFY |
| 12 |  | YIELD (6-methyl-pyridin-2-yl)-carbamic acid tert-butyl ester |

---

```
Reaction no 337
```

Generated by the Chemistry Development Kit (http://github.com/cdk)

|  | A | B |
| --- | --- | --- |
| 0 | ADD 2-chloropyrimidine-4-carboxylic acid chloride | ADD 2-chloropyrimidine-4-carboxylic acid chloride |
| 1 | ADD n-butanol | ADD n-butanol |
| 2 | STIR for 3600 s at 25 °C | REFLUX for 3600 s |
| 3 | ADD NaHCO3 | SETTEMPERATURE 25 °C |
| 4 | EXTRACT with ethyl acetate | ADD NaHCO3 |
| 5 | COLLECTLAYER organic | EXTRACT with ethyl acetate |
| 6 | WASH with sodium chloride | COLLECTLAYER organic |
| 7 | DRYSOLUTION over Na2SO4 | WASH with water |
| 8 | CONCENTRATE | WASH with brine |
| 9 | DRYSOLID under vacuum | DRYSOLUTION over magnesium sulfate |
| 10 | YIELD 2-chloropyrimidine-4-carboxylic Acid Butyl Ester | CONCENTRATE |
| 11 |  | PURIFY |
| 12 |  | YIELD 2-chloropyrimidine-4-carboxylic Acid Butyl Ester |

---

```
Reaction no 338
```

Generated by the Chemistry Development Kit (http://github.com/cdk)

|  | A | B |
| --- | --- | --- |
| 0 | ADD ethyl ‌(4-formyl-2-methylphenoxy)acetate | ADD 4′-(trifluoromethyl)-1,1′-biphenyl-3-ylamine |
| 1 | ADD dichloromethane | ADD ethyl ‌(4-formyl-2-methylphenoxy)acetate |
| 2 | ADD 4′-(trifluoromethyl)-1,1′-biphenyl-3-ylamine | ADD dichloromethane |
| 3 | STIR for 600 s at 25 °C | ADD acetic acid |
| 4 | ADD sodium triacetoxyborohydride | STIR for 3600 s at 25 °C |
| 5 | ADD acetic acid | ADD sodium triacetoxyborohydride |
| 6 | STIR for 86400 s | STIR for 86400 s at 25 °C |
| 7 | PH with sodium bicarbonate to pH basic | CONCENTRATE |
| 8 | EXTRACT with dichloromethane | PURIFY |
| 9 | PHASESEPARATION | YIELD ethyl [2-methyl-4-({[4′-(trifluoromethyl)-1,1′-biphenyl-3-yl]amino}methyl)phenoxy]acetate |
| 10 | COLLECTLAYER organic |  |
| 11 | CONCENTRATE |  |
| 12 | PURIFY |  |
| 13 | YIELD ethyl [2-methyl-4-({[4′-(trifluoromethyl)-1,1′-biphenyl-3-yl]amino}methyl)phenoxy]acetate |  |

---

```
Reaction no 339
```

Generated by the Chemistry Development Kit (http://github.com/cdk)

|  | A | B |
| --- | --- | --- |
| 0 | ADD diacetic acid iodobenzene | ADD diacetic acid iodobenzene |
| 1 | ADD dichloromethane | ADD dichloromethane |
| 2 | ADD trifluoromethanesulfonic acid dropwise at 0 °C | ADD trifluoromethanesulfonic acid dropwise at 0 °C |
| 3 | STIR for 3600 s | ADD 1,2-bis(trimethylsilyl)benzene |
| 4 | MAKESOLUTION with 1,2-bis(trimethylsilyl)benzene and dichloromethane | STIR for 3600 s at 0 °C |
| 5 | ADD SLN at 0 °C | STIR for 3600 s at 25 °C |
| 6 | STIR for 3600 s at 25 °C | CONCENTRATE |
| 7 | TRITURATE with ether | WASH with ether |
| 8 | FILTER keep precipitate | DRYSOLID under vacuum |
| 9 | YIELD (phenyl)[2-(trimethylsilyl)phenyl]iodonium triflate | YIELD (phenyl)[2-(trimethylsilyl)phenyl]iodonium triflate |

---

```
Reaction no 340
```

Generated by the Chemistry Development Kit (http://github.com/cdk)

|  | A | B |
| --- | --- | --- |
| 0 | ADD 6-ethoxy-2-methylthio-5-nitro-4-(1-oxido-thiomorpholino)-pyrimidine | ADD 6-ethoxy-2-methylthio-5-nitro-4-(1-oxido-thiomorpholino)-pyrimidine |
| 1 | ADD piperazine | ADD piperazine |
| 2 | ADD DMSO | ADD DMSO |
| 3 | STIR for 3600 s at 100 °C | STIR for 28800 s at 25 °C |
| 4 | PURIFY | ADD ice water |
| 5 | YIELD 6-Ethoxy-5-nitro-4-(1-oxido-thiomorpholino)-2-piperazino-pyrimidine | FILTER keep precipitate |
| 6 |  | WASH with water |
| 7 |  | RECRYSTALLIZE from ethanol |
| 8 |  | YIELD 6-Ethoxy-5-nitro-4-(1-oxido-thiomorpholino)-2-piperazino-pyrimidine |

---

```
Reaction no 341
```

Generated by the Chemistry Development Kit (http://github.com/cdk)

|  | A | B |
| --- | --- | --- |
| 0 | ADD tert-Butyl ‌(S)-1-(5-(3-methyl-2-oxo-2,3-dihydro-1H-benzo[d]imidazol-5-yl)-1,3,4-thiadiazol-2-yl-boc-amino)-3-(4-(trifluoromethyl)phenyl)propan-2-ylcarbamate | ADD tert-Butyl ‌(S)-1-(5-(3-methyl-2-oxo-2,3-dihydro-1H-benzo[d]imidazol-5-yl)-1,3,4-thiadiazol-2-yl-boc-amino)-3-(4-(trifluoromethyl)phenyl)propan-2-ylcarbamate |
| 1 | ADD dichloromethane | ADD dichloromethane |
| 2 | ADD TFA | ADD TFA over 3600 s |
| 3 | STIR for 3600 s at 25 °C | CONCENTRATE |
| 4 | CONCENTRATE | ADD ethyl acetate |
| 5 | PURIFY | WASH with sodium bicarbonate |
| 6 | YIELD 6-(5-((S)-2-amino-3-(4-(trifluoromethyl)phenyl)propylamino)-1,3,4-thiadiazol-2-yl)-1-methyl-1H-benzo[d]imidazol-2(3H)-one | WASH with NaOH |
| 7 |  | WASH with sodium bicarbonate |
| 8 |  | CONCENTRATE |
| 9 |  | YIELD 6-(5-((S)-2-amino-3-(4-(trifluoromethyl)phenyl)propylamino)-1,3,4-thiadiazol-2-yl)-1-methyl-1H-benzo[d]imidazol-2(3H)-one |

---

```
Reaction no 342
```

Generated by the Chemistry Development Kit (http://github.com/cdk)

|  | A | B |
| --- | --- | --- |
| 0 | ADD 4-(methylthio)phenol | ADD 4-(methylthio)phenol |
| 1 | ADD sulfolane | ADD sulfolane |
| 2 | ADD NaOH | ADD 1-chloro-2,4-dinitrobenzene |
| 3 | SETTEMPERATURE 60 °C | ADD NaOH |
| 4 | ADD 1-chloro-2,4-dinitrobenzene | STIR for 28800 s at 100 °C |
| 5 | STIR for 28800 s at 60 °C | SETTEMPERATURE 25 °C |
| 6 | FILTER keep precipitate | FILTER keep filtrate |
| 7 | RECRYSTALLIZE from ethanol | CONCENTRATE |
| 8 | YIELD 1-(4-(methylthio)phenoxy)-2,4-dinitrobenzene | RECRYSTALLIZE from ethanol |
| 9 |  | YIELD 1-(4-(methylthio)phenoxy)-2,4-dinitrobenzene |

---

```
Reaction no 343
```

Generated by the Chemistry Development Kit (http://github.com/cdk)

|  | A | B |
| --- | --- | --- |
| 0 | ADD (2R,3S)-2-(2,4-difluorophenyl)-3-methyl-2-[(1H-1,2,4-triazol-1-yl)methyl]oxirane | ADD (2R,3S)-2-(2,4-difluorophenyl)-3-methyl-2-[(1H-1,2,4-triazol-1-yl)methyl]oxirane |
| 1 | ADD NMP | ADD 3H-Pyrrolo[1,2-d][1,2,4]triazin-4-one |
| 2 | ADD K2CO3 | ADD K2CO3 |
| 3 | ADD 3H-Pyrrolo[1,2-d][1,2,4]triazin-4-one | ADD NMP |
| 4 | STIR for 604800 s at 100 °C | STIR for 86400 s at 100 °C |
| 5 | ADD water | SETTEMPERATURE 25 °C |
| 6 | EXTRACT with ethyl acetate | ADD water |
| 7 | DRYSOLUTION over Na2SO4 | EXTRACT with ethyl acetate |
| 8 | CONCENTRATE | COLLECTLAYER organic |
| 9 | PURIFY | WASH with water |
| 10 | YIELD 3-[(1R,2R)-2-(2,4-Difluorophenyl)-2-hydroxy-1-methyl-3-(1,2,4-triazol-1-yl)propyl]-3H-pyrrolo[1,2-d][1,2,4]triazin-4-one | WASH with brine |
| 11 |  | DRYSOLUTION over magnesium sulfate |
| 12 |  | CONCENTRATE |
| 13 |  | PURIFY |
| 14 |  | YIELD 3-[(1R,2R)-2-(2,4-Difluorophenyl)-2-hydroxy-1-methyl-3-(1,2,4-triazol-1-yl)propyl]-3H-pyrrolo[1,2-d][1,2,4]triazin-4-one |

---

```
Reaction no 344
```

Generated by the Chemistry Development Kit (http://github.com/cdk)

|  | A | B |
| --- | --- | --- |
| 0 | ADD sodium | ADD sodium |
| 1 | ADD p-cresol | ADD p-cresol |
| 2 | ADD 3-chloro-6-allylthiopyridazine | ADD 3-chloro-6-allylthiopyridazine |
| 3 | STIR for 28800 s at 100 °C | STIR for 28800 s at 100 °C |
| 4 | ADD 3-(4-methylphenoxy)-6-allylthiopyridazine | WAIT for 28800 s at 100 °C |
| 5 | YIELD 3-(4-methylphenoxy)-6-allylthiopyridazine | SETTEMPERATURE 25 °C |
| 6 |  | PH with NaOH to pH basic |
| 7 |  | EXTRACT with ether |
| 8 |  | WASH with water |
| 9 |  | DRYSOLUTION over magnesium sulfate |
| 10 |  | CONCENTRATE |
| 11 |  | SETTEMPERATURE 25 °C |
| 12 |  | YIELD 3-(4-methylphenoxy)-6-allylthiopyridazine |

---

```
Reaction no 345
```

Generated by the Chemistry Development Kit (http://github.com/cdk)

|  | A | B |
| --- | --- | --- |
| 0 | ADD p-anisidine | ADD p-anisidine |
| 1 | ADD pyridine | ADD pyridine |
| 2 | ADD 6-chloronicotinoyl chloride at 0 °C | ADD 6-chloronicotinoyl chloride |
| 3 | STIR for 86400 s at 25 °C | STIR for 28800 s at 100 °C |
| 4 | ADD water | STIR for 28800 s at 100 °C |
| 5 | FILTER keep precipitate | ADD water |
| 6 | WASH with water | FILTER keep precipitate |
| 7 | DRYSOLID under vacuum | WASH with sodium bicarbonate |
| 8 | YIELD 6-chloro-N-(4-methoxyphenyl)nicotinamide | CONCENTRATE |
| 9 |  | YIELD 6-chloro-N-(4-methoxyphenyl)nicotinamide |

---

```
Reaction no 346
```

Generated by the Chemistry Development Kit (http://github.com/cdk)

|  | A | B |
| --- | --- | --- |
| 0 | ADD Ethyl 3-amino-1-[4-(methyloxy)phenyl]-1H-pyrrole-2-carboxylate at 25 °C | ADD Ethyl 3-amino-1-[4-(methyloxy)phenyl]-1H-pyrrole-2-carboxylate |
| 1 | ADD cyanoacetic acid at 25 °C | ADD cyanoacetic acid |
| 2 | ADD triethylamine at 25 °C | ADD triethylamine |
| 3 | ADD acetonitrile at 25 °C | ADD acetonitrile |
| 4 | MAKESOLUTION with DCC and acetonitrile | ADD DCC at 0 °C |
| 5 | ADD SLN dropwise at 25 °C | STIR for 86400 s at 25 °C |
| 6 | STIR for 604800 s | FILTER keep filtrate |
| 7 | CONCENTRATE | CONCENTRATE |
| 8 | TRITURATE with ethanol | PURIFY |
| 9 | FILTER keep precipitate | YIELD ethyl 3-[(cyanoacetyl)amino]-1-[4-(methyloxy)phenyl]-1H-pyrrole-2-carboxylate |
| 10 | DRYSOLID |  |
| 11 | YIELD ethyl 3-[(cyanoacetyl)amino]-1-[4-(methyloxy)phenyl]-1H-pyrrole-2-carboxylate |  |

---

```
Reaction no 347
```

Generated by the Chemistry Development Kit (http://github.com/cdk)

|  | A | B |
| --- | --- | --- |
| 0 | ADD 4,6-dichloro-5-nitropyrimidine | ADD dichloromethane |
| 1 | ADD dichloromethane | ADD ammonia |
| 2 | MAKESOLUTION with tert-butyl piperazine-1-carboxylate and triethylamine and dichloromethane | ADD 4,6-dichloro-5-nitropyrimidine |
| 3 | ADD SLN dropwise at -70 °C | STIR for 3600 s at 25 °C |
| 4 | STIR for 3600 s at -70 °C | CONCENTRATE |
| 5 | ADD ammonia | ADD DMF |
| 6 | STIR for 3600 s at 25 °C | ADD tert-butyl piperazine-1-carboxylate |
| 7 | ADD water | ADD triethylamine |
| 8 | ADD chlorure de sodium | STIR for 28800 s at 100 °C |
| 9 | EXTRACT with dichloromethane | SETTEMPERATURE 25 °C |
| 10 | COLLECTLAYER organic | ADD water |
| 11 | DRYSOLUTION over Na2SO4 | ADD chlorure de sodium |
| 12 | FILTER keep filtrate | FILTER keep precipitate |
| 13 | CONCENTRATE | WASH with water |
| 14 | YIELD 4-(6-amino-5-nitro-4-pyrimidinyl)-1-piperazinecarboxylic acid tert-butyl ester | DRYSOLID |
| 15 |  | YIELD 4-(6-amino-5-nitro-4-pyrimidinyl)-1-piperazinecarboxylic acid tert-butyl ester |

---

```
Reaction no 348
```

Generated by the Chemistry Development Kit (http://github.com/cdk)

|  | A | B |
| --- | --- | --- |
| 0 | ADD 3-[4,5-Dimethoxy-2-pyrimidinyl]-6-fluoro-4-oxo-2-thioxo-1,2,3,4-tetrahydro-7-quinazolinecarboxylic acid | ADD 3-[4,5-Dimethoxy-2-pyrimidinyl]-6-fluoro-4-oxo-2-thioxo-1,2,3,4-tetrahydro-7-quinazolinecarboxylic acid |
| 1 | ADD DMF | ADD DMF |
| 2 | ADD DIPEA | ADD DIPEA |
| 3 | ADD HATU | ADD HATU |
| 4 | STIR for 3600 s | STIR for 3600 s |
| 5 | ADD 3-chlorobenzylamine | ADD 3-chlorobenzylamine |
| 6 | STIR for 86400 s at 25 °C | STIR for 86400 s at 25 °C |
| 7 | PURIFY | PURIFY |
| 8 | YIELD N-(3-Chlorobenzyl)-3-[4,5-dimethoxy-2-pyrimidinyl]-6-fluoro-4-oxo-2-thioxo-1,2,3,4-tetrahydro-7-quinazolinecarboxamide | YIELD N-(3-Chlorobenzyl)-3-[4,5-dimethoxy-2-pyrimidinyl]-6-fluoro-4-oxo-2-thioxo-1,2,3,4-tetrahydro-7-quinazolinecarboxamide |

---

```
Reaction no 349
```

Generated by the Chemistry Development Kit (http://github.com/cdk)

|  | A | B |
| --- | --- | --- |
| 0 | ADD (1H-indazol-5-yl)-[2-(1,2,3,6-tetrahydropyridin-4-yl)-1H-pyrrolo[2,3-b]pyridin4-yl]-amine trihydrochloride | ADD (1H-indazol-5-yl)-[2-(1,2,3,6-tetrahydropyridin-4-yl)-1H-pyrrolo[2,3-b]pyridin4-yl]-amine trihydrochloride |
| 1 | ADD DMF | ADD DIPEA |
| 2 | ADD DIPEA | ADD DMF |
| 3 | ADD tert-butyl isocyanate | ADD tert-butyl isocyanate at 0 °C |
| 4 | STIR for 3600 s at 25 °C | STIR for 86400 s at 25 °C |
| 5 | CONCENTRATE | PURIFY |
| 6 | PURIFY | YIELD 4-[4-(1H-Indazol-5-ylamino)-1H-pyrrolo[2,3-b]pyridin-2-yl]-3,6-dihydro-2H-pyridine-1-carboxylic acid tert-butylamide |
| 7 | YIELD 4-[4-(1H-Indazol-5-ylamino)-1H-pyrrolo[2,3-b]pyridin-2-yl]-3,6-dihydro-2H-pyridine-1-carboxylic acid tert-butylamide |  |

---

```
Reaction no 350
```

Generated by the Chemistry Development Kit (http://github.com/cdk)

|  | A | B |
| --- | --- | --- |
| 0 | ADD trans-(R)-2-(tert-butyl)-6-chloro-7-((3-(3-fluoropyrrolidin-1-yl)cyclobutyl)sulfonyl)benzo[d]oxazole | ADD trans-(R)-2-(tert-butyl)-6-chloro-7-((3-(3-fluoropyrrolidin-1-yl)cyclobutyl)sulfonyl)benzo[d]oxazole |
| 1 | ADD dioxane | ADD dioxane |
| 2 | ADD water | ADD water |
| 3 | ADD sulfuric acid | ADD sulfuric acid |
| 4 | STIR for 86400 s at 100 °C | STIR for 86400 s at 100 °C |
| 5 | SETTEMPERATURE 25 °C | SETTEMPERATURE 25 °C |
| 6 | CONCENTRATE | CONCENTRATE |
| 7 | PH with NaOH to pH ˜ at 0 °C | PH with NaOH to pH about 0 °C |
| 8 | EXTRACT with ethyl acetate | EXTRACT with ethyl acetate |
| 9 | COLLECTLAYER organic | COLLECTLAYER organic |
| 10 | DRYSOLUTION over sodium sulfate | DRYSOLUTION over sodium sulfate |
| 11 | FILTER keep filtrate | FILTER keep filtrate |
| 12 | CONCENTRATE | CONCENTRATE |
| 13 | YIELD trans-(R)-6-amino-3-chloro-2-((3-(3-fluoropyrrolidin-1-yl)cyclobutyl)sulfonyl)phenol | YIELD trans-(R)-6-amino-3-chloro-2-((3-(3-fluoropyrrolidin-1-yl)cyclobutyl)sulfonyl)phenol |

---

```
Reaction no 351
```

Generated by the Chemistry Development Kit (http://github.com/cdk)

|  | A | B |
| --- | --- | --- |
| 0 | ADD 1-[2-(8-Chloro-7-methyl-2,4-dioxo-3,4-dihydro-2H-benzo[g]pteridin-10-yl)-ethyl]-piperidine-4-carboxylic acid | ADD 1-[2-(8-Chloro-7-methyl-2,4-dioxo-3,4-dihydro-2H-benzo[g]pteridin-10-yl)-ethyl]-piperidine-4-carboxylic acid |
| 1 | ADD DMSO | ADD cyclopentylamine |
| 2 | ADD cyclopentylamine at 25 °C | ADD DMSO |
| 3 | STIR for 86400 s at 60 °C | STIR for 86400 s at 100 °C |
| 4 | ADD cyclopentylamine | SETTEMPERATURE 25 °C |
| 5 | STIR for 28800 s | ADD water |
| 6 | SETTEMPERATURE 25 °C | PURIFY |
| 7 | ADD water | YIELD 1-[2-(8-Cyclopentylamino-7-methyl-2,4-dioxo-3,4-dihydro-2H-benzo[g]pteridin-10-yl)-ethyl]-piperidine-4-carboxylic acid |
| 8 | PURIFY |  |
| 9 | YIELD 1-[2-(8-Cyclopentylamino-7-methyl-2,4-dioxo-3,4-dihydro-2H-benzo[g]pteridin-10-yl)-ethyl]-piperidine-4-carboxylic acid |  |

---

```
Reaction no 352
```

Generated by the Chemistry Development Kit (http://github.com/cdk)

|  | A | B |
| --- | --- | --- |
| 0 | ADD 4-(3-nitro-phenyl)-thiazol-2-ylamine hydrochloride | ADD 4-(3-nitro-phenyl)-thiazol-2-ylamine hydrochloride |
| 1 | ADD 4-chlorobenzenesulfonyl chloride | ADD 4-chlorobenzenesulfonyl chloride |
| 2 | ADD pyridine | ADD pyridine |
| 3 | STIR for 86400 s at 25 °C | STIR for 86400 s |
| 4 | ADD HCl | ADD HCl |
| 5 | EXTRACT with ethyl acetate | PHASESEPARATION |
| 6 | COLLECTLAYER organic | COLLECTLAYER organic |
| 7 | WASH with brine | MAKESOLUTION with ethanol and sodium hydroxide |
| 8 | DRYSOLUTION over magnesium sulfate | ADD SLN |
| 9 | CONCENTRATE | ADD charcoal |
| 10 | PURIFY | STIR for 3600 s at 25 °C |
| 11 | YIELD 4-chloro-N-[4-(3-nitro-phenyl)-thiazol-2-yl]-benzenesulfonamide | FILTER keep precipitate |
| 12 |  | PHASESEPARATION |
| 13 |  | RECRYSTALLIZE from ethanol |
| 14 |  | YIELD 4-chloro-N-[4-(3-nitro-phenyl)-thiazol-2-yl]-benzenesulfonamide |

---

```
Reaction no 353
```

Generated by the Chemistry Development Kit (http://github.com/cdk)

|  | A | B |
| --- | --- | --- |
| 0 | ADD acetic anhydride | ADD acetic anhydride |
| 1 | ADD formic acid dropwise at 0 °C | ADD formic acid dropwise at 0 °C |
| 2 | STIR for 3600 s at 60 °C | STIR for 3600 s at 60 °C |
| 3 | SETTEMPERATURE 25 °C | SETTEMPERATURE 25 °C |
| 4 | ADD THF | ADD THF |
| 5 | MAKESOLUTION with 1-(6-bromopyridin-2-yl)-N-(2,4-dimethoxybenzyl)methanamine and THF | ADD 1-(6-bromopyridin-2-yl)-N-(2,4-dimethoxybenzyl)methanamine at 0 °C over 600 s |
| 6 | ADD SLN dropwise at 0 °C | ADD THF at 0 °C over 600 s |
| 7 | STIR for 86400 s at 25 °C | STIR for 3600 s at 25 °C |
| 8 | CONCENTRATE | CONCENTRATE |
| 9 | PURIFY | PURIFY |
| 10 | YIELD N-((6-Bromopyridin-2-yl)methyl)-N-(2,4-dimethoxybenzyl)formamide | YIELD N-((6-Bromopyridin-2-yl)methyl)-N-(2,4-dimethoxybenzyl)formamide |

---

```
Reaction no 354
```

Generated by the Chemistry Development Kit (http://github.com/cdk)

|  | A | B |
| --- | --- | --- |
| 0 | ADD 2-(3-Methoxy-propyl)-4-(methyl-{1-[2-methyl-6-(3-trifluoromethyl-phenyl)-pyridin-3-yl]-butyl}-amino)-phenoxy-acetic acid ethyl ester | ADD 2-(3-Methoxy-propyl)-4-(methyl-{1-[2-methyl-6-(3-trifluoromethyl-phenyl)-pyridin-3-yl]-butyl}-amino)-phenoxy-acetic acid ethyl ester |
| 1 | ADD THF | ADD THF EtOH |
| 2 | ADD THF EtOH | ADD NaOH |
| 3 | ADD NaOH at 0 °C | STIR for 3600 s at 60 °C |
| 4 | STIR for 3600 s at 25 °C | ADD HCl |
| 5 | PH with HCl to pH neutral | CONCENTRATE |
| 6 | EXTRACT with ethyl acetate | FILTER keep precipitate |
| 7 | COLLECTLAYER organic | WASH with water |
| 8 | WASH with water | DRYSOLID under vacuum |
| 9 | DRYSOLUTION over sodium sulfate | YIELD [rac]-[2-(3-Methoxy-propyl)-4-(methyl-{1-[2-methyl-6-(3-trifluoromethyl-phenyl)-pyridin-3-yl]-butyl}-amino)-phenoxy]-acetic acid |
| 10 | CONCENTRATE |  |
| 11 | YIELD [rac]-[2-(3-Methoxy-propyl)-4-(methyl-{1-[2-methyl-6-(3-trifluoromethyl-phenyl)-pyridin-3-yl]-butyl}-amino)-phenoxy]-acetic acid |  |

---

```
Reaction no 355
```

Generated by the Chemistry Development Kit (http://github.com/cdk)

|  | A | B |
| --- | --- | --- |
| 0 | ADD tert-butyl ‌((5-chloro-2,4-difluorophenyl)sulfonyl)(thiazol-4-yl)carbamate | ADD tert-butyl ‌((5-chloro-2,4-difluorophenyl)sulfonyl)(thiazol-4-yl)carbamate |
| 1 | ADD DMF | ADD DMF |
| 2 | ADD (R)-1-benzyl-pyrrolidin-3-ylamine | ADD (R)-1-benzyl-pyrrolidin-3-ylamine at 0 °C |
| 3 | STIR for 3600 s at 25 °C | STIR for 86400 s at 25 °C |
| 4 | ADD water | ADD water |
| 5 | EXTRACT with ethyl acetate | FILTER keep precipitate |
| 6 | COLLECTLAYER organic | WASH with water |
| 7 | WASH with brine | YIELD tert-butyl ‌(R)-((4-((1-benzylpyrrolidin-3-yl)amino)-5-chloro-2-fluorophenyl)sulfonyl)(thiazol-4-yl)carbamate |
| 8 | DRYSOLUTION over sodium sulfate |  |
| 9 | FILTER keep filtrate |  |
| 10 | CONCENTRATE |  |
| 11 | PURIFY |  |
| 12 | YIELD tert-butyl ‌(R)-((4-((1-benzylpyrrolidin-3-yl)amino)-5-chloro-2-fluorophenyl)sulfonyl)(thiazol-4-yl)carbamate |  |

---

```
Reaction no 356
```

Generated by the Chemistry Development Kit (http://github.com/cdk)

|  | A | B |
| --- | --- | --- |
| 0 | ADD methyl 5-{[(tert-butoxycarbonyl)amino]methyl}-2,6-diisobutyl-4-(4-methylphenyl)nicotinate | ADD toluene |
| 1 | ADD toluene | ADD methyl 5-{[(tert-butoxycarbonyl)amino]methyl}-2,6-diisobutyl-4-(4-methylphenyl)nicotinate |
| 2 | SETTEMPERATURE -70 °C | ADD diisobutylaluminum hydride toluene dropwise at -70 °C |
| 3 | ADD diisobutylaluminum hydride toluene dropwise over 3600 s | STIR for 3600 s at -70 °C |
| 4 | STIR for 3600 s at -70 °C | ADD methanol |
| 5 | STIR for 3600 s at 0 °C | ADD sodium sulfate decahydrate |
| 6 | ADD methanol | STIR for 3600 s at 25 °C |
| 7 | STIR for 600 s | FILTER keep filtrate |
| 8 | ADD sodium sulfate decahydrate | CONCENTRATE |
| 9 | STIR for 3600 s | PURIFY |
| 10 | FILTER keep precipitate | YIELD tert-butyl {[5-(hydroxymethyl)-2,6-diisobutyl-4-(4-methylphenyl)pyridin-3-yl]methyl}carbamate |
| 11 | WASH with ethyl acetate |  |
| 12 | COLLECTLAYER organic |  |
| 13 | CONCENTRATE |  |
| 14 | PURIFY |  |
| 15 | YIELD tert-butyl {[5-(hydroxymethyl)-2,6-diisobutyl-4-(4-methylphenyl)pyridin-3-yl]methyl}carbamate |  |

---

```
Reaction no 357
```

Generated by the Chemistry Development Kit (http://github.com/cdk)

|  | A | B |
| --- | --- | --- |
| 0 | ADD 6-bromo-1,2-dimethylquinolinium triflate | ADD 6-bromo-1,2-dimethylquinolinium triflate |
| 1 | ADD 2,5-dimethyl-1-phenyl-1H-pyrrole-3-carbaldehyde | ADD 2,5-dimethyl-1-phenyl-1H-pyrrole-3-carbaldehyde |
| 2 | ADD methanol | ADD methanol |
| 3 | ADD piperidine | ADD piperidine |
| 4 | REFLUX for 28800 s | REFLUX for 28800 s |
| 5 | STIR for 28800 s at 25 °C | REFLUX for 28800 s under vacuum |
| 6 | FILTER keep precipitate | REFLUX for 28800 s |
| 7 | YIELD 6-bromo-2-[(E)-2-(2,5-dimethyl-1-phenyl-1H-pyrrol-3-yl)-vinyl]-1-methyl-quinolinium triflate | FILTER keep precipitate |
| 8 |  | YIELD 6-bromo-2-[(E)-2-(2,5-dimethyl-1-phenyl-1H-pyrrol-3-yl)-vinyl]-1-methyl-quinolinium triflate |

---

```
Reaction no 358
```

Generated by the Chemistry Development Kit (http://github.com/cdk)

|  | A | B |
| --- | --- | --- |
| 0 | ADD 7-Bromo-9-methyl-2,3,4,9-tetrahydro-1H-carbazol-1-one | ADD 7-Bromo-9-methyl-2,3,4,9-tetrahydro-1H-carbazol-1-one |
| 1 | ADD ethanol | ADD hydroxylamine hydrochloride |
| 2 | ADD water | ADD sodium acetate |
| 3 | ADD hydroxylamine hydrochloride | ADD ethanol |
| 4 | ADD sodium acetate | REFLUX for 86400 s |
| 5 | REFLUX for 86400 s | SETTEMPERATURE 25 °C |
| 6 | SETTEMPERATURE 25 °C | ADD water |
| 7 | CONCENTRATE | FILTER keep precipitate |
| 8 | TRITURATE with methanol | DRYSOLID under vacuum |
| 9 | DRYSOLID | YIELD 7-Bromo-9-methyl-2,3,4,9-tetrahydro-1H-carbazol-1-one oxime |
| 10 | YIELD 7-Bromo-9-methyl-2,3,4,9-tetrahydro-1H-carbazol-1-one oxime |  |

---

```
Reaction no 359
```

Generated by the Chemistry Development Kit (http://github.com/cdk)

|  | A | B |
| --- | --- | --- |
| 0 | ADD N-(4-bromo-3-fluorophenyl)cyclopropanesulfonamide | ADD N-(4-bromo-3-fluorophenyl)cyclopropanesulfonamide |
| 1 | ADD bis(pinacolato)diboron | ADD bis(pinacolato)diboron |
| 2 | ADD potassium acetate | ADD Pd(dppf)Cl2 |
| 3 | ADD Pd(dppf)Cl2 | ADD potassium acetate |
| 4 | ADD dioxane | ADD dioxane |
| 5 | STIR for 86400 s at 100 °C | STIR for 86400 s at 100 °C |
| 6 | ADD water | SETTEMPERATURE 25 °C |
| 7 | ADD water | ADD water |
| 8 | EXTRACT with ethyl acetate | EXTRACT with ethyl acetate |
| 9 | COLLECTLAYER organic | COLLECTLAYER organic |
| 10 | CONCENTRATE | WASH with brine |
| 11 | PURIFY | DRYSOLUTION over Na2SO4 |
| 12 | YIELD N-(3-fluoro-4-(4,4,5,5-tetramethyl-1,3,2-dioxaborolan-2-yl)phenyl) cyclopropanesulfonamide | CONCENTRATE |
| 13 |  | PURIFY |
| 14 |  | YIELD N-(3-fluoro-4-(4,4,5,5-tetramethyl-1,3,2-dioxaborolan-2-yl)phenyl) cyclopropanesulfonamide |

---

```
Reaction no 360
```

Generated by the Chemistry Development Kit (http://github.com/cdk)

|  | A | B |
| --- | --- | --- |
| 0 | ADD tert-Butyl 1-cyano-1-phenyl-6-azaspiro[2.5]octane-6-carboxylate | ADD tert-Butyl 1-cyano-1-phenyl-6-azaspiro[2.5]octane-6-carboxylate |
| 1 | ADD dichloromethane | ADD dichloromethane |
| 2 | ADD TFA at 0 °C | ADD TFA |
| 3 | STIR for 86400 s at 25 °C | STIR for 3600 s at 25 °C |
| 4 | CONCENTRATE | CONCENTRATE |
| 5 | ADD dichloromethane | YIELD 1-phenyl-6-azaspiro[2.5]octane-1-carbonitrile |
| 6 | COLLECTLAYER organic |  |
| 7 | WASH with NaHCO3 |  |
| 8 | WASH with brine |  |
| 9 | DRYSOLUTION over Na2SO4 |  |
| 10 | YIELD 1-phenyl-6-azaspiro[2.5]octane-1-carbonitrile |  |

---

```
Reaction no 361
```

Generated by the Chemistry Development Kit (http://github.com/cdk)

|  | A | B |
| --- | --- | --- |
| 0 | ADD (2E)-5-(tert-Butyloxycarbonylamino)-5-methylhex-2-enoic acid | ADD (2E)-5-(tert-Butyloxycarbonylamino)-5-methylhex-2-enoic acid |
| 1 | ADD HOAt | ADD HOAt |
| 2 | ADD 1-(3-dimethylaminopropyl)-3-ethylcarbodiimide hydrochloride at 0 °C | ADD dichloromethane |
| 3 | STIR for 600 s at 0 °C | ADD DMF |
| 4 | MAKESOLUTION with (2S)-6-acetylamino-2-{(2R)-2-[N-methyl-N-(2-(methylamino)-3-(2-naphthyl)propionyl)amino]-3-phenylpropionylamino}hexanoic acid N,N-dimethylamide and DMF and dichloromethane and DIPEA | SETTEMPERATURE 0 °C |
| 5 | ADD SLN | ADD 1-(3-dimethylaminopropyl)-3-ethylcarbodiimide hydrochloride |
| 6 | STIR for 86400 s | STIR for 600 s at 0 °C |
| 7 | SETTEMPERATURE 25 °C | MAKESOLUTION with (2S)-6-acetylamino-2-{(2R)-2-[N-methyl-N-(2-(methylamino)-3-(2-naphthyl)propionyl)amino]-3-phenylpropionylamino}hexanoic acid N,N-dimethylamide and dichloromethane and DIPEA |
| 8 | ADD ethyl acetate | ADD SLN |
| 9 | WASH with NaHSO4 | STIR for 86400 s |
| 10 | COLLECTLAYER aqueous | SETTEMPERATURE 25 °C |
| 11 | EXTRACT with ethyl acetate | ADD ethyl acetate |
| 12 | COLLECTLAYER organic | WASH with NaHSO4 |
| 13 | WASH with NaHCO3 | COLLECTLAYER aqueous |
| 14 | DRYSOLUTION over magnesium sulfate | EXTRACT with ethyl acetate |
| 15 | CONCENTRATE | COLLECTLAYER organic |
| 16 | PURIFY | WASH with NaHCO3 |
| 17 | YIELD {(3E)4-[N-((1R)-1-{N-[(1R)-1-((1S)-5-acetylamino-1-(dimethylcarbamoyl)pentylcarbamoyl)-2-phenylethyl]-N-methylcarbamoyl}2-(2-naphthyl)ethyl)-N-methylcarbamoyl]-1,1-dimethylbut-3-enyl}carbamic acid tert-butyl ester | DRYSOLUTION over magnesium sulfate |
| 18 |  | CONCENTRATE |
| 19 |  | PURIFY |
| 20 |  | YIELD {(3E)4-[N-((1R)-1-{N-[(1R)-1-((1S)-5-acetylamino-1-(dimethylcarbamoyl)pentylcarbamoyl)-2-phenylethyl]-N-methylcarbamoyl}2-(2-naphthyl)ethyl)-N-methylcarbamoyl]-1,1-dimethylbut-3-enyl}carbamic acid tert-butyl ester |

---

```
Reaction no 362
```

Generated by the Chemistry Development Kit (http://github.com/cdk)

|  | A | B |
| --- | --- | --- |
| 0 | ADD methyl 3-dimethylaminoacrylate | ADD methyl 3-dimethylaminoacrylate |
| 1 | ADD dioxane | ADD triethylamine |
| 2 | ADD triethylamine | ADD dioxane |
| 3 | MAKESOLUTION with 2,4-dichloro-5-fluorobenzoyl chloride and dioxane | MAKESOLUTION with 2,4-dichloro-5-fluorobenzoyl chloride and dioxane |
| 4 | ADD SLN dropwise at 0 °C | ADD SLN dropwise at 25 °C |
| 5 | SETTEMPERATURE 0 °C | STIR for 3600 s at 25 °C |
| 6 | STIR for 28800 s at 25 °C | FILTER keep filtrate |
| 7 | STIR for 3600 s at 60 °C | CONCENTRATE |
| 8 | CONCENTRATE | YIELD methyl 3-dimethylamino-2-(2,4-dichloro-5-fluorobenzoyl)acrylate |
| 9 | ADD dichloromethane / water |  |
| 10 | PHASESEPARATION |  |
| 11 | COLLECTLAYER aqueous |  |
| 12 | EXTRACT with dichloromethane |  |
| 13 | COLLECTLAYER organic |  |
| 14 | WASH with water |  |
| 15 | DRYSOLUTION over Na2SO4 |  |
| 16 | CONCENTRATE |  |
| 17 | RECRYSTALLIZE from methanol / water |  |
| 18 | YIELD methyl 3-dimethylamino-2-(2,4-dichloro-5-fluorobenzoyl)acrylate |  |

---

```
Reaction no 363
```

Generated by the Chemistry Development Kit (http://github.com/cdk)

|  | A | B |
| --- | --- | --- |
| 0 | ADD 4-cyclopropyl-2-(spiro[4,6-dihydropyrrolo[1,2-b]pyrazole-5,1′-cyclopropane]-3-ylamino)-7-(2-trimethylsilylethoxymethyl)pyrrolo[2,3-d]pyrimidine-5-carbonitrile | ADD 4-cyclopropyl-2-(spiro[4,6-dihydropyrrolo[1,2-b]pyrazole-5,1′-cyclopropane]-3-ylamino)-7-(2-trimethylsilylethoxymethyl)pyrrolo[2,3-d]pyrimidine-5-carbonitrile |
| 1 | ADD dichloromethane | ADD dichloromethane |
| 2 | ADD TFA | ADD TFA at 0 °C |
| 3 | STIR for 28800 s at 60 °C | STIR for 28800 s at 25 °C |
| 4 | CONCENTRATE | CONCENTRATE |
| 5 | ADD K2CO3 | ADD THF |
| 6 | ADD THF | ADD water |
| 7 | ADD water | ADD K2CO3 |
| 8 | STIR for 86400 s at 100 °C | STIR for 86400 s at 25 °C |
| 9 | ADD ice water | ADD ice water |
| 10 | EXTRACT with ethyl acetate | EXTRACT with ethyl acetate |
| 11 | COLLECTLAYER organic | COLLECTLAYER organic |
| 12 | WASH with brine | WASH with brine |
| 13 | DRYSOLUTION over Na2SO4 | DRYSOLUTION over Na2SO4 |
| 14 | FILTER keep filtrate | FILTER keep filtrate |
| 15 | CONCENTRATE | CONCENTRATE |
| 16 | PURIFY | PURIFY |
| 17 | YIELD 4-cyclopropyl-2-(spiro[4,6-dihydropyrrolo[1,2-b]pyrazole-5,1′-cyclopropane]-3-ylamino)-7H-pyrrolo[2,3-d]pyrimidine-5-carbonitrile | YIELD 4-cyclopropyl-2-(spiro[4,6-dihydropyrrolo[1,2-b]pyrazole-5,1′-cyclopropane]-3-ylamino)-7H-pyrrolo[2,3-d]pyrimidine-5-carbonitrile |

---

```
Reaction no 364
```

Generated by the Chemistry Development Kit (http://github.com/cdk)

|  | A | B |
| --- | --- | --- |
| 0 | ADD difluoromethyldiphenylphosphine oxide | ADD difluoromethyldiphenylphosphine oxide |
| 1 | ADD THF | ADD THF |
| 2 | ADD LDA at -30 °C | ADD LDA at -30 °C |
| 3 | STIR for 3600 s | STIR for 3600 s |
| 4 | MAKESOLUTION with 3-tetrahydropyranyloxy-estra-1,3,5(10),7-tetraen-17-one and THF | MAKESOLUTION with 3-tetrahydropyranyloxy-estra-1,3,5(10),7-tetraen-17-one and THF |
| 5 | ADD SLN | ADD SLN |
| 6 | STIR for 3600 s at -30 °C | STIR for 600 s at -30 °C |
| 7 | REFLUX for 3600 s | REFLUX for 3600 s |
| 8 | ADD ethyl acetate | ADD ethyl acetate |
| 9 | ADD water | ADD water |
| 10 | WASH with water | WASH with water |
| 11 | WASH with sodium chloride | WASH with sodium chloride |
| 12 | DRYSOLUTION over sodium sulfate | DRYSOLUTION over sodium sulfate |
| 13 | CONCENTRATE | CONCENTRATE |
| 14 | PURIFY | PURIFY |
| 15 | YIELD 17-difluoromethylene-3-tetrahydropyranyloxy-estra-1,3,5(10),7-tetraene | YIELD 17-difluoromethylene-3-tetrahydropyranyloxy-estra-1,3,5(10),7-tetraene |

---

```
Reaction no 365
```

Generated by the Chemistry Development Kit (http://github.com/cdk)

|  | A | B |
| --- | --- | --- |
| 0 | ADD 3-(Chloroacetyl)-1-[(2-chloro-1,3-thiazol-5-yl)methyl]-1H-imidazo[1,2-a]pyridin-4-ium-2-olate | ADD Cs2CO3 |
| 1 | ADD DMF | ADD pyrazole |
| 2 | ADD pyrazole | ADD DMF |
| 3 | ADD Cs2CO3 | ADD 3-(Chloroacetyl)-1-[(2-chloro-1,3-thiazol-5-yl)methyl]-1H-imidazo[1,2-a]pyridin-4-ium-2-olate |
| 4 | STIR for 86400 s at 25 °C | STIR for 86400 s at 25 °C |
| 5 | ADD H2O DCM | ADD H2O DCM |
| 6 | COLLECTLAYER aqueous | COLLECTLAYER organic |
| 7 | EXTRACT with dichloromethane | DRYSOLUTION over magnesium sulfate |
| 8 | COLLECTLAYER organic | CONCENTRATE |
| 9 | DRYSOLUTION over MgSO4 | PURIFY |
| 10 | CONCENTRATE | YIELD 1-[(2-chloro-1,3-thiazol-5-yl)methyl]-3-(1H-pyrazol-1-ylacetyl)-1H-imidazo[1,2-a]pyridin-4-ium-2-olate |
| 11 | PURIFY |  |
| 12 | YIELD 1-[(2-chloro-1,3-thiazol-5-yl)methyl]-3-(1H-pyrazol-1-ylacetyl)-1H-imidazo[1,2-a]pyridin-4-ium-2-olate |  |

---

```
Reaction no 366
```

Generated by the Chemistry Development Kit (http://github.com/cdk)

|  | A | B |
| --- | --- | --- |
| 0 | ADD N-t-butoxycarbonyl-D-serine | ADD N-t-butoxycarbonyl-D-serine |
| 1 | ADD THF | ADD triethylamine |
| 2 | ADD triethylamine at 0 °C | ADD THF |
| 3 | STIR for 600 s | ADD ethyl chloroformate at 0 °C |
| 4 | SETTEMPERATURE -30 °C | STIR for 3600 s at 0 °C |
| 5 | ADD dl-3-amino-2,2,4,4-tetramethyltetrahydrothiophene | ADD dl-3-amino-2,2,4,4-tetramethyltetrahydrothiophene at 0 °C |
| 6 | SETTEMPERATURE 25 °C | STIR for 86400 s at 25 °C |
| 7 | ADD ethyl acetate | ADD ethyl acetate |
| 8 | WASH with ethyl chloroformate | WASH with water |
| 9 | WASH with brine | WASH with brine |
| 10 | COLLECTLAYER organic | DRYSOLUTION over magnesium sulfate |
| 11 | DRYSOLUTION over Na2SO4 | CONCENTRATE |
| 12 | CONCENTRATE | PURIFY |
| 13 | YIELD N(2,2,4,4-tetramethyltetrahydrothiophene-3-yl)-t-butoxycarbonyl-D-serine amide | YIELD N(2,2,4,4-tetramethyltetrahydrothiophene-3-yl)-t-butoxycarbonyl-D-serine amide |

---

```
Reaction no 367
```

Generated by the Chemistry Development Kit (http://github.com/cdk)

|  | A | B |
| --- | --- | --- |
| 0 | ADD NaH | ADD trimethylsulfoxonium iodide |
| 1 | ADD DMSO | ADD DMSO |
| 2 | ADD trimethylsulfoxonium iodide at 25 °C | ADD NaH |
| 3 | STIR for 3600 s | STIR for 3600 s at 25 °C |
| 4 | MAKESOLUTION with benzyl 4-formylpiperidine-1-carboxylate and DMSO | MAKESOLUTION with benzyl 4-formylpiperidine-1-carboxylate and DMSO |
| 5 | ADD SLN | ADD SLN |
| 6 | STIR for 3600 s at 25 °C | STIR for 86400 s at 25 °C |
| 7 | ADD water | ADD water |
| 8 | EXTRACT with ether | EXTRACT with ethyl acetate |
| 9 | COLLECTLAYER organic | COLLECTLAYER organic |
| 10 | WASH with water | WASH with brine |
| 11 | WASH with brine | DRYSOLUTION over sodium sulfate |
| 12 | DRYSOLUTION over Na2SO4 | CONCENTRATE |
| 13 | CONCENTRATE | PURIFY |
| 14 | YIELD Phenylmethyl 4-(2-oxiranyl)-1-piperidinecarboxylate | YIELD Phenylmethyl 4-(2-oxiranyl)-1-piperidinecarboxylate |

---

```
Reaction no 368
```

Generated by the Chemistry Development Kit (http://github.com/cdk)

|  | A | B |
| --- | --- | --- |
| 0 | ADD 1-(SR)-(3,5-Bis(trifluoromethyl)phenyl)methoxy-2-(SR)-phenyl-3-(RS)-(N-(benzyloxycarbonyl)-N-methylamino)cyclopentane | ADD 1-(SR)-(3,5-Bis(trifluoromethyl)phenyl)methoxy-2-(SR)-phenyl-3-(RS)-(N-(benzyloxycarbonyl)-N-methylamino)cyclopentane |
| 1 | ADD methanol | ADD ethyl acetate |
| 2 | ADD ethyl acetate | ADD methanol |
| 3 | ADD Pd/C | ADD Pd/C |
| 4 | STIR for 3600 s | STIR for 3600 s at 25 °C |
| 5 | FILTER keep filtrate | FILTER keep filtrate |
| 6 | CONCENTRATE | WASH with methanol |
| 7 | YIELD 1-(SR)-(3,5-Bis(trifluoromethyl)phenyl)methoxy-2-(SR)-phenyl-3-(RS)-(methylamino)cyclopentane | CONCENTRATE |
| 8 |  | YIELD 1-(SR)-(3,5-Bis(trifluoromethyl)phenyl)methoxy-2-(SR)-phenyl-3-(RS)-(methylamino)cyclopentane |

---

```
Reaction no 369
```

Generated by the Chemistry Development Kit (http://github.com/cdk)

|  | A | B |
| --- | --- | --- |
| 0 | ADD 2-hydroxyimino-4-(4-methoxy-phenyl)-but-3-enenitrile | ADD 2-hydroxyimino-4-(4-methoxy-phenyl)-but-3-enenitrile |
| 1 | ADD THF | ADD THF |
| 2 | SETTEMPERATURE 0 °C | ADD triethylamine |
| 3 | ADD methanesulfonyl chloride | ADD methanesulfonyl chloride |
| 4 | ADD triethylamine dropwise at 0 °C over 600 s | STIR for 86400 s at 25 °C |
| 5 | STIR for 3600 s at 0 °C | ADD water |
| 6 | ADD water | FILTER keep precipitate |
| 7 | EXTRACT with ethyl acetate | WASH with water |
| 8 | WASH with water | DRYSOLID |
| 9 | WASH with brine | YIELD 2-methylsulfonyloxyimino-4-(4-methoxy-phenyl)-but-3-enenitrile |
| 10 | DRYSOLUTION over magnesium sulfate |  |
| 11 | RECRYSTALLIZE from ethanol |  |
| 12 | YIELD 2-methylsulfonyloxyimino-4-(4-methoxy-phenyl)-but-3-enenitrile |  |

---

```
Reaction no 370
```

Generated by the Chemistry Development Kit (http://github.com/cdk)

|  | A | B |
| --- | --- | --- |
| 0 | ADD DMSO | ADD N1-[4-[3-(4-cyanophenyl)-5-trifluoromethyl-4,5-dihydroisoxazol-5-yl]-2-methylphenyl]-3-iodo-N2-isopropylphthalic diamide |
| 1 | ADD N1-[4-[3-(4-cyanophenyl)-5-trifluoromethyl-4,5-dihydroisoxazol-5-yl]-2-methylphenyl]-3-iodo-N2-isopropylphthalic diamide | ADD DMSO |
| 2 | ADD K2CO3 | ADD K2CO3 |
| 3 | ADD hydrogen peroxide dropwise at 25 °C | ADD hydrogen peroxide |
| 4 | STIR for 3600 s at 25 °C | STIR for 3600 s at 25 °C |
| 5 | ADD water | ADD water |
| 6 | EXTRACT with ethyl acetate | EXTRACT with ethyl acetate |
| 7 | COLLECTLAYER organic | COLLECTLAYER organic |
| 8 | DRYSOLUTION over sodium sulfate | WASH with water |
| 9 | CONCENTRATE | WASH with brine |
| 10 | YIELD N1-[4-[3-(4-carbamoylphenyl)-5-trifluoromethyl-4,5-dihydroisoxazol-5-yl]-2-methylphenyl]-3-iodo-N2-isopropylphthalic diamide | DRYSOLUTION over sodium sulfate |
| 11 |  | FILTER keep filtrate |
| 12 |  | CONCENTRATE |
| 13 |  | PURIFY |
| 14 |  | YIELD N1-[4-[3-(4-carbamoylphenyl)-5-trifluoromethyl-4,5-dihydroisoxazol-5-yl]-2-methylphenyl]-3-iodo-N2-isopropylphthalic diamide |

---

```
Reaction no 371
```

Generated by the Chemistry Development Kit (http://github.com/cdk)

|  | A | B |
| --- | --- | --- |
| 0 | ADD 1-{4-[2-(4-chloro-phenylsulfanyl)-phenylamino]-piperidin-1-yl}-ethanone | ADD 1-{4-[2-(4-chloro-phenylsulfanyl)-phenylamino]-piperidin-1-yl}-ethanone |
| 1 | ADD m-chloroperbenzoic acid | ADD m-chloroperbenzoic acid |
| 2 | SETTEMPERATURE 0 °C | ADD 1-{4-[2-(4-chlorobenzenesulfonyl)-phenylamino]-piperidin-1-yl}-ethanone |
| 3 | ADD m-chloroperbenzoic acid | PURIFY |
| 4 | STIR for 3600 s at 0 °C | YIELD 1-{4-[2-(4-chlorobenzenesulfonyl)-phenylamino]-piperidin-1-yl}-ethanone |
| 5 | QUENCH with NaHCO3 |  |
| 6 | COLLECTLAYER aqueous |  |
| 7 | WASH with dichloromethane |  |
| 8 | COLLECTLAYER organic |  |
| 9 | DRYSOLUTION over MgSO4 |  |
| 10 | FILTER keep filtrate |  |
| 11 | CONCENTRATE |  |
| 12 | PURIFY |  |
| 13 | YIELD 1-{4-[2-(4-chlorobenzenesulfonyl)-phenylamino]-piperidin-1-yl}-ethanone |  |

---

```
Reaction no 372
```

Generated by the Chemistry Development Kit (http://github.com/cdk)

|  | A | B |
| --- | --- | --- |
| 0 | ADD 3-cyclopropyl-1-phenylpropan-1-one | ADD 3-cyclopropyl-1-phenylpropan-1-one |
| 1 | ADD THF | ADD THF |
| 2 | ADD (3-(bis(trimethylsilyl)amino)-4-fluorophenyl)magnesium bromide at 0 °C | MAKESOLUTION with (3-(bis(trimethylsilyl)amino)-4-fluorophenyl)magnesium bromide and THF |
| 3 | STIR for 86400 s at 25 °C | ADD SLN at 0 °C |
| 4 | QUENCH with HCl | STIR for 3600 s at 0 °C |
| 5 | STIR for 28800 s | QUENCH with ammonium chloride |
| 6 | ADD NaOH | STIR for 3600 s |
| 7 | EXTRACT with ethyl acetate | EXTRACT with ethyl acetate |
| 8 | COLLECTLAYER organic | COLLECTLAYER organic |
| 9 | WASH with ammonium chloride | WASH with water |
| 10 | DRYSOLUTION over MgSO4 | WASH with brine |
| 11 | FILTER keep filtrate | DRYSOLUTION |
| 12 | CONCENTRATE | FILTER keep filtrate |
| 13 | PURIFY | CONCENTRATE |
| 14 | YIELD 1-(3-amino-4-fluorophenyl)-3-cyclopropyl-1-phenylpropan-1-ol | PURIFY |
| 15 |  | YIELD 1-(3-amino-4-fluorophenyl)-3-cyclopropyl-1-phenylpropan-1-ol |

---

```
Reaction no 373
```

Generated by the Chemistry Development Kit (http://github.com/cdk)

|  | A | B |
| --- | --- | --- |
| 0 | ADD 4-(1-tert-Butoxycarbonyl-5-methoxymethyloxy-indol-3-yl)-2-hydroxy-4-oxo-2-butenoic acid methyl ester | ADD 4-(1-tert-Butoxycarbonyl-5-methoxymethyloxy-indol-3-yl)-2-hydroxy-4-oxo-2-butenoic acid methyl ester |
| 1 | ADD dioxane | ADD dioxane |
| 2 | ADD LiOH | ADD LiOH |
| 3 | STIR for 3600 s at 25 °C | STIR for 86400 s at 25 °C |
| 4 | CONCENTRATE | CONCENTRATE |
| 5 | ADD water | PARTITION with water and ether |
| 6 | COLLECTLAYER aqueous | COLLECTLAYER aqueous |
| 7 | WASH with ethyl acetate | PH with HCl to pH acidic |
| 8 | PH with HCl to pH neutral | EXTRACT with ethyl acetate |
| 9 | EXTRACT with ethyl acetate | COLLECTLAYER organic |
| 10 | COLLECTLAYER organic | WASH with sodium chloride |
| 11 | WASH with water | DRYSOLUTION over magnesium sulfate |
| 12 | WASH with brine | FILTER keep filtrate |
| 13 | DRYSOLUTION | CONCENTRATE |
| 14 | CONCENTRATE | YIELD 4-(1-tert-Butoxycarbonyl-5-methoxymethyloxy-indol-3-yl)-2-hydroxy-4-oxo-2-butenoic acid |
| 15 | WASH with ethyl acetate |  |
| 16 | YIELD 4-(1-tert-Butoxycarbonyl-5-methoxymethyloxy-indol-3-yl)-2-hydroxy-4-oxo-2-butenoic acid |  |

---

```
Reaction no 374
```

Generated by the Chemistry Development Kit (http://github.com/cdk)

|  | A | B |
| --- | --- | --- |
| 0 | ADD 6-(4-chlorobenzoyl)-4-(3-methoxyphenyl)-2(1H)-quinolinone | ADD methyl iodide |
| 1 | ADD THF | MAKESOLUTION with 6-(4-chlorobenzoyl)-4-(3-methoxyphenyl)-2(1H)-quinolinone and benzyltriethylammonium chloride and THF |
| 2 | ADD NaOH | ADD SLN |
| 3 | ADD benzyltriethylammonium chloride | ADD NaOH |
| 4 | ADD methyl iodide | STIR for 3600 s at 25 °C |
| 5 | STIR for 28800 s at 25 °C | ADD water |
| 6 | ADD water | EXTRACT with ethyl acetate |
| 7 | FILTER keep precipitate | COLLECTLAYER organic |
| 8 | DRYSOLID under vacuum | DRYSOLUTION over MgSO4 |
| 9 | YIELD 6-(4-chlorobenzoyl)-4-(3-methoxyphenyl)-1-methyl-2(1H)-quinolinone | FILTER keep filtrate |
| 10 |  | CONCENTRATE |
| 11 |  | PURIFY |
| 12 |  | FILTER keep precipitate |
| 13 |  | CONCENTRATE |
| 14 |  | YIELD 6-(4-chlorobenzoyl)-4-(3-methoxyphenyl)-1-methyl-2(1H)-quinolinone |

---

```
Reaction no 375
```

Generated by the Chemistry Development Kit (http://github.com/cdk)

|  | A | B |
| --- | --- | --- |
| 0 | ADD 4-bromoquinolin-2-amine | ADD 4-bromoquinolin-2-amine |
| 1 | ADD dichloromethane | ADD dichloromethane |
| 2 | ADD triethylamine | SETTEMPERATURE 0 °C |
| 3 | ADD acetyl chloride | ADD acetyl chloride dropwise |
| 4 | STIR for 3600 s at 25 °C | STIR for 3600 s at 25 °C |
| 5 | CONCENTRATE | QUENCH with water |
| 6 | PURIFY | EXTRACT with ethyl acetate |
| 7 | YIELD N-(4-bromoquinolin-2-yl)acetamide | COLLECTLAYER organic |
| 8 |  | WASH with NaHCO3 |
| 9 |  | WASH with brine |
| 10 |  | DRYSOLUTION over Na2SO4 |
| 11 |  | FILTER keep filtrate |
| 12 |  | CONCENTRATE |
| 13 |  | YIELD N-(4-bromoquinolin-2-yl)acetamide |

---

```
Reaction no 376
```

Generated by the Chemistry Development Kit (http://github.com/cdk)

|  | A | B |
| --- | --- | --- |
| 0 | ADD 2-Methyl-4H-thiochromen-4-one | ADD 2-Methyl-4H-thiochromen-4-one |
| 1 | ADD iodine | ADD acetonitrile |
| 2 | ADD ceric ammonium nitrate | ADD iodine |
| 3 | ADD acetonitrile | ADD ceric ammonium nitrate |
| 4 | STIR for 28800 s at 60 °C | STIR for 86400 s at 25 °C |
| 5 | PH with sodium thiosulfate to pH neutral at 0 °C | ADD sodium thiosulfate |
| 6 | EXTRACT with dichloromethane | FILTER keep precipitate |
| 7 | COLLECTLAYER organic | WASH with water |
| 8 | DRYSOLUTION over magnesium sulfate | DRYSOLID under vacuum |
| 9 | CONCENTRATE | YIELD 3-iodo-2-methyl-4H-thiochromen-4-one |
| 10 | PURIFY |  |
| 11 | YIELD 3-iodo-2-methyl-4H-thiochromen-4-one |  |

---

```
Reaction no 377
```

Generated by the Chemistry Development Kit (http://github.com/cdk)

|  | A | B |
| --- | --- | --- |
| 0 | ADD 4,5-dichloro-2-ethyl-2H-pyridazin-3-one | ADD 4,5-dichloro-2-ethyl-2H-pyridazin-3-one |
| 1 | ADD 2-(4-methoxyphenyl)ethylamine | ADD 2-(4-methoxyphenyl)ethylamine |
| 2 | ADD K2CO3 | ADD K2CO3 |
| 3 | ADD dioxane | ADD water |
| 4 | STIR for 86400 s at 100 °C | ADD dioxane |
| 5 | SETTEMPERATURE 25 °C | REFLUX for 28800 s |
| 6 | ADD water | CONCENTRATE |
| 7 | EXTRACT with ethyl acetate | EXTRACT with ethyl acetate |
| 8 | COLLECTLAYER organic | WASH with HCl |
| 9 | DRYSOLUTION over Na2SO4 | WASH with water |
| 10 | FILTER keep filtrate | DRYSOLUTION over sodium sulfate |
| 11 | CONCENTRATE | CONCENTRATE |
| 12 | PURIFY | PURIFY |
| 13 | YIELD 4-Chloro-5-[2-(4-methoxyphenyl)ethylamino]-2-ethyl-3(2H)pyridazinone | YIELD 4-Chloro-5-[2-(4-methoxyphenyl)ethylamino]-2-ethyl-3(2H)pyridazinone |

---

```
Reaction no 378
```

Generated by the Chemistry Development Kit (http://github.com/cdk)

|  | A | B |
| --- | --- | --- |
| 0 | ADD (R)-1-(2-((1-((tert-butyldimethylsilyl)oxy)butan-2-yl)(2-((3-chloro-2-fluorobenzyl)amino)-2-oxoethyl)amino)-2-oxoethyl)-1H-indazole-3-carboxamide | ADD (R)-1-(2-((1-((tert-butyldimethylsilyl)oxy)butan-2-yl)(2-((3-chloro-2-fluorobenzyl)amino)-2-oxoethyl)amino)-2-oxoethyl)-1H-indazole-3-carboxamide |
| 1 | ADD MTBE | ADD HCl |
| 2 | ADD HCl at 25 °C | ADD MTBE |
| 3 | STIR for 3600 s | STIR for 3600 s at 0 °C |
| 4 | CONCENTRATE | CONCENTRATE |
| 5 | PH with NaHCO3 to pH basic | YIELD (R)-1-(2-((2-((3-chloro-2-fluorobenzyl)amino)-2-oxoethyl)(1-hydroxybutan-2-yl)amino)-2-oxoethyl)-1H-indazole-3-carboxamide |
| 6 | EXTRACT with ethyl acetate |  |
| 7 | COLLECTLAYER organic |  |
| 8 | DRYSOLUTION |  |
| 9 | FILTER keep filtrate |  |
| 10 | CONCENTRATE |  |
| 11 | PURIFY |  |
| 12 | YIELD (R)-1-(2-((2-((3-chloro-2-fluorobenzyl)amino)-2-oxoethyl)(1-hydroxybutan-2-yl)amino)-2-oxoethyl)-1H-indazole-3-carboxamide |  |

---

```
Reaction no 379
```

Generated by the Chemistry Development Kit (http://github.com/cdk)

|  | A | B |
| --- | --- | --- |
| 0 | ADD potassium 2-cyclopentyl-1,2,3,4-tetrahydro-isoquinoline-6-carboxylate | ADD potassium 2-cyclopentyl-1,2,3,4-tetrahydro-isoquinoline-6-carboxylate |
| 1 | ADD THF DMF | ADD THF DMF |
| 2 | ADD triethylamine at 0 °C | ADD isobutyl chloroformate |
| 3 | ADD isobutyl chloroformate at 0 °C | STIR for 86400 s at 25 °C |
| 4 | STIR for 86400 s | CONCENTRATE |
| 5 | SETTEMPERATURE 25 °C | PURIFY |
| 6 | CONCENTRATE | YIELD 2-Cyclopentyl-1,2,3,4-tetrahydro-isoquinoline-6-carboxylic acid isobutyric anhydride |
| 7 | ADD brine |  |
| 8 | EXTRACT with dichloromethane |  |
| 9 | YIELD 2-Cyclopentyl-1,2,3,4-tetrahydro-isoquinoline-6-carboxylic acid isobutyric anhydride |  |

---

```
Reaction no 380
```

Generated by the Chemistry Development Kit (http://github.com/cdk)

|  | A | B |
| --- | --- | --- |
| 0 | ADD 4-[4-[(3S)-3-Methylmorpholin-4-yl]-6-(1-pyridin-2-ylsulfonylcyclobutyl)pyrimidin-2-yl]aniline | ADD 4-[4-[(3S)-3-Methylmorpholin-4-yl]-6-(1-pyridin-2-ylsulfonylcyclobutyl)pyrimidin-2-yl]aniline |
| 1 | ADD dioxane | ADD NaHCO3 |
| 2 | ADD NaHCO3 | ADD dioxane |
| 3 | ADD phenyl chloroformate | ADD phenyl chloroformate dropwise |
| 4 | STIR for 3600 s at 25 °C | STIR for 3600 s at 25 °C |
| 5 | CONCENTRATE | CONCENTRATE |
| 6 | ADD dichloromethane | ADD dichloromethane |
| 7 | WASH with water | WASH with water |
| 8 | COLLECTLAYER organic | COLLECTLAYER organic |
| 9 | DRYSOLUTION over MgSO4 | DRYSOLUTION over MgSO4 |
| 10 | FILTER keep filtrate | FILTER keep filtrate |
| 11 | CONCENTRATE | CONCENTRATE |
| 12 | TRITURATE with ether | TRITURATE with ether |
| 13 | YIELD Phenyl N-[4-[4-[(3S)-3-methylmorpholin-4-yl]-6-(1-pyridin-2-ylsulfonylcyclobutyl)pyrimidin-2-yl]phenyl]carbamate | YIELD Phenyl N-[4-[4-[(3S)-3-methylmorpholin-4-yl]-6-(1-pyridin-2-ylsulfonylcyclobutyl)pyrimidin-2-yl]phenyl]carbamate |

---

```
Reaction no 381
```

Generated by the Chemistry Development Kit (http://github.com/cdk)

|  | A | B |
| --- | --- | --- |
| 0 | ADD 2,6-difluorophenol | ADD 2,6-difluorophenol |
| 1 | ADD carbon disulfide | ADD carbon disulfide |
| 2 | MAKESOLUTION with bromine and carbon disulfide | MAKESOLUTION with bromine and carbon disulfide |
| 3 | ADD SLN dropwise at 0 °C | ADD SLN over 600 s |
| 4 | STIR for 86400 s at 25 °C | ADD HBr |
| 5 | ADD sodium metabisulfite | REFLUX for 600 s |
| 6 | ADD HBr | WAIT for 86400 s at 25 °C |
| 7 | EXTRACT with ethyl acetate | WAIT for 86400 s |
| 8 | COLLECTLAYER organic | ADD water |
| 9 | WASH with sodium chloride | ADD sodium metabisulfite |
| 10 | DRYSOLUTION over magnesium sulfate | PHASESEPARATION |
| 11 | CONCENTRATE | COLLECTLAYER organic |
| 12 | PURIFY | WASH with NaHCO3 |
| 13 | YIELD 4-bromo-2,6-difluorophenol | WASH with water |
| 14 |  | COLLECTLAYER organic |
| 15 |  | DRYSOLUTION over Na2SO4 |
| 16 |  | CONCENTRATE |
| 17 |  | PURIFY |
| 18 |  | RECRYSTALLIZE from 4-bromo-2,6-difluorophenol |
| 19 |  | YIELD 4-bromo-2,6-difluorophenol |

---

```
Reaction no 382
```

Generated by the Chemistry Development Kit (http://github.com/cdk)

|  | A | B |
| --- | --- | --- |
| 0 | ADD 3-(4-Chlorophenyl)-3-hydroxy-2-phenethylisoindolin-1-one | ADD 3-(4-Chlorophenyl)-3-hydroxy-2-phenethylisoindolin-1-one |
| 1 | ADD thionyl chloride | ADD thionyl chloride |
| 2 | STIR for 3600 s at 25 °C | ADD DMF |
| 3 | CONCENTRATE | CONCENTRATE |
| 4 | YIELD 3-chloro-3-(4-chlorophenyl)-2-phenethylisoindolin-1-one | YIELD 3-chloro-3-(4-chlorophenyl)-2-phenethylisoindolin-1-one |

---

```
Reaction no 383
```

Generated by the Chemistry Development Kit (http://github.com/cdk)

|  | A | B |
| --- | --- | --- |
| 0 | ADD (S)-(1-Benzylpiperidin-4-yl)methyl 2-hydroxy-2-(3-hydroxyphenyl)-2-phenylacetate | ADD (S)-(1-Benzylpiperidin-4-yl)methyl 2-hydroxy-2-(3-hydroxyphenyl)-2-phenylacetate |
| 1 | ADD dichloromethane | ADD triethylamine |
| 2 | ADD triethylamine | ADD dichloromethane |
| 3 | ADD 2-[N,N-bis(trifluoromethylsulfonyl)amino]-5-chloropyridine | ADD 2-[N,N-bis(trifluoromethylsulfonyl)amino]-5-chloropyridine |
| 4 | STIR for 86400 s at 25 °C | STIR for 86400 s at 25 °C |
| 5 | CONCENTRATE | QUENCH with NaHCO3 |
| 6 | PURIFY | EXTRACT with dichloromethane |
| 7 | YIELD (1-benzylpiperidin-4-yl)methyl ‌(S)-2-hydroxy-2-phenyl-2-(3-(((trifluoromethyl)sulfonyl)oxy)phenyl)acetate | COLLECTLAYER organic |
| 8 |  | CONCENTRATE |
| 9 |  | ADD acetonitrile |
| 10 |  | SETTEMPERATURE 25 °C |
| 11 |  | WASH with acetonitrile |
| 12 |  | WASH with triethylamine |
| 13 |  | COLLECTLAYER organic |
| 14 |  | CONCENTRATE |
| 15 |  | YIELD (1-benzylpiperidin-4-yl)methyl ‌(S)-2-hydroxy-2-phenyl-2-(3-(((trifluoromethyl)sulfonyl)oxy)phenyl)acetate |

---

```
Reaction no 384
```

Generated by the Chemistry Development Kit (http://github.com/cdk)

|  | A | B |
| --- | --- | --- |
| 0 | ADD 4-(2-(4-methylpiperazin-1-yl)ethoxy)benzene-1,2-diamine | ADD 4-(2-(4-methylpiperazin-1-yl)ethoxy)benzene-1,2-diamine |
| 1 | ADD water | ADD acetic acid |
| 2 | ADD acetic acid | MAKESOLUTION with cyanogen bromide and water |
| 3 | STIR for 600 s at 25 °C | ADD SLN dropwise |
| 4 | SETTEMPERATURE 0 °C | STIR for 86400 s at 25 °C |
| 5 | ADD cyanogen bromide | CONCENTRATE |
| 6 | SETTEMPERATURE 25 °C | PURIFY |
| 7 | STIR for 86400 s | YIELD 5-(2-(4-methylpiperazin-1-yl)ethoxy)-1H-benzo[d]imidazol-2-amine |
| 8 | CONCENTRATE |  |
| 9 | ADD acetone |  |
| 10 | DRYSOLUTION over sodium sulfate |  |
| 11 | FILTER keep filtrate |  |
| 12 | CONCENTRATE |  |
| 13 | YIELD 5-(2-(4-methylpiperazin-1-yl)ethoxy)-1H-benzo[d]imidazol-2-amine |  |

---

```
Reaction no 385
```

Generated by the Chemistry Development Kit (http://github.com/cdk)

|  | A | B |
| --- | --- | --- |
| 0 | ADD 2-[(E)-1-(4-methanesulfonyl-phenyl)-3,3-dimethyl-but-1-enyl]-1H-pyrrolo[2,3-b]pyridine | ADD 2-[(E)-1-(4-methanesulfonyl-phenyl)-3,3-dimethyl-but-1-enyl]-1H-pyrrolo[2,3-b]pyridine |
| 1 | ADD Pd/C | ADD Pd/C |
| 2 | ADD methanol | ADD methanol |
| 3 | STIR for 86400 s at 60 °C | STIR for 28800 s at 60 °C |
| 4 | SETTEMPERATURE 25 °C | SETTEMPERATURE 25 °C |
| 5 | FILTER keep precipitate | FILTER keep filtrate |
| 6 | WASH with ethyl acetate | WASH with ethyl acetate |
| 7 | CONCENTRATE | CONCENTRATE |
| 8 | PURIFY | PURIFY |
| 9 | YIELD 2-[1-(4-methanesulfonyl-phenyl)-3,3-dimethyl-butyl]-1H-pyrrolo[2,3-b]pyridine | YIELD 2-[1-(4-methanesulfonyl-phenyl)-3,3-dimethyl-butyl]-1H-pyrrolo[2,3-b]pyridine |

---

```
Reaction no 386
```

Generated by the Chemistry Development Kit (http://github.com/cdk)

|  | A | B |
| --- | --- | --- |
| 0 | ADD (3R\*,4S\*)-3-benzyl-4-[(4-chloro-phenylamino)-methyl]-pyrrolidine-1-carboxylic acid tert-butyl ester | ADD (3R\*,4S\*)-3-benzyl-4-[(4-chloro-phenylamino)-methyl]-pyrrolidine-1-carboxylic acid tert-butyl ester |
| 1 | ADD 2-(chloromethyl)phenyl acetate | ADD DMF |
| 2 | ADD K2CO3 | ADD K2CO3 |
| 3 | ADD Nal | ADD Nal |
| 4 | ADD DMF | ADD 2-(chloromethyl)phenyl acetate |
| 5 | STIR for 28800 s at 100 °C | STIR for 86400 s at 25 °C |
| 6 | ADD NaHCO3 | ADD NaHCO3 |
| 7 | EXTRACT with ethyl acetate | EXTRACT with ethyl acetate |
| 8 | COLLECTLAYER organic | COLLECTLAYER organic |
| 9 | DRYSOLUTION over Na2SO4 | WASH with water |
| 10 | CONCENTRATE | WASH with brine |
| 11 | PURIFY | DRYSOLUTION over MgSO4 |
| 12 | YIELD (3S\*,4R\*)-3-{[(2-Acetoxy-benzyl)-(4-chloro-phenyl)-amino]-methyl}-4-benzyl-pyrrolidine-1-carboxylic acid tert-butyl ester | FILTER keep filtrate |
| 13 |  | CONCENTRATE |
| 14 |  | PURIFY |
| 15 |  | YIELD (3S\*,4R\*)-3-{[(2-Acetoxy-benzyl)-(4-chloro-phenyl)-amino]-methyl}-4-benzyl-pyrrolidine-1-carboxylic acid tert-butyl ester |

---

```
Reaction no 387
```

Generated by the Chemistry Development Kit (http://github.com/cdk)

|  | A | B |
| --- | --- | --- |
| 0 | ADD tert-butyl 4-(1-cyclopropyl-6-fluoro-4-hydroxy-8-methyl-2-oxo-1,2-dihydroquinolin-7-yl)piperazine-1-carboxylate | ADD tert-butyl 4-(1-cyclopropyl-6-fluoro-4-hydroxy-8-methyl-2-oxo-1,2-dihydroquinolin-7-yl)piperazine-1-carboxylate |
| 1 | ADD triethylamine | ADD DMF |
| 2 | ADD DMF | ADD triethylamine |
| 3 | MAKESOLUTION with N-phenylbis(trifluoromethanesulfonimide) and DMF | ADD N-phenylbis(trifluoromethanesulfonimide) |
| 4 | ADD SLN dropwise at 0 °C | STIR for 3600 s at 25 °C |
| 5 | STIR for 3600 s | ADD water |
| 6 | ADD water | EXTRACT with ethyl acetate |
| 7 | EXTRACT with ethyl acetate | COLLECTLAYER organic |
| 8 | WASH with brine | WASH with brine |
| 9 | COLLECTLAYER organic | DRYSOLUTION over sodium sulfate |
| 10 | DRYSOLUTION over MgSO4 | FILTER keep filtrate |
| 11 | FILTER keep filtrate | CONCENTRATE |
| 12 | CONCENTRATE | PURIFY |
| 13 | PURIFY | YIELD tert-butyl 4-(1-cyclopropyl-6-fluoro-8-methyl-2-oxo-4-(((trifluoromethyl)sulfonyl)oxy)-1,2-dihydroquinolin-7-yl)piperazine-1-carboxylate |
| 14 | YIELD tert-butyl 4-(1-cyclopropyl-6-fluoro-8-methyl-2-oxo-4-(((trifluoromethyl)sulfonyl)oxy)-1,2-dihydroquinolin-7-yl)piperazine-1-carboxylate |  |

---

```
Reaction no 388
```

Generated by the Chemistry Development Kit (http://github.com/cdk)

|  | A | B |
| --- | --- | --- |
| 0 | ADD acetic acid | ADD (R)-3-(4-chloro-3-fluorophenyl)-N-(5-(3,5-dimethylisoxazol-4-yl)-2-(((R)-1-(methylsulfonyl) pyrrolidin-3-yl)amino)phenyl)-2-methylpropanamide |
| 1 | ADD (R)-3-(4-chloro-3-fluorophenyl)-N-(5-(3,5-dimethylisoxazol-4-yl)-2-(((R)-1-(methylsulfonyl) pyrrolidin-3-yl)amino)phenyl)-2-methylpropanamide | ADD acetic acid |
| 2 | STIR for 86400 s at 60 °C | STIR for 86400 s at 100 °C |
| 3 | STIR for 604800 s at 100 °C | SETTEMPERATURE 25 °C |
| 4 | CONCENTRATE | CONCENTRATE |
| 5 | TRITURATE with toluene | PURIFY |
| 6 | PURIFY | YIELD 4-(2-((R)-1-(4-chloro-3-fluorophenyl)propan-2-yl)-1-((S)-1-(methylsulfonyl)pyrrolidin-3-yl)-1H-benzo[d]imidazol-5-yl)-3,5-dimethylisoxazole |
| 7 | YIELD 4-(2-((R)-1-(4-chloro-3-fluorophenyl)propan-2-yl)-1-((S)-1-(methylsulfonyl)pyrrolidin-3-yl)-1H-benzo[d]imidazol-5-yl)-3,5-dimethylisoxazole |  |

---

```
Reaction no 389
```

Generated by the Chemistry Development Kit (http://github.com/cdk)

|  | A | B |
| --- | --- | --- |
| 0 | ADD (3R,4S)-2-oxo-4-[3-(trifluoromethyl)phenyl]-3-pyrrolidinecarboxylic acid | ADD (3R,4S)-2-oxo-4-[3-(trifluoromethyl)phenyl]-3-pyrrolidinecarboxylic acid |
| 1 | ADD p-toluenesulfonic acid | ADD toluene |
| 2 | ADD toluene | ADD p-toluenesulfonic acid |
| 3 | STIR for 86400 s at 100 °C | REFLUX for 86400 s with Dean-Stark apparatus |
| 4 | CONCENTRATE | CONCENTRATE |
| 5 | YIELD (4S)-4-[3-(trifluoromethyl)phenyl]-2-pyrrolidinone | PURIFY |
| 6 |  | YIELD (4S)-4-[3-(trifluoromethyl)phenyl]-2-pyrrolidinone |

---

```
Reaction no 390
```

Generated by the Chemistry Development Kit (http://github.com/cdk)

|  | A | B |
| --- | --- | --- |
| 0 | ADD 1-bromo-4-methylnaphthalene | ADD benzoyl peroxide |
| 1 | ADD N-bromosuccinimide | ADD trifluoromethylbenzene |
| 2 | ADD benzoyl peroxide | REFLUX for 3600 s |
| 3 | ADD trifluoromethylbenzene | SETTEMPERATURE 60 °C |
| 4 | REFLUX for 28800 s | ADD 1-bromo-4-methylnaphthalene |
| 5 | SETTEMPERATURE 25 °C | ADD N-bromosuccinimide |
| 6 | FILTER keep filtrate | STIR for 3600 s at 60 °C |
| 7 | CONCENTRATE | FILTER keep filtrate |
| 8 | PURIFY | CONCENTRATE |
| 9 | YIELD 1-bromo-4-bromomethyl-naphthalene | RECRYSTALLIZE from heptane |
| 10 |  | YIELD 1-bromo-4-bromomethyl-naphthalene |

---

```
Reaction no 391
```

Generated by the Chemistry Development Kit (http://github.com/cdk)

|  | A | B |
| --- | --- | --- |
| 0 | ADD 2-(5-((3,5-dimethylisoxazol-4-yl)methoxy)pyridin-2-yl)acetic acid | ADD 2-(5-((3,5-dimethylisoxazol-4-yl)methoxy)pyridin-2-yl)acetic acid |
| 1 | ADD THF | ADD THF |
| 2 | ADD 1,2-dichloroethane | ADD 1,2-dichloroethane |
| 3 | ADD HOBt | STIR for 3600 s at 25 °C |
| 4 | ADD triethylamine | ADD rac-4-chlorobenzhydrylamine hydrochloride |
| 5 | STIR for 3600 s at 25 °C | ADD HOBt |
| 6 | ADD rac-4-chlorobenzhydrylamine hydrochloride | ADD triethylamine |
| 7 | STIR for 86400 s at 25 °C | STIR for 86400 s at 25 °C |
| 8 | ADD water | ADD water |
| 9 | EXTRACT with ethyl acetate | EXTRACT with ethyl acetate |
| 10 | COLLECTLAYER organic | COLLECTLAYER organic |
| 11 | DRYSOLUTION over Na2SO4 | DRYSOLUTION over Na2SO4 |
| 12 | FILTER keep filtrate | CONCENTRATE |
| 13 | CONCENTRATE | PURIFY |
| 14 | PURIFY | YIELD N-((4-chlorophenyl)(phenyl)methyl)-2-(5-((3,5-dimethylisoxazol-4-yl)methoxy)pyridin-2-yl)acetamide |
| 15 | YIELD N-((4-chlorophenyl)(phenyl)methyl)-2-(5-((3,5-dimethylisoxazol-4-yl)methoxy)pyridin-2-yl)acetamide |  |

---

```
Reaction no 392
```

Generated by the Chemistry Development Kit (http://github.com/cdk)

|  | A | B |
| --- | --- | --- |
| 0 | ADD 1-tert-butyl 2-methyl pyrrolidine-1,2-dicarboxylate | ADD 1-tert-butyl 2-methyl pyrrolidine-1,2-dicarboxylate |
| 1 | ADD THF | ADD THF |
| 2 | ADD LiHMDS at -70 °C | ADD LiHMDS at -30 °C |
| 3 | STIR for 3600 s at -70 °C | STIR for 3600 s |
| 4 | MAKESOLUTION with 3,5-dibromobenzyl bromide and THF | ADD 3,5-dibromobenzyl bromide dropwise at -30 °C |
| 5 | ADD SLN | STIR for 3600 s at 25 °C |
| 6 | STIR for 86400 s at 25 °C | QUENCH with ammonium chloride at 0 °C |
| 7 | QUENCH with ammonium chloride | COLLECTLAYER organic |
| 8 | EXTRACT with ethyl acetate | WASH with water |
| 9 | CONCENTRATE | EXTRACT with ethyl acetate |
| 10 | PURIFY | COLLECTLAYER organic |
| 11 | YIELD 1-tert-butyl 2-methyl 2-(3,5-dibromobenzyl)pyrrolidine-1,2-dicarboxylate | DRYSOLUTION over Na2SO4 |
| 12 |  | CONCENTRATE |
| 13 |  | PURIFY |
| 14 |  | YIELD 1-tert-butyl 2-methyl 2-(3,5-dibromobenzyl)pyrrolidine-1,2-dicarboxylate |

---

```
Reaction no 393
```

Generated by the Chemistry Development Kit (http://github.com/cdk)

|  | A | B |
| --- | --- | --- |
| 0 | ADD (4-chloro-5,6,7,8-tetrahydroquinazolin-7-yl)methanol | ADD (4-chloro-5,6,7,8-tetrahydroquinazolin-7-yl)methanol |
| 1 | ADD THF | ADD K2CO3 |
| 2 | ADD K2CO3 | ADD THF |
| 3 | ADD methyl iodide | ADD methyl iodide |
| 4 | STIR for 86400 s at 25 °C | STIR for 86400 s |
| 5 | FILTER keep filtrate | PARTITION with ethyl acetate and water |
| 6 | CONCENTRATE | COLLECTLAYER organic |
| 7 | PURIFY | WASH with brine |
| 8 | YIELD 4-chloro-7-(methoxymethyl)-5,6,7,8-tetrahydroquinazoline | DRYSOLUTION over magnesium sulfate |
| 9 |  | FILTER keep filtrate |
| 10 |  | CONCENTRATE |
| 11 |  | PURIFY |
| 12 |  | YIELD 4-chloro-7-(methoxymethyl)-5,6,7,8-tetrahydroquinazoline |

---

```
Reaction no 394
```

Generated by the Chemistry Development Kit (http://github.com/cdk)

|  | A | B |
| --- | --- | --- |
| 0 | ADD ethanol | ADD 4-nitrophenylalanine |
| 1 | ADD thionyl chloride at 0 °C over 600 s | ADD ethanol |
| 2 | STIR for 600 s at 0 °C | ADD thionyl chloride dropwise at 0 °C |
| 3 | STIR for 3600 s at 25 °C | REFLUX for 86400 s |
| 4 | ADD 4-nitrophenylalanine | CONCENTRATE |
| 5 | REFLUX for 86400 s | YIELD 4-nitrophenylalanine ethyl ester hydrochloride |
| 6 | CONCENTRATE |  |
| 7 | YIELD 4-nitrophenylalanine ethyl ester hydrochloride |  |

---

```
Reaction no 395
```

Generated by the Chemistry Development Kit (http://github.com/cdk)

|  | A | B |
| --- | --- | --- |
| 0 | ADD 1-(1-benzyl-4-piperidinyl)-3-bromo-4-chloro-1H-pyrazolo[3,4-d]pyrimidine | ADD 1-(1-benzyl-4-piperidinyl)-3-bromo-4-chloro-1H-pyrazolo[3,4-d]pyrimidine |
| 1 | ADD dioxane | ADD ammonium hydroxide |
| 2 | ADD ammonium hydroxide | ADD dioxane |
| 3 | STIR for 86400 s at 100 °C | STIR for 86400 s at 100 °C |
| 4 | CONCENTRATE | CONCENTRATE |
| 5 | PURIFY | YIELD 1-(1-benzyl-4-piperidinyl)-3-bromo-1H-pyrazolo[3,4-d]pyrimidin-4-amine |
| 6 | YIELD 1-(1-benzyl-4-piperidinyl)-3-bromo-1H-pyrazolo[3,4-d]pyrimidin-4-amine |  |

---

```
Reaction no 396
```

Generated by the Chemistry Development Kit (http://github.com/cdk)

|  | A | B |
| --- | --- | --- |
| 0 | ADD 3-(difluoromethyl)-5-nitro-1H-indazole | ADD 3-(difluoromethyl)-5-nitro-1H-indazole |
| 1 | ADD trityl chloride | ADD acetonitrile |
| 2 | ADD K2CO3 | ADD K2CO3 |
| 3 | ADD acetonitrile | ADD trityl chloride |
| 4 | STIR for 86400 s at 60 °C | STIR for 28800 s at 25 °C |
| 5 | ADD water | ADD water |
| 6 | EXTRACT with ethyl acetate | EXTRACT with ethyl acetate |
| 7 | WASH with brine | COLLECTLAYER organic |
| 8 | DRYSOLUTION over sodium sulfate | DRYSOLUTION over Na2SO4 |
| 9 | CONCENTRATE | CONCENTRATE |
| 10 | PURIFY | PURIFY |
| 11 | YIELD 3-(difluoromethyl)-5-nitro-1-trityl-1H-indazole | YIELD 3-(difluoromethyl)-5-nitro-1-trityl-1H-indazole |

---

```
Reaction no 397
```

Generated by the Chemistry Development Kit (http://github.com/cdk)

|  | A | B |
| --- | --- | --- |
| 0 | ADD (1,4-diazepan-5-yl)methanol | ADD (1,4-diazepan-5-yl)methanol |
| 1 | ADD dioxane | ADD dioxane |
| 2 | ADD NaOH | ADD NaOH |
| 3 | MAKESOLUTION with di-tert-butyl dicarbonate and dioxane | ADD di-tert-butyl dicarbonate at 0 °C |
| 4 | ADD SLN dropwise at 0 °C | STIR for 86400 s at 25 °C |
| 5 | STIR for 3600 s at 0 °C | CONCENTRATE |
| 6 | CONCENTRATE | ADD water |
| 7 | EXTRACT with dichloromethane | EXTRACT with dichloromethane |
| 8 | EXTRACT with isopropanol / chloroform | COLLECTLAYER organic |
| 9 | COLLECTLAYER organic | DRYSOLUTION over Na2SO4 |
| 10 | DRYSOLUTION over Na2SO4 | FILTER keep filtrate |
| 11 | CONCENTRATE | CONCENTRATE |
| 12 | YIELD tert-butyl 5-(hydroxymethyl)-1,4-diazepane-1-carboxylate | YIELD tert-butyl 5-(hydroxymethyl)-1,4-diazepane-1-carboxylate |

---

```
Reaction no 398
```

Generated by the Chemistry Development Kit (http://github.com/cdk)

|  | A | B |
| --- | --- | --- |
| 0 | ADD 3-bromoaniline | ADD 3-bromoaniline at 0 °C |
| 1 | ADD HCl | ADD HCl at 0 °C |
| 2 | ADD water | ADD NaNO2 at 0 °C |
| 3 | SETTEMPERATURE 0 °C | ADD water at 0 °C |
| 4 | MAKESOLUTION with NaNO2 and water | STIR for 3600 s at 0 °C |
| 5 | ADD SLN dropwise | MAKESOLUTION with sodium acetate and tert-butyl acetoacetate and ethanol and water |
| 6 | STIR for 600 s at 0 °C | ADD SLN |
| 7 | MAKESOLUTION with tert-butyl acetoacetate and ethanol and sodium acetate and water | STIR for 86400 s at 25 °C |
| 8 | ADD SLN | FILTER keep precipitate |
| 9 | MAKESOLUTION with tert-butyl acetoacetate and ethanol and sodium acetate and water | WASH with water |
| 10 | ADD SLN at 0 °C over 600 s | WASH with $3$ / $6$ |
| 11 | STIR for 3600 s | WASH with hexane |
| 12 | ADD NaHCO3 | DRYSOLID |
| 13 | WASH with water | YIELD tert-Butyl ‌(2E)-2-[2-(3-bromophenyl)hydrazinylidene]-3-oxobutanoate |
| 14 | COLLECTLAYER aqueous |  |
| 15 | EXTRACT with ethyl acetate |  |
| 16 | COLLECTLAYER organic |  |
| 17 | WASH with brine |  |
| 18 | DRYSOLUTION over sodium sulfate |  |
| 19 | FILTER keep filtrate |  |
| 20 | YIELD tert-Butyl ‌(2E)-2-[2-(3-bromophenyl)hydrazinylidene]-3-oxobutanoate |  |

---

```
Reaction no 399
```

Generated by the Chemistry Development Kit (http://github.com/cdk)

|  | A | B |
| --- | --- | --- |
| 0 | ADD 1-(3-phenyl-isoxazol-4-yl)-ethanone | ADD 1-(3-phenyl-isoxazol-4-yl)-ethanone |
| 1 | ADD formamide | ADD formamide |
| 2 | ADD water | STIR for 86400 s at 100 °C |
| 3 | MICROWAVE for 3600 s at 100 °C | SETTEMPERATURE 25 °C |
| 4 | ADD HCl | ADD water |
| 5 | EXTRACT with ethyl acetate | PH with HCl to pH acidic |
| 6 | COLLECTLAYER aqueous | EXTRACT with ethyl acetate |
| 7 | PH with sodium hydroxide to pH basic | COLLECTLAYER organic |
| 8 | EXTRACT with ethyl acetate | WASH with water |
| 9 | COLLECTLAYER organic | WASH with brine |
| 10 | DRYSOLUTION over Na2SO4 | DRYSOLUTION over Na2SO4 |
| 11 | CONCENTRATE | CONCENTRATE |
| 12 | PURIFY | PURIFY |
| 13 | YIELD 4-(1H-Imidazol-4-yl)-3-phenyl-isoxazole | YIELD 4-(1H-Imidazol-4-yl)-3-phenyl-isoxazole |

---

```
Reaction no 400
```

Generated by the Chemistry Development Kit (http://github.com/cdk)

|  | A | B |
| --- | --- | --- |
| 0 | ADD benzoyl chloride | ADD 2-amino-4-methoxybenzoic acid |
| 1 | ADD pyridine | ADD pyridine |
| 2 | MAKESOLUTION with 2-amino-4-methoxybenzoic acid and pyridine | ADD benzoyl chloride at 0 °C |
| 3 | ADD SLN dropwise at 25 °C | STIR for 3600 s at 25 °C |
| 4 | STIR for 28800 s at 25 °C | ADD ice water |
| 5 | ADD ice water | FILTER keep precipitate |
| 6 | EXTRACT with ethyl acetate | WASH with water |
| 7 | DRYSOLUTION over Na2SO4 | DRYSOLID |
| 8 | FILTER keep filtrate | YIELD 7-methoxy-2-phenyl -4H-benzo[d][1,3] oxazin -4-one |
| 9 | CONCENTRATE |  |
| 10 | PURIFY |  |
| 11 | YIELD 7-methoxy-2-phenyl -4H-benzo[d][1,3] oxazin -4-one |  |

---

```
Reaction no 401
```

Generated by the Chemistry Development Kit (http://github.com/cdk)

|  | A | B |
| --- | --- | --- |
| 0 | ADD tert-butyl [4-chloro-2-(trifluoromethyl)phenyl]acetate | ADD tert-butyl [4-chloro-2-(trifluoromethyl)phenyl]acetate |
| 1 | ADD DMF | ADD DMF |
| 2 | ADD NaH at 0 °C | ADD NaH at 0 °C |
| 3 | STIR for 3600 s at 25 °C | STIR for 3600 s at 25 °C |
| 4 | ADD 1-bromo-3-chloropropane | ADD 1-bromo-3-chloropropane at 0 °C |
| 5 | STIR for 3600 s at 25 °C | STIR for 86400 s at 25 °C |
| 6 | ADD water | ADD water |
| 7 | EXTRACT with ethyl acetate | EXTRACT with ethyl acetate |
| 8 | COLLECTLAYER organic | WASH with water |
| 9 | WASH with brine | WASH with brine |
| 10 | DRYSOLUTION over MgSO4 | DRYSOLUTION over magnesium sulfate |
| 11 | FILTER keep filtrate | CONCENTRATE |
| 12 | CONCENTRATE | PURIFY |
| 13 | PURIFY | YIELD tert-butyl 5-chloro-2-[4-chloro-2-(trifluoromethyl)phenyl]pentanoate |
| 14 | YIELD tert-butyl 5-chloro-2-[4-chloro-2-(trifluoromethyl)phenyl]pentanoate |  |

---

```
Reaction no 402
```

Generated by the Chemistry Development Kit (http://github.com/cdk)

|  | A | B |
| --- | --- | --- |
| 0 | ADD Benzyl 3,6,9,12-Tetraoxatridecanoate | ADD Benzyl 3,6,9,12-Tetraoxatridecanoate |
| 1 | ADD methanol | ADD methanol |
| 2 | ADD Pd/C | ADD Pd/C |
| 3 | STIR for 86400 s at 25 °C | FILTER keep filtrate |
| 4 | FILTER keep filtrate | WASH with 3,6,9,12-Tetraoxatridecanoic acid |
| 5 | CONCENTRATE | CONCENTRATE |
| 6 | YIELD 3,6,9,12-Tetraoxatridecanoic acid |  |

---

```
Reaction no 403
```

Generated by the Chemistry Development Kit (http://github.com/cdk)

|  | A | B |
| --- | --- | --- |
| 0 | ADD N-[1-Amino-2,3-dihydro-1H-inden-2-yl]-5-chloro-1H-indole-2-carboxamide trifluoroacetic acid salt | ADD N-[1-Amino-2,3-dihydro-1H-inden-2-yl]-5-chloro-1H-indole-2-carboxamide trifluoroacetic acid salt at 25 °C |
| 1 | ADD dichloromethane | ADD triethylamine at 25 °C |
| 2 | ADD triethylamine at 0 °C | ADD dichloromethane at 25 °C |
| 3 | ADD chloroacetyl chloride at 0 °C | MAKESOLUTION with chloroacetyl chloride and dichloromethane |
| 4 | STIR for 3600 s at 25 °C | ADD SLN |
| 5 | ADD dichloromethane | SETTEMPERATURE 25 °C |
| 6 | WASH with NaHCO3 | STIR for 3600 s |
| 7 | WASH with water | FILTER keep filtrate |
| 8 | WASH with brine | WASH with 5-Chloro-N-((1R,2R)-1-{[(2R)-2,3-dihydroxypropyl]amino}-2,3-dihydro-1H-inden-2-yl)-1H-indole-2-carboxamide |
| 9 | DRYSOLUTION over Na2SO4 |  |
| 10 | CONCENTRATE |  |
| 11 | PURIFY |  |
| 12 | YIELD 5-Chloro-N-((1R,2R)-1-{[(2R)-2,3-dihydroxypropyl]amino}-2,3-dihydro-1H-inden-2-yl)-1H-indole-2-carboxamide |  |

---

```
Reaction no 404
```

Generated by the Chemistry Development Kit (http://github.com/cdk)

|  | A | B |
| --- | --- | --- |
| 0 | ADD N-(4-fluorophenyl)-N-methyl-2-[(3R)-3-methylmorpholin-4-yl]-8-[1-(tetrahydro-2H-pyran-2-yl)-1H-pyrazol-5-yl]-1,7-naphthyridin-4-amine | ADD N-(4-fluorophenyl)-N-methyl-2-[(3R)-3-methylmorpholin-4-yl]-8-[1-(tetrahydro-2H-pyran-2-yl)-1H-pyrazol-5-yl]-1,7-naphthyridin-4-amine |
| 1 | ADD methanol | ADD methanol |
| 2 | ADD HCl | ADD HCl |
| 3 | STIR for 3600 s at 25 °C | STIR for 3600 s at 25 °C |
| 4 | ADD NaHCO3 | ADD NaHCO3 |
| 5 | EXTRACT with ethyl acetate / THF | EXTRACT with ethyl acetate |
| 6 | FILTER keep filtrate | FILTER keep filtrate |
| 7 | CONCENTRATE | CONCENTRATE |
| 8 | PURIFY | PURIFY |
| 9 | YIELD N-(4-fluorophenyl)-N-methyl-2-[(3R)-3-methylmorpholin-4-yl]-8-(1H-pyrazol-5-yl)-1,7-naphthyridin-4-amine | YIELD N-(4-fluorophenyl)-N-methyl-2-[(3R)-3-methylmorpholin-4-yl]-8-(1H-pyrazol-5-yl)-1,7-naphthyridin-4-amine |

---

```
Reaction no 405
```

Generated by the Chemistry Development Kit (http://github.com/cdk)

|  | A | B |
| --- | --- | --- |
| 0 | ADD tert-butyl N-(2-bromo-4-(5-(3,5-dichlorophenyl)-5-trifluoromethyl-4,5-dihydroisoxazole-3-yl) phenylmethyl)-N-(cyclopropylcarbonyl) carbamate | ADD tert-butyl N-(2-bromo-4-(5-(3,5-dichlorophenyl)-5-trifluoromethyl-4,5-dihydroisoxazole-3-yl) phenylmethyl)-N-(cyclopropylcarbonyl) carbamate |
| 1 | ADD THF | ADD THF |
| 2 | ADD lithium hexamethyldisilazane tetrahydrofuran at 25 °C | ADD lithium hexamethyldisilazane tetrahydrofuran at -70 °C |
| 3 | STIR for 3600 s at 25 °C | STIR for 3600 s at -70 °C |
| 4 | ADD ice water | ADD HCl |
| 5 | ADD HCl | EXTRACT with ethyl acetate |
| 6 | EXTRACT with ethyl acetate | WASH with brine |
| 7 | COLLECTLAYER organic | DRYSOLUTION over sodium sulfate |
| 8 | WASH with water | CONCENTRATE |
| 9 | DRYSOLID under vacuum | PURIFY |
| 10 | CONCENTRATE | YIELD tert-butyl N-(2-bromo-4-((2Z)-3-(3,5-dichlorophenyl)-1-hydroxyimino-4,4,4-trifluoro-2-butenyl) phenylmethyl)-N-(cyclopropylcarbonyl) carbamate |
| 11 | PURIFY |  |
| 12 | YIELD tert-butyl N-(2-bromo-4-((2Z)-3-(3,5-dichlorophenyl)-1-hydroxyimino-4,4,4-trifluoro-2-butenyl) phenylmethyl)-N-(cyclopropylcarbonyl) carbamate |  |

---

```
Reaction no 406
```

Generated by the Chemistry Development Kit (http://github.com/cdk)

|  | A | B |
| --- | --- | --- |
| 0 | ADD 4-Acetamido-3-nitrobenzaldehyde | ADD 4-Acetamido-3-nitrobenzaldehyde |
| 1 | ADD (carbethoxyethylidene)triphenylphosphorane | ADD dichloromethane |
| 2 | ADD dichloromethane | MAKESOLUTION with (carbethoxyethylidene)triphenylphosphorane and dichloromethane |
| 3 | STIR for 86400 s at 25 °C | ADD SLN dropwise at 25 °C |
| 4 | CONCENTRATE | STIR for 86400 s at 25 °C |
| 5 | PURIFY | CONCENTRATE |
| 6 | YIELD (E)-3-(4-Acetylamino-3-nitro-phenyl)-2-methyl-acrylic acid ethyl ester | PURIFY |
| 7 |  | YIELD (E)-3-(4-Acetylamino-3-nitro-phenyl)-2-methyl-acrylic acid ethyl ester |

---

```
Reaction no 407
```

Generated by the Chemistry Development Kit (http://github.com/cdk)

|  | A | B |
| --- | --- | --- |
| 0 | ADD crizotinib | ADD 3-(7-((8-iodooctyl)oxy)-4-methyl-2-oxoquinolin-1(2H)-yl)piperidine-2,6-dione |
| 1 | ADD 3-(7-((8-iodooctyl)oxy)-4-methyl-2-oxoquinolin-1(2H)-yl)piperidine-2,6-dione | ADD crizotinib |
| 2 | ADD DMF | ADD DMF |
| 3 | ADD DIPEA | ADD DIPEA |
| 4 | STIR for 86400 s at 25 °C | STIR for 86400 s at 60 °C |
| 5 | PURIFY | SETTEMPERATURE 25 °C |
| 6 | YIELD 3-(7-((8-(4-(4-(6-amino-5-((R)-1-(2,6-dichloro-3-fluorophenyl)ethoxy)pyridin-3-yl)-1H-pyrazol-1-yl)piperidin-1-yl)octyl)oxy)-4-methyl-2-oxoquinolin-1(2H)-yl)piperidine-2,6-dione | CONCENTRATE |
| 7 |  | PURIFY |
| 8 |  | YIELD 3-(7-((8-(4-(4-(6-amino-5-((R)-1-(2,6-dichloro-3-fluorophenyl)ethoxy)pyridin-3-yl)-1H-pyrazol-1-yl)piperidin-1-yl)octyl)oxy)-4-methyl-2-oxoquinolin-1(2H)-yl)piperidine-2,6-dione |

---

```
Reaction no 408
```

Generated by the Chemistry Development Kit (http://github.com/cdk)

|  | A | B |
| --- | --- | --- |
| 0 | ADD 3-bromo-4-hydroxy-6-phenylindeno[1,2-b]pyrano[2,3-d]pyridine-2,5(6H,11H)-dione | ADD 3-bromo-4-hydroxy-6-phenylindeno[1,2-b]pyrano[2,3-d]pyridine-2,5(6H,11H)-dione |
| 1 | ADD thiophenol | ADD thiophenol |
| 2 | ADD K2CO3 | ADD K2CO3 |
| 3 | ADD DMF | ADD DMF |
| 4 | STIR for 86400 s at 60 °C | STIR for 3600 s at 100 °C |
| 5 | SETTEMPERATURE 25 °C | YIELD 4-Hydroxy-6-phenyl-3-(phenylthio)indeno[1,2-b]pyrano[2,3-d]pyridine-2,5(6H,11H)-dione |
| 6 | PARTITION with HCl and ether |  |
| 7 | FILTER keep precipitate |  |
| 8 | WASH with ether |  |
| 9 | DRYSOLID under vacuum |  |
| 10 | YIELD 4-Hydroxy-6-phenyl-3-(phenylthio)indeno[1,2-b]pyrano[2,3-d]pyridine-2,5(6H,11H)-dione |  |

---

```
Reaction no 409
```

Generated by the Chemistry Development Kit (http://github.com/cdk)

|  | A | B |
| --- | --- | --- |
| 0 | ADD (3R\*,4S\*)-N-[3,5-bis(trifluoromethyl)benzyl]-N-methyl-3-phenylpiperidine-4-carboxamide hydrochloride | ADD (3R\*,4S\*)-N-[3,5-bis(trifluoromethyl)benzyl]-N-methyl-3-phenylpiperidine-4-carboxamide hydrochloride |
| 1 | ADD triethylamine | ADD triethylamine |
| 2 | ADD THF | ADD THF |
| 3 | ADD acetyl chloride | ADD acetyl chloride at 0 °C |
| 4 | STIR for 86400 s at 25 °C | STIR for 3600 s at 25 °C |
| 5 | ADD water | ADD water |
| 6 | EXTRACT with ethyl acetate | EXTRACT with ethyl acetate |
| 7 | COLLECTLAYER organic | WASH with water |
| 8 | WASH with citric acid | WASH with brine |
| 9 | WASH with brine | DRYSOLUTION |
| 10 | DRYSOLUTION | CONCENTRATE |
| 11 | CONCENTRATE | PURIFY |
| 12 | PURIFY | YIELD (3R\*,4S\*)-1-acetyl-N-[3,5-bis(trifluoromethyl)benzyl]-N-methyl-3-phenylpiperidine-4-carboxamide |
| 13 | YIELD (3R\*,4S\*)-1-acetyl-N-[3,5-bis(trifluoromethyl)benzyl]-N-methyl-3-phenylpiperidine-4-carboxamide |  |

---

```
Reaction no 410
```

Generated by the Chemistry Development Kit (http://github.com/cdk)

|  | A | B |
| --- | --- | --- |
| 0 | ADD 5-hydroxy-2,3-dihydrobenzothiophene | ADD 5-hydroxy-2,3-dihydrobenzothiophene |
| 1 | ADD acetone | ADD acetone |
| 2 | ADD K2CO3 | ADD K2CO3 |
| 3 | ADD allyl bromide | SETTEMPERATURE 25 °C |
| 4 | STIR for 86400 s at 25 °C | ADD allyl bromide |
| 5 | FILTER keep filtrate | REFLUX for 86400 s |
| 6 | CONCENTRATE | SETTEMPERATURE 25 °C |
| 7 | YIELD 5-Allyloxy-2,3-dihydrobenzothiophene | FILTER keep filtrate |
| 8 |  | CONCENTRATE |
| 9 |  | ADD hexane / dichloromethane |
| 10 |  | FILTER keep filtrate |
| 11 |  | PURIFY |
| 12 |  | YIELD 5-Allyloxy-2,3-dihydrobenzothiophene |

---

```
Reaction no 411
```

Generated by the Chemistry Development Kit (http://github.com/cdk)

|  | A | B |
| --- | --- | --- |
| 0 | ADD 3-(3-chloropropyl)-6-fluoro-1,2-benzisoxazole | ADD DMF |
| 1 | ADD 1-methylpiperazine | ADD 1-methylpiperazine |
| 2 | ADD NaHCO3 | ADD 3-(3-chloropropyl)-6-fluoro-1,2-benzisoxazole |
| 3 | ADD Kaliumiodid | ADD NaHCO3 |
| 4 | ADD DMF | ADD Kaliumiodid |
| 5 | STIR for 3600 s at 100 °C | STIR for 3600 s at 100 °C |
| 6 | FILTER keep filtrate | FILTER keep filtrate |
| 7 | CONCENTRATE | CONCENTRATE |
| 8 | ADD water | ADD water |
| 9 | EXTRACT with ether | STIR for 600 s |
| 10 | WASH with water | EXTRACT with ether |
| 11 | WASH with sodium chloride | WASH with water |
| 12 | DRYSOLUTION over magnesium sulfate | WASH with sodium chloride |
| 13 | FILTER keep filtrate | DRYSOLUTION over magnesium sulfate |
| 14 | CONCENTRATE | FILTER keep filtrate |
| 15 | ADD ether / HCl | CONCENTRATE |
| 16 | YIELD CN1CCN(CCCc2noc3cc(F)ccc23)CC1.Cl.Cl | ADD ether |
| 17 |  | PH with ether / HCl to pH acidic |
| 18 |  | RECRYSTALLIZE from ethyl acetate / methanol / ether |
| 19 |  | YIELD CN1CCN(CCCc2noc3cc(F)ccc23)CC1.Cl.Cl |
| 20 |  | RECRYSTALLIZE from ethyl acetate / methanol / ether |

---

```
Reaction no 412
```

Generated by the Chemistry Development Kit (http://github.com/cdk)

|  | A | B |
| --- | --- | --- |
| 0 | ADD 5-(trifluoromethyl)pyridin-2-amine | ADD 5-(trifluoromethyl)pyridin-2-amine |
| 1 | ADD dichloromethane | ADD dichloromethane |
| 2 | ADD pyridine | ADD pyridine |
| 3 | ADD 4-nitrophenyl chloroformate | ADD 4-nitrophenyl chloroformate at 0 °C |
| 4 | STIR for 3600 s at 25 °C | STIR for 3600 s at 25 °C |
| 5 | FILTER keep precipitate | FILTER keep precipitate |
| 6 | DRYSOLID under vacuum | WASH with dichloromethane |
| 7 | YIELD (4-Nitrophenyl) N-[4-(trifluoromethyl)phenyl]carbamate | DRYSOLID under vacuum |
| 8 |  | YIELD (4-Nitrophenyl) N-[4-(trifluoromethyl)phenyl]carbamate |

---

```
Reaction no 413
```

Generated by the Chemistry Development Kit (http://github.com/cdk)

|  | A | B |
| --- | --- | --- |
| 0 | ADD 1,4-dioxaspiro[4.5]decan-8-one | ADD 1,4-dioxaspiro[4.5]decan-8-one |
| 1 | ADD methanol | ADD methanol |
| 2 | SETTEMPERATURE 0 °C | ADD sodium borohydride at 0 °C |
| 3 | ADD sodium borohydride | STIR for 3600 s at 0 °C |
| 4 | STIR for 3600 s at 25 °C | ADD ammonium chloride |
| 5 | ADD ammonium chloride | EXTRACT with ethyl acetate |
| 6 | COLLECTLAYER aqueous | COLLECTLAYER organic |
| 7 | EXTRACT with dichloromethane | WASH with brine |
| 8 | COLLECTLAYER organic | DRYSOLUTION over sodium sulfate |
| 9 | FILTER keep filtrate | CONCENTRATE |
| 10 | CONCENTRATE | YIELD 1,4-dioxaspiro[4.5]decan-8-ol |
| 11 | YIELD 1,4-dioxaspiro[4.5]decan-8-ol |  |

---

```
Reaction no 414
```

Generated by the Chemistry Development Kit (http://github.com/cdk)

|  | A | B |
| --- | --- | --- |
| 0 | ADD 5-(4-chlorobenzenesulfonamido)-2-(3chloro-5-pyridyloxy)benzoic acid | ADD 5-(4-chlorobenzenesulfonamido)-2-(3chloro-5-pyridyloxy)benzoic acid |
| 1 | ADD dichloromethane | ADD dichloromethane |
| 2 | ADD pyridine | ADD pyridine |
| 3 | ADD thionyl chloride | ADD thionyl chloride |
| 4 | STIR for 3600 s at 25 °C | WAIT for 3600 s |
| 5 | ADD ammonia | CONCENTRATE |
| 6 | STIR for 86400 s at 25 °C | DRYSOLID for 86400 s under vacuum |
| 7 | CONCENTRATE | ADD methanol |
| 8 | PURIFY | ADD dichloromethane |
| 9 | YIELD 5-(4-chlorobenzenesulfonamido)-2-(3-chloro-5-pyridyloxy)benzamide | ADD ammonia dropwise |
| 10 |  | ADD ethanol |
| 11 |  | STIR for 28800 s |
| 12 |  | CONCENTRATE |
| 13 |  | PURIFY |
| 14 |  | YIELD 5-(4-chlorobenzenesulfonamido)-2-(3-chloro-5-pyridyloxy)benzamide |

---

```
Reaction no 415
```

Generated by the Chemistry Development Kit (http://github.com/cdk)

|  | A | B |
| --- | --- | --- |
| 0 | ADD 3-phenyl-1H-pyrazol-5-amine | ADD ethyl 4,4-difluoro-3-oxobutanoate |
| 1 | ADD ethyl 4,4-difluoro-3-oxobutanoate | ADD acetic acid water |
| 2 | ADD acetic acid water | ADD 3-phenyl-1H-pyrazol-5-amine |
| 3 | STIR for 86400 s at 100 °C | STIR for 86400 s at 100 °C |
| 4 | SETTEMPERATURE 25 °C | SETTEMPERATURE 25 °C |
| 5 | CONCENTRATE | FILTER keep precipitate |
| 6 | PURIFY | WASH with acetic acid |
| 7 | YIELD 4-(difluoromethyl)-3-phenyl-1H-indazol-6-ol | DRYSOLID under vacuum |
| 8 |  | YIELD 4-(difluoromethyl)-3-phenyl-1H-indazol-6-ol |

---

```
Reaction no 416
```

Generated by the Chemistry Development Kit (http://github.com/cdk)

|  | A | B |
| --- | --- | --- |
| 0 | ADD (5-Bromo-9H-thioxanthen-2-yl)-carbamic acid tert-butyl ester | ADD (5-Bromo-9H-thioxanthen-2-yl)-carbamic acid tert-butyl ester |
| 1 | ADD bis(pinacolato)diboron | ADD bis(pinacolato)diboron |
| 2 | ADD potassium acetate | ADD Pd(dppf)Cl2 |
| 3 | ADD dioxane | ADD 1,1′-bis(diphenylphosphino)ferrocene |
| 4 | ADD Pd(dppf)Cl2 | ADD potassium acetate |
| 5 | ADD 1,1′-bis(diphenylphosphino)ferrocene | ADD dioxane |
| 6 | STIR for 86400 s at 100 °C | STIR for 86400 s at 100 °C |
| 7 | PURIFY | CONCENTRATE |
| 8 | YIELD [5-(4,4,5,5-Tetramethyl-[1,3,2]dioxaborolan-2-yl)-9H-thioxanthen-2-yl]-carbamic acid tert-butyl ester | PURIFY |
| 9 |  | YIELD [5-(4,4,5,5-Tetramethyl-[1,3,2]dioxaborolan-2-yl)-9H-thioxanthen-2-yl]-carbamic acid tert-butyl ester |

---

```
Reaction no 417
```

Generated by the Chemistry Development Kit (http://github.com/cdk)

|  | A | B |
| --- | --- | --- |
| 0 | ADD 6-fluoro-1-phenyl-1H-4,2,1-benzoxathiazine 2,2-dioxide | ADD 6-fluoro-1-phenyl-1H-4,2,1-benzoxathiazine 2,2-dioxide |
| 1 | ADD THF | ADD THF |
| 2 | ADD LiHMDS at -70 °C | SETTEMPERATURE -70 °C |
| 3 | STIR for 3600 s at -70 °C | ADD LiHMDS |
| 4 | ADD allyl bromide | ADD THF |
| 5 | STIR for 86400 s at 25 °C | STIR for 3600 s at -70 °C |
| 6 | QUENCH with ammonium chloride | ADD allyl bromide |
| 7 | EXTRACT with ethyl acetate | STIR for 86400 s at 25 °C |
| 8 | COLLECTLAYER organic | CONCENTRATE |
| 9 | DRYSOLUTION over MgSO4 | ADD dichloromethane |
| 10 | FILTER keep filtrate | EXTRACT with HCl |
| 11 | CONCENTRATE | COLLECTLAYER organic |
| 12 | PURIFY | DRYSOLUTION over MgSO4 |
| 13 | YIELD 3-allyl-6-fluoro-1-phenyl-1H-4,2,1-benzoxathiazine 2,2-dioxide | CONCENTRATE |
| 14 |  | PURIFY |
| 15 |  | YIELD 3-allyl-6-fluoro-1-phenyl-1H-4,2,1-benzoxathiazine 2,2-dioxide |

---

```
Reaction no 418
```
[truncated: 102,371 more chars]
